# Synthetic Surrogates of Collagen-Rich Microenvironments: Integrating Modular Bioactive Fibrillar Structure and Tunable Viscoelasticity via Multifunctional Assembling Peptides

**DOI:** 10.1021/acscentsci.5c02175

**Published:** 2026-05-16

**Authors:** Rafael A. Castro, Lina Pradhan, Jeffrey Caplan, Caitlin D’Ambrosio, April M. Kloxin

**Affiliations:** † Department of Chemical and Biomolecular Engineering, 5972 University of Delaware, 150 Academy Street, Newark, Delaware 19716, United States; ‡ Department of Plant and Soil Sciences, University of Delaware, 531 South College Avenue, Newark, Delaware 19716, United States; § Delaware Biotechnology Institute, University of Delaware, 590 Avenue 1743, Newark, Delaware 19713, United States; ∥ Department of Materials Science and Engineering, University of Delaware, 127 The Green, Newark, Delaware 19716, United States

## Abstract

Collagen imparts structure, viscoelasticity, and bioactivity to the extracellular matrix (ECM) with variance between organs and between healthy and diseased states. Synthetic mimics of collagen-rich tissues remain a need in applications, from biological studies to regenerative medicine, for parsing and controlling these properties. We designed multifunctional collagen mimetic peptides (mfCMPs) that self-assemble into triple helices and fibrils *and* contain integrin binding motifs: GFOGER, a binding site within intact collagen I, and RGD, a cryptic binding site available within denatured collagen I. These mfCMPs are incorporated into hydrogel-based synthetic ECMs to impart collagen-like hierarchical structures, viscoelasticity, and bioactivity with modularity. We establish innovative methods for imaging the resulting nano- and micro-structures within the hydrogel using super-resolution microscopy. The physically assembled mfCMPs impart tunable, concentration-dependent viscoelasticity within otherwise elastic, covalently cross-linked hydrogels, exhibiting relaxation half times over orders of magnitude and similar to soft tissues. Notably, breast cancer cells encapsulated and cultured in synthetic ECMs with a bioactive fibrillar structure and viscoelastic properties formed large, growing spheroids. Overall, these modular building blocks provide an innovative tool for creating fully synthetic surrogates of collagen-rich microenvironments, aiding both fundamental and translational biological studies and providing a framework to imbue tunable viscoelasticity to synthetic ECMs.

## Graphical abstract

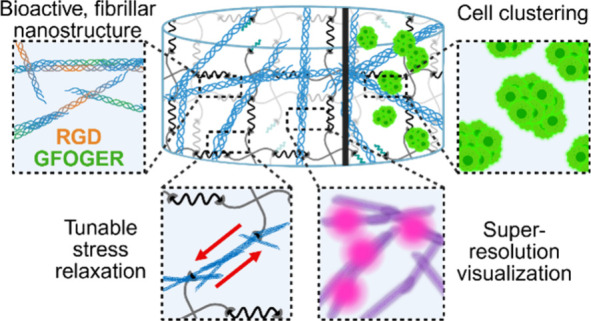

## Introduction

Advances in biocompatible, tunable, and responsive soft material systems have enabled researchers to study how cells are affected by their surroundings in both healthy and diseased microenvironments.^1,2^ Hydrogels, water-swollen polymer networks, are a common starting point when selecting a material system for 3D cell culture experiments. A range of hydrogel designs have been shown to serve as versatile cell culture platforms for bridging the biological complexity between animal models and tissue-culture plastic models. Recently, the importance of viscoelasticity within these systems and its regulation of cell spreading,^3,4^ differentiation,^5,6^ proliferation,^7^ and organoid formation^8^ has emerged. Despite advances, there remains a great need for molecularly engineered building blocks and innovative approaches to independently control viscoelasticity without altering the synthetic matrix stiffness or its inherent biochemical cues. The ability to decouple and control these pivotal properties is needed for both hypothesis testing and accurately guiding cellular behavior.

Viscoelastic materials have properties of both solids (storage of mechanical energy) and fluids (dissipation of mechanical energy).^9–11^ Viscoelastic materials also exhibit stress relaxation, a time-dependent decrease in stress in response to a constant strain. Stress relaxation can be characterized by the relaxation half time (τ_1/2_), or the amount of time it takes for 50% of the original stress to be dissipated, with a faster time corresponding to higher viscoelasticity.^9^ For many healthy soft tissues, τ_1/2_ often ranges from ∼10–10000 s.^7,9^ However, many malignant tissues display higher viscoelasticity compared to benign or normal tissue;^12–14^ for example, human breast tumors are reported to have a τ_1/2_ ∼ 10 s.^7^ The viscoelastic effects of the cancer microenvironment on cells are also a growing area of research. Pancreatic cancer cells have shown a higher degree of proliferation and larger spheroid diameter in fast-relaxing hydrogels (τ_1/2_ ∼ 10^1^ s) compared to slow-relaxing hydrogels.^15^ Many proteins are responsible for the viscoelastic behavior of the extracellular matrix (ECM) through the breaking of weak cross-links^16–18^ and protein unfolding.^11,19^ Notably, collagen allows for native tissues to exhibit viscoelasticity as adjacent fibrils slide past one another, breaking and reforming physical cross-links after physical deformation.^20,21^

Collagen I is the most abundant (up to 90%) type of collagen found in connective and other soft tissues.^22^ This assembled protein provides cells with a physically cross-linked network of fibrillar structures on which they can move using the molecular clutch model of mechanotransduction and engagement of integrins.^23^ Accordingly, stiffness and viscoelasticity of the matrix are both crucial physical properties that influence cell behavior.^24^ For example, mesenchymal stem/stromal cells encapsulated in viscoelastic hydrogels showed more spreading, clustering, and nascent protein deposition in compositions with higher degrees of stress relaxation.^25^ This structural ECM function of collagen I can be traced back to its amino acid structure. Collagen has the repeat structure (X-Y-G)_n_, where X and Y can be any amino acid residue (most commonly proline (P) and hydroxyproline (O) in humans, respectively) and G is a glycine residue.^26^ This triplet repeat allows for peptide assembly into a triple helix (tropocollagen) conformation when three polyproline type IIhelices join through hydrogen bonding. These triple helices further assemble into hierarchical structures like fibrils (nanoscale) and fibers (microscale) by the oxidation of lysine and hydroxylysine residues in the telopeptide domains during fibrillogenesis.^26–28^ These biophysical structures and their dynamics are important to capture in systems for studying cellular responses in 3D collagen-rich microenvironments.

Differences in collagen content have been found between healthy microenvironments compared to diseased, cancerous, or fibrotic microenvironments, where the structure, density, and cross-linking of collagen not only impacts the biophysical properties of these microenvironments but also their biochemical properties.^29–31^ For example, upregulated collagen I production in mammary tissue as part of the wound healing response results in a stiffness of ∼5 kPa compared to the softer, healthy tissue with a stiffness of ∼0.5 kPa, of relevance in both injury and disease including cancer.^32^ Further, in healthy microenvironments, cells can biochemically interact with collagen I through integrin binding sequences such as GFOGER that are displayed by intact, assembled collagen fibrils for integrin (α_2_β_1_) binding.^33^ However, in remodeled and diseased microenvironments, denaturation or breaking of these collagen fibrillar structures reveals other integrin binding sequences, such as RGD. The RGD sequence is found in denatured collagen, as well as in fibronectin among other ECM proteins, and as fibrillar denaturation occurs, cryptic RGD binding sites are exposed to be readily available for integrin (α_5_β_1_ and α_v_) binding.^34–36^ An opportunity lies in the design of collagen mimetic systems that enable the controlled presentation of these binding sites from fibrillar structures within synthetic ECMs toward representing aspects of both intact and denatured collagen I and progress toward a fully synthetic mimic of collagen-rich tissues.

In this work, we aimed to capture the structural, biophysical, and bioactive complexity of the native collagen-rich ECMs in a completely synthetic hydrogel system, allowing for a high degree of tunability in both viscoelasticity and bioactivity and for representing a range of collagen-rich microenvironments. Here, we built upon a base multifunctional collagen mimetic peptide (mfCMP) design that self-assembles into fibrillar structures, much like natural collagen I, and allows light-triggered covalent cross-linking with polymer macromers as well as orthogonal modification using azide–alkyne chemistry.^37^ We first hypothesized that relevant integrin binding sequences, GFOGER for intact collagen and RGD for denatured collagen, could be incorporated within mfCMPs without disrupting their multiscale assembly. To test this, we designed two new sequences with the integrin-binding unit within a lengthened hydrogen bonding (POG)_n_ block, utilizing computational approaches to inform and visualize the resulting structures. Triple helix formation and stability was examined with circular dichroism (CD) spectroscopy, elucidating multiple melting events at biologically relevant temperatures. For probing fibrillar assembly, an innovative technique was developed to allow for super-resolution imaging, revealing hierarchical mfCMP structures within photo-cross-linked polymer–peptide synthetic ECMs. We next hypothesized that the self-assembled nature of the mfCMP could be leveraged to impart viscoelasticity to these hydrogel-based synthetic ECMs by increasing the number of physical cross-links within the covalently cross-linked network. Importantly, with increasing mfCMP content, faster and tunable stress relaxation was observed as the percentage of cross-links in the hydrogel network shifted from irreversible covalent linkages to reversible physical mfCMP linkages. To evaluate the effect of the resulting properties of this system on cellular responses, breast cancer cells were encapsulated in stress-relaxing, bioactive mfCMP ECMs and exhibited the formation of large, growing cell clusters with limited cluster formation in traditional elastic polymer–peptide materials. Overall, this work establishes a collagen-inspired, fully synthetic ECM with self-assembled structures that impart both tunable bioactivity and viscoelasticity for controlling 3D cellular microenvironments, as well as establishes a unique framework of assembled cross-links to imbue stress relaxation within synthetic ECMs.

## Results and Discussion

### Design of Integrin Binding mfCMP Sequences

Toward creating a fully synthetic collagen-like ECM, we first incorporated bioactivity into the fibrillar architecture of mfCMP hydrogel networks rather than stochastically throughout the bulk network using pendent integrin-binding peptides as done traditionally in polymer–peptide synthetic ECMs. To achieve this, we designed new mfCMP variants containing the integrin-binding motifs GFOGER (found in collagen I^33^) and RGD (found in fibronectin and denatured collagen I^36^): mfCMPa-G-az, K­(azide)­G­(PKG)_4_PK­(alloc)­G­(PO**G**)_6_ **FOGER**G­(POG)_6_(DOG)_4_, and mfCMPa-R-az, K­(azide)­G­(PKG)_4_PK­(alloc)­G­(POG)_6_G**RGDSP** (POG)_6_(DOG)_4_ (Figure 1). These new sequences were based on our previous design^38^ mfCMPa-az, K­(azide)­G­(PKG)_4_PK­(alloc)­G­(POG)_6_(DOG)_4_, and now incorporate the integrin binding sequences *within* a lengthened hydrogen-bonding block [(POG)_n=6→12_] to ensure presentation from the triple helix, which previous works have shown important for integrin binding and retention of structure.^39,40^ These mfCMPs also include (i) charge-paired (PKG)_n_ and (DOG)_n_ blocks for elongation of fibrils through end-to-end electrostatic interactions and functional handles for orthogonal reactions, (ii) an alloc-protected lysine for covalent cross-linking by photoinitiated thiol–ene click chemistry, and (iii) an azide-functionalized lysine for in situ modification and labeling by azide–alkyne click chemistry. We hypothesized that integrin binding sequences could be integrated within mfCMP designs while maintaining these properties by lengthening the H-bonding (POG)_n_ block. These designs were rapidly synthesized by microwave-assisted solid phase peptide synthesis (SPPS), purified by heated reverse phase high performance liquid chromatography (HPLC) followed by dialysis, and the sequence identity verified by ultraperformance liquid chromatography-tandem mass spectrometry (UPLC-MS) ().

**Figure 1:**
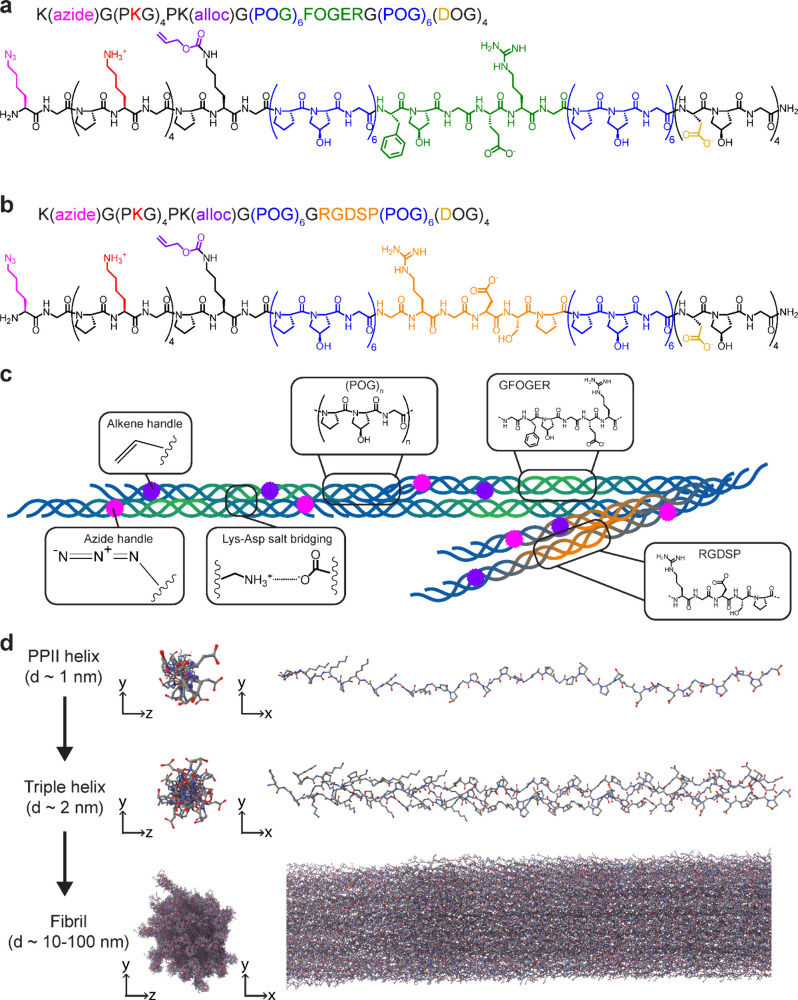
mfCMP hierarchical structure. Amino acid sequence and primary structure for a) mfCMP-G-az and b) mfCMP-R-az. c) Schematic of mfCMP functionality. (POG)_n_ blocks stabilize the triple helix; integrin binding sequences are incorporated into mfCMP amino acid structure; salt-bridging between (PKG)_n_ and (DOG)_n_ blocks promotes fibrillar formation; alkene and azide groups serve as orthogonal reactive handles. d) Visual representations of hierarchical mfCMP assemblies. Individual peptides have a right-handed polyproline type II conformation, which then assemble into a triple helix. These triple helices then assemble into higher-order fibrils and fibers. AlphaFold 3 ^42^ was used to produce representations of the α-helix and triple helix, and ColBuilder^43^ was used for the microfibril representation.

Despite the long sequences of amino acids comprising these peptides (up to 71 residues), yields relevant for construction of materials were achieved with this approach: mfCMPa-G-az, 33.6% yield; mfCMPa-R-az, 32.2% yield; and mfCMPa-az, 39.8% yield. Note, while we observe consistent properties batch to batch as detailed below, multiple peaks were observed in UPLC traces of these peptides because of minor impurities due to amino acid residue deletions. Specifically, one common side product is the PO deletion, where one group of adjacent P and O residues are not coupled during synthesis of these long sequences with multiple (POG) repeats as previously reported for mfCMP designs.^41^ Other minor impurities also are observed that are not easily identifiable, which we speculate are due to side chains of amino acid residues that react during the lengthy synthesis and purification process. To attempt to separate the desired mfCMP peptides from these impurities, we used a slow gradient during HPLC purification (∼0.7% acetonitrile per minute) along with a column heater set to 65 °C. While slower HPLC gradients were tried after this, further separation was not achieved (), and we speculated that the challenges in purification were due to assembly or aggregation of the peptides. To probe this, dynamic light scattering (DLS) measurements were performed on mfCMPs dissolved in HPLC solvent (), and different size scales of aggregates were observed at both 25 and 65 °C. Despite the challenges that this aggregation presents during purification, we observe that the majority product is the desired mfCMP molecular weight via UPLC-MS, and these mfCMPs reproducibly assemble into triple helices and fibrils and tune the properties of hydrogels built with them, as described below.

After the synthesis and purification of these new integrin-binding mfCMPs, we next verified their triple helix formation using CD spectroscopy. Wavelength scans show a characteristic polyproline type II peak at 225 nm associated with a collagen-like triple helix^44^ for both mfCMPa-G-az and mfCMPa-R-az, as well as the mfCMPa-az control (Figure 2a–c). Increasing the temperature decreased the magnitude of the characteristic peaks nonlinearly for all the mfCMPs, indicating that triple helices dissociated and ‘melted.’ Both mfCMPa-G-az and mfCMPa-R-az showed peaks with a higher magnitude compared to the control mfCMPa-az, supporting the stabilizing effect of the increased number of (POG)_n_ repeats in the integrin-binding mfCMPs compared to the control. Note, in the central bioactive area of these peptides, mfCMPa-G-az keeps every third residue as glycine, while mfCMPa-R-az contains a proline residue in the RGDSP motif that falls in the G position of the (X-Y-G) structure, decreasing the relative stability of the triple helix. Indeed, mfCMPa-G-az showed the highest magnitude of 225 nm peaks, potentially imparted by the hydrogen bonding within the GXYGER sequence^45^ in addition to the consistent staggering of (X-Y-G) sequence relative to mfCMPa-R-az. Future peptide designs could substitute the proline with a glycine for a simplified motif of RGDS toward increasing the triple helix stability.

**Figure 2:**
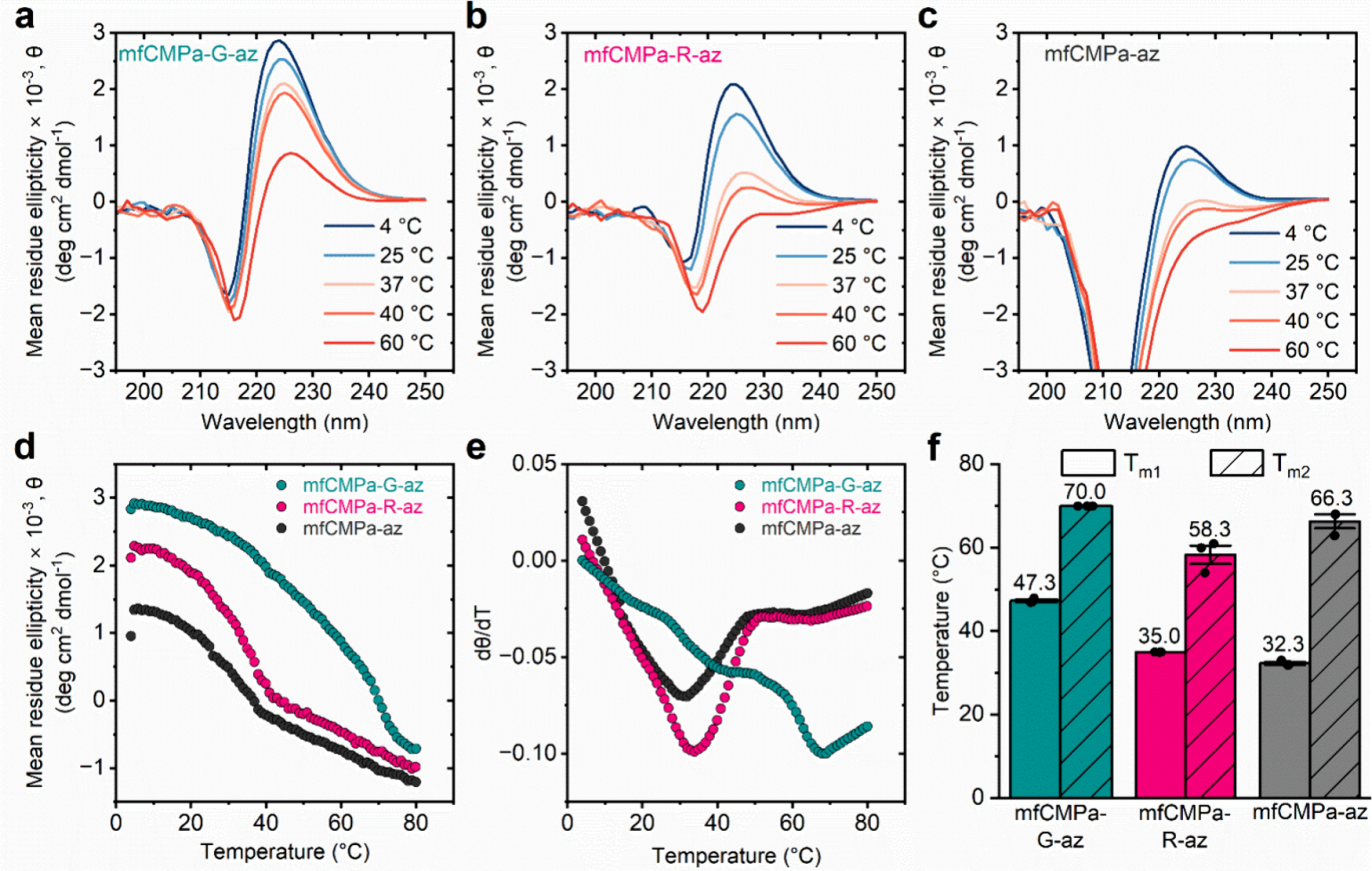
Triple helix characterization of mfCMPs in Dulbecco’s phosphate-buffered saline by CD spectroscopy. Wavelength scans of a) mfCMPa-G-az, b) mfCMPa-R-az, and c) mfCMP-az as temperature is ramped from 4 to 80 °C show the characteristic polyproline type II peak (225 nm). d) Temperature scans of mfCMPs at 225 nm show nonlinear “melting” as temperature increases, indicating that triple-helical conformations are present. e) First-order derivative curves of d) show minima that describe melting events for mfCMP triple helices. f) Temperatures of melting events of mfCMPs. The two largest melting events are reported for each sequence as determined by the inflection points of (d), identified by analysis of second-order derivatives of the data in (d) (). Results shown are from a representative sample of multiple trials (*n* = 3). All replicates are shown in .

To further quantify triple helix stability, we performed CD temperature scans for each mfCMP at 225 nm with a temperature ramp from 4 to 80 °C, creating ‘melting’ curves (Figure 2d). For these assembling peptide sequences, these melting curves are measuring how ordered the polyproline type II helix is due to the triple helical conformation and are performed at lower concentrations (0.3 mM) than used in hydrogel formulations to avoid the formation of higher ordered structures that would scatter light and impede CD measurements (e.g., fibrils formed by sticky end interactions). The first- and second-order derivatives of these curves were then taken to identify inflection points as melting events (Figure 2e,f). All mfCMPs showed a first melting event (*T*_m1_) near 37 °C, indicating that around 50% of the triple helices remained intact at this temperature, like natural collagen I.^46,47^ The percentages of assembled triple helices at 37 °C for mfCMPa-G-az, mfCMPa-R-az, and mfCMPa-az were estimated to be 79.6 ± 0.211%, 47.5 ± 0.213%, and 46.2 ± 0.172%, respectively. These values were calculated by dividing the mean residue ellipticity at 37 °C by the difference between the maximum and minimum mean residue ellipticities for each melting curve. Secondary melting events (*T*_m2_) are shown for all mfCMPs, possibly due to the dissociation of one end of the triple helix at a lower temperature before the other end at a higher temperature.^48^ In particular, differences in the strength of intrahelical interactions between (PKG)_n_ and (DOG)_n_ blocks have been postulated to produce multiple melting events in other mfCMP designs.^49^ Note, below 215 nm, high tension voltages were above 800 V for most mfCMPs measured; as these values are above the highest acceptable value of 600 V, we attribute shifts at 215 nm to noise produced at these high voltages.^50^

### Visualizing mfCMP Fibrillar Architecture in Covalent Hydrogels

We incorporated the integrin-binding mfCMPs into polymer–peptide hydrogels covalently to introduce bioactive, fibrillar structure. Using a four-arm PEG-SH as the polymer macromer (10 wt %, 20 mM thiols), hydrogels were formed using the cell-degradable linker peptide sequence KK­(alloc)­GGPQG↓IWGQGK­(alloc)K (13 mM alloc functional handles), alloc-functionalized mfCMP (5 mM), and pendent alloc-functionalized integrin-binding peptide K­(alloc)­GWG**RGDS** (2 mM) by thiol-ene click chemistry after irradiation with cytocompatible doses of UV light (365 nm) and the photoinitiator lithium phenyl-2,4,6-trimethylbenzoylphosphinate (LAP; 2.2 mM). In this hydrogel formulation, thiol:alloc functional handles were kept equal, and separate hydrogels were made for the integrin-binding mfCMP-G-az and mfCMP-R-az, as well as the control mfCMPa-az. Separate negative control hydrogels were also made without any mfCMP. Linker and pendant peptides were purified by HPLC and characterized by UPLC-MS (). Note these non-mfCMP peptides show some minor impurities as described above for the mfCMPs.

To visualize mfCMPs in hydrogels, we exploited their azide reactive handles and labeled them using fluorophores. First, cover glasses were washed, dried, and coated with poly-l-lysine for increased hydrophilicity, so that hydrogels would adhere tightly to the glass. After photo-cross-linking of hydrogels directly on cover glasses, we let them equilibrium-swell in Dulbecco’s phosphate-buffered saline (DPBS) for 12 h in a nontreated 6-well plate. mfCMPs covalently incorporated in the hydrogels were then labeled with alkyne-functionalized AlexaFluor fluorescent molecules through copper-catalyzed azide–alkyne cycloaddition (CuAAC) in the well plate. Briefly, reagents were diluted into the wells containing hydrogels on cover glasses, and then the entire plate was gently shaken for an hour, followed by two 30 min washes and one final wash overnight. The hydrogels were then stored in fresh DPBS and imaged the following day. With a 63× oil lens objective, confocal microscopy was first used to visualize AlexaFluor 488-labeled fibrillar mfCMP structures in the hydrogel (). The images were deconvolved by using Huygens software to improve the apparent resolution and signal-to-noise ratio of the images. Fibrils appear randomly throughout the hydrogel in terms of distribution and orientation given the mixing approach followed by rapid photo-cross-linking, which was desired for mimicking collagen-rich, loose connective tissues.

To better resolve the mfCMP fibrillar structures in the hydrogels, we used super-resolution imaging techniques. Direct stochastic optical reconstruction microscopy (STORM) is a super-resolution imaging technique that localizes individual fluorophores in a set of images over time. This allows for a much higher resolution (20–60 nm) to be obtained from fluorescence microscopy images.^51^ A small portion of the sample is excited with a high-intensity laser, driving the fluorophores into a dark state. This leads to stochastic blinking of individual fluorophores as they transition between their fluorescent and dark states (Figure 3a).^52^ Each blinking event is associated with the release of photons from a single fluorophore, and many total internal reflection fluorescence microscopy (TIRFM) images of the exposed area are rapidly collected. Blinking events are localized using the Picasso software^53^ and then rendered into a single image. Here, we labeled the mfCMPs fibrils in hydrogels with AlexaFluor 647, a cyanine dye that is optimal for this technique,^54^ and deployed STORM to generate high resolution images that confirm retention of the fibrillar assembly with integrin-binding mfCMPa-G-az and mfCMPa-R-az synthetic ECMs, as well as the mfCMPa-az control (Figure 3). The structures were consistent with observations of the different mfCMPs imaged using transmission electron microscopy (TEM) (assembled at 1 mM, diluted to 0.3 mM, cast on copper grids, counterstained with uranyl acetate), with lengths on the order of microns and widths on the order of hundreds of nanometers (), *and* consistent with natural collagen that contains fibrils with diameters from around 10 to 500 nm.^26,55,56^

**Figure 3:**
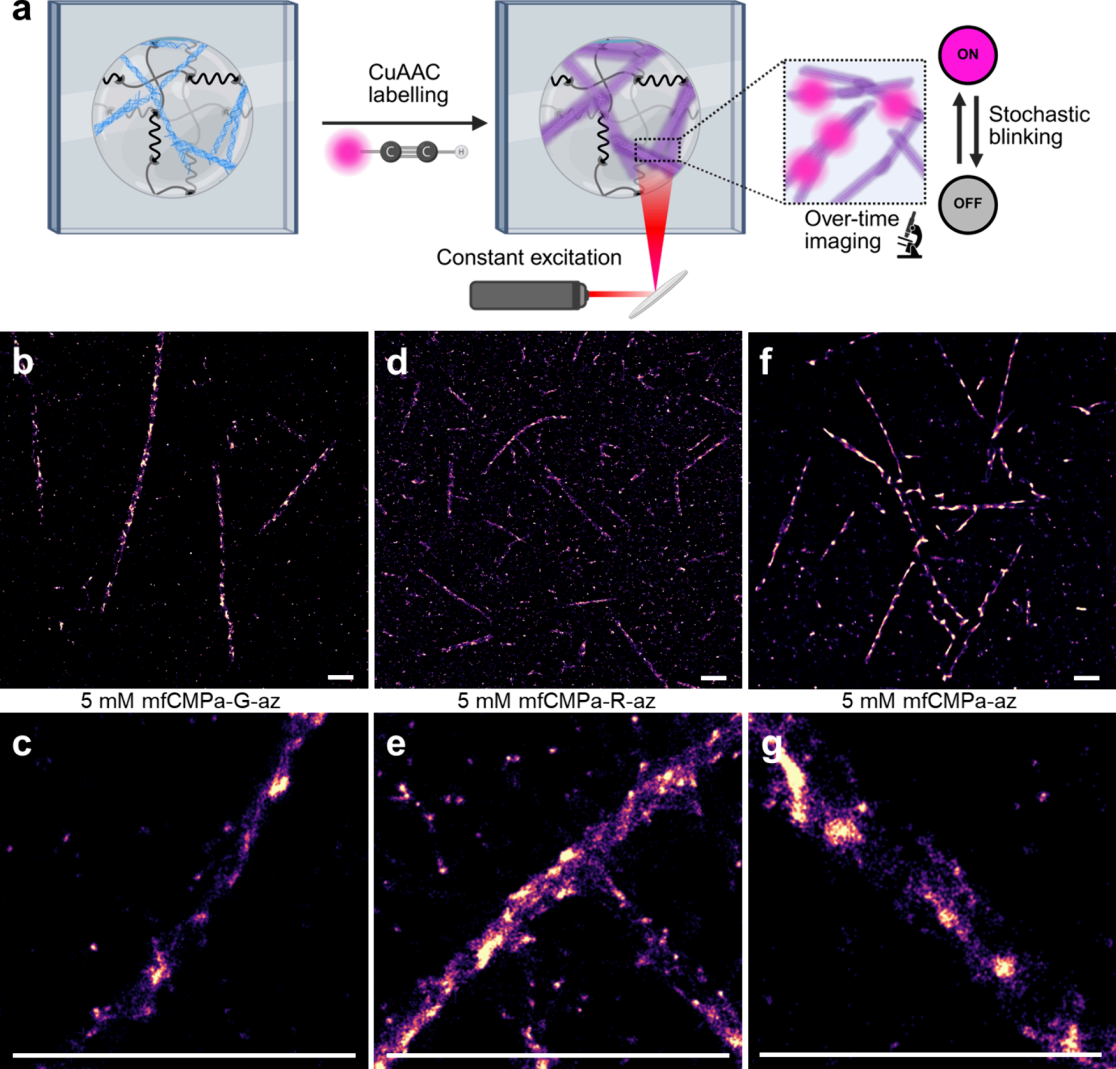
mfCMP visualization in hydrogels with STORM imaging. a) Schematic showing the approach to form hydrogels with mfCMPs on charged cover glasses followed by labeling with a fluorophore for STORM imaging. Samples are excited with a high-power laser on a small area, then imaged over time. This results in the stochastic blinking of fluorophores as they alternate between fluorescent “on” and dark “off” states. Blinking events captured in images over time are processed to localize singular fluorophores, which are then rendered to produce the final images. Images of hydrogels containing b, c) mfCMPa-G-az, d, e) mfCMPa-R-az, or f, g) mfCMPa-az. Scale bar = 2 μm.

STORM images were used to quantify fibril lengths and widths (). Notably, mfCMPa-R-az showed significantly reduced lengths and widths compared to the other mfCMPs. We hypothesize that this difference is due to the instability to its hierarchical structure introduced by the proline residue in the RGDSP domain disrupting the secondary and tertiary structures of the mfCMP-R-az as described above in the melting temperature analysis (Figure 2). Negative control hydrogels without any mfCMP did not show any fibrillar structure (). Hydrogels with higher (13 mM) concentrations of mfCMP were also imaged using STORM (), showing a higher density of fibrils for each mfCMP. Note that 20 mM mfCMP hydrogels could not be imaged using STORM owing to their loss of integrity with equilibrium swelling and sample processing in preparation for STORM imaging.

### Probing the Viscoelasticity of mfCMP-PEG Hydrogel Networks

Assembled collagen I units within native tissues slide past each other in response to applied stress, imparting viscoelasticity from the molecular to the tissue level^19,20^ and representing an important feature that synthetic microenvironments need to capture. A variety of cross-linking strategies have been implemented in systems for controlled cell culture to create different types of dynamic, viscoelastic hydrogels using building blocks that are harvested, synthetic, or both:^57^ for example, calcium-cross-linked, alginate-based hydrogels;^4,58,59^ hydrogen-bonded, methacrylic acid-methacrylamide supramolecular hydrogels;^60^ or hydrogels integrating dynamic covalent chemistries.^61–63^ We hypothesized that hydrogels with mfCMPs would exhibit stress relaxation because of the collagen-like physical cross-links between mfCMP peptides and fibrils (i.e., hydrogen bonding and sticky-end electrostatic interactions), allowing fibrils to slide past each other to dissipate stress induced by applied strain (Figure 4a). In the hydrogel, assembled mfCMPs are covalently cross-linked into the polymer network, and the mfCMPs then serve as physical cross-links within the network owing to their physical interactions with each other via hydrogen bonding within triple helices and salt bridging within fibrils. The dynamics of these physical cross-links have the potential to impart bioinspired, tunable viscoelasticity and provide a new complementary mechanism for modulating synthetic matrix properties.

**Figure 4:**
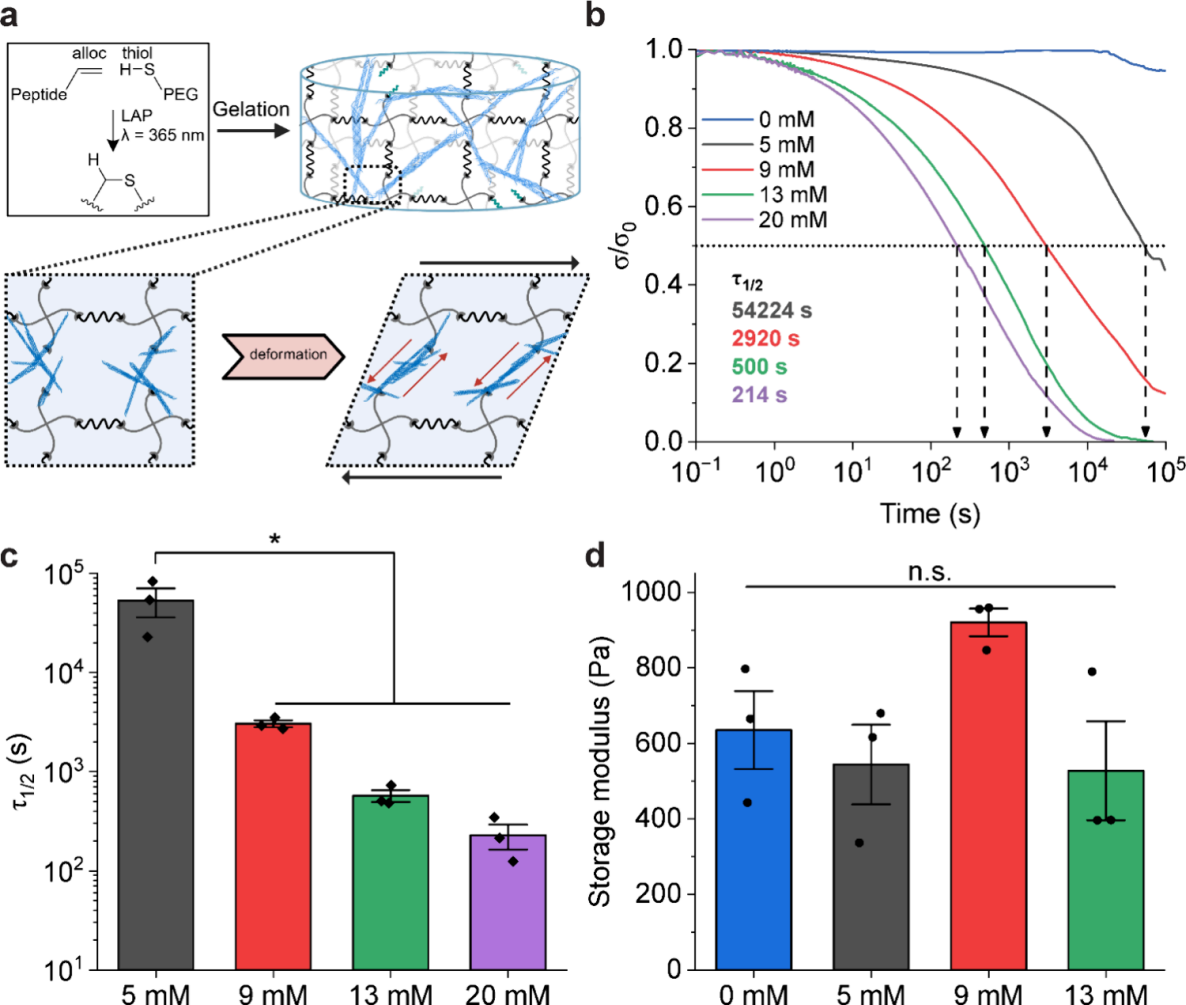
Viscoelasticity of the mfCMP-PEG hydrogels. a) Hydrogel photo-cross-linking schematic and proposed mechanism of stress relaxation in mfCMP-PEG hydrogels in response to a deformation: physical cross-links relax as the network is deformed and mfCMP fibrils slide past each other, dissipating mechanical energy. b) In situ stress relaxation profiles of representative hydrogels that contain increasing concentrations of mfCMPa-az. All replicates shown in . c) τ_1/2_ of hydrogels that contain increasing concentrations of mfCMPa-az. d) Equilibrium-swollen storage moduli of hydrogels containing 0 mM, 5 mM, 9 mM, and 13 mM of mfCMPa-az. Means ± standard error for each condition are shown for (*n* = 3) independent sample measurements. Statistical significance was determined by one-way ANOVA with Tukey’s multiple comparisons test. Statistical significance is shown (*p < 0.05; n.s. *p* > 0.05).

To test the viscoelasticity of our mfCMP-PEG hydrogel system, we performed shear stress relaxation tests on hydrogels formed in situ on a rheometer. Strain was first ramped from 0 to 15% over 2 s (2 s rise time to minimize noise at the start of the measurement^64^), then held constant at 15% for each sample for 10^5^ seconds. This strain was determined to be in the linear viscoelastic (LVE) region for the hydrogels in earlier tests (). We chose to perform stress relaxation experiments first on a mfCMP design (mfCMPa-az) and hydrogel formulation that had been shown previously to influence cellular responses (mesenchymal cell morphology and motility^37^) to understand its stress relaxation profile and probe if stress relaxation was imparted by the inclusion of mfCMP, which we had not previously examined.

Different hydrogel compositions were tested with increasing amounts of mfCMPa-az: 0 mM, 5 mM, 9 mM, 13 mM, and 20 mM, and decreasing amounts of cell-degradable linker. In the formulation with 0 mM mfCMP, we hypothesized that this hydrogel would show elastic behavior since the network is completely made of irreversible covalent bonds between the cell-degradable linker peptide and PEG backbone and, accordingly, would show no stress relaxation. As seen in the results (Figure 4b), this was confirmed to be the case: in the representative sample shown, there was only ∼3% stress relaxation after the duration of the test. Further, in the formulation with 5 mM mfCMP, the concentration that has been used by our group in previous studies,^37,38^ hydrogels showed a τ_1/2_ of 53400 ± 17400 s, indicating that the network has viscoelasticity being imparted by the mfCMPs. When higher concentrations of 9 mM, 13 mM, and 20 mM mfCMP were incorporated in the network, the resulting τ_1/2_ are 3050 ± 242, 573 ± 78.5, and 228 ± 64.3 s, respectively (Figure 4c).

These data () were then fitted () with a generalized Maxwell model with three modes (), a viscoelastic model that describes three stress relaxation events in parallel.^65^ The fitted parameters describing the stress, *A*_*i*_, and relaxation time, *τ*_*i*_, for each were determined (). This model fitting suggests that there are at least three modes of relaxation in our PEG-mfCMP hydrogels. We hypothesize that faster modes may be associated with the relaxation of the triple helix or fibril mfCMP assemblies in response to a deformation, and slower modes may be associated with a poroelastic effect of the network,^9^ with opportunities to test this mechanistic hypothesis of the origin of the modes in future studies. After swelling, a similar increase in volume is seen in all conditions, decreasing the moduli to similar levels, where the equilibrium-swollen storage moduli of the hydrogels containing 0, 5, 9, or 13 mM mfCMPa-az were statistically similar and remained in the range 500–1000 Pa (Figure 4d). These results support that mfCMPs impart concentration-dependent stress relaxation to otherwise elastic materials as the percentage of cross-links in the hydrogel network shifts from irreversible covalent linkages to reversible physical mfCMP linkages. Note that the equilibrium storage moduli of hydrogels containing 20 mM mfCMPa-az could not be measured owing to their loss of integrity with equilibrium swelling and sample processing.

Based on the viscoelastic behavior we observed in hydrogels with mfCMPa-az, we then tested the hypothesis that integrin-binding peptides could be presented from the mfCMP while still achieving similar stress relaxation profiles. Indeed, hydrogels containing 5 mM mfCMPa-G-az and mfCMPa-R-az showed viscoelastic behavior similar to mfCMPa-az (). For hydrogels containing mfCMPa-az, viscoelastic behavior was also seen as a crossover point between the storage and loss moduli in shear strain sweep tests, suggesting strain yielding. However, no crossover points were present in the frequency sweeps as is seen in some types of viscoelastic materials.^66^

### Exploring Cellular Response to mfCMPs

With the ability to tune viscoelasticity in the mfCMP-PEG synthetic matrix, we next aimed to test if this would affect the response of encapsulated cells. Additionally, leveraging the bioactive mfCMP designs, we wanted to probe cell responses to integrin-binding sequence presentation from the mfCMP fibril versus throughout the bulk through a pendent group presentation from the polymer network. Well-defined 3D cell culture platforms remain a need for studies of cancer among other indications, where luminal A breast cancer cells comprise over 70% of all new breast cancer diagnoses^67^ and are known to be responsive to the matrix viscoelasticity and composition.^3^ The tumor microenvironment itself is rich in collagen I and fibronectin and undergoes constant remodeling. During tumor growth and disease progression, both collagen and fibronectin ECM content increase as a result of aberrant protein expression.^68,69^ Here, we chose to use T47D-GFP (green fluorescent protein), a human breast cancer cell line transduced for constitutive expression of GFP, to facilitate the characterization of breast cancer cell morphology and growth. In these studies, we wished to focus on the effects of the designed materials on cell proliferation and growth, and accordingly, selected luminal A breast cancer cells known for spheroid formation and limited migration,^70^ where we have previously observed the importance of β1 integrin-binding by RGDS and GFOGER in T47D cluster growth within elastic PEG-peptide hydrogels.^68^ We hypothesized that (i) the viscoelasticity imparted by mfCMPs to the hydrogel-based synthetic matrix and (ii) in addition to integrin-binding sequence presentation from the mfCMP fibrils would affect breast cancer cell cluster growth.

To form each hydrogel for these experiments, we took a dual-layer hydrogel approach, as illustrated in ,^68^ now integrating the mfCMP within the cell-laden top layer for 3D culture (Figure 5a). Here, we chose a total mfCMP concentration of 5 mM based on our previous studies showing this concentration was effective for promoting increased fibroblast speed and elongation.^37^ First, a bottom hydrogel layer (15 μL) was formed using the basic components of PEG-SH macromer (10 wt %, 20 mM thiols), cell-degradable linker peptide (13 mM), and desired pendent peptides (2 mM). Next, an additional layer (5 μL) was formed on top that contained basic components as well as assembled mfCMP (5 mM total mfCMP) and T47D cells. We used this dual-layer approach for a variety of reasons: to provide 1) a cushion between the cell-laden layer and tissue culture plastic (TCP) toward avoiding cells being influenced by the vastly higher modulus of the TCP; 2) increased homogeneity of cell responses throughout the cell-laden layer compared to a larger volume where mass transfer may become limited in thicker hydrogels; 3) easy handling of the hydrogels using the lower surface without disturbing the cells or breaking the hydrogels when moving between plates; and 4) an opportunity to reduce the amount of valuable mfCMP material and cells. The MMP-cleavable linker concentration (13 mM) was constant in all cases. Here, to probe for changes in cellular responses to the properties imparted by the mfCMPs, we tested three different hydrogel formulations (Figure 5b): (i) an elastic (E) formulation without any mfCMP and with pendent integrin-binding peptides (1 mM GFOGER and 1 mM RGD); (ii) a viscoelastic, fibrillar (VF) formulation with 5 mM mfCMPa-az and with pendent integrin-binding peptides (1 mM GFOGER and 1 mM RGD); and (iii) a viscoelastic formulation of bioactive mfCMP fibrils (VBF; 3 mM mfCMPa-az, 1 mM mfCMP-G-az, 1 mM mfCMP-R-az). Note that the pendent GFOGER peptide used also forms a stable triple helix characterized by CD with a *T*_m_ of 49.4 ± 0.4 °C (). Further, each hydrogel composition (E, VF, and VBF) was probed for viscoelastic behavior (), where the VF and VBF formulations showed significantly greater stress relaxation than the E formulation over a period of 10^5^ seconds. Additionally, these hydrogel compositions showed statistically similar swollen equilibrium storage moduli (). Together these rheometric measurements confirm that adding physical mfCMP cross-links imparts stress relaxation behavior while modulus and cross-link density are held constant.

**Figure 5:**
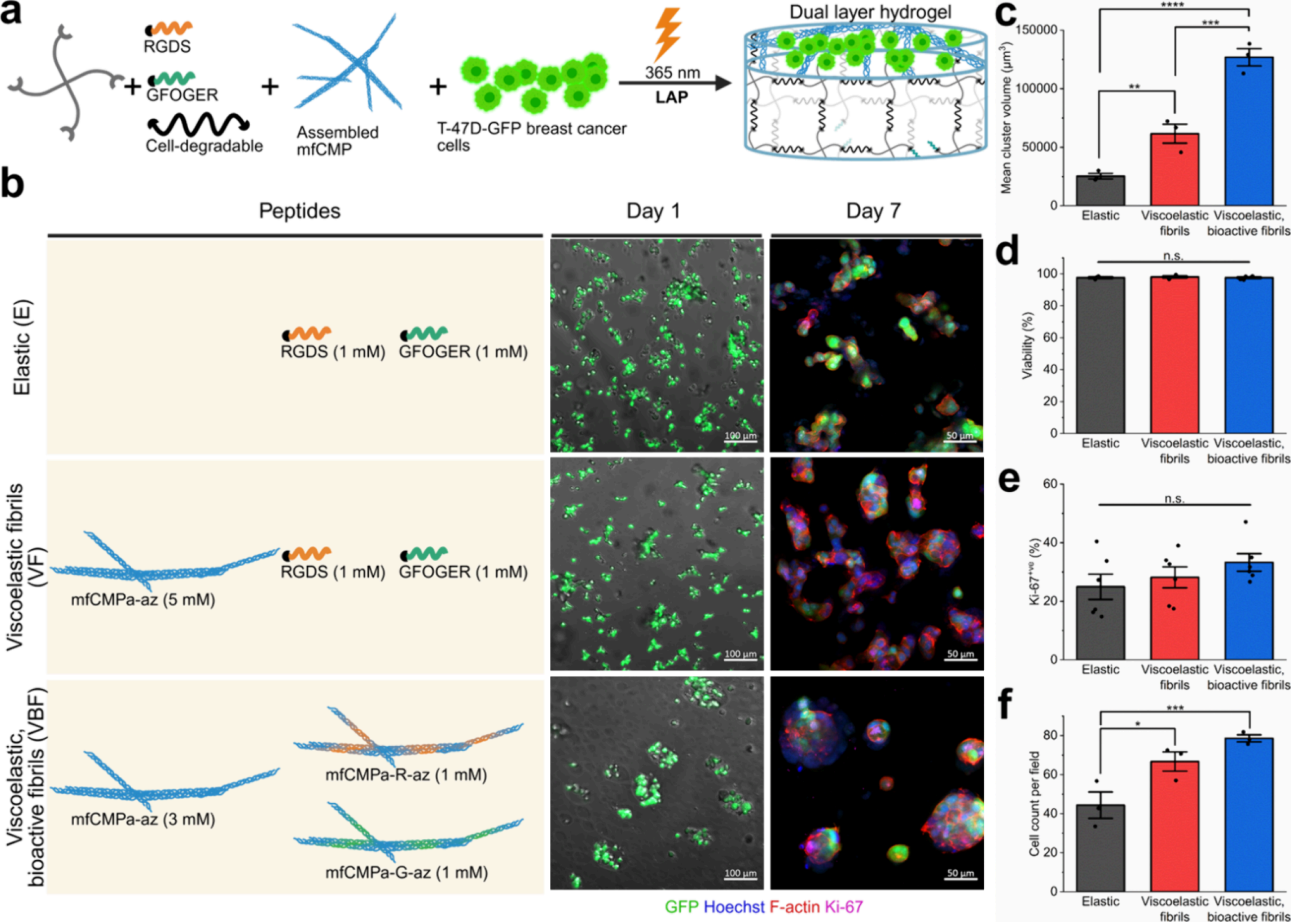
Cell encapsulation in the mfCMP-PEG hydrogels. a) Schematic of cell encapsulation where PEG-SH macromer and basic components are photo-cross-linked to form a larger bottom hydrogel layer. The same components plus mfCMP and cells are then photo-cross-linked on top to form the cell-laden layer. b) Hydrogel compositions include (i) an elastic formulation with pendent bioactive peptides; (ii) a viscoelastic composition with added mfCMP; and (iii) viscoelastic, bioactive fibrillar composition where the pendent integrin binding peptides are replaced by bioactive mfCMPs (total mfCMP concentration 5 mM). Representative images are shown on days 1 (live imaging) and 7 (fixed immunostaining). c) Mean cluster volume of cell objects imaged in mfCMP-PEG hydrogels based on F-actin cytoskeleton staining on day 7. d) Viability of cells after 7 days based on Hoechst (nuclear) and ethidium homodimer (dead cells) staining. e) Percentage of Ki-67 positive cells based on staining on day 7. f) Number of cells per field of view based counting of Hoechst positive cells in imaged, fixed immunostained gels. Means ± standard error are shown for each condition for (*n* = 3) independent sample measurements. Statistical significance was determined by one-way ANOVA with Tukey’s multiple comparisons test. Statistical significance is shown (*p < 0.05; **p < 0.01; ***p < 0.001; ****p < 0.0001).

After cell encapsulation (Figure 5a) on day 0, cellular responses were monitored over time with confocal microscopy (Figure 5b, ). Live images were captured after sufficient time for equilibrium swelling of the photo-cross-linked synthetic ECMs in real-time (∼8–22 h, ) and then on days 1, 4, and 7 for qualitative observations of cells and cell clusters. Metabolic activity was examined on days 1, 4, and 7 using the Alamar Blue assay. Cell viability was probed with ethidium homodimer and Hoechst nuclear staining on days 4 and 7. Cluster volume and growth were quantitatively examined by immunostaining of fixed samples, with labeling of F-actin, DNA, and either Ki-67, β1 integrin, or secreted ECM proteins.

From the real-time videos following the encapsulation, we observe little migration for this epithelial-like luminal A breast cancer cell type, which is expected for these spheroid-forming, weakly metastatic cells. Importantly, we found significant differences in mean cell cluster volume among the formulations (Figure 5c). In formulation E, after 7 days, the breast cancer cells formed small, uniform clusters (∼25000 ± 2400 μm^3^) throughout the 3D culture (top layer of the synthetic ECM). These results match the type of growth previously seen in similar hydrogel-based synthetic ECMs in prior studies by our group.^68^ In the VF condition, we observed clusters forming initially as before, but after 7 days, the cluster size was significantly larger (∼61000 ± 8100 μm^3^), suggesting cluster growth over time. Finally, in the VBF condition, the clusters were statistically the largest (∼127000 ± 7400 μm^3^) among the conditions probed. Interestingly, while collagen I and fibronectin deposition were observed under all conditions, increased fibronectin was observed under the VBF condition (). While quantitative differences in collagen I expression were not observed, localization of secreted collagen I within the pericellular matrix within these synthetic ECM is consistent with prior observations with other cell types.^71^

Integrin β1 was also stained under all conditions (). While β1 integrin is observed in all conditions, small puncta-like fluorescent regions were observed the in VBF condition, suggesting integrin clustering^72^ which has been reported more broadly as a cellular response to collagen I.^73^ β1-integrin clustering also has been reported to promote fibronectin deposition, suggesting a potential mechanism by which increased fibronectin expression was observed in the VBF condition. Overall, these observations support the relevance of the integrin-binding mfCMPs to capture aspects of the functions of collagen I with a more native-like conformation for presentation of the integrin-binding site from the fibrils, rather than as a pendant group from amorphous PEG, leading to difference in β1-integrin engagement and cellular responses.

Across all conditions, we observed >95% cell viability after 7 days and found no significant differences in viability among conditions (Figure 5d). We also found no significant differences among conditions for days 1 and 4 but did observe higher metabolic activity in condition E compared to VF and VBF on day 7 (). All conditions also contained cell clusters positive for Ki-67 (a nuclear marker associated with cell cycle and proliferation) on day 7, indicating cell proliferation (Figure 5e). Cells in the viscoelastic hydrogel formulations trended toward a greater percentage of Ki-67 positive (%Ki-67^+ve^) cells, although no statistical differences were observed. Importantly, consistent with this trend, we observed that the VF and VBF conditions both had significantly higher numbers of cells per field (Figure 5f), suggesting that cluster volume arises from increased proliferation in the viscoelastic hydrogel formulations with mfCMP. Further, we did not observe differences in cell volume (e.g., cells taking up more space owing to synthetic matrix viscoelasticity^5^) () or motility (e.g., cells migrating to form larger clusters^37,74^ ) (). Note, we observed strain yielding behavior for mfCMP synthetic ECMs () suggesting viscoplasticity, which may contribute to large spheroid formation based on the literature.^9^ While the VBF condition captures some of aspects of the tumor microenvironment ECM, our aim with this work was to decouple viscoelasticity from integrin binding presentation and probe how those effects change the cellular response, inspired broadly by aspects of the collagen-rich microenvironments in which cancer cells grow with future opportunities for integrating complexity for representing specific tissue microenvironments. Overall, these observations of increased cluster growth, FN-rich matrix deposition, and higher numbers of cells per field support the relevance of the viscoelastic, bioactive mfCMP synthetic ECMs as a platform for 3D cancer cell cultures inspired by collagen-rich microenvironments.

Taken together, these results demonstrate the establishment of a collagen-inspired, fully synthetic ECM with self-assembled structures that impart both tunable bioactivity and viscoelasticity for controlling 3D cellular microenvironments. We have shown how these synthetic ECMs can be photo-cross-linked for imaging using super-resolution microscopy to visualize their fibrillar mfCMP hierarchical structure. Future studies may build upon this framework and methods with the use of other peptide–protein materials to form hierarchically structured, bioinspired soft materials and imaged using STORM. For example, bundlemers, tetrad assemblies of coiled-coil peptides, have been of recent interest in the biomaterials community due to their ability to be used as molecular building blocks for assembly into rigid rods,^75^ lattices,^76,77^ brushes,^78^ and even bundlemer-PEG hydrogels.^79^ Further, other CMP building blocks may prove useful for integration within the polymer–peptide framework, for example, sequences with triple helices further stabilized by using non-natural glycine substitutions^80^ or intra- and interhelical covalent linkages,^81–83^ toward the development of polymer-free, mfCMP-only hydrogel biomaterials. Building upon their tailorable, bioinspired properties, the mfCMP-PEG hydrogels established here can also be used to further probe how matrix viscoelasticity and fibrillar integrin binding affect mechanotransduction, differentiation, or other important cell processes.

## Conclusion

In summary, we constructed a completely synthetic hydrogel system with the structural, biophysical, and bioactive complexity found in many native collagen-rich ECMs. The bioactivity and viscoelasticity of this system are highly tunable and can be used to mimic microenvironments for 3D cell culture studies. The tunable properties of the matrix stem from our use of self-assembling fibrillar mfCMPs designed with integrin binding motifs GFOGER and RGD in their amino acid sequence. To visualize them within equilibrium swollen hydrogels, we have developed hydrogel preparation techniques to allow for STORM imaging, elucidating how hierarchical mfCMP structures appear within a photo-cross-linked polymer–peptide synthetic ECM. Further, we have demonstrated that these ECM mimics are viscoelastic when mfCMPs are introduced, likely due to the physical linkages between mfCMP assembled structures. Notably, increasing mfCMP concentration resulted in faster stress relaxation as the proportion of physical mfCMP cross-links increased. Inspired by collagen-rich tumor microenvironments, T47D breast cancer cells were encapsulated in stress-relaxing, bioactive mfCMP ECMs and exhibited the formation of large, growing cell clusters with limited cluster formation in elastic polymer–peptide materials. Collectively, our studies demonstrate that a completely synthetic collagen-inspired microenvironment can be formed and tuned using bioactive, hierarchically assembled peptide materials as a platform for biomaterials and cell culture applications and provide a framework for controlling viscoelasticity within synthetic matrices.

## Supplementary Material







## Notes

The following files are available free of charge. The Supporting Information is available free of charge at https://pubs.acs.org/doi/10.1021/acscentsci.5c02175.

Experimental methods, supplementary figures (Figures S1–S25) including additional materials characterization, rheology, and images (PDF)
Real-time live cell video (Videos S1–S9) (ZIP)
Transparent Peer Review report available (PDF)

## References

[1] Caliari S. R., Burdick J. A. (2016). A practical guide to hydrogels for cell culture. Nat. Methods.

[2] Lou J., Mooney D. J. (2022). Chemical strategies
to engineer hydrogels for cell
culture. Nature Reviews Chemistry.

[3] Wisdom K. M., Adebowale K., Chang J. (2018). Matrix mechanical plasticity
regulates cancer cell migration through confining microenvironments. Nat. Commun..

[4] Feliciano A. J., Grant R., Fernández-Pérez J., Giselbrecht S., Baker M. B. (2024). Introducing Dynamicity: Engineering
Stress Relaxation Into Hydrogels Via Thiol-Ene Modified Alginate for
Mechanobiological in vitro Modeling of the Cornea. Macromol. Biosci..

[5] Lee H.-P., Stowers R., Chaudhuri O. (2019). Volume expansion and TRPV4 activation
regulate stem cell fate in three-dimensional microenvironments. Nat. Commun..

[6] Lee H.-P., Gu L., Mooney D. J., Levenston M. E., Chaudhuri O. (2017). Mechanical
confinement regulates cartilage matrix formation by chondrocytes. Nat. Mater..

[7] Nam S., Gupta V. K., Lee H.-P. (2019). Cell cycle progression
in confining microenvironments is regulated by a growth-responsive
TRPV4-PI3K/Akt-p27 Kip1 signaling axis. Science
Advances.

[8] Ruiter F. A. A., Morgan F. L. C., Roumans N. (2022). Soft, Dynamic Hydrogel
Confinement Improves Kidney Organoid Lumen Morphology and Reduces
Epithelial-Mesenchymal Transition in Culture,. Advanced Science.

[9] Chaudhuri O., Cooper-White J., Janmey P. A., Mooney D. J., Shenoy V. B. (2020). Effects
of extracellular matrix viscoelasticity on cellular behaviour,. Nature.

[10] Dai X., Wu D., Xu K., Ming P., Cao S., Yu L. (2025). Viscoelastic
Mechanics: From Pathology and Cell Fate to Tissue Regeneration Biomaterial
Development. ACS Appl. Mater. Interfaces.

[11] Zhao X. (2014). Multi-scale
multi-mechanism design of tough hydrogels: building dissipation into
stretchy networks. Soft Matter.

[12] Sinkus R., Siegmann K., Xydeas T., Tanter M., Claussen C., Fink M. (2007). MR elastography of
breast lesions: Understanding the solid/liquid
duality can improve the specificity of contrast-enhanced MR mammography,. Magn. Reson. Med..

[13] Bayat M., Nabavizadeh A., Kumar V. (2018). Automated in vivo sub-Hertz
analysis of viscoelasticity (SAVE) for evaluation of breast lesions. IEEE Transactions on Biomedical Engineering.

[14] Kumar V., Denis M., Gregory A. (2018). Viscoelastic parameters
as discriminators of breast masses: Initial human study results. PLoS One.

[15] Nguyen H., Luong N. H., Peil J. K. (2025). Fast-Relaxing
Hydrogels
Promote Pancreatic Adenocarcinoma Cell Aggressiveness through Integrin
β1 Signaling. Biomacromolecules.

[16] Münster S., Jawerth L. M., Leslie B. A., Weitz J. I., Fabry B., Weitz D. A. (2013). Strain history dependence
of the nonlinear stress response
of fibrin and collagen networks. Proc. Natl.
Acad. Sci. U. S. A..

[17] Guidry C., Grinnell F. (1987). Contraction of Hydrated
Collagen Gels by Fibroblasts:
Evidence for Two Mechanisms by which Collagen Fibrils are Stabilized,. Collagen and Related Research.

[18] Kurniawan N. A., Wong L. H., Rajagopalan R. (2012). Early Stiffening
and Softening of
Collagen: Interplay of Deformation Mechanisms in Biopolymer Networks. Biomacromolecules.

[19] Debenedictis E. P., Keten S. (2019). Mechanical
unfolding of alpha- and beta-helical protein motifs. Soft Matter.

[20] Silver F. H., Freeman J. W., Seehra G. P. (2003). Collagen
self-assembly and the development
of tendon mechanical properties. J. Biomech..

[21] Yang W., Sherman V. R., Gludovatz B. (2015). On the tear resistance
of skin. Nat. Commun..

[22] Balasubramanian, P. ; Prabhakaran, M. P. ; Sireesha, M. ; Ramakrishna, S. Collagen in Human Tissues: Structure, Function, and Biomedical Implications from a Tissue Engineering Perspective. In Advances in Polymer Science; Springer: Berlin, Heidelberg, 2012; Vol. 251, pp 173–206.

[23] Elosegui-Artola A., Trepat X., Roca-Cusachs P. (2018). Control of
Mechanotransduction by
Molecular Clutch Dynamics. Trends in Cell Biology.

[24] Ma Y., Han T., Yang Q. (2021). Viscoelastic Cell Microenvironment: Hydrogel-Based
Strategy for Recapitulating Dynamic ECM Mechanics. Adv. Funct. Mater..

[25] Borelli A. N., Young M. W., Kirkpatrick B. E. (2022). Stress Relaxation and
Composition of Hydrazone-Crosslinked Hybrid Biopolymer-Synthetic Hydrogels
Determine Spreading and Secretory Properties of MSCs,. Adv. Healthcare Mater..

[26] Shoulders M. D., Raines R. T. (2009). Collagen Structure and Stability. Annu. Rev. Biochem..

[27] Adam O., Theobald K., Lavall D. (2011). Increased lysyl oxidase
expression and collagen cross-linking during atrial fibrillation. Journal of Molecular and Cellular Cardiology.

[28] Herchenhan A., Uhlenbrock F., Eliasson P. (2015). Lysyl Oxidase Activity
Is Required for Ordered Collagen Fibrillogenesis by Tendon Cells. J. Biol. Chem..

[29] Akhmanova M., Osidak E., Domogatsky S., Rodin S., Domogatskaya A. (2015). Physical,
Spatial, and Molecular Aspects of Extracellular Matrix of In Vivo
Niches and Artificial Scaffolds Relevant to Stem Cells Research. Stem Cells International.

[30] Tang H., Buehler M. J., Moran B. (2009). A Constitutive Model of Soft Tissue:
From Nanoscale Collagen to Tissue Continuum. Annals of Biomedical Engineering.

[31] Blokland K. E. C., Nizamoglu M., Habibie H. (2022). Substrate
stiffness
engineered to replicate disease conditions influence senescence and
fibrotic responses in primary lung fibroblasts. Frontiers in Pharmacology.

[32] Tlsty T. D., Coussens L. M. (2006). Tumor, Stroma and Regulation of Cancer Development,. Annual Review of Pathology: Mechanisms of Disease.

[33] Knight C. G., Morton L. F., Peachey A. R., Tuckwell D. S., Farndale R. W., Barnes M. J. (2000). The Collagen-binding
A-domains of Integrins α1β1
and α2β1 Recognize the Same Specific Amino Acid Sequence,
GFOGER, in Native (Triple-helical) Collagens. J. Biol. Chem..

[34] Davis G. E. (1992). Affinity
of integrins for damaged extracellular matrix: αvβ3 binds
to denatured collagen type I through RGD sites,. Biochem. Biophys. Res. Commun..

[35] Taubenberger A. V., Woodruff M. A., Bai H., Muller D. J., Hutmacher D. W. (2010). The effect
of unlocking RGD-motifs in collagen I on pre-osteoblast adhesion and
differentiation. Biomaterials.

[36] Barczyk M., Carracedo S., Gullberg D. (2010). Integrins. Cell Tissue Res..

[37] Ford E. M., Hilderbrand A. M., Kloxin A. M. (2024). Harnessing multifunctional collagen
mimetic peptides to create bioinspired stimuli responsive hydrogels
for controlled cell culture. J. Mater. Chem.
B.

[38] Hilderbrand A. M., Ford E. M., Guo C., Sloppy J. D., Kloxin A. M. (2020). Hierarchically
structured hydrogels utilizing multifunctional assembling peptides
for 3D cell culture. Biomaterials Science.

[39] Yao L., Ling B., Zhao S. (2024). Versatile Self-Assembly
of Triblock Peptides into Stable Collagen Mimetic Heterotrimers. International Journal of Molecular Sciences.

[40] Sun X., Liu Z., Zhao S. (2019). A self-assembling collagen mimetic peptide
system to simultaneously characterize the effects of osteogenesis
imperfecta mutations on conformation, assembly and activity. J. Mater. Chem. B.

[41] Ford E. M., Kloxin A. M. (2021). Rapid Production
of Multifunctional Self-Assembling
Peptides for Incorporation and Visualization within Hydrogel Biomaterials. ACS Biomaterials Science & Engineering.

[42] Abramson J., Adler J., Dunger J. (2024). Accurate structure prediction
of biomolecular interactions with AlphaFold 3. Nature.

[43] Obarska-Kosinska A., Rennekamp B., Ünal A., Gräter F. (2021). ColBuilder:
A server to build collagen fibril models. Biophys.
J..

[44] Kotch F. W., Raines R. T. (2006). Self-assembly of
synthetic collagen triple helices. Proc. Natl.
Acad. Sci. U. S. A..

[45] Emsley J., Knight C. G., Farndale R. W., Barnes M. J. (2004). Structure of the
Integrin α2β1-binding Collagen Peptide. J. Mol. Biol..

[46] Brown J. C., Golbik R., Mann K., Timpl R. (1994). Structure and stability
of the triple-helical domains of human collagen XIV. Matrix Biology.

[47] Leikina E., Mertts M. V., Kuznetsova N., Leikin S. (2002). Type I collagen is
thermally unstable at body temperature. Proc.
Natl. Acad. Sci. U. S. A..

[48] Hilderbrand A. M., Taylor P. A., Stanzione F. (2021). Combining simulations
and experiments for the molecular engineering of multifunctional collagen
mimetic peptide-based materials. Soft Matter.

[49] Mukherjee S., Sundarapandian A., Ayyadurai N., Shanmugam G. (2024). Collagen Mimicry
with a Short Collagen Model Peptide. Macromol.
Rapid Commun..

[50] Rodger A., Marshall D. (2021). Beginners guide to
circular dichroism. Biochemist.

[51] Endesfelder, U. ; Heilemann, M. Direct Stochastic Optical Reconstruction Microscopy (dSTORM). In Advanced Fluorescence Microscopy. Methods in Molecular Biology; Verveer, P. , Eds.; Humana Press, New York, NY, 2015; p 1251. 10.1007/978-1-4939-2080-8_14. 25391804

[52] Jensen E., Crossman D. J. (2014). Technical Review: Types of Imaging—Direct STORM,. Anatomical Record.

[53] Schnitzbauer J., Strauss M. T., Schlichthaerle T., Schueder F., Jungmann R. (2017). Super-resolution
microscopy with DNA-PAINT. Nat. Protoc..

[54] Dempsey G. T., Vaughan J. C., Chen K. H., Bates M., Zhuang X. (2011). Evaluation
of fluorophores for optimal performance in localization-based super-resolution
imaging. Nat. Methods.

[55] O’Leary L. E. R., Fallas J. A., Bakota E. L., Kang M. K., Hartgerink J. D. (2011). Multi-hierarchical
self-assembly of a collagen mimetic peptide from triple helix to nanofibre
and hydrogel. Nat. Chem..

[56] Dai N., Wang X. J., Etzkorn F. A. (2008). The Effect of a Trans-Locked Gly-Pro
Alkene Isostere on Collagen Triple Helix Stability. J. Am. Chem. Soc..

[57] Zhang Y., Wang Z., Sun Q., Li Q., Li S., Li X. (2023). Dynamic Hydrogels with Viscoelasticity
and Tunable Stiffness for
the Regulation of Cell Behavior and Fate. Materials.

[58] Tansik G., Stowers R. (2024). Viscoelastic and phototunable
GelMA-alginate hydrogels
for 3D cell culture. MRS Advances.

[59] Charbonier F., Indana D., Chaudhuri O. (2021). Tuning Viscoelasticity
in Alginate
Hydrogels for 3D Cell Culture Studies. Current
Protocols.

[60] Wang Y. J., Zhang X. N., Song Y. (2019). Ultrastiff and Tough
Supramolecular Hydrogels with a Dense and Robust Hydrogen Bond Network. Chem. Mater..

[61] Su H., Zheng R., Jiang L. (2021). Dextran hydrogels via
disulfide-containing Schiff base formation: Synthesis, stimuli-sensitive
degradation and release behaviors. Carbohydr.
Polym..

[62] Rodell C. B., MacArthur J. W., Dorsey S. M. (2015). Shear-Thinning
Supramolecular Hydrogels with Secondary Autonomous Covalent Crosslinking
to Modulate Viscoelastic Properties In Vivo. Adv. Funct. Mater..

[63] Wei Z., Schnellmann R., Pruitt H. C., Gerecht S. (2020). Hydrogel Network Dynamics
Regulate Vascular Morphogenesis. Cell Stem Cell.

[64] Nelson B. R., Kirkpatrick B. E., Miksch C. E. (2024). Photoinduced Dithiolane
Crosslinking for Multiresponsive Dynamic Hydrogels. Adv. Mater..

[65] Babaei B., Davarian A., Pryse K. M., Elson E. L., Genin G. M. (2016). Efficient
and optimized identification of generalized Maxwell viscoelastic relaxation
spectra. J. Mech Behav Biomed Mater..

[66] Stojkov G., Niyazov Z., Picchioni F., Bose R. K. (2021). Relationship between
Structure and Rheology of Hydrogels for Various Applications. Gels.

[67] Acheampong T., Kehm R. D., Terry M. B., Argov E. L., Tehranifar P. (2020). Incidence
Trends of Breast Cancer Molecular Subtypes by Age and Race/Ethnicity
in the US From 2010 to 2016. JAMA Network Open.

[68] Sawicki L. A., Ovadia E. M., Pradhan L. (2019). Tunable synthetic extracellular
matrices to investigate breast cancer response to biophysical and
biochemical cues. APL Bioengineering.

[69] Fernandez-Garcia B., Eiró N., Marín L. (2014). Expression and prognostic
significance of fibronectin and matrix metalloproteases in breast
cancer metastasis. Histopathology.

[70] Holliday D. L., Speirs V. (2011). Choosing the right
cell line for breast cancer research. Breast
Cancer Research.

[71] Locke R. C., Ford E. M., Silbernagel K. G., Kloxin A. M., Killian M. L. (2020). Success
Criteria and Preclinical Testing of Multifunctional Hydrogels for
Tendon Regeneration. Tissue Engineering Part
C: Methods.

[72] Mana G., Valdembri D., Askari J. A. (2023). The βI domain
promotes active β1 integrin clustering into mature adhesion
sites. Life Science Alliance.

[73] Ostrowska-Podhorodecka Z., Ding I., Lee W. (2021). Vimentin tunes cell
migration on collagen by controlling β1 integrin activation
and clustering. Journal of Cell Science.

[74] da
Rocha-Azevedo B., Grinnell F. (2013). Fibroblast morphogenesis on 3D collagen
matrices: The balance between cell clustering and cell migration,. Exp. Cell Res..

[75] Sinha N. J., Wu D., Kloxin C. J., Saven J. G., Jensen G. V., Pochan D. J. (2019). Polyelectrolyte
character of rigid rod peptide bundlemer chains constructed via hierarchical
self-assembly. Soft Matter.

[76] McCahill A. L., Zhang T., Saven J. G., Kloxin C. J., Pochan D. J. (2024). Peptide
Bundlemer Networks or Lattices: Controlling Cross-Linking and Self-Assembly
Using Protein-like Display of Chemistry,. ACS
Nano.

[77] McCahill A., Weisen A. R., Zhang T. (2025). Porous lattice formation
through coiled-coil peptide nanoparticle assembly driven by natural
and non-natural hydrophobic side chains. Polymer.

[78] Langenstein M. G., Crane-Moscowitz K., Brennan J. M., Kloxin C. J., Furst E. M., Pochan D. J. (2025). Sequential
Growth of Quantized Peptide Brushes on Colloidal
Gold. Langmuir.

[79] Meisenhelter J. E., Petrich N. R., Blum J. E. (2024). Impact
of Peptide Length
and Solution Conditions on Tetrameric Coiled Coil Formation. Biomacromolecules.

[80] Kasznel A. J., Zhang Y., Hai Y., Chenoweth D. M. (2017). Structural
Basis for Aza-Glycine Stabilization of Collagen. J. Am. Chem. Soc..

[81] Peterson C. M., Helterbrand M. R., Hartgerink J. D. (2022). Covalent Capture of a Collagen Mimetic
Peptide with an Integrin-Binding Motif. Biomacromolecules.

[82] Li I. C., Hulgan S. A. H., Walker D. R., Farndale R. W., Hartgerink J. D., Jalan A. A. (2019). Covalent Capture
of a Heterotrimeric Collagen Helix. Org. Lett..

[83] Hentzen N. B., Smeenk L. E. J., Witek J., Riniker S., Wennemers H. (2017). Cross-Linked
Collagen Triple Helices by Oxime Ligation. J.
Am. Chem. Soc..

