# Supplementary material for: Synthetic Surrogates of Collagen-Rich Microenvironments: Integrating Modular Bioactive Fibrillar Structure and Tunable Viscoelasticity via Multifunctional Assembling Peptides
Source: ACS Cent Sci. 2026 May 16;12(6):777–88. doi: 10.1021/acscentsci.5c02175 (PMC13306593; doi:10.1021/acscentsci.5c02175)
Supplement: Supplementary file 1 [file oc5c02175_si_004.pdf]

Supporting Information

Synthetic Surrogates of Collagen-Rich Microenvironments: Integrating Modular Bioactive Fibrillar Structure and Tunable Viscoelasticity via Multifunctional Assembling Peptides

*Rafael A. Castro [a], Lina Pradhan [a], Jeffrey Caplan [b,c], Caitlin D'Ambrosio [a], and April M. Kloxin [a,d]**

[a] Department of Chemical and Biomolecular Engineering, University of Delaware, 150 Academy Street, Newark, DE 19716, United States.

[b] Department of Plant and Soil Sciences, University of Delaware, 531 South Collagen Avenue, Newark, DE 19716, United States

[c] Delaware Biotechnology Institute, University of Delaware, 590 Avenue 1743, Newark, DE 19713, United States

[d] Department of Materials Science and Engineering, University of Delaware, 127 The Green, Newark, DE 19716, USA

*Corresponding author Email: akloxin@udel.edu

METHODS

Peptide Synthesis: All peptides were synthesized by Fmoc-based solid phase peptide synthesis (SPPS). Amino acids were dissolved at 0.2 M in DMF and loaded onto a Liberty Blue automated, microwave-assisted peptide synthesizer.

The sequences for mfCMPs are as follows:

- K(azide)G(PKG)$_4$PK(alloc)G(PO**G**)$_6$**FOGER**G(POG)$_6$(DOG)$_4$,
- K(azide)G(PKG)$_4$PK(alloc)G(POG)$_6$G**RGDSP**(POG)$_6$(DOG)$_4$, and
- K(azide)G(PKG)$_4$PK(alloc)G(POG)$_6$(DOG)$_4$.

The sequences for non-mfCMP peptides are as follows:

- K(alloc)GWG**RGDS**,
- K(alloc)G(POG)$_3$PO**GFOGERG**(POG)$_4$G, and
- KK(alloc)GGPQG↓IWGQGK(alloc)K (degradable linker).

The peptides were synthesized starting on a solid polymer resin bead. For mfCMPs, TentaGel Resin was used (Peptides International, Louisville, KY; 0.19 mmol g^{-1}). For all other peptides, Rink Amide MBHA Resin was used (MilliporeSigma, Burlington, MA, 100-200 mesh; 0.65 mmol g^{-1}). For each amino acid, the Fmoc protecting group was removed twice using 20% piperidine (Millipore Sigma, Burlington, MA) in *N,N'*-dimethylformamide (DMF; ThermoFisher, Waltham, MA), followed by 3 coupling steps for the subsequent amino acid at 75 ˚C using oxyma (CEM, Charlotte, NC) and *N,N'*-diisopropylcarbodiimide (DIC; Chem-Impex, Wood Dale, IL). The peptide was cleaved from the solid resin support for 2 hours using a cleavage cocktail (10 mL): 95% v/v trifluoroacetic acid (TFA; ThermoFisher, Waltham, MA), 2.5% v/v water, 2.5% v/v triisoproylsilane (TIPS; ThermoFisher, Waltham, MA), and 2.5% w/v phenol (Sigma-Aldrich, St. Louis, MO). The peptide was then precipitated in 4 ˚C diethyl ether (ThermoFisher, Waltham,

MA) and centrifuged five times (5 minutes, 4 ˚C, 4400 rpm). The peptide was then dried in a fume hood overnight, dissolved in 95/5% water/acetonitrile (HPLC grade), and purified by HPLC (XBridge C18 OBD 5 µm column; Waters Corporation, Milford, MA). Finally, the peptide was lyophilized and stored at -80 ˚C. Ultra-performance liquid chromatography-tandem mass spectrometry (UPLC-MS, Xevo G2-S QTof; Waters, Milford, MA) was used to confirm peptide identity. Note, in addition to the prominent product peaks, some lower molecular weight species were observed that were unable to be separated by HPLC purification; these largely are minor residue deletions that are not observed to affect downstream properties batch-to-batch.

For mfCMP synthesis, the same steps described above were used with some key differences.[1] High-swelling resin was used to better allow for amino acid coupling of these long peptide sequences (up to ~70 amino acids long). For coupling of P or O residues within $(POG)_n$ blocks, any past the first four blocks were coupled using dual-chemistry methods. Briefly, 2-(1H-7-Azabenzotriazol-1-yl)-1,1,3,3-tetramethyluronium hexafluorophosphate (HATU, TCI America, Portland, OR) and diisopropylethylamine (Sigma-Aldrich, St. Louis, MO) were used for 2 couplings, followed by 2 additional DIC and oxyma couplings as described above to increase the coupling efficiency. After HPLC purification (column heater set to 65 ˚C, Jasco CO-2060 Plus; JASCO Corporation, Easton, MD), mfCMPs were further purified by dialysis.[1] First, mfCMPs were dissolved (0.5 mM) and dialyzed (100-500 Da MWCO, Spectrum Laboratories, Boston, MA) against HCl-acidified pH 4 water for 48 hours to remove TFA counterions from the cleavage step. Next, mfCMPs were dialyzed again against neutral pH deionized water for 24 hours to ensure removal of smaller peptide impurities and Cl counterions, then lyophilized and stored at -80 ˚C.

PEG-SH Macromer Synthesis: Following previously published methods,[2] 4-arm poly(ethylene glycol) tetrathiol (PEG-SH) was synthesized starting from 4-arm PEG-OH (JenKem Technology

USA, Plano, TX). The 4-arm PEG-OH (20,000 g mol^{-1}; 10 g, 0.5 mmol) was functionalized with allyl ether groups, which were then reacted with thioacetic acid using radical-mediated thiol-ene ‘click’ chemistry to form PEG-thioacetate. Next, thioacetate groups were deprotected using sodium hydroxide, resulting in PEG-SH. PEG-SH (10 g, 0.5 mmol) was dissolved in water (300 mL) and treated with tris(2carbonxyethyl)phosphine (TCEP, 3.5 g, 12.2 mmol) stirred overnight to break disulfide bonds. Finally, PEG-SH was dialyzed (1 kDa MWCO, Spectrum Laboratories, Boston, MA) against pH 4 deionized water to remove TCEP. PEG-SH functionalization was confirmed by ^{1}H NMR in DMSO-d6 before lyophilization and storage at -80 ˚C. ^{1}H NMR (400 MHz, [D_6] DMSO): δ = 2.3 (m, 1 H; SH). Stock solutions were prepared at 50 mM thiol groups, and concentration was confirmed using Ellman’s assay.

LAP Photoinitiator Synthesis: Following previously published methods,[2-3] LAP was synthesized. Briefly, 2,4,6-trimethylbenzoyl chloride (3.2 g, 18 mmol) was added dropwise to dimethyl phenylphosphinite (3.0 g, 18 mmol) and reacted overnight under argon at room temperature. Four fold excess of lithium bromide (6.1 g, 72 mmol) in 2-butanone (100 mL) was then added, heated to 50 °C, and stirred for 10 minutes. The resulting white precipitate was filtered and rinsed three times with 2-butanone and dried under vacuum before storage at -80 ˚C.

mfCMP Sample preparation and Assembly: mfCMP powder was dissolved at the desired concentration (0.3 – 5 mM) in deionized water or 1X Dulbecco’s phosphate buffered saline (DPBS; ThermoFisher, Waltham, MA). The peptide solution was heated to 85 ˚C for 15 minutes to dissociate any aggregates and triple helices, then allowed to cool to room temperature (~1 ˚C min^{-1}) and assemble for 48 hours before characterization or use for making hydrogels.

Dynamic Light Scattering: Aggregation of mfCMPs in 95/5% water/acetonitrile (HPLC grade), mimicking the conditions used for peptide purification, was examined using dynamic light

scattering. Lyophilized mfCMPs were dissolved (10 mg/mL), filtered with a 0.45 µm nylon filter, loaded in a polystyrene cuvette (2 mL), and measured using a Zetasizer Ultra Red instrument (Malvern Panalytical; Malvern, Worcestershire, UK). For each measurement, 30 scans were collected after a 120 s equilibrium wait time. Three independent measurements were collected per mfCMP peptide, then averaged.

Circular Dichroism: Triple helix formation and melting temperatures in DPBS were characterized using a circular dichroism (CD) spectropolarimeter (J-810 CD Spectropolarimeter; JASCO corporation, Easton, MD). Samples were prepared in triplicate by diluting to 0.3 mM after mfCMP assembly described above. Measurements were conducted in a quartz cuvette with a 1 mm path length, ranging from 250 nm to 195 nm (scan rate of 50 nm min^{-1}) at 4 ˚C, 25 ˚C, 37 ˚C, 40 ˚C, and 60 ˚C, averaged over 3 scans per replicate. Data points were recorded at each nanometer, and the resulting CD data were transformed to mean residue ellipticity [θ] (deg cm^2 dmol^{-1}) by Equation 1:

$$[\theta] = \theta / (L*c*N) \quad (1)$$

where θ is the measured ellipticity (millidegrees), L is the pathlength (mm), c is the peptide concentration (mM), and N is the number of amino acid residues in the peptide. To characterize melting events (T_{mx}, where x represents the index of the melting event, with the first event representing the temperature at which approximately 50% of the triple helices are assembled and 50% have thermally dissociated), a similar protocol was implemented. Here, the wavelength of interest was 225 nm (where a characteristic peak can be found that is associated with a polyproline type II helix) over a temperature range of 4 ˚C to 80 ˚C. Data points were collected at every degree over the temperature range with a heating rate of 10 ˚C hr^{-1}. The resulting data was transformed using Equation 1 as described above. Origin software (OriginLab Corporation, Northampton, MA)

was used to calculate the first- and second-order derivatives of each melting curve with the Savitzky-Golay smoothing algorithm. For each peptide, the two largest changes in magnitude from positive to negative or negative to positive in the second-order derivative curves were reported as a melting event. Note, the absorbance for NaCl and KCl found in PBS, the mfCMP solvent used here, can interfere with CD data near 195-200 nm.

Transmission Electron Microscopy: Carbon-coated copper grids (200 mesh; Electron Microscopy Sciences, Hatfield, PA) were treated with a glow discharge plasma cleaner (PELCO easiGlow, Ted Pella Inc., Redding, CA) for 1 minute to improve hydrophilicity and peptide adhesion. Assembled mfCMP samples (4 µL, assembled at 1 mM and diluted to 0.3 mM immediately before imaging) were drop-cast onto the grid and allowed to sit for 1 minute, then blotted using filter paper. mfCMP-cast grids were air-dried for 10 minutes, then negatively stained with uranyl acetate. A solution of 2% uranyl acetate (4 µL, Electron Microscopy Sciences, Hatfield, PA) in deionized water was drop-cast onto the grid for 20 seconds, then blotted with filter paper. Stained mfCMP-cast grids were allowed to air-dry for at least 1 hour, then imaged by transmission electron microscopy (TEM; TALOS L120C, FEI Company, Hillsboro, OR).

Hydrogel Formation: Components for hydrogel formation included 4-arm PEG-SH (20 kDa), nonassembling matrix metalloproteinase (MMP)-degradable linker peptide (K(alloc)GGPQG↓IWGQGK(alloc)K), pendent integrin-binding peptides (K(alloc)GWGRGDS, K(alloc)G$(POG)_3$POGFOGERG$(POG)_4$G), mfCMPs, and lithium phenyl-2,4,6-trimethylbenzoyl-phosphinate (LAP) photoinitiator. PEG-SH macromer (~50 mM thiols), linker peptide (~40 mM alloc), pendent peptides (~40 mM), and photoinitoator (~25 mM) stock solutions were prepared in DPBS, and concentrations were confirmed with Ellman's assay or UV-Vis spectroscopy as previously published.[2, 4] Stock solutions were stored frozen, then thawed before use. For

mfCMPs, stock solutions were prepared and assembled as described above, frozen, and lyophilized.

Hydrogel precursor solutions were formed in DPBS at 10 wt% PEG-SH (20 mM thiols), 2.2 mM LAP, and varying concentrations of linker peptide, pendent peptides, and mfCMP: 20 mM combined alloc functional handles, keeping 1:1 SH:alloc for rheology measurements; and constant cell-degradable linker peptide concentration (13 mM alloc functional handles) to keep hydrogel stiffness the same between conditions for cell culture experiments. All other components were mixed thoroughly before being added to lyophilized mfCMP aliquots. Once added, the precursor solution containing mfCMP was gently pipette mixed and microcentrifuged. This process was repeated for a total of 3 times, after which the precursor solution was ready for cell encapsulation or direct polymerization by irradiation (10 mW cm^{-2} at 365 nm for 4 minutes; Exfo Omnicure Series 2000 light source with 365 nm bandpass filter; Excelitas Technologies Corp., Waltham, MA). For in situ measurements, 10 μL of precursor solution was used to form a hydrogel on a rheometer as described below. For equilibrium-swollen hydrogels, 20 μL of precursor solution was pipetted onto a syringe mold as described below. For imaging mfCMPs in hydrogels, 5 μL of precursor solution was pipetted directly onto a cleaned microscope cover glass (22x22 mm, No. 1.5; Fisher Scientific, Pittsburgh, PA). Hydrogels formed on cover glasses were transferred to non-treated tissue culture plastic 6-well plates and let equilibrium-swell in DPBS for at least 8 hours.

mfCMP Labelling in Hydrogels: mfCMPs were labelled by copper-catalyzed azide-alkyne cycloaddition (CuAAC), where an alkyne-functionalized fluorophore was attached to the azide-functionalized peptide. DPBS was removed from equilibrium-swollen hydrogels formed on cover glasses before labeling. Fresh DPBS was added, then CuAAC reagents were dissolved in water and added in-order for the following final concentrations: copper sulfate ($CuSO_4$, 1 mM), BTTAA

(BTTAA in water, 140 μM), sodium l-ascorbate (1.2 mM), and the desired fluorophore. For imaging by confocal microscopy, AlexaFluor 488 (20 mM in water, ThermoFisher, Waltham, MA, Cat. No. A10267) was used. For super-resolution STORM imaging, AlexaFluor 647 (0.2 mM in water, ThermoFisher, Waltham, MA, Cat. No. A10278) was used for its high number of cycles and photon output per cycle and its high resistance to photobleaching. The reaction was allowed to proceed at room temperature in low-light conditions for 1 hour with gentle shaking. The reaction solution was then removed, and hydrogels were washed twice with DPBS for 30 minutes with gentle shaking. A final wash in DPBS was conducted overnight at 4 ˚C after which DPBS was replaced with fresh stock, and hydrogels were stored in the dark until they were imaged.

STORM and Confocal Imaging of mfCMPs in Hydrogels: Hydrogels formed on cover glasses were mounted on stainless steel gaskets (10 mm inner diameter). STORM imaging and buffer preparation methods were based on previously published methods.[5] Frozen aliquots of solutions A (30 mM Tris/I, pH 8.5, 1 mM ethylenediaminetetraacetic acid, 6.25 μM glucose oxidase, 2.5 μM catalase), B (250 mM cysteamine-HCl in water), and C (250 mM glucose in water) were thawed to room temperature. To create STORM oxygen scavenging buffer, the solutions were combined in the following order: 800 μL of solution A was gently pipette mixed into 100 μL of solution B; the resulting solution A+B was then gently pipette mixed into 100 μL of solution C and loaded into a Hamilton GASLIGHT syringe (PEEK capillary tubing tip). Bubbles were removed, and the combined solution was loaded to fill the metal gasket containing a hydrogel. A coverglass was then used to seal the gasket, and air bubbles were removed to create an oxygen-free chamber for imaging. Hydrogels were imaged using the Andor Dragonfly Spinning Disk Confocal microscope (63 x 1.47 NA Leica TIRF objective, Andor Zyla 4.2 camera). A 1024 x 1024 pixel imaging area was excited with a 638 nm laser at 100% power with a power density

filter of 3 and 50 ms exposure time. A bandpass emission filter of 455 nm was in place to prevent damage to the camera. Once the fluorophores in the field of view were sufficiently driven into a dark state (~1 minute), 10000 frames were captured. Data was then localized and rendered using Picasso[6] software. The data was filtered for an uncertainty value < 10, and a cross correlation drift correction was applied.

mfCMP fibril size analysis: Length and width of mfCMP fibrils were characterized by processing STORM images in Fiji ImageJ software. First, scale was set for each image using the scale bar. Next, images were converted to 8-bit, background was removed using the despeckle function, then the lower bound of the image threshold was increased by 1-2 points until individual fibrils were visible. Fibril length and width were then determined using the Ridge Detection plugin with the following parameters: line width = 20, high contrast = 1000, low contrast = 200, extend line on, estimate width on, and slope method for overlap resolution. At least 70 fibrils were measured for each mfCMP.

Rheology on in situ Hydrogels: Hydrogels with different concentrations of mfCMP (0 mM, 5 mM, 9 mM, 13 mM, 20 mM) were formed on a rheometer (HR 30 Discovery Hybrid Rheometer; TA instruments, New Castle, DE). A UV-visible light accessory with a quartz plate was attached to a filtered mercury lamp using a liquid-filled light guide (Exfo Omnicure Series 2000 light source with 365 nm bandpass filter; Excelitas Technologies Corp., Waltham, MA) for in situ polymerization. Hydrogel precursor solution (10 µL) was pipetted onto the center of the bottom quartz plate, and the geometry (8 mm sandblasted parallel plate, TA Instruments, New Castle, DE) was lowered to fill the gap. Measurements were collected for 1 minute before activating the light source. Hydrogels were then formed by irradiating for 4 minutes (10 mW cm^{-2} at 365 nm) while the storage (*G'*) and loss (*G''*) moduli were measured at 2% strain and a frequency of 2 rad s^{-1} as

a time sweep. Subsequent frequency (2% strain, 0.1 rad s^{-1} to 500 rad s^{-1}) and strain (2 rad s^{-1}, 1% to 5000% strain) sweeps were also performed. For stress relaxation measurements, 10^5 s of measurements followed a 15% strain (determined to be in the LVR by the strain sweeps). Before collecting stress relaxation measurements, the strain was first ramped from 0 to 15% over a period of 2 seconds (2 second rise time), immediately after which measurements were collected for up to 10^5 seconds. A ring of light mineral oil was applied around the hydrogel to minimize drying effects over the duration of the experiment.

Rheology on Equilibrium Swollen Hydrogels: Hydrogel precursor solutions containing mfCMP were prepared and then pipetted into a cylindrical syringe mold (1 mL syringe with the tip removed). The syringe molds were then irradiated for 4 minutes upright with a collimated light source (10 mW cm^{-2} at 365 nm). After the hydrogels were polymerized, they were transferred to a non-treated tissue culture plastic 48-well plate for equilibrium swelling (>8 hours) in DPBS (500 µL). Before rheology measurements, the diameter of each gel was recorded and inputted into the software. Time sweeps were performed as described above to find G' and G'', replacing the quartz plate with a Peltier plate.

Fitting Stress Relaxation Data to the Generalized Maxwell Model: Stress relaxation curves from Figures 4 and S15 were fitted to the generalized Maxwell model described by Equation 2, producing Figure S16 and Table S1:

$$\sigma(t) = \varepsilon_0 \sum_{i=1}^{n} G_i e^{-t/\tau_i} \quad (2)$$

where $\sigma(t)$ is the sheer stress as a function of t time (s) normalized to maximum stress, ε_0 is the applied step shear strain, G_i is the modulus of mode i normalized to maximum stress, and τ_i is the relaxation time (s) of mode i. The following variables were defined: let A_i be equal to $\varepsilon_0 G_i$, the stress of mode i normalized to maximum stress, and let i = 3 modes. Fitting was done using the

Microsoft Excel solver function. The objective was set to the sum of the squares of the measured normalized stresses for each timepoint, variables being changed were set to $A_{1\text{-}3}$ and $\tau_{1\text{-}3}$ with the constraints of positive values, and solving method was set to GRG Nonlinear. Initial guesses for $A_{1\text{-}3}$ and $\tau_{1\text{-}3}$ were set to 0.4, 0.35, 0.25, 200 s, 2000 s, and 20000 s, respectively.

Culture and Transduction of T47D Breast Cancer Cells: Following a previously published protocol,[7] T47D breast cancer cells (passages 16-24, ATCC) were cultured on tissue culture polystyrene in Dulbecco's Modified Eagle's Medium (DMEM)/Hams F-12 50/50 mix with L-glutamine, 15 mM Hepes (DMEM-F12, Corning Cellgro) supplemented with 10% v/v fetal bovine serum (Invitrogen), 1% penicillin-streptomycin (PS), and 0.2% v/v fungizone. The culture media were replaced with freshly prepared media every 48 to 72 hours, and cells were trypsinized (trypsin/ethylenediaminetetraacetic acid, Thermo Fisher Scientific) at ~80% to 85% confluency. For GFP expression, cells were stably transduced using a commercially available lentiviral system as previously reported.[7]

Encapsulation of T47D-GFPs in Hydrogels: T47D-GFPs were encapsulated based on previously published protocols.[7-8] Cells were collected, centrifuged, and suspended in hydrogel precursor solution at a density of 5000 cells μL^{-1}. First, 15 μL of cell-free hydrogel precursor solution was polymerized in a sterile syringe mold (90 seconds, 365 nm, 10 mW cm^{-2}). Next, 5 μL of cells suspended in hydrogel precursor solution was pipetted, then polymerized on top of the 15 μL base layer (90 seconds, 365 nm, 10 mW cm^{-2}). Directly after polymerization, dual-layer hydrogels were moved to sterile non-treated tissue culture plastic 48-well plates and incubated with F12 media. After 30 minutes and 24 hours, the media were replaced to remove unreacted hydrogel components. Culture medium was then replaced every 48-72 hours. This dual-layer hydrogel formation method was used to allow for the cell-laden layer to be in contact with a substrate of the

same stiffness, compared to the much stiffer tissue culture plastic below. The dual-layer approach also reduces cell migration out of the hydrogel, provides easier handling of the hydrogel, and saves valuable peptide material.

Viability of T47D-GFPs in Hydrogels: Following a previously published protocol,[7] cell viability in hydrogels was assessed after 7 days of culture using a LIVE/DEAD kit (Thermo Fisher Scientific). Media were removed from the cell-containing hydrogels ($n = 3$), followed by 2 washes with 400 μL of sterile DPBS for 5 min and then incubated with Hoechst nuclear stain (40 μL mL^{-1} in DPBS) and ethidium homodimer (4 μM in DPBS). Before imaging, hydrogels were washed twice with 400 μL of DPBS for 5 min, then transferred to an eight-well glass-bottom chamber slide (Nunc Lab-Tek II Chambered Coverglass, Thermo Fisher Scientific, Waltham, MA). Fresh DPBS was used to fill each chamber, then hydrogels were imaged using a confocal microscope (Zeiss LSM 800, 10x objective and image frame size of 1024x1024, ~100 μm z-stack, three images per hydrogel). Orthogonal projections were processed for each z-stack, and live (blue) and dead (red) cells were counted using Volocity software. The percentage of viable cells was calculated using the number of live cells divided by the total number (live + dead) of cells multiplied by 100.

Immunostaining and Imaging of T47D-GFPs in Hydrogels: Following a previously published protocol,[7] encapsulated T47D breast cancer cells were fixed in 4% methanol-free paraformaldehyde (PFA; Thermo Fisher Scientific, Waltham, MA) for 30 minutes after 7 days of culture. The hydrogels were then washed twice in DPBS, then permeabilized (3% w/v BSA and 0.5% v/v Triton X-100 in DPBS) and blocked (5% w/v BSA and 0.1% v/v Triton X-100 in DPBS). After blocking, hydrogels were incubated with primary antibody (1:300 dilution of rabbit anti-human Ki-67; Abcam, Walthan, MA) in blocking solution overnight at 4 ˚C. The following day, hydrogels were washed three times with permeabilization solution for 60 minutes, then incubated

overnight with the secondary antibody Alexa Fluor 647 goat anti-rabbit (1:200 dilution; Thermo Fisher Scientific, Waltham, MA) and phalloidin-TRITC to stain F-actin (40 µg mL^{-1}; Sigma-Aldrich, St. Louis, MO) in blocking solution at 4 ˚C. The samples were protected from light using aluminum foil. The hydrogels were then washed (45 min) three times with DPBS and incubated with Hoechst 33342 (2 µg mL^{-1} in DPBS) at room temperature for 30 minutes to stain DNA in cell nuclei. The hydrogels were then washed again (30 min) three times with DPBS before storage at 4 ˚C in DPBS until imaging. Samples were imaged (n = 3 hydrogels, 3 images per hydrogel, >50 objects counted) on a confocal microscope (Zeiss LSM 800). Quantitative analysis of Ki-67^{+ve} cells and Hoechst-stained nucleus numbers were performed using Fiji ImageJ software. Similarly, additional samples were stained with β1 antibody AIIB2 (rat, Developmental Studies HybridomaBank, University of Iowa; deposited by C. H. Damsky) at a 1:300 dilution, then using a secondary antibody AlexaFluor 647 goat anti-mouse (1:200 dilution; Thermo Fisher Scientific, Waltham, MA).

Cell Cluster Volume Analysis: Following a previously published protocol,[8] cell cluster volume of stained cells (phalloidin-TRITC, staining F-actin) was quantitatively analyzed with Volocity 3D image analysis software. Z-stack images were imported into Volocity, and cell clusters were identified by searching for objects in the red channel (TRITC-labeled F-actin). An object was defined as a single cell or a cluster of cells directly touching. Filters were used to improve the accuracy of object volume measurements (filter to close the objects, 4 iterations; filter to fill holes in the objects). Noise was removed from the objects using a fine filter to precisely resolve the object surface. Objects with volumes below 3000 μm^3 were not measured and considered to be debris or non-specific staining. The volume of each object in an image was calculated, and the

mean volume was recorded. For each hydrogel condition, mean volumes were averaged and reported (n = 3 hydrogels, 3 images per hydrogel, >50 objects counted).

Staining of T47D Secreted Proteins in Hydrogels: Secreted collagen I and fibronectin were stained from T47D breast cancer cells encapsulated in hydrogels. All antibodies were diluted in 1.5% w/v BSA in DPBS. All washes were done using 1.5% w/v BSA and 0.2% v/v tween-20 in DPBS at room temperature with gentle rocking unless otherwise noted. Cells were encapsulated, cultured for 7 days, then fixed as previously described. Hydrogels were washed (5 min) twice in DPBS, then blocked overnight (5% w/v BSA in DPBS) and washed (5 min) four times. Hydrogels were permeabilized (0.2% v/v Triton X-100 in DPBS) and washed (5 min) three times. Next, hydrogels were incubated with primary antibody (1:200 dilution of mouse anti-fibronectin; Abcam, Walthan, MA) overnight at 4 ˚C. Hydrogels were washed (20 min) three times, then incubated with secondary antibody Alexa Fluor 568 goat anti-mouse (1:300 dilution, Thermo Fisher Scientific) overnight at 4 ˚C. Hydrogels were washed (20 min) three times, then incubated with primary antibody (1:200 dilution of rabbit anti-collagen I; Abcam, Walthan, MA) overnight at 4 ˚C. Hydrogels were then washed (20 min) three times, then incubated with secondary antibody Alexa Fluor 405 goat anti-rabbit (1:300 dilution, Thermo Fisher Scientific) and ActinGreen (1:500 dilution, Thermo Fisher Scientific) overnight at 4 ˚C. Hydrogels were then washed once (30 min) then three times (10 min).

Live Imaging of T47Ds in Hydrogels: Live imaging experiments were performed 8 hours after T47D encapsulation to allow for hydrogels to equilibrium-swell. A Zeiss LSM 800 confocal microscope equipped with an incubation chamber (37 ˚C, 5% CO_2, with humidity control) was used to capture brightfield and green channels every 30 minutes over 14 hours (29 timepoints). These data are included as .avi video files as Supporting Information.

Statistical Analysis: All values are the mean ± standard error for each condition for three independent sample measurements unless otherwise stated. Statistical significance was determined by one-way ANOVA with Tukey's multiple comparisons test. Statistical significance is shown (*p < 0.05; **p < 0.01; ***p < 0.001; ****p < 0.0001).

SUPPLEMENTAL FIGURES

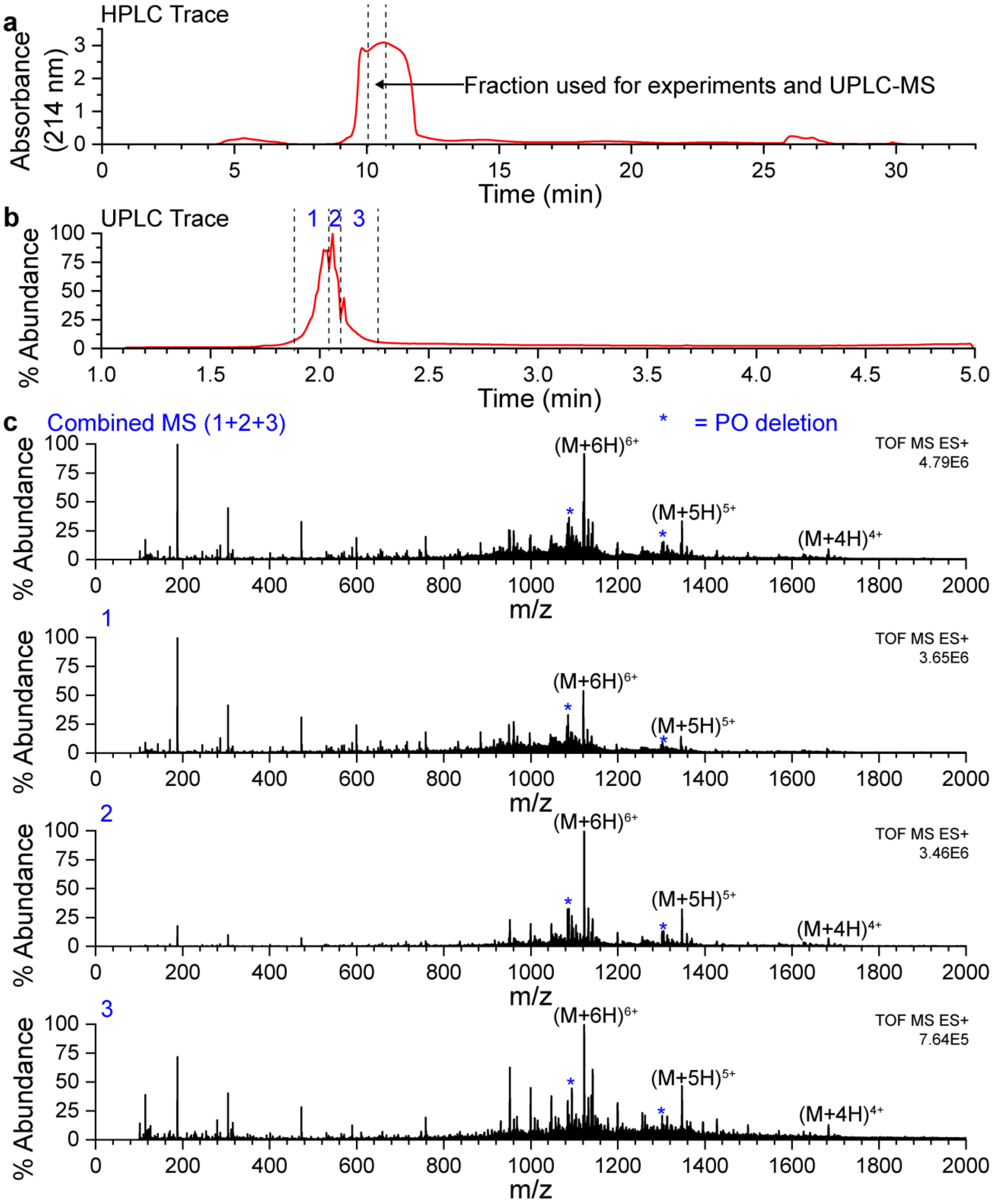

Figure S1. Purification and characterization of mfCMPa-G-az. a) Absorbance channel (214 nm) during HPLC purification showing collection time (10.0 to 10.8 min) of the fraction used for all experiments in this work. The peptide eluted from 23.9 to 24.9% acetonitrile (9.5 to 12.0 min) over a gradient of 0.7% acetonitrile per minute (18% to 30%). b) UPLC-MS chromatograms for purified peptide showing c) MS integration for the UPLC trace followed by separate MS integration for peaks within UPLC trace. Note, in addition to the prominent product peaks, some lower molecular weight species were observed that were unable to be separated by HPLC purification; these largely are minor residue deletions that are not observed to affect downstream properties batch-to-batch.

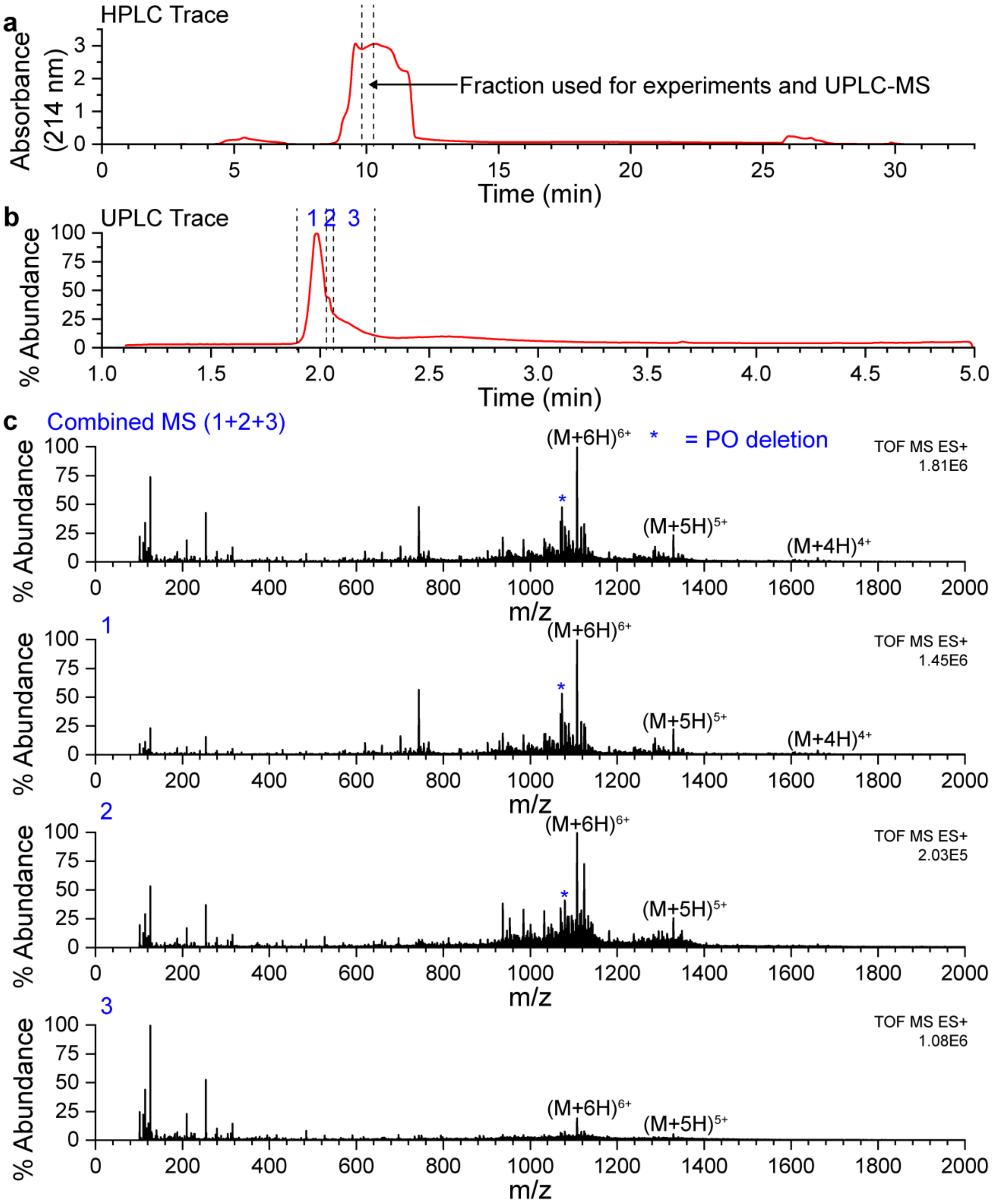

Figure S2. Purification and characterization of mfCMPa-R-az. a) Absorbance channel (214 nm) during HPLC purification showing collection time (9.9 to 10.2 min) of the fraction used for all experiments in this work. The peptide eluted from 23.5 to 24.9% acetonitrile (9.0 to 12 min) over

a gradient of 0.7% acetonitrile per minute (18% to 30%). b) UPLC-MS chromatograms for purified peptide showing c) MS integration for the UPLC trace followed by separate MS integration for peaks within UPLC trace. Note, in addition to the prominent product peaks, some lower molecular weight species were observed that were unable to be separated by HPLC purification; these largely are minor residue deletions that are not observed to affect downstream properties batch-to-batch.

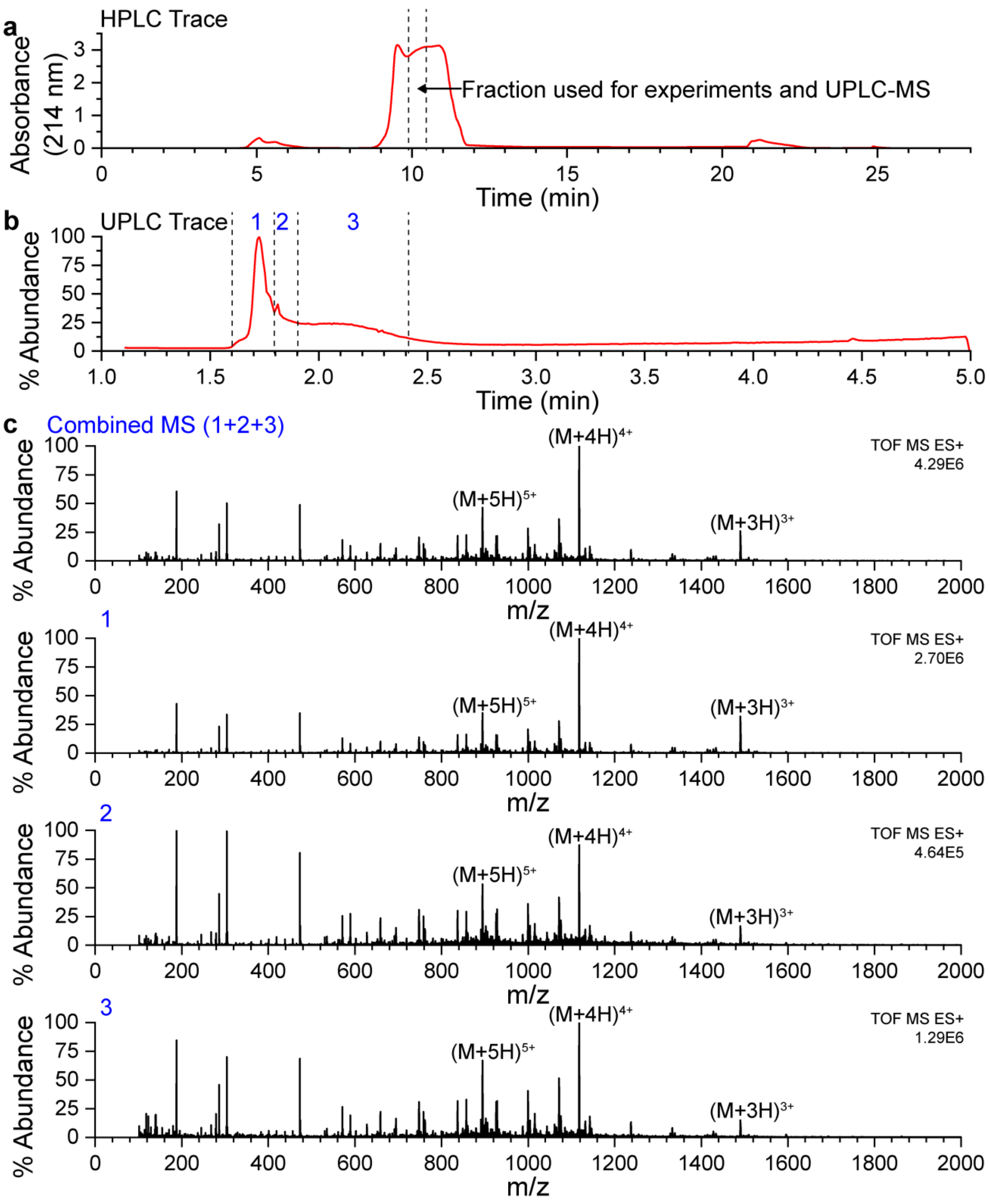

Figure S3. Purification and characterization of mfCMPa-az. a) Absorbance channel (214 nm) during HPLC purification showing collection time (9.9 to 10.3 min) of the fraction used for all experiments in this work. The peptide eluted from 24.2 to 26.4% acetonitrile (9.0 to 11.7 min) over

a gradient of 0.83% acetonitrile per minute (20% to 30%). b) UPLC-MS chromatograms for purified peptide showing c) MS integration for the UPLC trace followed by separate MS integration for peaks within UPLC trace. Note, in addition to the prominent product peaks, some lower molecular weight species were observed that were unable to be separated by HPLC purification; these largely are minor residue deletions that are not observed to affect downstream properties batch-to-batch.

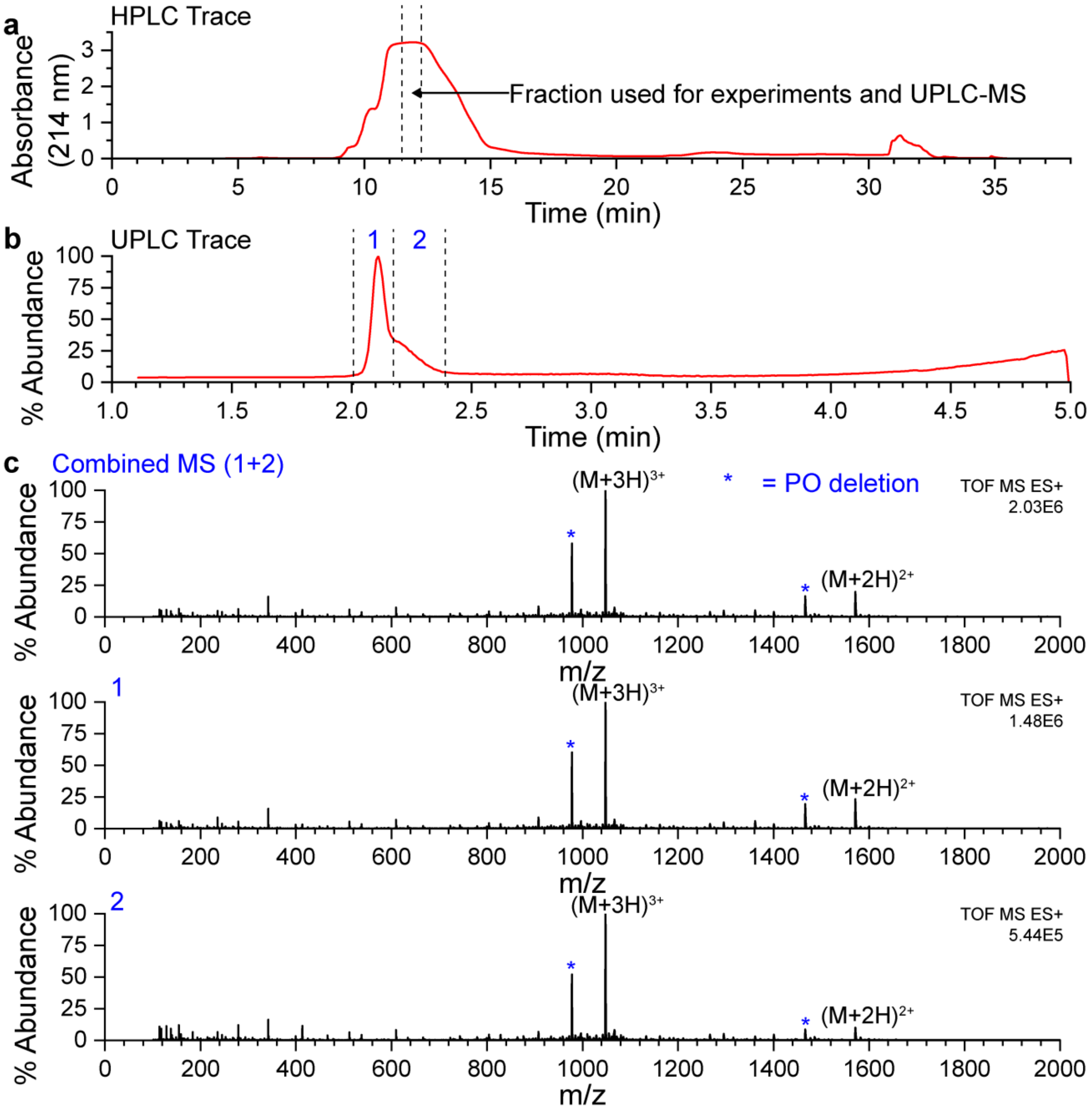

Figure S4. Purification and characterization of pendent GFOGER peptide. a) Absorbance channel (214 nm) during HPLC purification showing collection time (11.3 to 12.2 min) of the fraction used for all experiments in this work. The peptide eluted from 20.4 to 23.3% acetonitrile (10-14 min) over a gradient of 0.73% acetonitrile per minute (16% to 32%). b) UPLC-MS chromatograms for purified peptide showing c) MS integration for the UPLC trace followed by separate MS integration for peaks within UPLC trace. Note, in addition to the prominent product peaks, some lower molecular weight species were observed that were unable to be separated by HPLC

purification; these largely are minor residue deletions that are not observed to affect downstream properties batch-to-batch.

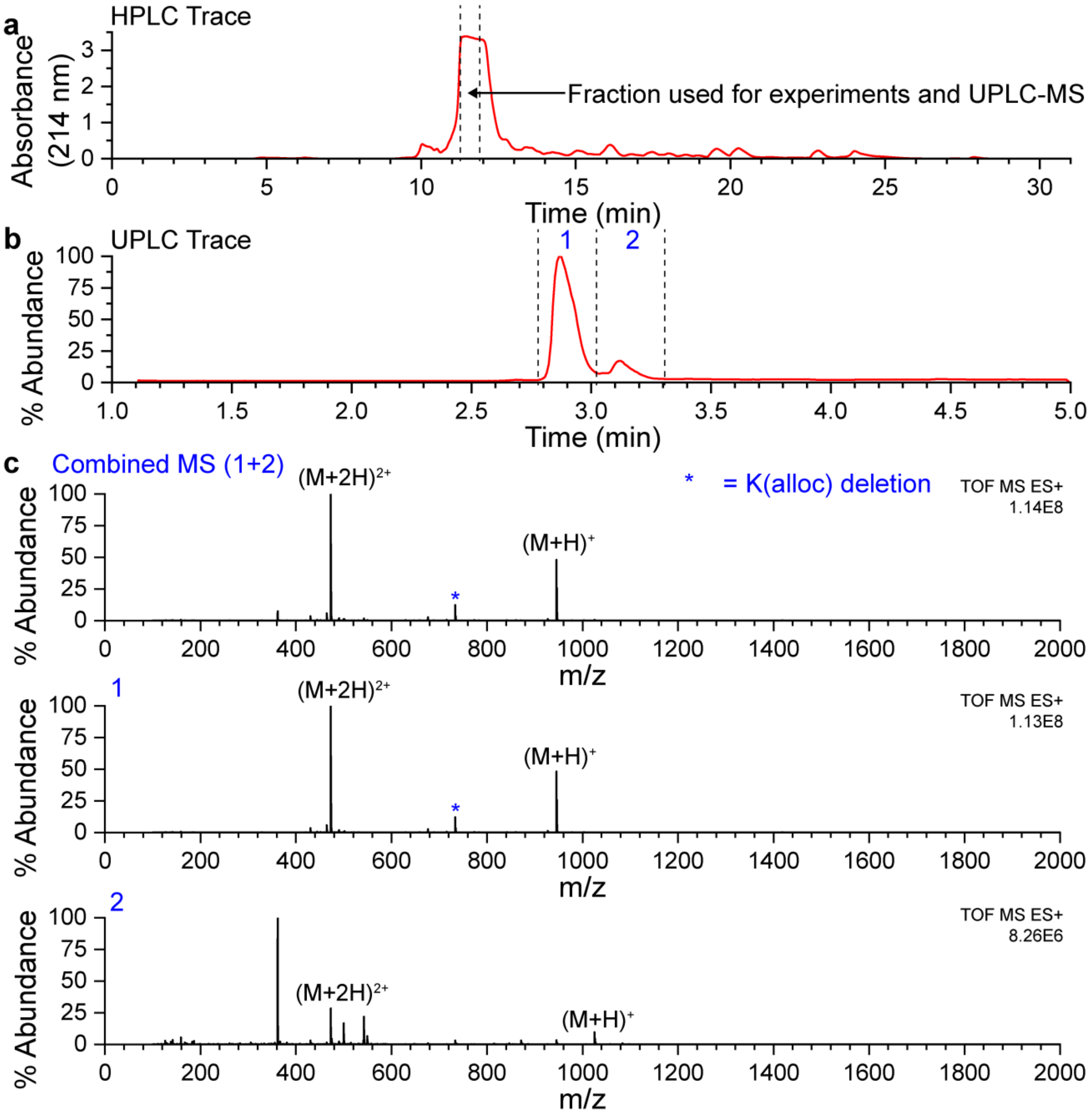

Figure S5. Purification and characterization of pendent RGD peptide. a) Absorbance channel (214 nm) during HPLC purification showing collection time (11.2 to 11.8 min) of the fraction used for all experiments in this work. The peptide eluted from 26.5 to 28.5% acetonitrile (10.5 to 12.5 min) over a gradient of 1% acetonitrile per minute (20%-35). b) UPLC-MS chromatograms for purified peptide showing c) MS integration for the UPLC trace followed by separate MS integration for peaks within UPLC trace. Note, in addition to the prominent product peaks, some lower molecular weight species were observed; these largely are minor residue deletions that are not observed to affect downstream properties batch-to-batch.

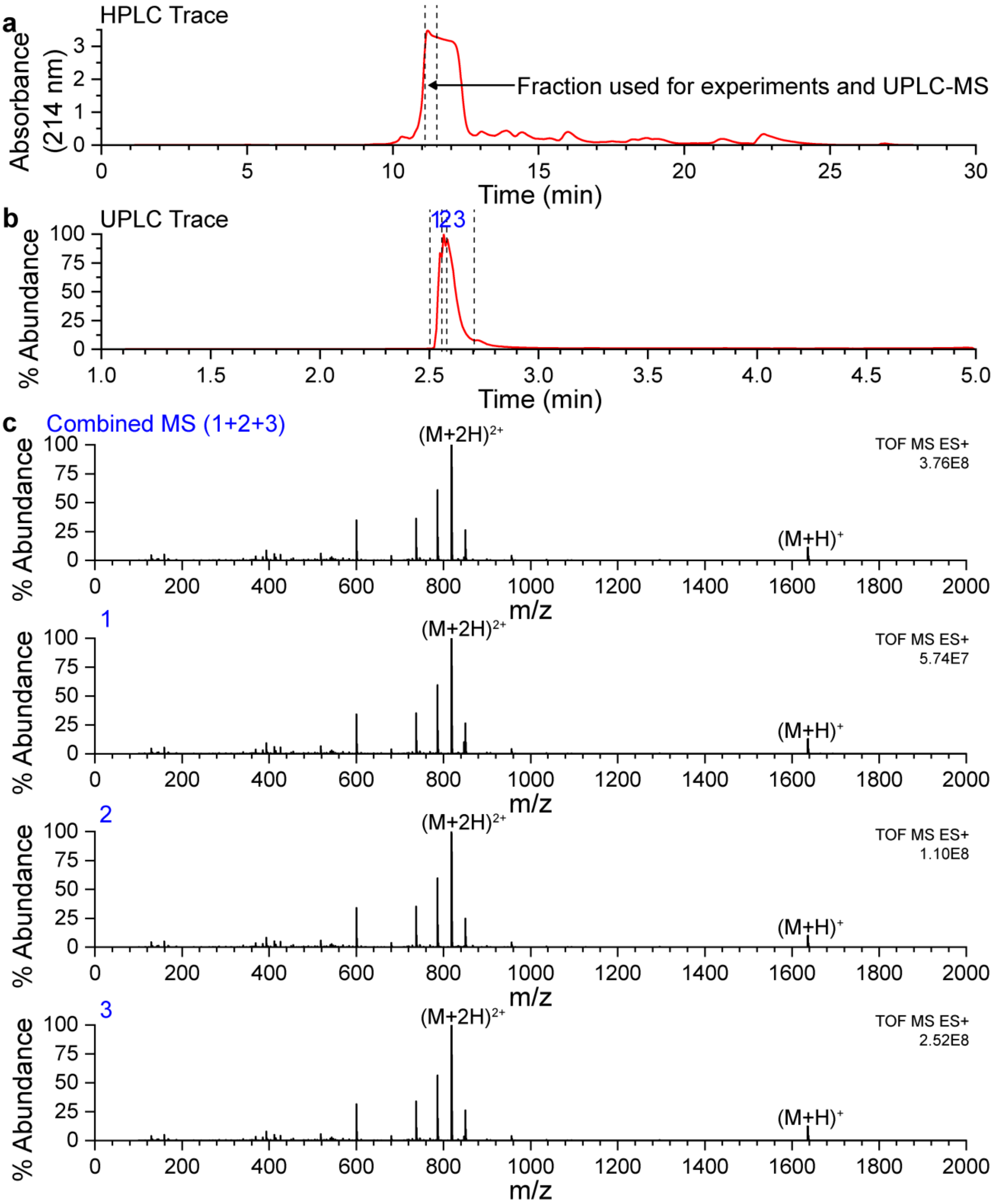

Figure S6. Purification and characterization of degradable linker peptide. a) Absorbance channel (214 nm) during HPLC purification showing collection time (11.1 to 11.5 min) of the fraction used for all experiments in this work. The peptide eluted from 29.0 to 30.1% acetonitrile (10.8 to 12.5

min) over a gradient of 0.6% acetonitrile per minute (25% to 33%). b) UPLC-MS chromatograms for purified peptide showing c) MS integration for the UPLC trace followed by separate MS integration for peaks within UPLC trace. Note, in addition to the prominent product peaks, some lower molecular weight species were observed; these largely are minor residue deletions that are not observed to affect downstream properties batch-to-batch.

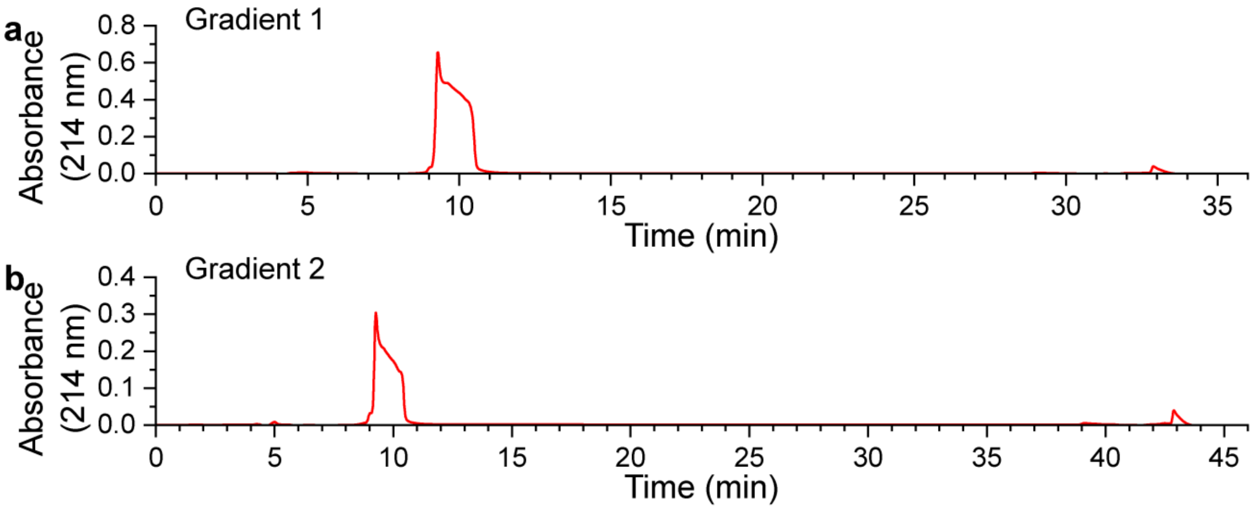

Figure S7. HPLC traces of mfCMPa-R-az at slower gradients. a) Gradient 1: 22 to 23.7% acetonitrile over 20 minutes (4 to 24 min; 0.085% acetonitrile per min). b) Gradient 2: 23.4 to 23.7% acetonitrile over 30 minutes (4 to 34 min; 0.01% acetonitrile per min). Using slower gradients did not change peptide elution time (9 min to 11 min) or separation.

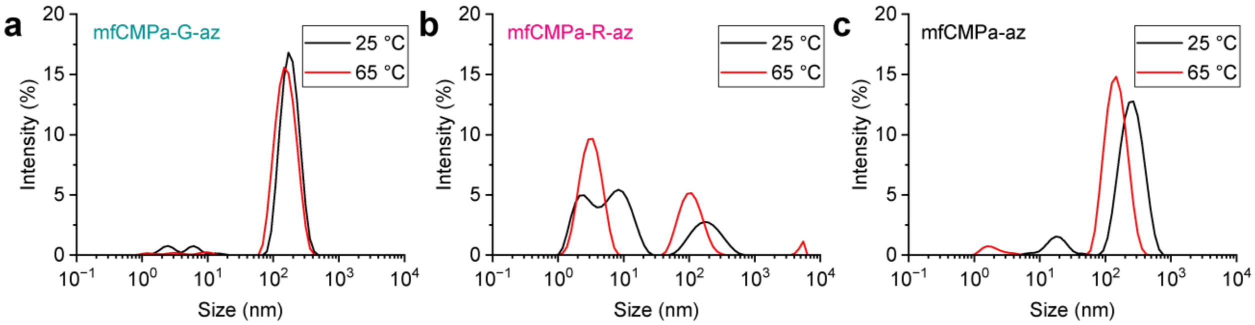

Figure S8. DLS measurements of mfCMPs in 95/5% water/acetonitrile at 25 ˚C and 65 ˚C. a) mfCMPa-G-az b) mfCMPa-R-az, and c) mfCMPa-az. All mfCMPs show multiple broad peaks across many orders of magnitude of size, demonstrating aggregation in the solvent used during HPLC purification despite heating of the column. We hypothesize that peaks below 10 nm in size correspond to the triple helix, while peaks at larger sizes correspond to larger assembled structures or aggregates. For each peptide, independent samples (*n* = 3) were measured and averaged.

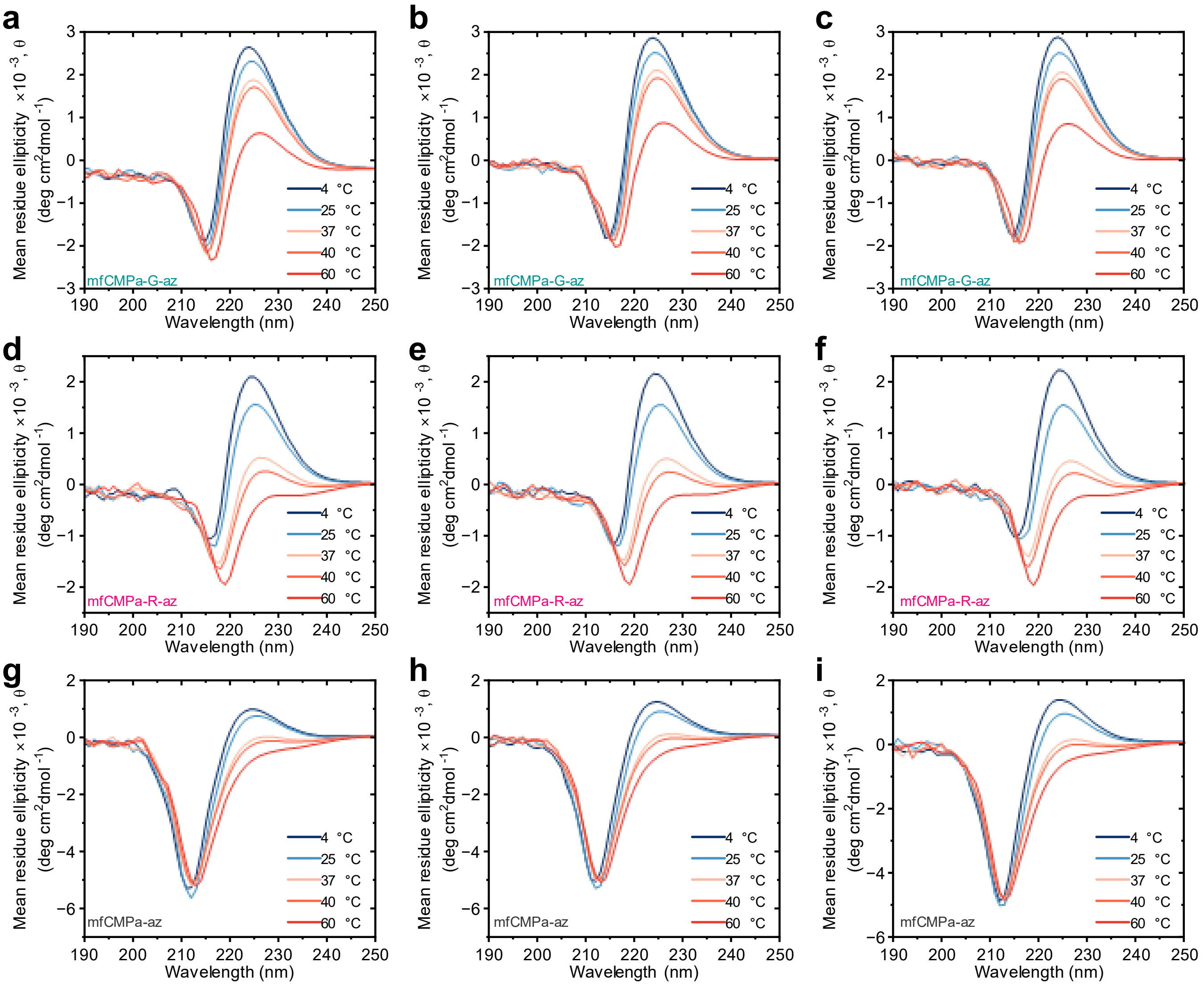

Figure S9. CD wavelength scans for a-c) mfCMPa-G-az, d-f) mfCMPa-R-az, and g-i) mfCMPa-az in DPBS measured at 0.3 mM after assembly. For each, (n = 3) independent samples were measured.

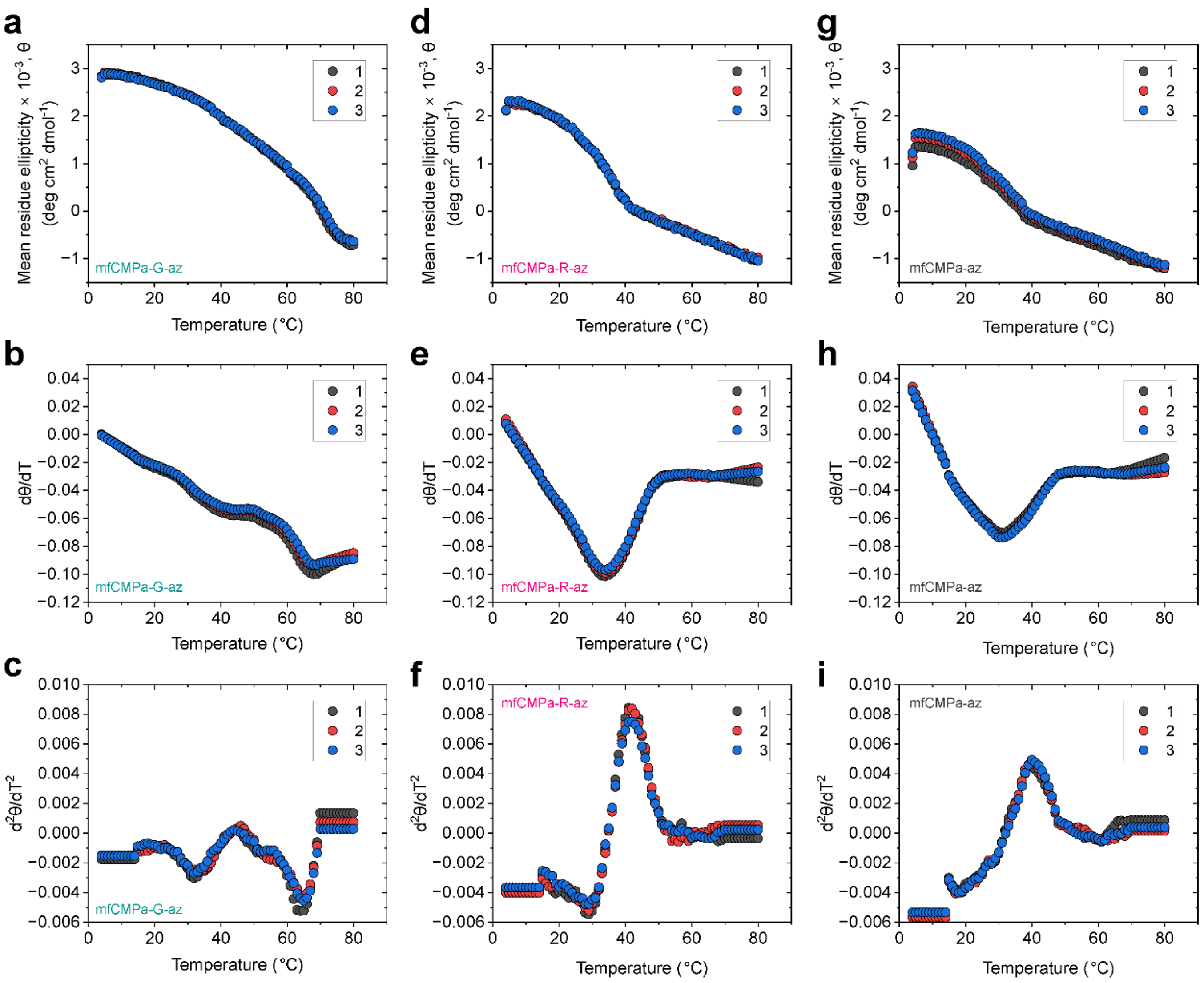

Figure S10. Temperature scans of mfCMPs at 225 nm with their first- and second-order derivatives. a-c) mfCMPa-G-az, d-f) mfCMPa-R-az, and g-i) mfCMPa-az in DPBS measured at 0.3 mM after assembly. For each, (*n* = 3) independent samples were measured. The two points where the second order derivative plot crosses zero with the highest changes in magnitude were identified and reported as the prominent melting temperatures (**Figure 2**).

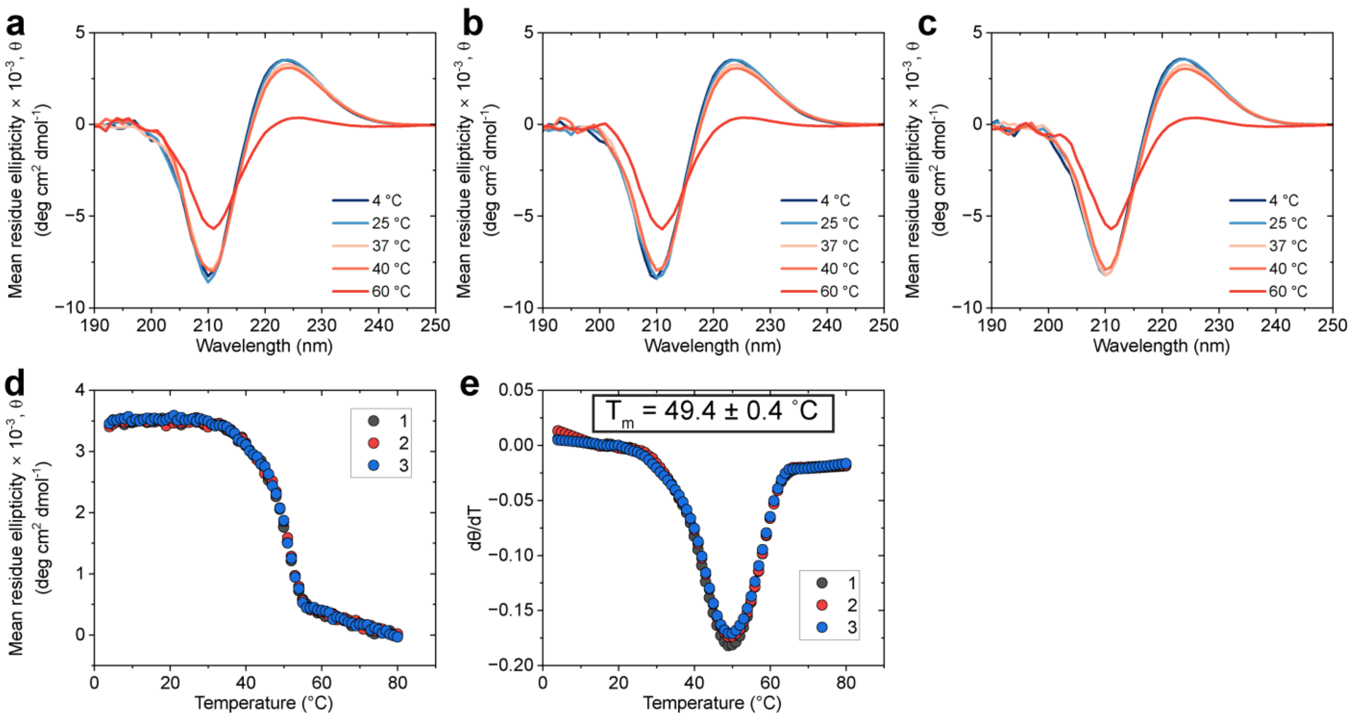

Figure S11. CD measurements for pendent alloc-functionalized GFOGER peptide in DPBS measured at 0.3 mM after assembly. a-c) Wavelength scans, d) temperature scans, and e) first-order derivatives of the temperature scan curves. For each type of scan, (n = 3) independent samples were measured.

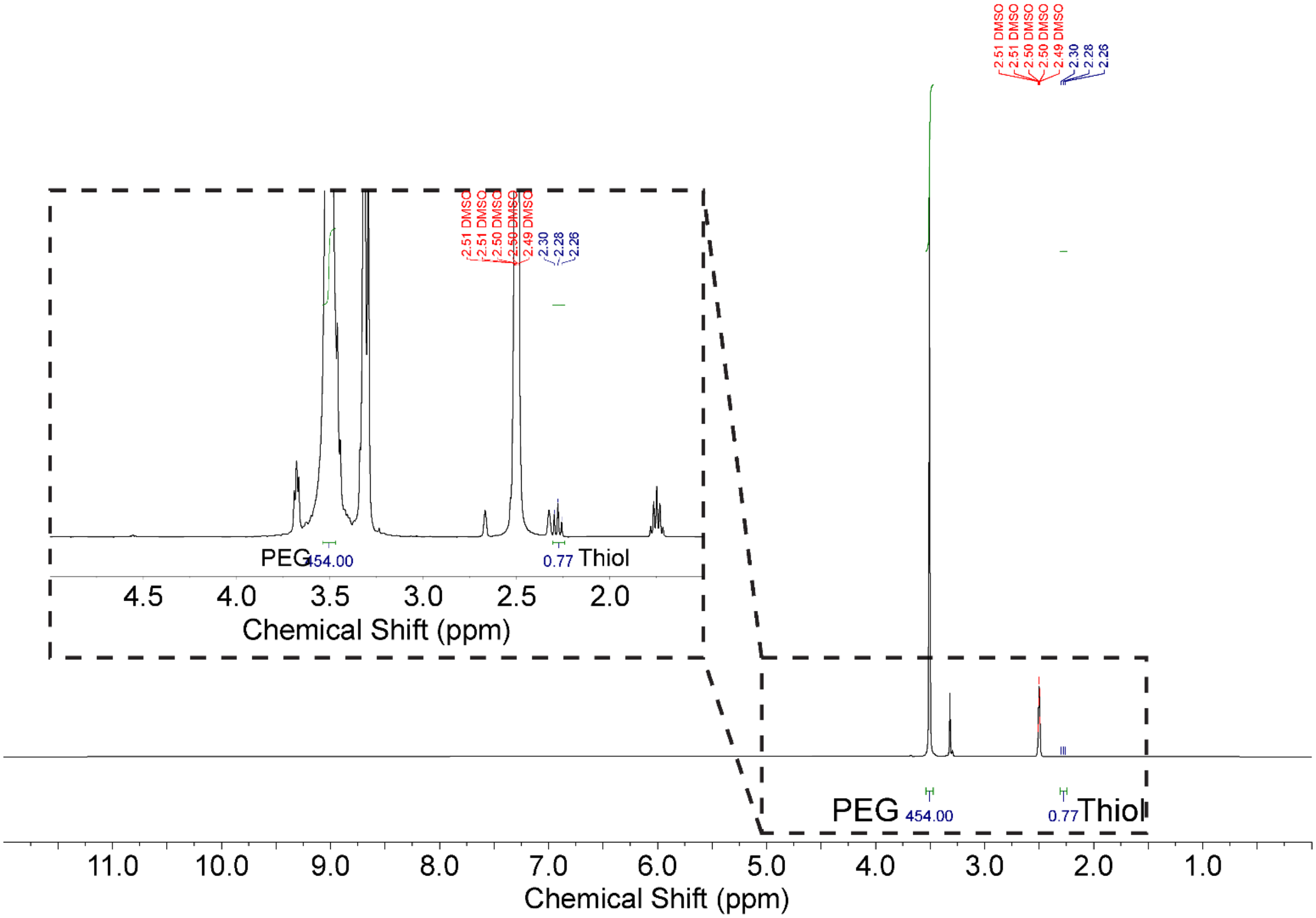

Figure S12. ^{1}H NMR spectrum for synthesized PEG-SH.

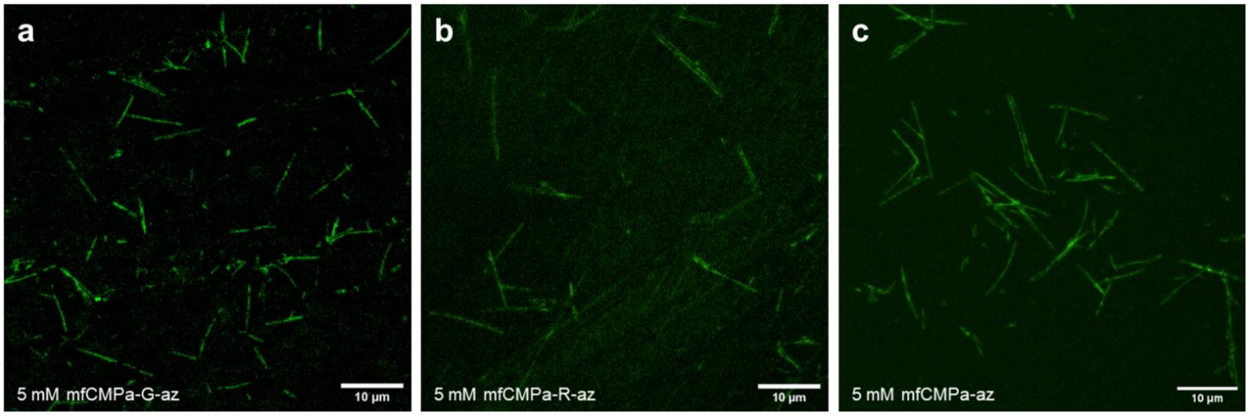

Figure S13. Confocal imaging of mfCMPs in PEG hydrogels. a) mfCMPa-G-az. b) mfCMPa-R-az. c) mfCMPa-az.

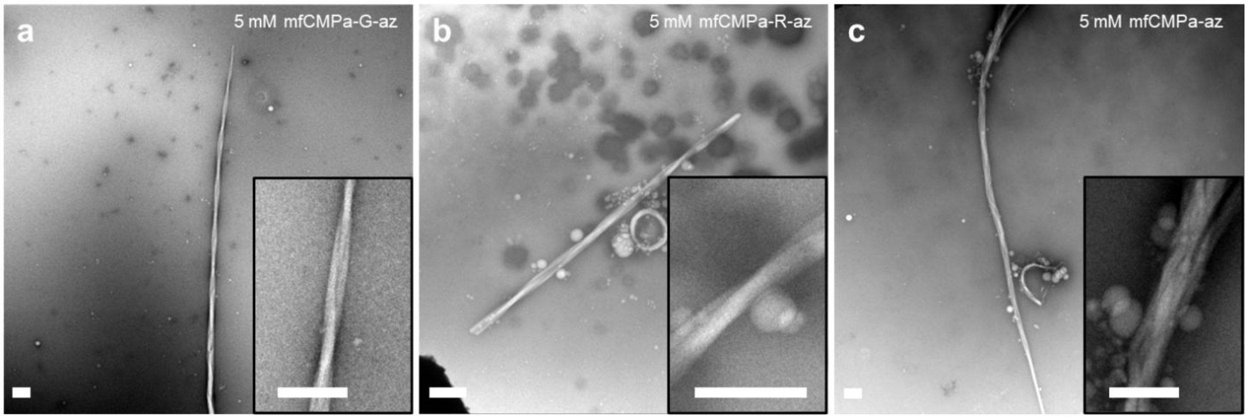

Figure S14. Fibrillar characterization of mfCMPs by TEM. a) mfCMPa-G-az, b) mfCMPa-R-az, c) mfCMPa-az. (scale bars = 375 nm).

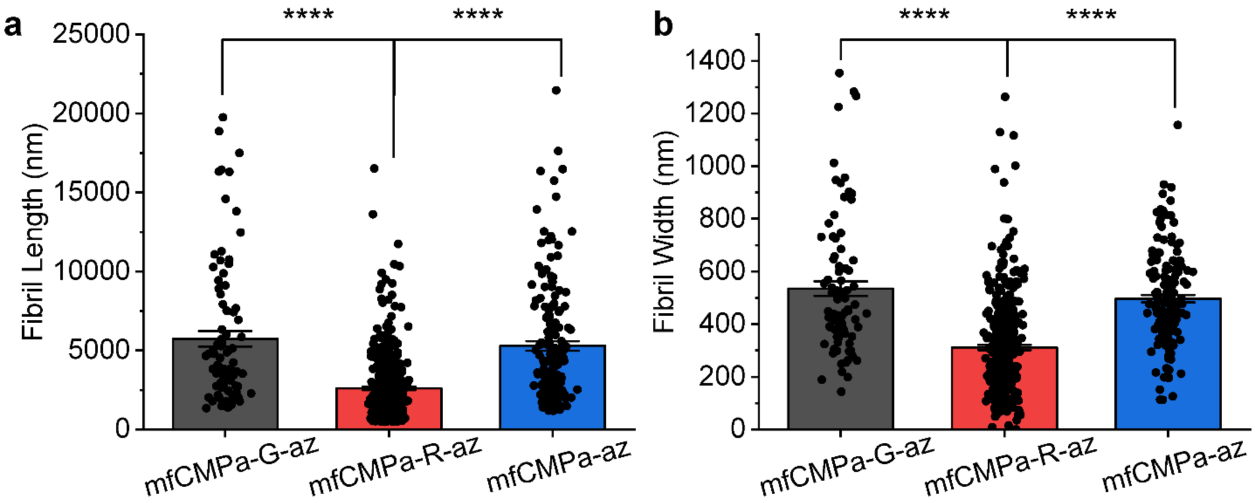

Figure S15. mfCMP fibril a) length and b) width analysis and comparison for mfCMPa-G-az, mfCMPa-R-az, and mfCMPa-az from STORM images (representative images shown in **Figure 3**), where statistical differences were observed for mfCMPa-R-az fibril lengths and widths relative to mfCMPa-G-az and mfCMPa-az (>70 fibrils measured for each mfCMP; ****$p < 0.0001$).

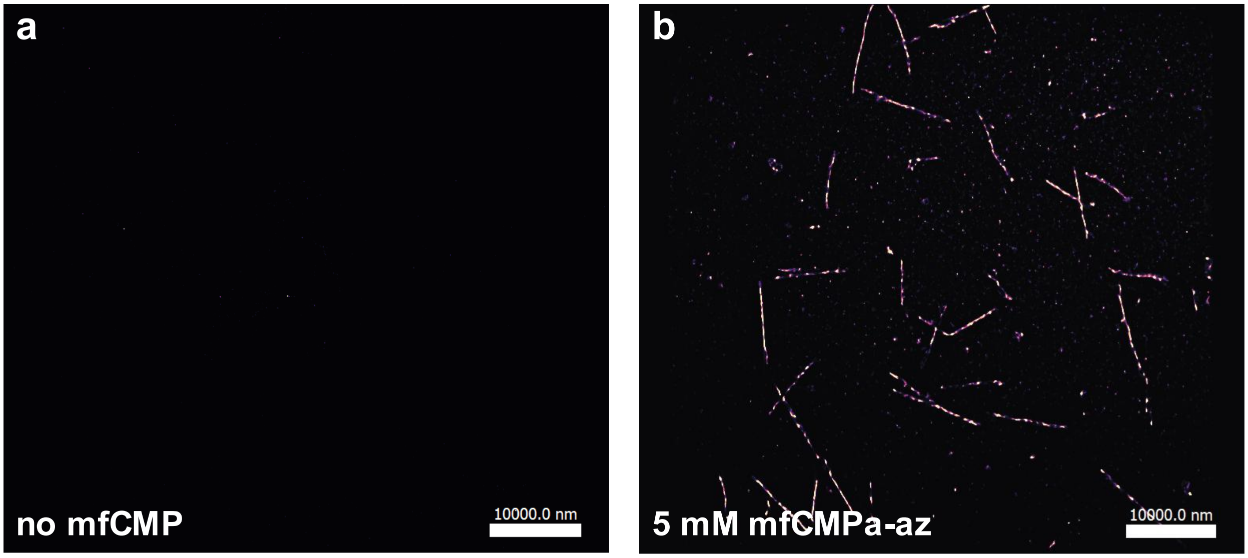

Figure S16. STORM imaging of PEG hydrogel containing a) no mfCMP compared to b) 5 mM mfCMPa-az.

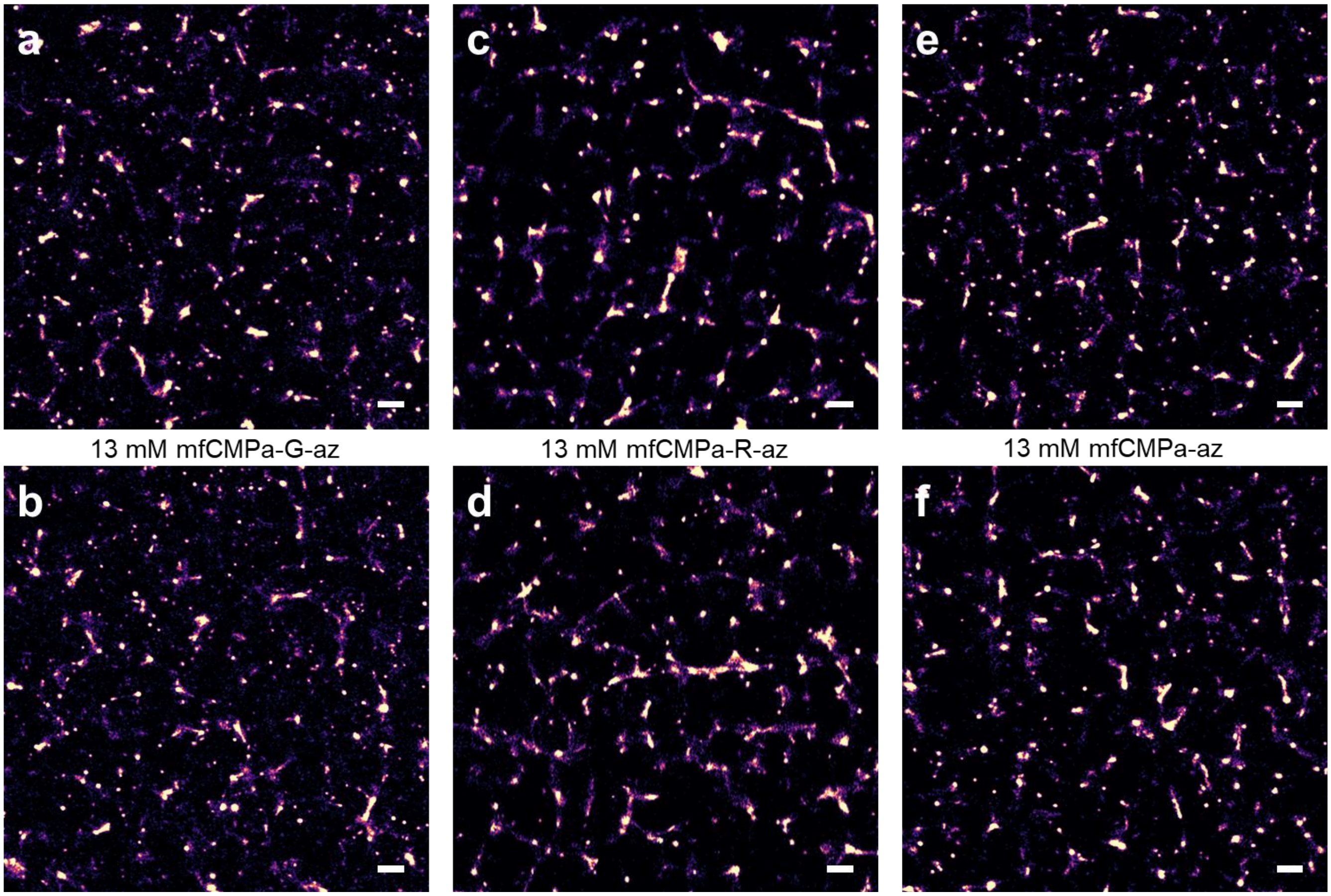

Figure S17. STORM imaging of PEG hydrogel containing mfCMPs at 13 mM. a-b) mfCMPa-G-az, c-d) mfCMPa-R-az, and e-f). mfCMPa-az. Scale bar = 500 nm.

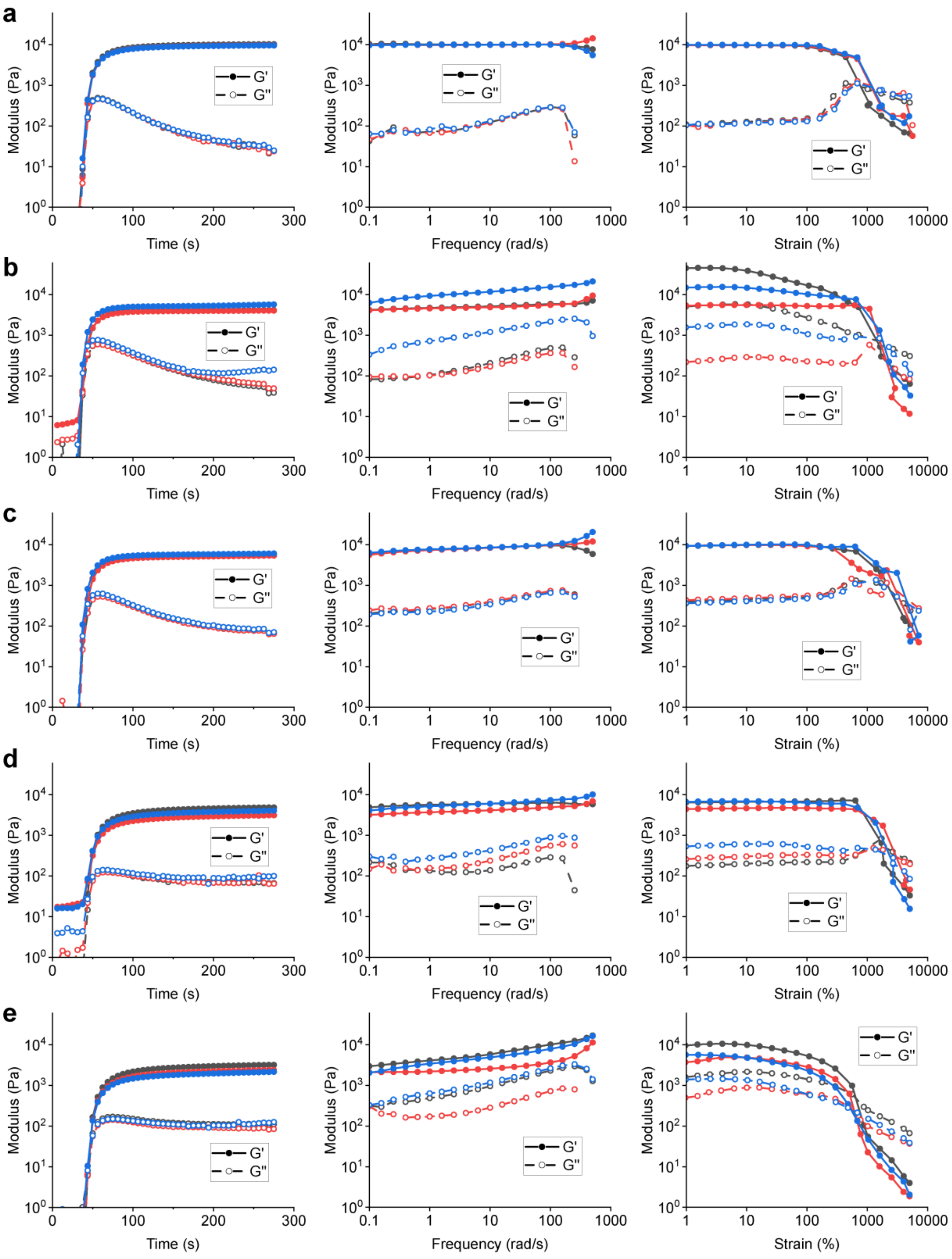

Figure S18. In situ rheology of mfCMP-PEG hydrogels with increasing concentrations of mfCMPa-az: a) 0 mM, b) 5 mM, c) 9 mM, d) 13 mM, and e) 20 mM. Gelation time sweeps, frequency sweeps, and strain sweeps were performed for (n = 3) hydrogels per condition.

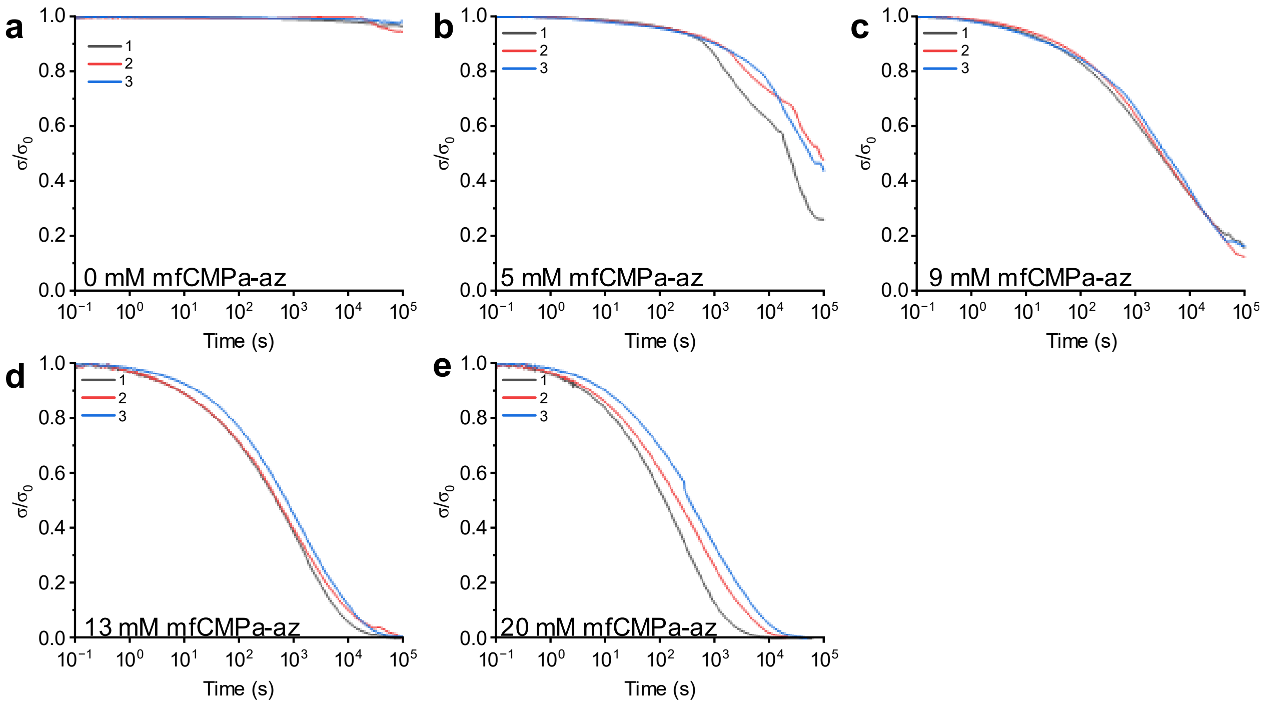

Figure S19. Stress relaxation profiles for mfCMPa-az at a) 0 mM, b) 5 mM, c) 9 mM, d) 13 mM, and e) 20 mM. For each concentration, (n = 3) hydrogels were measured.

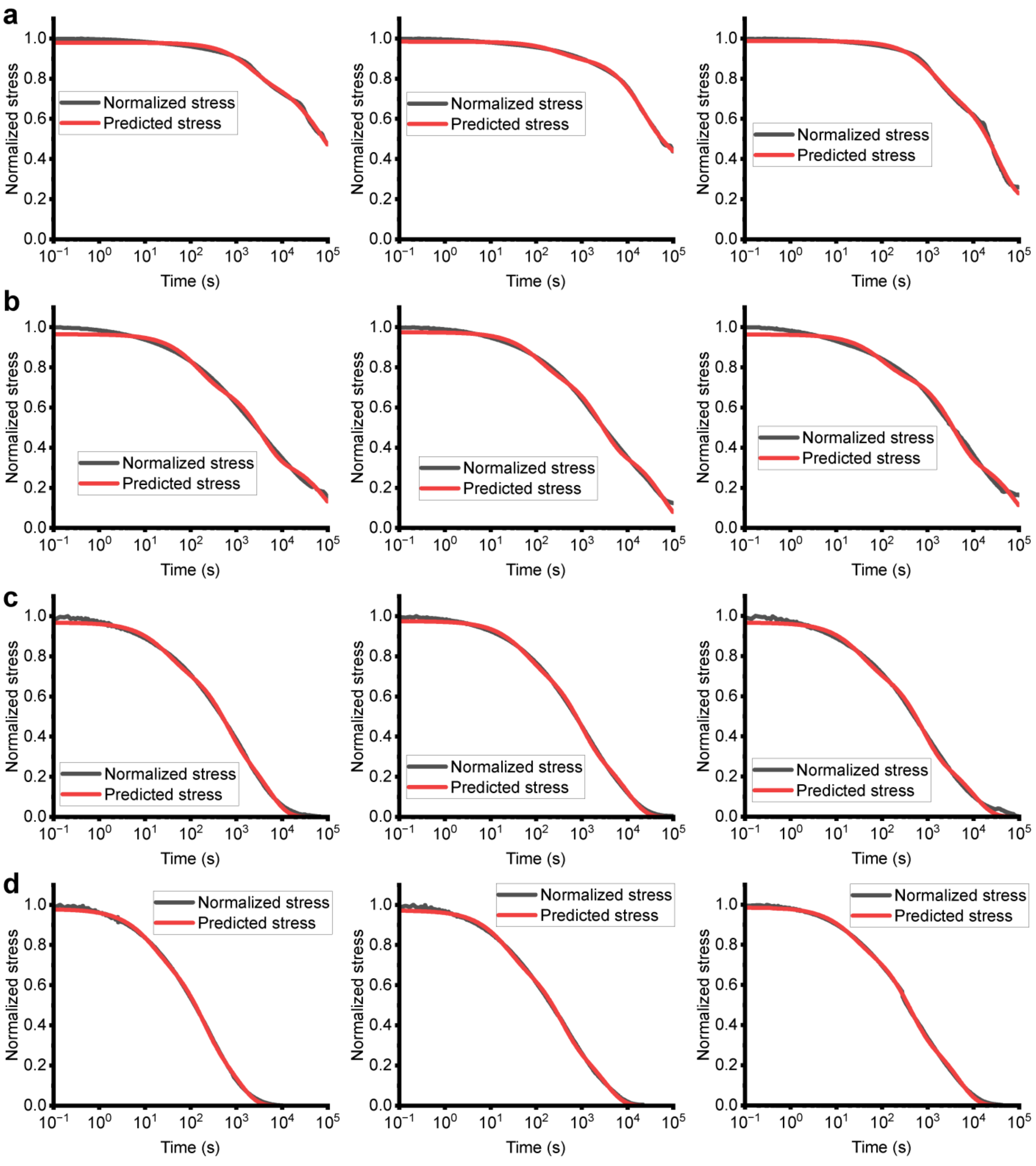

Figure S20. Stress relaxation curves containing mfCMPa-az at a) 5 mM, b) 9 mM, c) 13 mM, and d) 20 mM fitted to the generalized three mode Maxwell model.

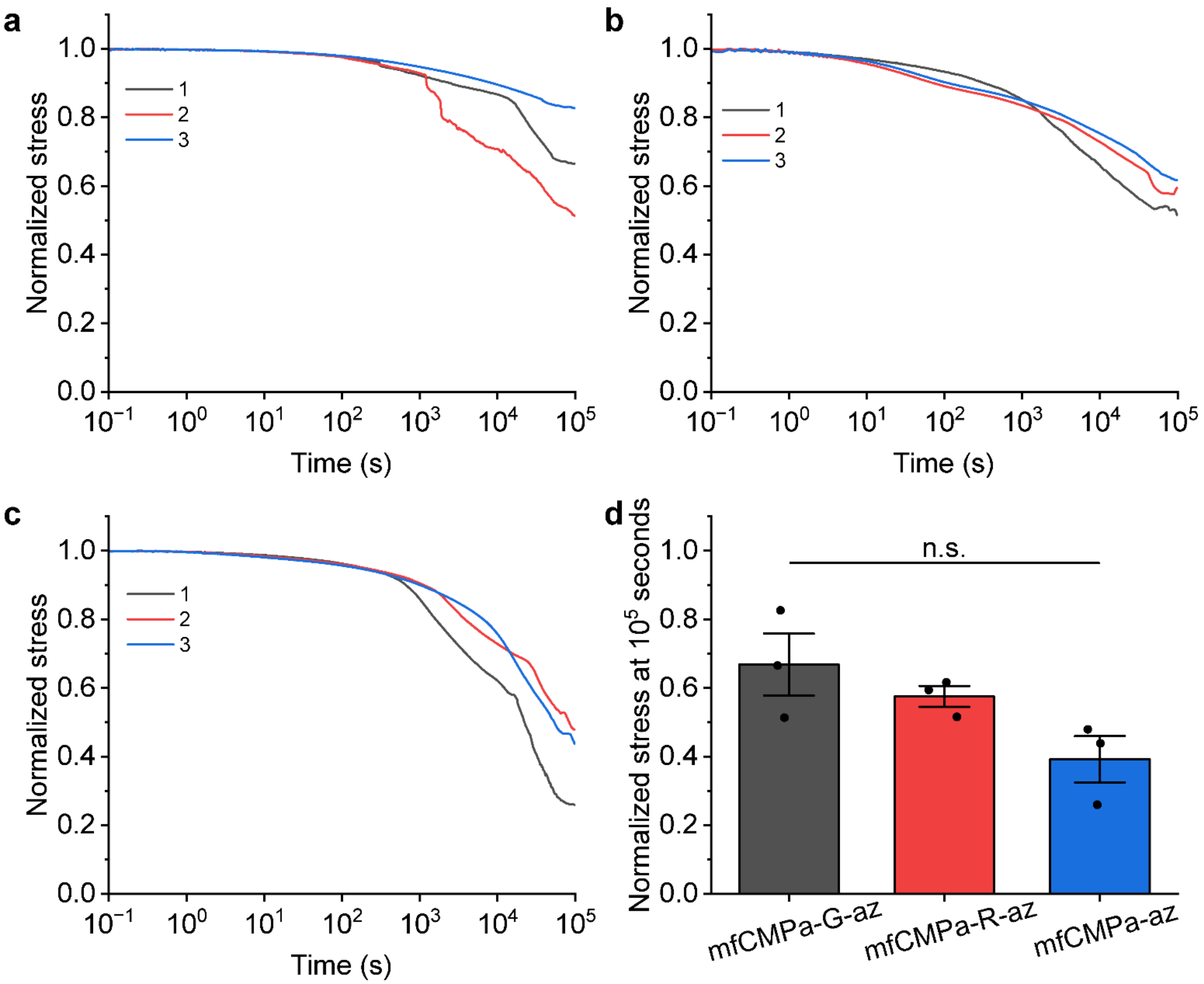

Figure S21. Stress relaxation behavior for hydrogels containing 5 mM a) mfCMPa-G-az, b) mfCMP-R-az, and c) mfCMPa-az. Stress is normalized to the maximum stress measured for each sample. Note, data shown in panel c) are the same as shown in Figure 4 and Figure S15 and are included here for ease of comparison. d) Comparison of stress relaxation at 10^5 seconds for these compositions. Means ± standard error are shown for each condition for (n = 3) independent sample measurements. Statistical significance was determined by one-way ANOVA with Tukey's multiple comparisons test (n.s. = no statistical difference).

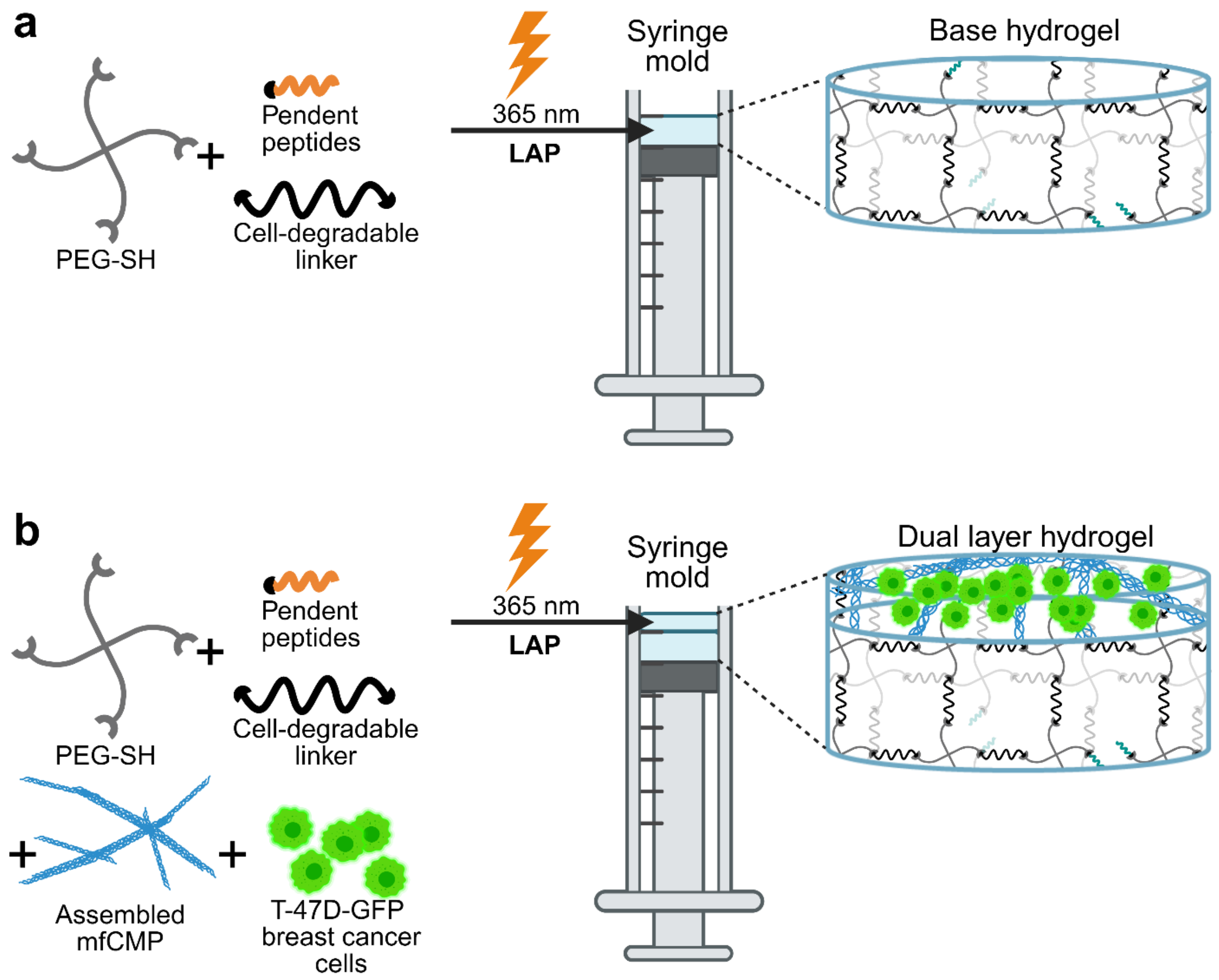

Figure S22. Schematic showing how dual-layer hydrogels were made for cell encapsulation experiments. a) First, PEG-SH, any pendent peptides, cell degradable linker peptide, and LAP are mixed and added to a syringe mold, then photocrosslinked. b) Next, the same monomer components are mixed and added to lyophilized mfCMP, then added to a cell pellet. After mixing again, this precursor solution is added to the same syringe mold containing the base layer, and photocrosslinked.

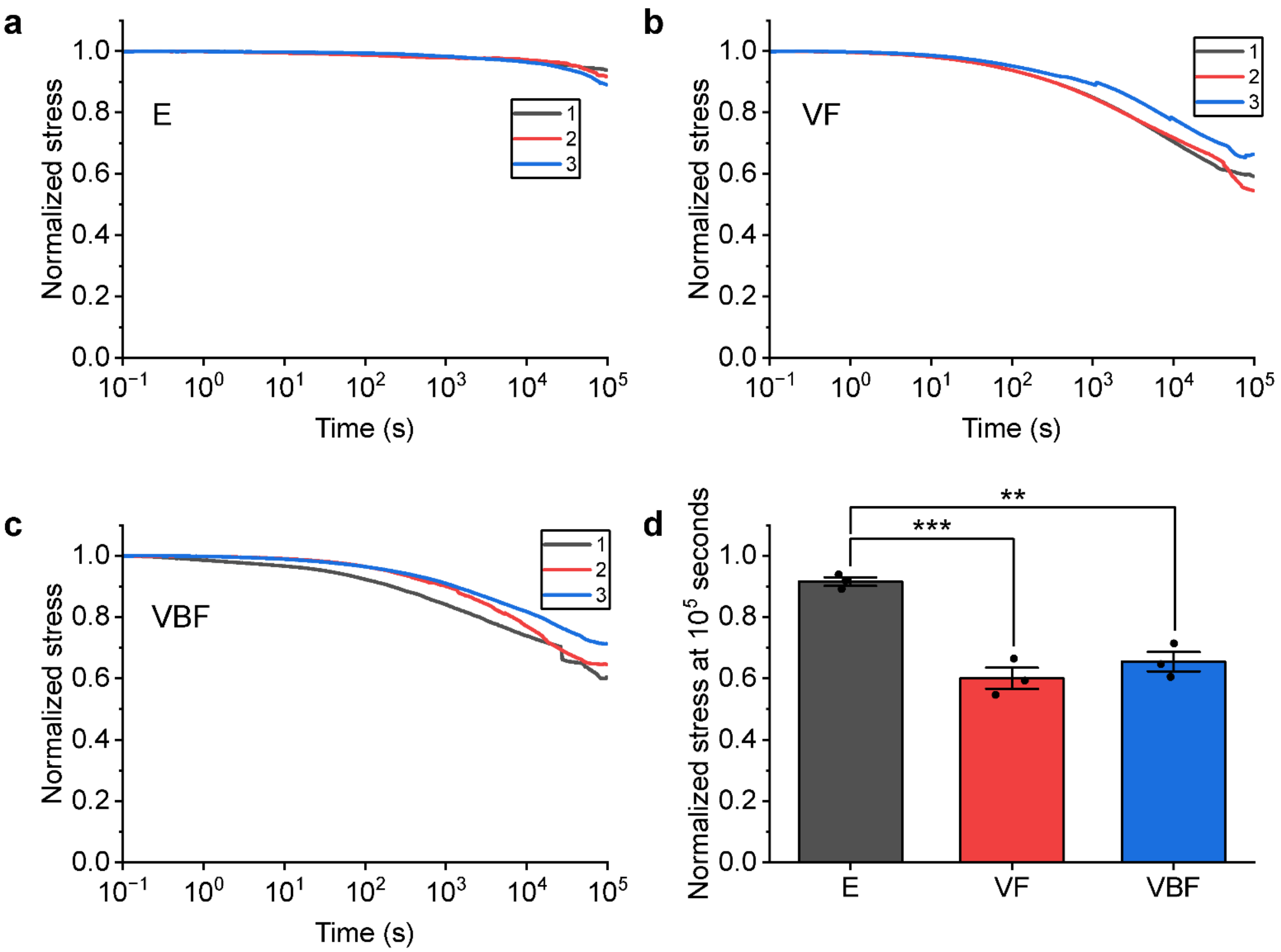

Figure S23. Stress relaxation behavior of hydrogel formulations used in cellular studies: a) E, b) VF, and c) VBF. Stress is normalized to the maximum stress measured for each sample. d) Comparison of average values for stress relaxation at 10^5 seconds for these compositions. Means ± standard error are shown for each condition for (n = 3) independent sample measurements. Statistical significance was determined by one-way ANOVA with Tukey's multiple comparisons test. Statistical significance is shown (*$p < 0.05$; **$p < 0.01$; ***$p < 0.001$; ****$p < 0.0001$).

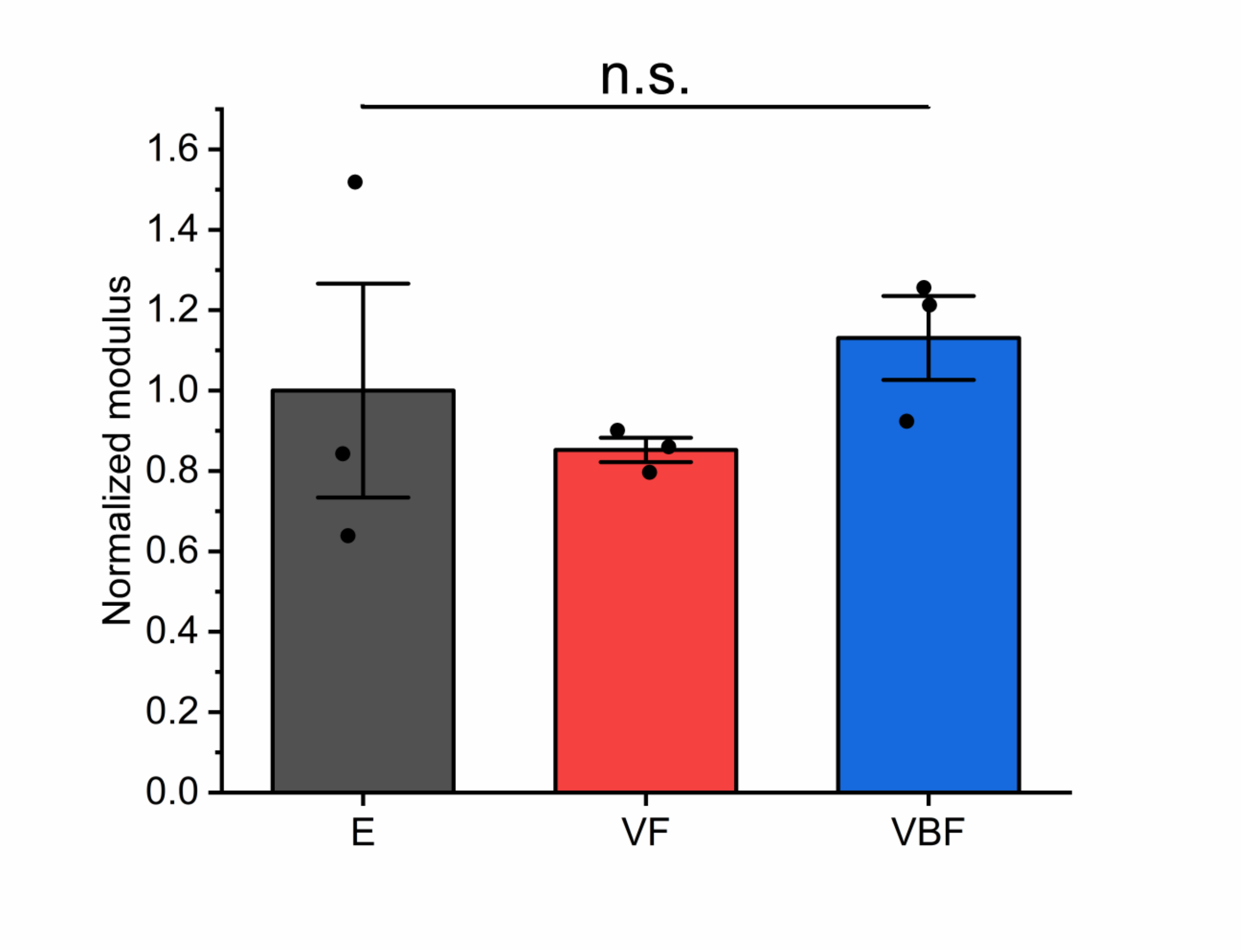

Figure S24. Equilibrium-swollen storage moduli of E, VF, and VBF hydrogel compositions, normalized to the E condition. Means ± standard error are shown for each condition for (n = 3) independent sample measurements. Statistical significance was determined by one-way ANOVA with Tukey's multiple comparisons test (n.s. = no statistical significance).

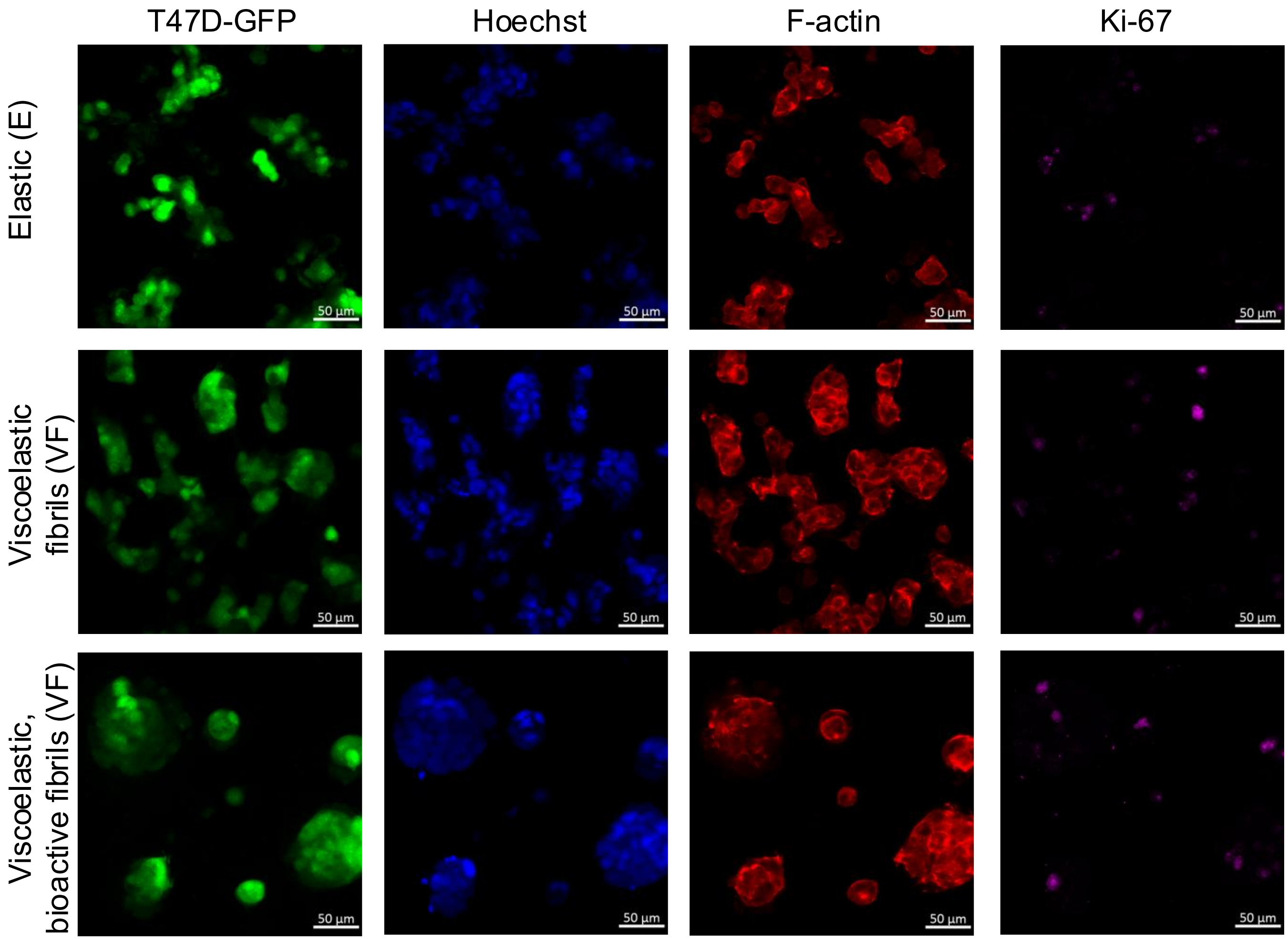

Figure S25. Individual fluorescence channel images of T47D-GFPs in mfCMP-PEG hydrogels after 7 days of culture, fixation, and immunostaining.

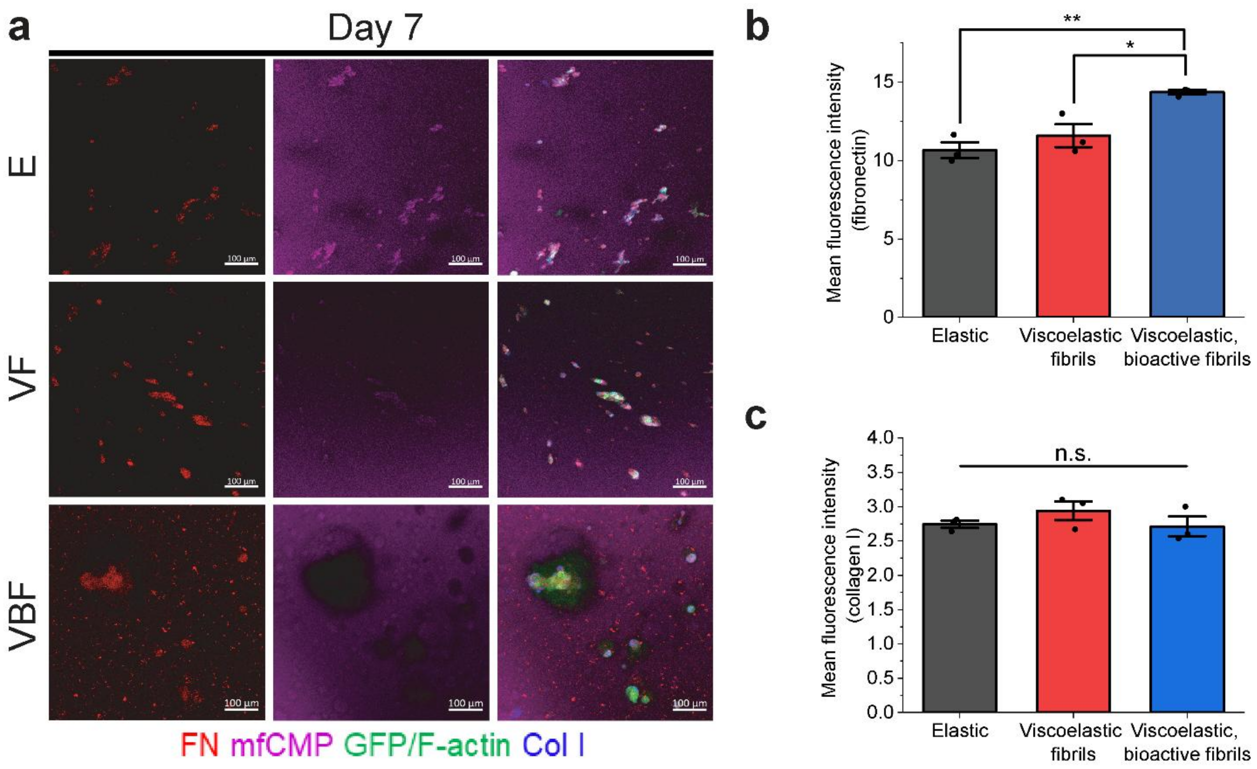

Figure S26. Secreted protein staining. a) Hydrogels with encapsulated T47D cells were stained for fibronectin, F-actin, collagen I, and mfCMP. Mean fluorescence intensity of immunostained b) fibronectin and c) collagen I in E, VF, and VBF hydrogels 7 days after encapsulation of T47Ds. Note that in all conditions, there remains background fluorescence from the alkyne-AlexaFluor 647 used to label mfCMP. Means ± standard error are shown for each condition for (n = 3) independent sample measurements. Statistical significance was determined by one-way ANOVA with Tukey's multiple comparisons test. Statistical significance is shown (*p < 0.05; **p < 0.01).

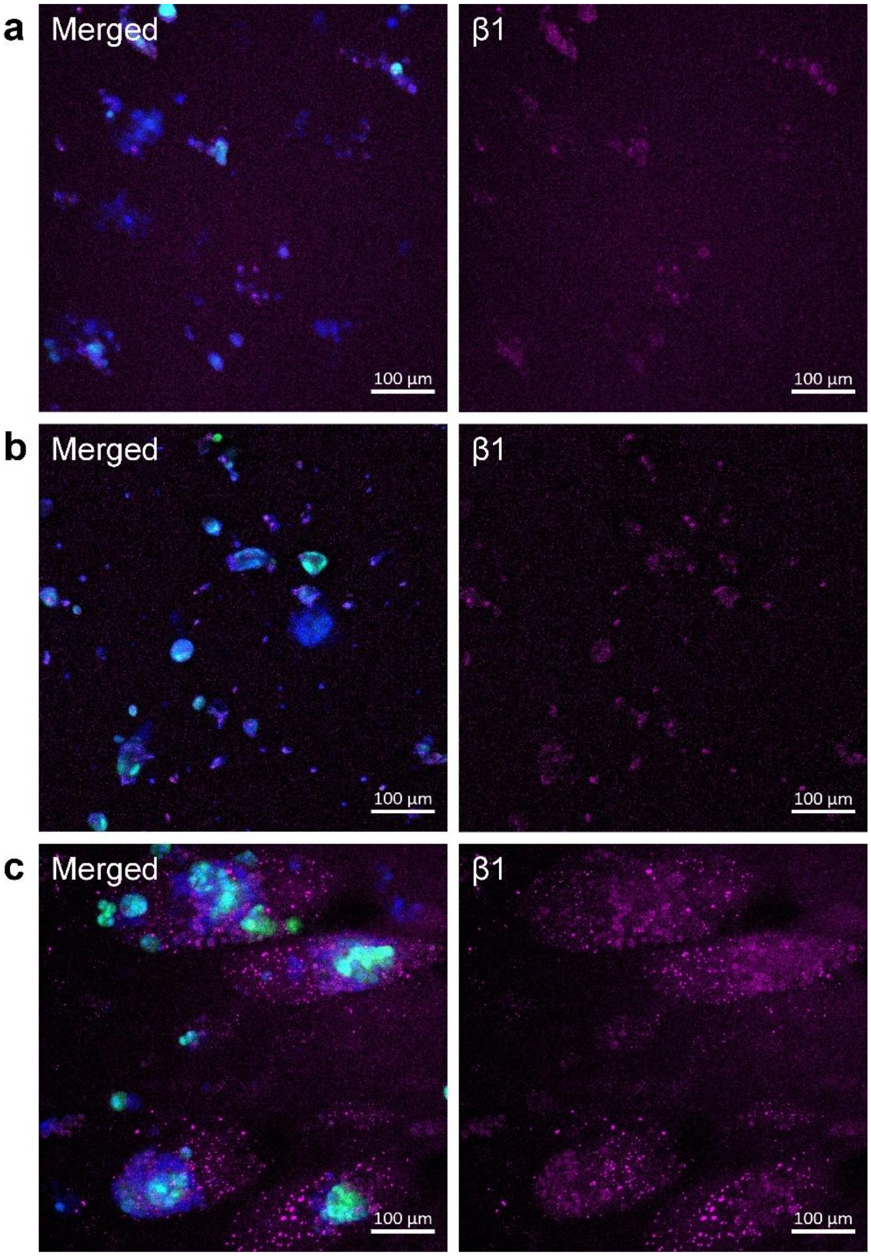

Figure S27. β1 integrin staining of fixed hydrogels for conditions a) E, b) VF, and c) VBF. Fixed samples and stained (nuclei, blue; GFP, green; β1 integrin, magenta; scale bars = 100 µm). We hypothesize that the fluorescent puncta observed for β1 integrin in VBF condition are clustered integrins in response to the fibrillar, bioactive mfCMPs. Note, α2 integrin, which is associated with α2β1 binding to collagen I, is not internalized intracellularly when integrin turnover occurs,[9] which we hypothesize leads to some retention of integrins surrounding some cell clusters.

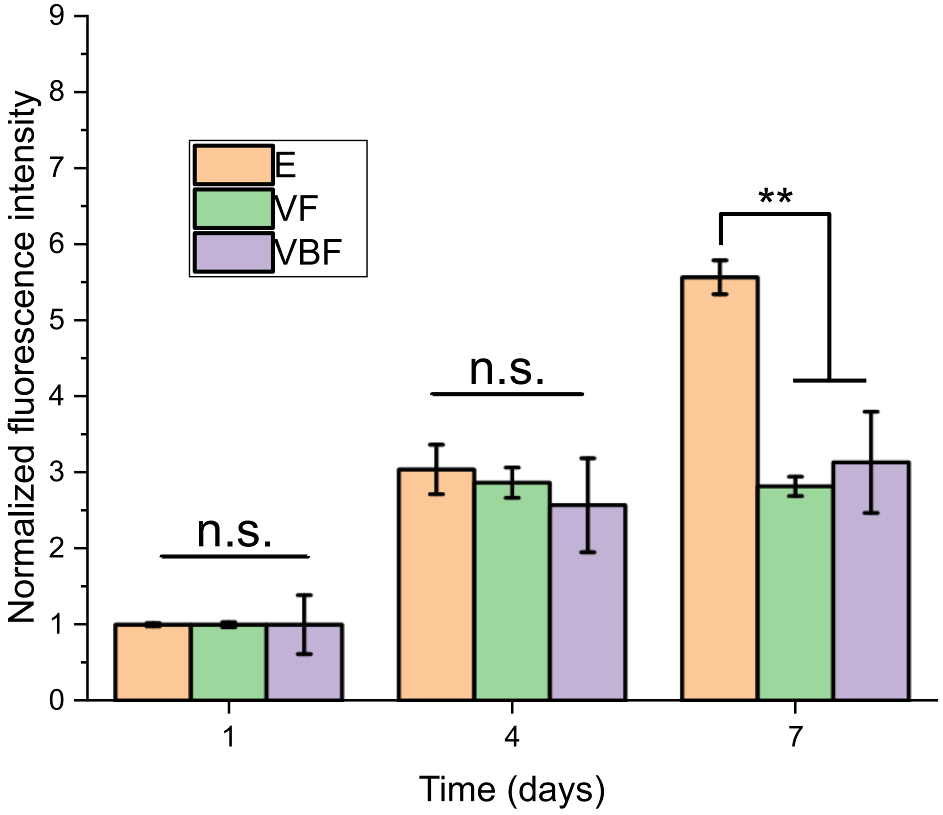

Figure S28. Metabolic activity of T47Ds in mfCMP-PEG hydrogels (conditions E, VF, VBF) on days 1, 4, and 7. Each condition is normalized to day 1. Means ± standard error are shown for each condition for (n = 4) independent sample measurements. Statistical significance was determined by one-way ANOVA with Tukey's multiple comparisons test. Statistical significance is shown (*$p < 0.05$; **$p < 0.01$; ***$p < 0.001$; ****$p < 0.0001$).

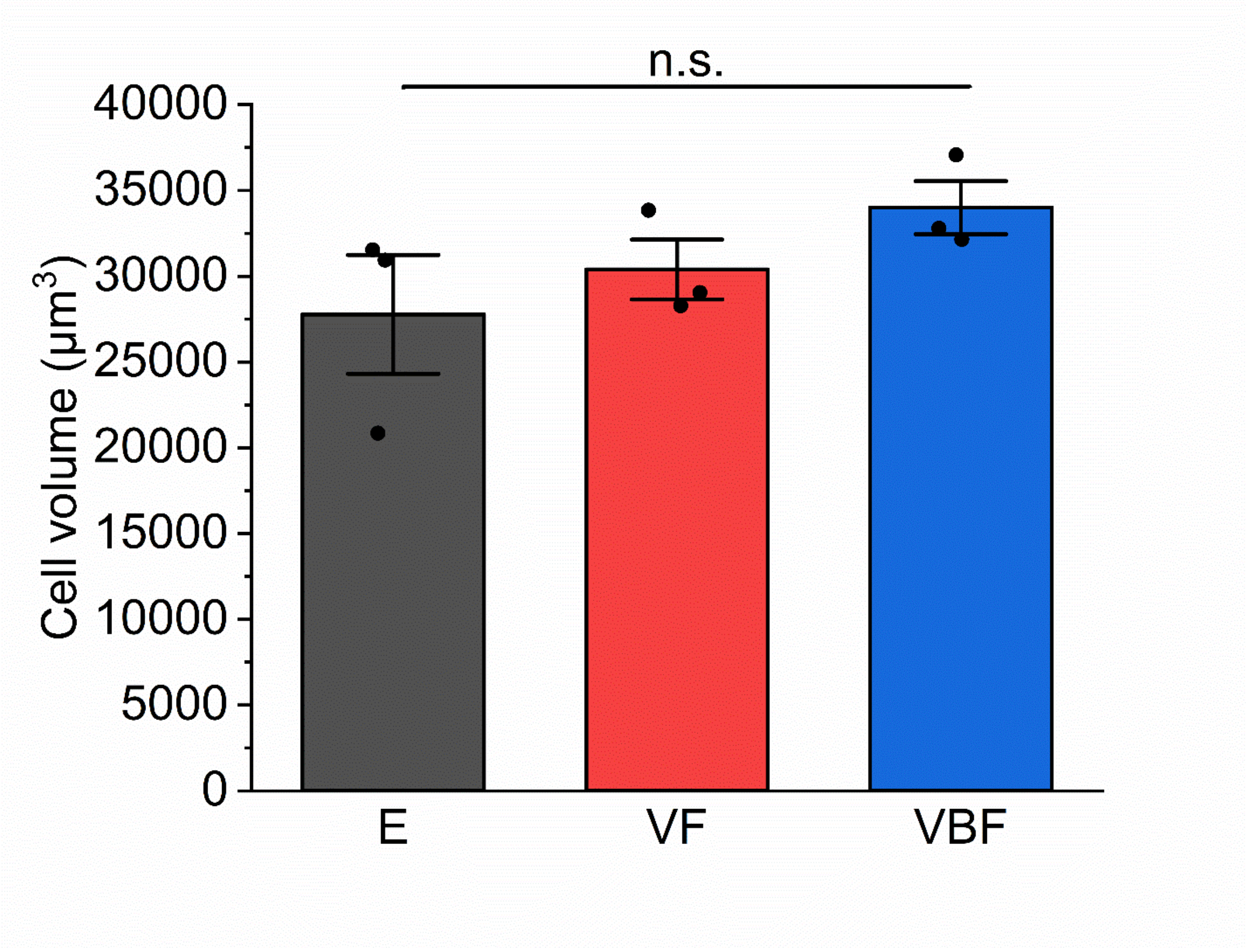

Figure S29. Comparison of mean cell volume for T47Ds in mfCMP-PEG hydrogels (conditions E, VF, VBF) on day 7 based on F-actin fluorescence. Means ± standard error are shown for each condition for (n = 3) independent averaged sample measurements. Statistical significance was determined by one-way ANOVA with Tukey's multiple comparisons test. Statistical significance is shown (*$p < 0.05$; **$p < 0.01$; ***$p < 0.001$; ****$p < 0.0001$).

Table S1. Fitted 3-mode Maxwell model parameters for hydrogels containing increasing concentrations of mfCMPa-az.

[mfCMPa-az] (mM)	A_1	A_2	A_3	τ_1	τ_2	τ_3
5	0.162 ± 0.048	0.390 ± 0.095	0.432 ± 0.12	1250 ± 520	31000 ± 5600	10300000 ± 980000
9	0.196 ± 0.018	0.406 ± 0.013	0.366 ± 0.017	111 ± 12	3140 ± 370	83100 ± 12000
13	0.200 ± 0.0093	0.417 ± 0.018	0.353 ± 0.023	36.8 ± 7.4	681 ± 88	6690 ± 1600
20	0.203 ± 0.017	0.421 ± 0.011	0.356 ± 0.010	17.7 ± 2.7	111 ± 64	2860 ± 1000

Table S2. Concentrations of monomers used to form hydrogels for in situ gelation time sweeps, frequency sweeps, strain sweeps, and stress relaxation experiments.

	0 mM	5 mM	9 mM	13 mM	20 mM
PEG (mM thiol)	20	20	20	20	20
Linker Peptide (mM alloc)	18	13	9	5	0
mfCMPa-az (mM alloc)	0	5	9	13	20
Pendent RGD (mM alloc)	2	2	2	2	0

Table S3. Concentrations of monomers used to form hydrogels for stress relaxation experiments.

	E	VF	VBF
PEG (mM thiol)	20	20	20
Linker Peptide (mM alloc)	13	13	13
mfCMPa-az (mM alloc)	0	5	3
mfCMPa-G-az (mM alloc)	0	0	1
mfCMPa-R-az (mM alloc)	0	0	1
Pendent RGD (mM alloc)	1	1	0
Pendent GFOGER (mM alloc)	1	1	0

REFERENCES

1. E. M. Ford, A. M. Kloxin, "Rapid Production of Multifunctional Self-Assembling Peptides for Incorporation and Visualization within Hydrogel Biomaterials," *ACS Biomaterials Science & Engineering* **2021**, 7, 4175, https://doi.org/10.1021/acsbiomaterials.1c00589

2. L. A. Sawicki, A. M. Kloxin, "Design of thiol–ene photoclick hydrogels using facile techniques for cell culture applications," *Biomater. Sci.* **2014**, 2, 1612, https://doi.org/10.1039/c4bm00187g

3. B. D. Fairbanks, M. P. Schwartz, C. N. Bowman, K. S. Anseth, "Photoinitiated polymerization of PEG-diacrylate with lithium phenyl-2,4,6-trimethylbenzoylphosphinate: polymerization rate and cytocompatibility," *Biomaterials* **2009**, 30, 6702, https://doi.org/10.1016/j.biomaterials.2009.08.055

4. A. M. Hilderbrand, E. M. Ford, C. Guo, J. D. Sloppy, A. M. Kloxin, "Hierarchically structured hydrogels utilizing multifunctional assembling peptides for 3D cell culture," *Biomaterials Science* **2020**, 8, 1256, https://doi.org/10.1039/c9bm01894h

5. D. Wu, N. Sinha, J. Lee, et al., "Polymers with controlled assembly and rigidity made with click-functional peptide bundles," *Nature* **2019**, 574, 658, https://doi.org/10.1038/s41586-019-1683-4

6. J. Schnitzbauer, M. T. Strauss, T. Schlichthaerle, F. Schueder, R. Jungmann, "Super-resolution microscopy with DNA-PAINT," *Nature Protocols* **2017**, 12, 1198, https://doi.org/10.1038/nprot.2017.024

7. L. Pradhan, D. Moore, E. M. Ovadia, et al., "Dynamic bioinspired coculture model for probing ER+ breast cancer dormancy in the bone marrow niche," *Science Advances* **2023**, 9, https://doi.org/10.1126/sciadv.ade3186

8. L. A. Sawicki, E. M. Ovadia, L. Pradhan, et al., "Tunable synthetic extracellular matrices to investigate breast cancer response to biophysical and biochemical cues," *APL Bioengineering* **2019**, 3, 016101, https://doi.org/10.1063/1.5064596

9. N. Rintanen, M. Karjalainen, J. Alanko, et al., "Calpains Promote A2β1 Integrin Turnover in Nonrecycling Integrin Pathway," *Molecular Biology of the Cell* **2012**, 23, 448, https://doi.org/10.1091/mbc.e11-06-0548
