# Supplementary material for: Synthetic Surrogates of Collagen-Rich Microenvironments: Integrating Modular Bioactive Fibrillar Structure and Tunable Viscoelasticity via Multifunctional Assembling Peptides
Source: ACS Cent Sci. 2026 May 16;12(6):777–88. doi: 10.1021/acscentsci.5c02175 (PMC13306593; doi:10.1021/acscentsci.5c02175)
Supplement: Supplementary file 3 [file oc5c02175_si_003.pdf]

oc-2025-021753.R1

Name: Peer Review Information for "Synthetic Surrogates of Collagen-Rich Microenvironments: Integrating Modular Bioactive Fibrillar Structure and Tunable Viscoelasticity via Multifunctional Assembling Peptides"

First/Second/Third Round of Reviewer Comments

Reviewer: 1

Comments to the Author

Here, the authors reported a fully synthetic polymer-peptide composite that mimics the behavior of collagen-rich extracellular matrices (ECM). The authors first designed specific, modular peptide sequences, functionalized with alkene pendant groups for use as crosslinkers in thiol-functionalized hydrogel precursors, and with azide-alkyne groups for functionalization with fluorescent probes for imaging. The stability and helical structure of the peptide sequences was characterized well with circular dichroism spectroscopy, and they displayed stable triple helix formation, similar to collagen. These peptides were incorporated into covalently crosslinked hydrogels based on a 4-arm polyethylene glycol (PEG)-thiol. Rheological measurements showed that the inclusion of peptides induced concentration-dependent viscoelastic behavior in the otherwise elastic hydrogels. The authors also studied the effect of this mechanical behavior on cell growth.

The manuscript is well-written and well-organized, and the authors have used a range of different techniques to characterize material properties. However, some unanswered questions remain, particularly about the purity of the peptides, quantification from microscopy images, and mechanical characterization.

Comments:

1. UPLC-MS traces are provided to characterize the peptide synthesis, but many of these have multiple peaks or peaks with shoulders. Is it possible to extract and report a percent purity for the synthesized peptides? Please note the retention times of the peptides and any associated side products in the MS traces (Figures S1-S4).

2. In the CD spectroscopy data, there is a shift in the peak at 215 nm as the temperature is increased. What molecular mechanism or change in self-assembly is this shift attributed to? In addition, two of the three fibrils melt before 37 °C. How does this impact fibril presentation during cell culture?

3. Figure 2e: It looks like there are multiple melting events for the first-order derivative plot of sample “mfCMPa-G-az”. Please discuss how the melting points were determined in this case, and why other minima were not considered for this assessment.

4. Page 13, Line 6: Please change nomenclature to “hydrogels were formed…(not polymerized)” as the process described here is a crosslinking, not a polymerization. Throughout the manuscript, it would be helpful to replace the term “photopolymerization” with “photocrosslinking” as the chemistry described here is a photoinitiated thiol-ene crosslinking – not polymerization from a monomer block.

5. In general, the data in Figure 3 is intriguing but largely qualitative. How does the inclusion of different peptide sequences affect fibrillar characteristics? Please quantify differences between G, R, and control via image analysis. It would be helpful to quantify connectivity, fiber length, diameter, and compare across different samples.

6. For improved clarity, please include a table of hydrogel formulations used in this study and a schematic of how the hydrogels were made. Please also specify why 5 mM concentration was used.

7. Page 17, line 48-49: Please specify what is meant by physical crosslinks.

8. Page 18, line 4: Please clarify how long the gels were stabilized before measurements were started (e.g., 105 s as mentioned in the manuscript?). Please also include these details in the methods section of the SI.

9. Figure 4: Please enhance Figure 4a with a Chemdraw of crosslinking between the alkene groups on the peptide, cell-degradable linkers, and the PEG-SH.

10. Figure 4 only contains measurements for the control mfCMP condition; however, Figure 5 uses mfCMPa-R-az and assumes viscoelastic behavior. It would be helpful and rigorous to support this assumption with rheological data.

11. Figure 4b: What was the fitting used for the half-lives in this figure?

12. Typically, viscoelastic behavior is noted from the frequency response of the gels, and from analyzing the crossover frequency and slope of G' and G" in the frequency sweep. The frequency range used in this manuscript is quite small. Please perform the frequency sweep over an expanded range for formulations with different concentrations of peptide.

13. Please include STORM imaging for the 20 mM gels. Would you expect to see a much more interconnected network here due to its stress relaxation behavior?

14. Please include stress relaxation data for the viscoelastic, bioactive hydrogels shown in Figure 5. Please include a table of the gels formulated for this study for clarity.

Miscellaneous:

1. Purification of peptides: On page 8 line 27 of the main manuscript it says HPLC was used for purification, but in the SI figures it says UPLC. Which type of liquid chromatography was used? Please, keep it consistent.

2. Could you add references for the statement of page 10 line 36? "Wavelength scans show a

characteristic polyproline type II peak at 225 nm associated with a collagen-like triple helix..."

3. Figure S7: Please show full NMR spectrum from 0 to 12 ppm. Please do not truncate relevant polymer peaks (i.e., please zoom out until the peaks are not cropped from the top). Please integrate and label all peaks of relevance.

Reviewer: 2

Comments to the Author

The authors have developed a fully synthetic materials system that captures much of the complexity of the native extracellular matrix: fibrillar architecture, viscoelastic mechanical properties, presentation of relevant bioactive signals (from the fibrillar structures), and proteolytic remodelability. The primary novelty derives from the use of the self-assembled fibrillar structures to (1) impart modes of viscous dissipation in the networks and (2)

present relevant cell-adhesive motifs. These materials thus capture two key facets of natural ECM proteins like collagen that are not typically found in engineered ECM materials. The authors demonstrate using super resolution microscopy that the fibers maintain their structure after assembly into the composite hydrogels and exhibit diameters similar to native collagen. Finally, the authors demonstrate biological relevance for their engineered system using a cancer cell spheroid growth assay.

Major comments:

1. A key advantage of the system is the ability to decouple cell adhesive ligand presentation from microstructure and mechanics in the system. From the STORM imaging, are the networks of the three different self-assembling peptides statistically similar in terms of their structural properties (e.g., fiber density, orientation, etc.)?

2. What is the cause of the larger spheroid volume in the bioactive CMP containing materials? Is it due to increased proliferation, or increased migration that allows the cells to cluster? Tracking the cells at day 1 vs. 4 vs. 7 using the existing data could provide insight. As the Ki67 levels at day 7 are not that different, if it is a proliferation effect, perhaps it is more pronounced at earlier time points. A third possibility is that the cells themselves take up more volume in materials that are more permissive (i.e., viscoelastic). Are the numbers of cells per field of view similar?

3. Potentially related to comment 2, are the materials viscoplastic? That is, are the physical crosslinks from the fibrils in sufficiently high concentration to allow physical rearrangement in the networks without proteolysis? This could be one mechanism by which the cells make space to permit larger spheroid formation.

4. Do the authors have a hypothesis as to why the gels presenting the adhesive sequences from the fibers, as opposed to pendant from the PEG network, yield the largest spheroids? This result highlights a key feature of their system, which is that adhesive sequences can be presented in more native-like conformations from the fibrils.

Minor comments:

1. On page 18, the mean t1/2 for the 20 mM mfCMP gels is listed as 125 s, but this does not seem to agree with the graphs in figure 4b,c.

2. The storage modulus for the 20 mM mfCMP networks is not included in figure 4d, despite the respective stress relaxation data being reported.

3. In figure S14, there appears to be staining for the mfCMP in the purely elastic sample, though I believe from the text that this sample should not contain any mfCMP.

Reviewer: 3

Comments to the Author

In their manuscript, Kloxin and coworkers describe the use of multifunctional collagen-mimetic peptide (mfCMP)-containing hydrogels to create synthetic collagen network mimics with tunable viscoelasticity. The authors extend their previous system of mfCMP-containing hydrogels to include mfCMPs embedded with integrin-binding sequences, thereby enabling the controlled presentation of distinct cell-binding sites for modeling both intact and denatured collagen I. The authors describe the design of their mfCMPs to include functional groups for (1) end-to-end fibril elongation via electrostatic interactions (using (PKG) and (DOG), (2) covalent cross-linking via photoinitiated thiol-ene click chemistry previously used by the group (using alloc-functionalized lysine), and (3) reactive handles for in situ hydrogel labeling (using azide-functionalized lysine). The authors describe the self-assembly of their mfCMPs into triple helices, which hierarchically assemble into fibrillar structures, as visualized by transmission electron microscopy. The authors confirmed the incorporation of fibrillar, fluorophore-labeled mfCMPs into their PEG-SH hydrogel system using a super-resolution imaging technique (stochastic optical reconstruction (STORM) imaging) that allows for the localization of single fluorophores. Furthermore, the authors demonstrated that incorporating mfCMPs into hydrogels altered the material's shear-stress relaxation properties, with hydrogels containing higher concentrations of mfCMPs exhibiting greater viscoelasticity and shorter stress-relaxation half-times. The authors attributed this shift in viscoelasticity to a change in the crosslinking network, from irreversible covalent linkages between polymers to reversible physical linkages between the mfCMPs, thereby enabling tunable viscoelasticity in their system. Finally, the authors performed cell culture experiments using T47D-GFP breast cancer cells

in dual-layer hydrogels of varying compositions to evaluate changes in cellular responses. The authors assessed three different hydrogel formulations: (1) a hydrogel with pendant integrin binding peptides added but no mfCMPs (referred to as elastic (E) formulation), (2) a hydrogel with pendant integrin binding peptides added and an mfCMP that formed fibrillar structures but did not include an integrin binding sequence (referred to as viscoelastic fibrillar (VF) formulation), and (3) a hydrogel with no pendant integrin binding peptides added and with fibrillar mfCMPs incorporating both RGD and FOGER integrin binding sequences (referred to as viscoelastic, bioactive fibrillar (VBF) formulation). The authors showed that cells grown on their VF and VBF hydrogels produced larger cell clusters, with the VBF group forming the largest clusters and exhibiting the highest fibronectin production. However, all three groups showed similar cell viability and proliferation, as measured by Ki-67 nuclear marker expression. The authors concluded that their synthetic, self-assembling mfCMP-hydrogel system enables tunable viscoelasticity and bioactivity and represents a step toward better modeling of diverse biological collagen environments.

This work confirms previous findings from the Kloxin Lab that mfCMPs can be incorporated into hydrogels to form fibrillar structures, yielding synthetic materials that more closely recapitulate endogenous collagen matrices. This work builds on their prior work by including super-resolution STORM imaging data, which enables visualization of the fibrillar mfCMP structures within the hydrogels. Furthermore, this work demonstrates that mfCMPs can impart concentration-dependent, tunable viscoelasticity to hydrogel systems and could enable improved modeling of the mechanical properties of endogenous collagen-rich tissues. The work is also novel for including an integrin-binding sequence embedded in the mfCMP structure. The authors claim that this sequence could increase the bioactivity of their system by enabling greater cell-cell interactions and the modeling of binding sites in both intact and degraded matrices. Yet, the authors present data only for T47D breast cancer cells that form clusters and do not show their interactions with the surrounding hydrogel matrix.

Comments

1. In Figure 2, could the authors comment on why the melt curve for the mfCMPa-G-az FOGER-containing peptide has a different shape than the mfCMP-R-az and mfCMP-a-az ones?

2. In the caption of Figure 4, the descriptions for panels a) and b) are swapped.

3. Could the authors include a comment on how the stress relaxation properties of their mf-CMP-containing hydrogels might be different while still preserving a similar equilibrium-swollen storage modulus?

4. Could the authors comment on why they chose to perform the stress-relaxation experiments exclusively with their mfCMP-a-az peptide, which lacks the FOGER and RGD integrin-binding motifs? Additionally, could the authors comment on how they expected the mechanical properties of their system to differ compared to their previously studied system (Kloxin et al., Biomater. Sci. 2020), which used a similar mfCMP lacking an integrin binding sequence and that seems to differ only in the addition of the N-terminal azide-functionalized lysine and glycine residues? Was the purpose of this experiment to evaluate the properties of their existing system further?

5. Could the authors include a comment reconciling their previous statement that "Inclusion of the alloc reactive handle was allowing the mfCMPs to serve as crosslinks within the photopolymerized network, enabling the formation of hydrogels with integrated assembled mfCMP nanostructures while maintaining control over mechanical properties." (Kloxin et al., Biomater. Sci. 2020) with the physical, reversible mfCMP linkages imparting different stress relaxation properties described in this work? Could the authors clarify whether they are referring to covalent crosslinks between the mfCMPs and the hydrogel polymer network in this previous work, but attributing the change in stress relaxation properties in this work to physical linkages between the mfCMPs?

6. For their cellular response experiments, could the authors clarify the purpose and configuration of the pendant FOGER integrin-binding peptides in the E and VF conditions? Did the authors verify that this peptide was in triple-helical conformation? Given that this sequence must be in a triple-helical conformation to be active, as the authors note in the Introduction section, I am concerned that it is not an appropriate control for comparison with the fibrillar mfCMPs if its conformation was inactive for cell recognition.

7. In Figure 5, could the authors elaborate on the rationale for testing the cell response of their system exclusively with the T47D cell line? Given that a novel aspect of the mfCMPs presented in their manuscript seems to be the inclusion of the integrin-binding motif, why did the authors choose a cell line that forms clusters and is less able to show interaction between the cell and the mfCMP–hydrogel system? Especially given that previous work by the group has used hMSC cells to show differences in cell morphology and interaction with mfCMP-containing hydrogels? Could the authors either show both cell types or provide a compelling rationale for testing only the T47D cells?

8. In Figure 5/S14, could the authors comment on why cells might be producing more fibronectin in their VBF hydrogel system? And, why is this increased fibronectin production and cell cluster volume evidence that their VBF system is a better model of the collagen-rich microenvironments of tumors?

9. In Figure S14, the authors report using an anti-collagen I antibody. Could the authors comment on differences in type I collagen production between cells cultured on their E, VF, and VBF formulations? Or, comment on why they chose not to quantify type I collagen production between the conditions and only quantify fibronectin expression?

Reviewer: 4

Comments to the Author

With pleasure, I have read the article "Synthetic Surrogates of Collagen-Rich Microenvironments: Integrating Modular Bioactive Fibrillar Structure and Tunable Viscoelasticity via Multifunctional Assembling Peptides" by Castro and coworkers. The paper is relevant timely, and well executed. There are a few places where the article could be strengthened in presentation and analysis.

Major:

1) The claim is that these materials integrate adhesion motifs non-stochastically within a hydrogel; however, the hydrogel formation and functionalization results in a fairly stochastic nature. Can this claim be sharpened? I understand the fact that the adhesion sites are positioned in the self-assembled structures, yet these are still stochastically incorporated in the microstructure of the hydrogel. Sharpening this claim will help the understanding/acceptance.

2) Can the rheology be modelled via a maxwell or similar function? I think that you do not have just changes in the t ½, but also in the onset of the relaxation and the balance between slow and fast events. In a perfectly ideal network, this can be modeled a bit, and I wonder how far off these networks are. Can this give some insight into the mechanism as well? Katashima has a nice review a few years back in Polymer Journal, though I know some good work has been done since then.

3) The results shown in Figure 5 are impressive and nicely show differences, but it looks to me like these are yet another formulation. Here the MMP degradable linkers remain constat, while Figure 4 shows that replacing MMP linkers with CMPs leads to the stress relaxation. In Figure 5 the number of covalent crosslinks is not changed, and more dynamic crosslinks are added, which also likely increases the moduli of the hydrogel. If I am missing something critical, then please try to make it more clear to the reader what the alignment of the experiments is.

4) There are some claims in the paper that are not supported by the data.

a. Page 11 line 28, “All mfCMPs showed a first melting event (Tm1) near 37 ˚C, indicating that around 50% of the triple helices remained intact at this temperature, like natural collagen I” Figure 2d does not support this claim based on the CD intensities and changes. If there is more data to support this claim, please make sure to point it out.

Minor:

1) There is a decent difference in Figure 2 on the melting behavior of the RGD vs COGEFR derivative. Any clue why? Or what this difference is?

2) I am not sure I fully understand the hydrogel formulations. It seems that the formulation for the functionalized CMPs also contains K(alloc)GWGRGDS? So two different binding sites in the hydrogel (one in the helix, one pendant)?

3) I do not see the supplier or structure of the alkyne fluorophores provided in the manuscript.

4) The mechanism of CMP fiber slipping as a mechanism for stress relaxation is a good hypothesis, but I would argue that it is hard to prove. The CMP’s could simply be disassociating and reassociating via sticky ends, no? As in the melting curves.

5) I wonder what the driving force for the cells to infiltrate into the upper hydrogel (the one experimentally being tested) are. How is it possible to have the cells only infiltrate upwards and the analysis be done only in the upper hydrogel. I could understand if the bottom was not degradable; however, in this setup it is. Both a scientific and a technical question. It is a neat setup, but curious how it works and how data analysis is done here.

6) Were any difference other than cluster size noticed in the cells?

7) The cell cluster size from day 1 to day 7 seems to follow the same trend. Is there a chance that the seeding methods or viscosity of the gel precursors themselves led to this difference as well? Meaning an indirect effect?

Editorial:

none

Author's Response to Peer Review Comments:

RESPONSE TO REVIEWERS

Reviewer: 1

Comments:
Here, the authors reported a fully synthetic polymer-peptide composite that mimics the behavior of collagen-rich extracellular matrices (ECM). The authors first designed specific, modular peptide sequences, functionalized with alkene pendant groups for use as crosslinkers in thiol-functionalized hydrogel precursors, and with azide-alkyne groups for functionalization with fluorescent probes for imaging. The stability and helical structure of the peptide sequences was characterized well with circular dichroism spectroscopy, and they displayed stable triple helix formation, similar to collagen. These peptides were incorporated into covalently crosslinked hydrogels based on a 4-arm polyethylene glycol (PEG)-thiol. Rheological measurements showed that the inclusion of peptides induced concentration-dependent viscoelastic behavior in the otherwise elastic hydrogels. The authors also studied the effect of this mechanical behavior on cell growth.

The manuscript is well-written and well-organized, and the authors have used a range of different techniques to characterize material properties. However, some unanswered questions remain, particularly about the purity of the peptides, quantification from microscopy images, and mechanical characterization.

Response: We thank the reviewer for their valuable insights and the constructive feedback.

Comments:
1. UPLC-MS traces are provided to characterize the peptide synthesis, but many of these have multiple peaks or peaks with shoulders. Is it possible to extract and report a percent purity for the synthesized peptides? Please note the retention times of the peptides and any associated side products in the MS traces (Figures S1-S4).

Response: We appreciate the reviewer's insights and suggestion. After trying slower gradients using column-heated HPLC purification shown in the example data below (gradient 1: 22 to 23.7% ACN over 30 minutes; gradient 2: 23.4 to 23.7% ACN over 30 minutes), we were unable to separate peaks and shoulders shown in Figures S1-S4. We hypothesize that these shoulders are peptide aggregates that are challenging to separate. As the different mfCMP designs are 47-70 amino acids long, there are side products associated with amino acid deletions present even within the purified samples. Nevertheless, we see consistency in properties from batch-to-batch with the synthesis and characterization workflows reported. To provide better resolution of impurities amongst the different mfCMP designs, we have updated the UPLC-MS data shown in

Figures S1c and S2c to be for mfCMPa-az injected at a higher concentration (0.5 mM from ~0.1 mM previously), consistent with the original panels for mfCMPa-G-az and mfCMPa-R-az. For all peptides, we also have added the known impurities associated with residue deletions in the captions and figures.

Example data:

Gradient 1: mfCMPa-R-az

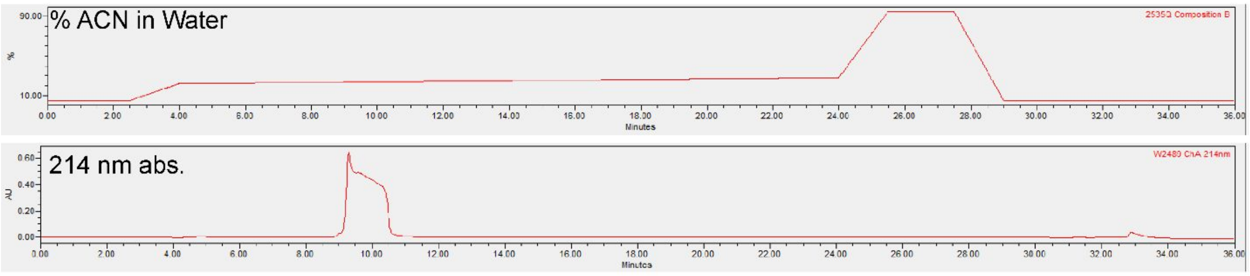

Gradient 2: mfCMPa-R-az

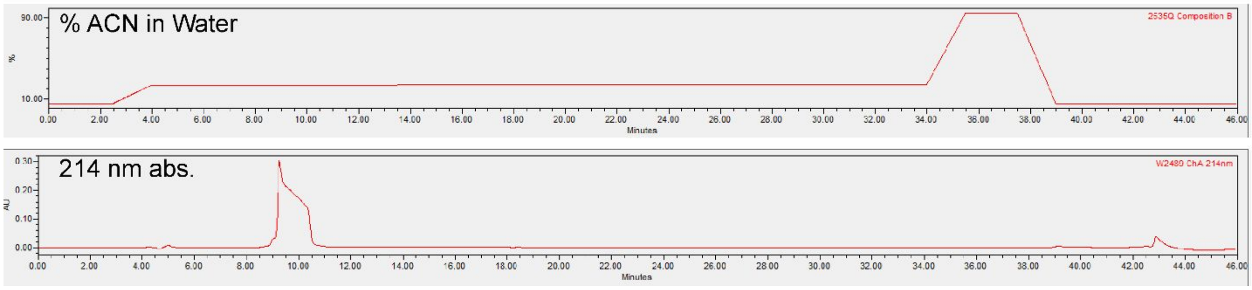

<u>Excerpts from revised text:</u>

SI page 2:

Finally, the peptide was lyophilized and stored at -80 ˚C. Ultra-performance liquid chromatography-tandem mass spectrometry (UPLC-MS, Xevo G2-S QTof; Waters, Milford, MA) was used to confirm peptide identity. Note, in addition to the prominent product peaks, some lower molecular species were observed that were unable to be separated by HPLC purification; these largely are minor residue deletions that are not observed to affect downstream properties batch-to-batch.

SI Figures:

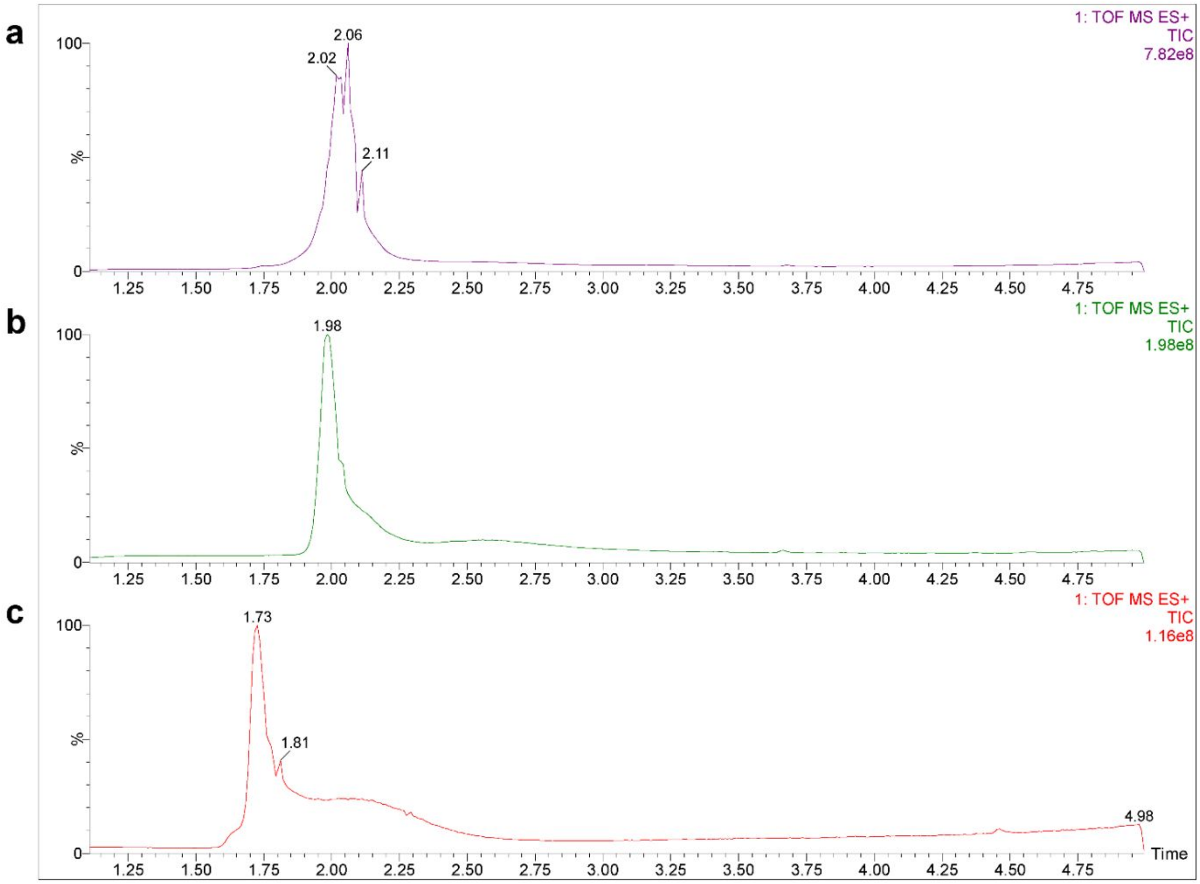

Figure S1. UPLC-MS chromatograms for purified a) mfCMP-G-az, b) mfCMP-R-az, and c) mfCMPa-az. Before UPLC-MS characterization, peptides were purified by reverse-phase HPLC (Waters XBridge C18 OBD 5 μm column), where the desired peptides with minor impurities shown here eluted from 21.9 to 23.3% acetonitrile in water (from 9.5 to 12 minutes) over a gradient of 0.7% acetonitrile per minute (18% to 30% acetonitrile).

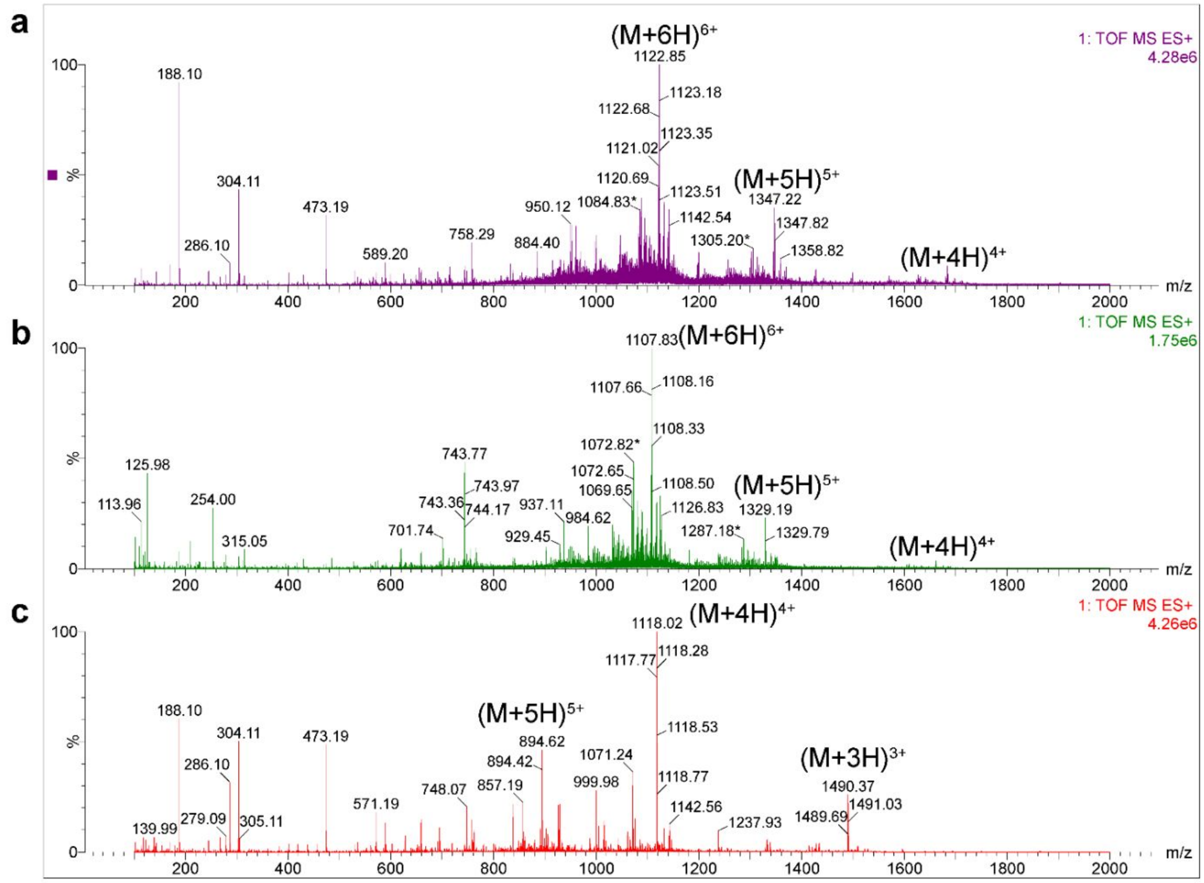

Figure S2. UPLC-MS spectra for purified a) mfCMP-G-az, b) mfCMP-R-az, and c) mfCMPa-az. Note, in addition to the prominent product peaks, some lower molecular species were observed that were unable to be separated by HPLC purification; these largely are minor residue deletions that are not observed to affect downstream properties batch-to-batch. PO deletion impurities are denoted with a (*) next to the m/z value.

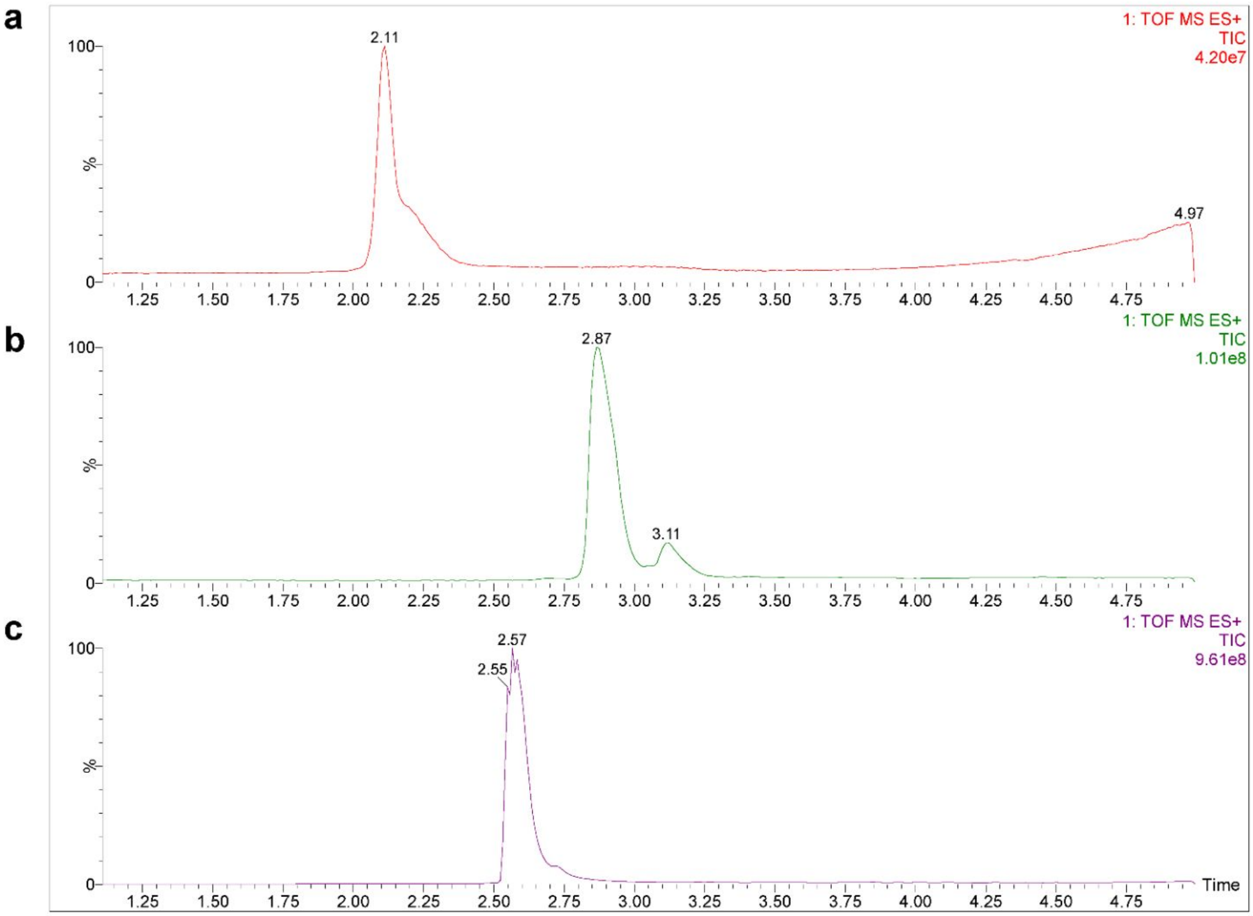

Figure S3. UPLC-MS chromatograms for purified peptides: a) K(alloc) functionalized GFOGER, b) K(alloc) functionalized RGD and c) MMP-degradable linker. Before UPLC-MS characterization, peptides were purified by reverse-phase HPLC (Waters XBridge C18 OBD 5 µm column). During HPLC, K(alloc) functionalized GFOGER with minor impurities shown here eluted from 20.4 to 23.3% acetonitrile in water (from 10 to 14 minutes) over a gradient of 0.73% acetonitrile per minute (16 to 32% acetonitrile). K(alloc) functionalized RGD with minor impurities shown here eluted from 26.5 to 28.5% acetonitrile (from 10.5 to 12.5 minutes) over a gradient of 1 % acetonitrile per minute (20 to 35% acetonitrile). MMP-degradable linker with minor impurities shown here eluted from 29.0 to 30.1% acetonitrile (10.8 to 12.5 minutes) over a gradient of 0.6% acetonitrile per minute (25 to 33% acetonitrile).

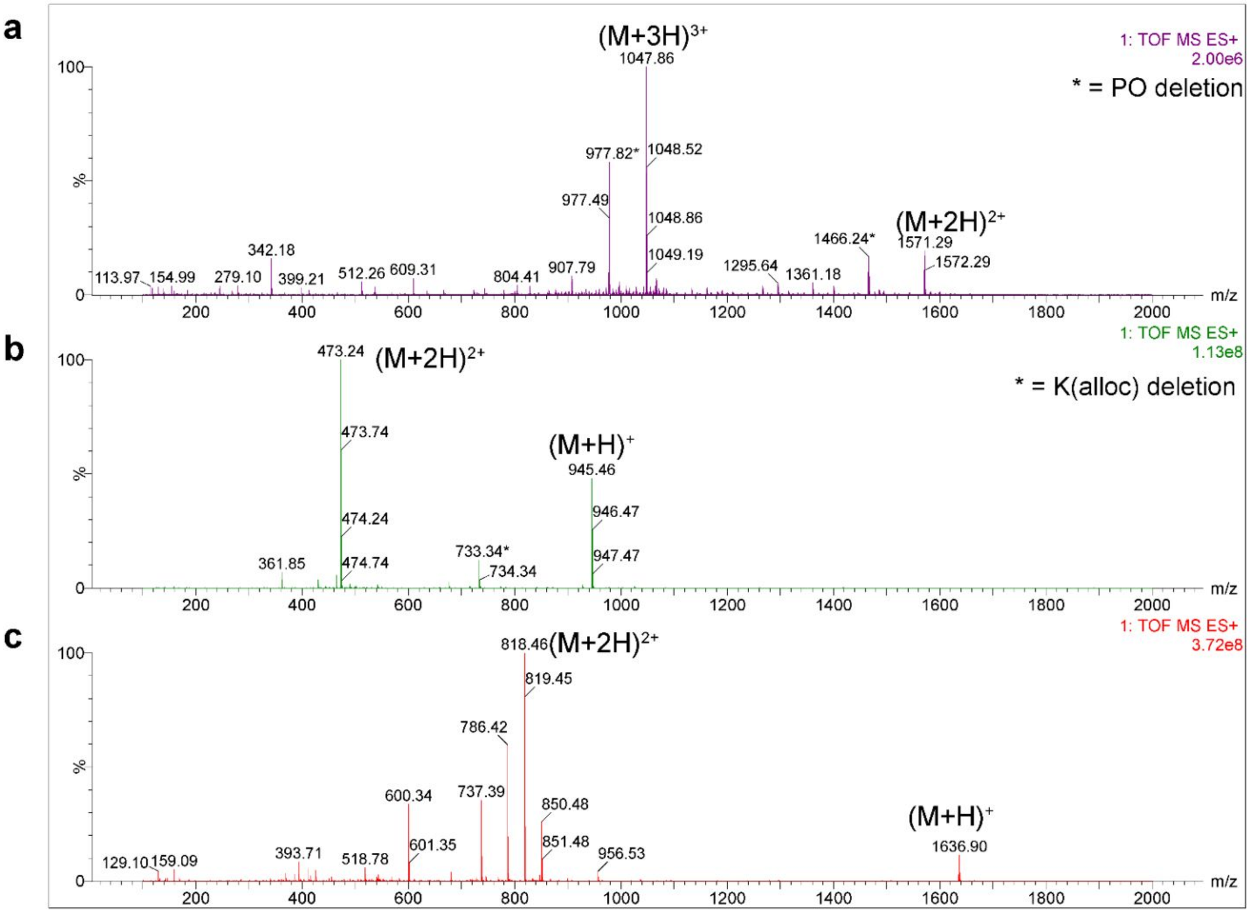

Figure S4. UPLC-MS spectra for purified peptides: a) K(alloc) functionalized GFOGER, b) K(alloc) functionalized RGD and c) MMP-degradable linker. Note, in addition to the prominent product peaks, some lower molecular species were observed that were unable to be separated by HPLC purification; these largely are minor residue deletions that are not observed to affect downstream properties batch-to-batch. Known impurities are labeled with a (*) next to the m/z value and noted on the right side of the spectrum.

2. In the CD spectroscopy data, there is a shift in the peak at 215 nm as the temperature is increased. What molecular mechanism or change in self-assembly is this shift attributed to? In addition, two of the three fibrils melt before 37 °C. How does this impact fibril presentation during cell culture?

Response: We appreciate the reviewer's comments. Revisiting the raw data, we noted that, below 215 nm, high tension voltages were above 800 V for most mfCMPs measured. As these values are above the highest acceptable value of 500 V, we attribute shifts at 215 nm to noise produced by the instrument at these high voltages. The absorbance for NaCl and KCl found in PBS, the mfCMP solvent used here, also interferes with CD data near 195-200 nm.

Guided by the reviewer's observations, we have performed further quantitative analyses of the CD data and estimated that the percentage of assembled triple helices at 37 ˚C for mfCMPa-G-az, mfCMPa-R-az, and mfCMPa-az are as follows: 79.6 ± 0.211%, 47.5 ± 0.213%, and 46.2 ± 0.172%, respectively, as described in the revised text. These values were calculated by dividing the mean residue ellipticity at 37 ˚C by the difference between the maximum and minimum mean residue ellipticity for each melting plot.

Excerpts from revised text:

Main text Page 10:

To further quantify triple helix stability, we performed CD temperature scans for each mfCMP at 225 nm with a temperature ramp from 4 ˚C to 80 ˚C, creating 'melting' curves (**Figure 2d**). For these assembling peptide sequences, these melting curves are measuring how ordered the polyproline type II helix is due to the triple helical conformation and are performed at lower concentrations (0.3 mM) than used in hydrogel formulations to avoid the formation of higher ordered structures that would scatter light and impede CD measurements (e.g., fibrils formed by sticky end interactions). The first- and second-order derivatives of these curves were then taken to identify inflection points as melting events (**Figure 2e-f**). All mfCMPs showed a first melting event (T_{m1}) near 37 ˚C, indicating that around 50% of the triple helices remained intact at this temperature, like natural collagen I.[45-46] The percentage of assembled triple helices at 37 ˚C for mfCMPa-G-az, mfCMPa-R-az, and mfCMPa-az were estimated to be 79.6 ± 0.211%, 47.5 ± 0.213%, and 46.2 ± 0.172%, respectively. These values were calculated by dividing the mean residue ellipticity at 37 ˚C by the difference between the maximum and minimum mean residue ellipticities for each melting curve. Secondary melting events (T_{m2}) are shown for all mfCMPs, possibly due to the dissociation of one end of the triple helix at a lower temperature before the other end at a higher temperature.[47] In particular, differences in the strength of intrahelical interactions between $(PKG)_n$ and $(DOG)_n$ blocks have been postulated to produce multiple melting events in other mfCMP designs.[48] Note, below 215 nm, high tension voltages were above 800 V for most mfCMPs measured; as these values are above the highest acceptable value of 600 V, we attribute shifts at 215 nm to noise produced at these high voltages.[49]

SI Page 5:

Note, the absorbance for NaCl and KCl found in PBS, the mfCMP solvent used here, can interfere with CD data near 195-200 nm.

3. Figure 2e: It looks like there are multiple melting events for the first-order derivative plot of sample "mfCMPa-G-az". Please discuss how the melting points were determined in this case, and why other minima were not considered for this assessment.

Response: We thank the reviewer their insightful observations and the opportunity to clarify and refine the approach. To better identify the melting events and describe the approach for reproducibility, we now have quantitatively identified melting events by analyzing the second order derivatives for the melting curves for all mfCMPs. The two points where the second order derivative plot crosses zero with the highest changes in magnitude are reported. Note, minor differences in the melting temperatures were observed relative to what was reported in the original manuscript. Figure S6 has been updated to include these plots, and Figure 2 has been updated to reflect the melting temperatures identified with this analysis.

Excerpts from revised text:

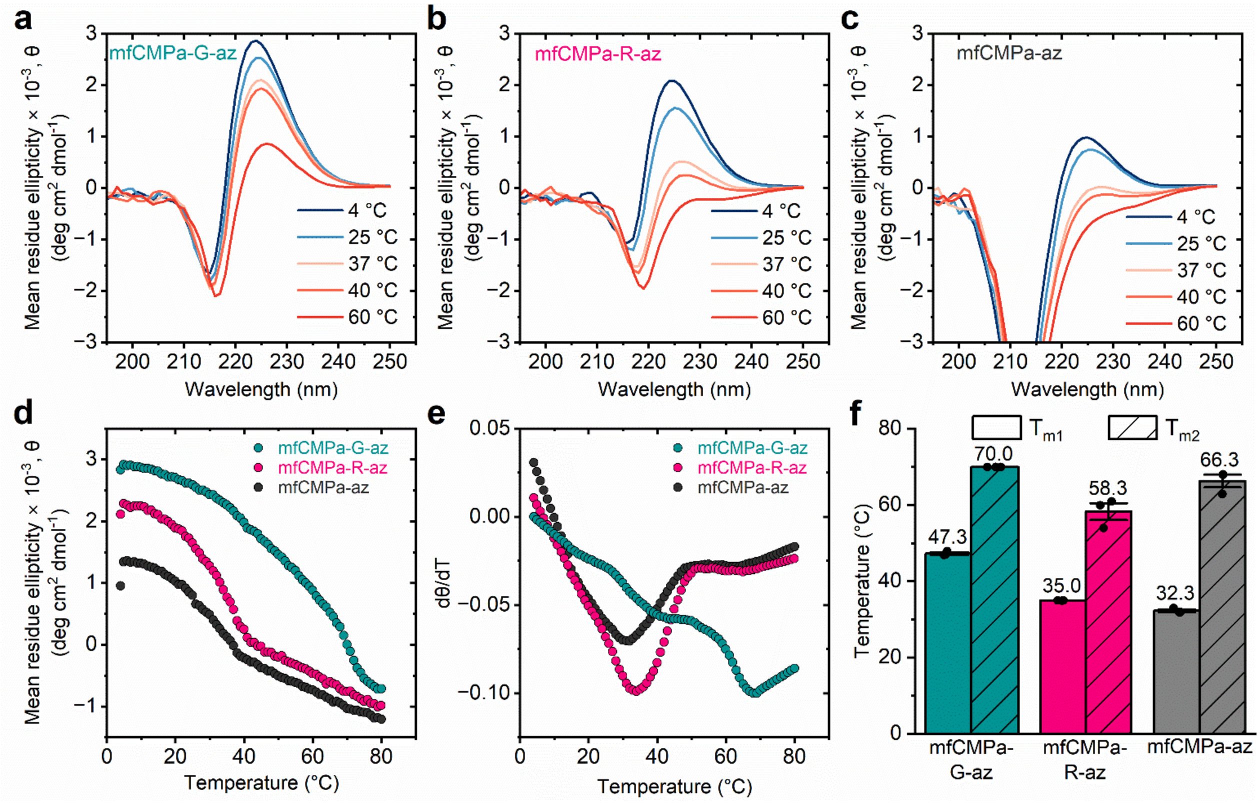

Figure 2. Triple helix characterization of mfCMPs in Dulbecco's phosphate-buffered saline by CD spectroscopy. Wavelength scans of a) mfCMPa-G-az, b) mfCMPa-R-az, and c) mfCMP-az as temperature is ramped from 4 ˚C to 80 ˚C show the characteristic polyproline type II peak (225 nm). d) Temperature scans of mfCMPs at 225 nm show nonlinear "melting" as temperature increases, indicating that triple-helical conformations are present. e) First-order derivative curves of d) show minima that describe melting events for mfCMP triple helices. f) Temperatures of melting events of mfCMPs. The two largest melting events are reported for each sequence as determined by the inflection points of (d), identified by analysis of second-order derivatives of the data in (d) (**Figure S6**). Results shown are from a representative sample of multiple trials (*n* = 3). All replicates are shown in **Figures S5-6**.

SI Page 5: Origin software (OriginLab Corporation, Northampton, MA) was used to calculate the first- and second-order derivatives of each melting curve with the Savitzky-Golay smoothing algorithm. For each peptide, the two largest changes in magnitude from positive to negative or negative to positive in the second-order derivative curves were reported as a melting event.

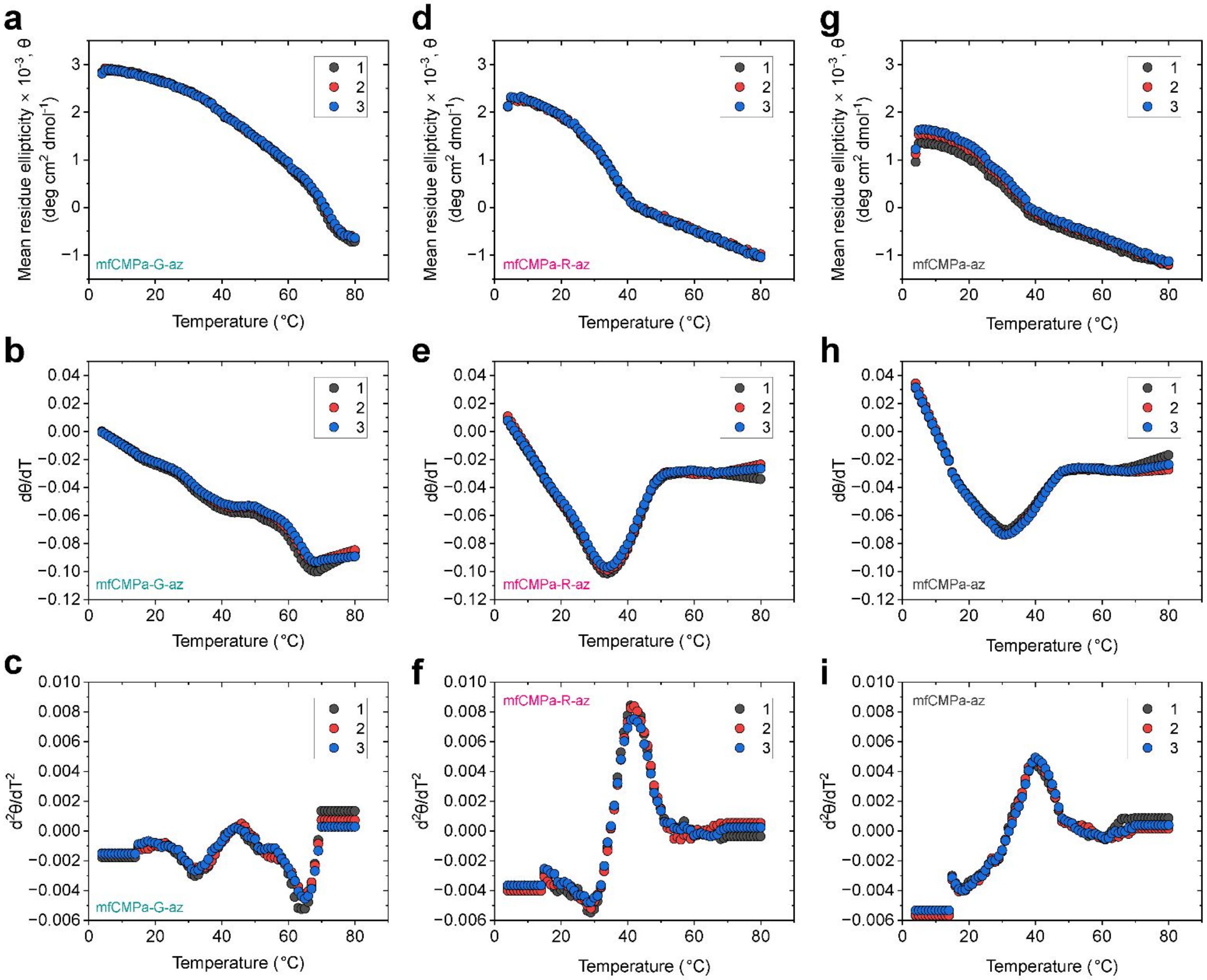

Figure S6. Temperature scans of mfCMPs at 225 nm with their first- and second-order derivatives. a-c) mfCMPa-G-az, d-f) mfCMPa-R-az, and g-i) mfCMPa-az in DPBS measured at 0.3 mM after assembly. For each, (n = 3) independent samples were measured. The two points where the second order derivative plot crosses zero with the highest changes in magnitude were identified and reported as the prominent melting temperatures (**Figure 2)**.

4. Page 13, Line 6: Please change nomenclature to "hydrogels were formed…(not polymerized)" as the process described here is a crosslinking, not a polymerization. Throughout the manuscript, it would be helpful to replace the term "photopolymerization" with "photocrosslinking" as the chemistry described here is a photoinitiated thiol-ene crosslinking – not polymerization from a monomer block.

Response: We thank the reviewer for their suggestion and have changed the text accordingly.

Excerpts from revised text:

Page 5: For probing fibrillar assembly, an innovative technique was developed to allow for super-resolution imaging, revealing hierarchical mfCMP structures within photocrosslinked polymer-peptide synthetic ECMs.

Page 12:
Using a four-arm PEG-SH as the polymer macromer (10 wt%, 20 mM thiols), hydrogels were formed using the cell-degradable linker peptide sequence KK(alloc)GGPQG↓IWGQGK(alloc)K (13 mM alloc functional handles), alloc-functionalized mfCMP (5 mM), and pendent alloc-functionalized integrin-binding peptide K(alloc)GWG**RGDS** (2 mM) by thiol-ene click chemistry after irradiation with cytocompatible doses of UV light (365 nm) and the photoinitiator lithium phenyl-2,4,6-trimethylbenzoylphosphinate (LAP; 2.2 mM).

After photocrosslinking of hydrogels directly on cover glasses, we let them equilibrium-swell in Dulbecco's phosphate-buffered saline (DPBS) for 12 hours in a non-treated 6-well plate.

Page 13: Fibrils appear randomly throughout the hydrogel in terms of distribution and orientation given the mixing approach followed by rapid photocrosslinking, which was desired for mimicking collagen-rich, loose connective tissues.

Page 19: **Figure 4**. Viscoelasticity of mfCMP-PEG hydrogels. a) Hydrogel photocrosslinking schematic and proposed mechanism of stress relaxation in mfCMP-PEG hydrogels in response to a deformation.

Page 22: Live images were captured after sufficient time for equilibrium swelling of the photocrosslinked synthetic ECMs in real-time (~8-22 hours, Videos S1-9) and then on days 1, 4, and 7 for qualitative observations of cells and cell clusters. Metabolic activity was examined on days 1, 4, and 7 using the Alamar Blue assay.

Page 25: **Figure 5.** Cell encapsulation in mfCMP-PEG hydrogels. a) Schematic of cell encapsulation where PEG-SH macromer and basic components are photocrosslinked to form a larger bottom hydrogel layer. The same components plus mfCMP and cells are then photocrosslinked on top to form the cell-laden layer.

Page 26: We have shown how these synthetic ECMs can be photocrosslinked for imaging using super-resolution microscopy to visualize their fibrillar mfCMP hierarchical structure.

Page 27: To visualize them within equilibrium swollen hydrogels, we have developed hydrogel preparation techniques to allow for STORM imaging, elucidating how hierarchical mfCMP structures appear within a photocrosslinked polymer-peptide synthetic ECM.

5. In general, the data in Figure 3 is intriguing but largely qualitative. How does the inclusion of different peptide sequences affect fibrillar characteristics? Please quantify differences between G, R, and control via image analysis. It would be helpful to quantify connectivity, fiber length, diameter, and compare across different samples.

Response: We thank the reviewer for their suggestions. Accordingly, we have quantitatively analyzed fibril length and width differences between the three different mfCMP sequences and added this analysis to a new Figure S11. We found that mfCMPa-R-az has significantly shorter lengths and narrower widths than both mfCMPa-G-az and mfCMPa-az. We hypothesize that this difference is due to the instabilities introduced by the integrin binding domain (RGDS**P**) within the mfCMPa-R-az sequence as described in the text.

Excerpts from revised text:

Page 13-14:
STORM images were used to quantify fibril lengths and widths (**Figure S11**). Notably, mfCMPa-R-az showed significantly reduced lengths and widths compared to the other mfCMPs. We hypothesize that this difference is due to the instability to its hierarchical structure introduced by the proline residue in the RGDSP domain disrupting the secondary and tertiary structures of the mfCMP-R-az as described above in the melting temperature analysis (**Figure 2**).

SI:

Page 8: *mfCMP fibril size analysis*: Length and width of mfCMP fibrils were characterized by processing STORM images in Fiji ImageJ software. First, scale was set for each image using the scale bar. Next, images were converted to 8-bit, background was removed using the despeckle function, then the lower bound of the image threshold was increased by 1-2 points until individual fibrils were visible. Fibril length and width were then determined using the Ridge Detection plugin with the following parameters: line width = 20, high contrast = 1000, low contrast = 200, extend line on, estimate width on, and slope method for overlap resolution. At least 70 fibrils were measured for each mfCMP.

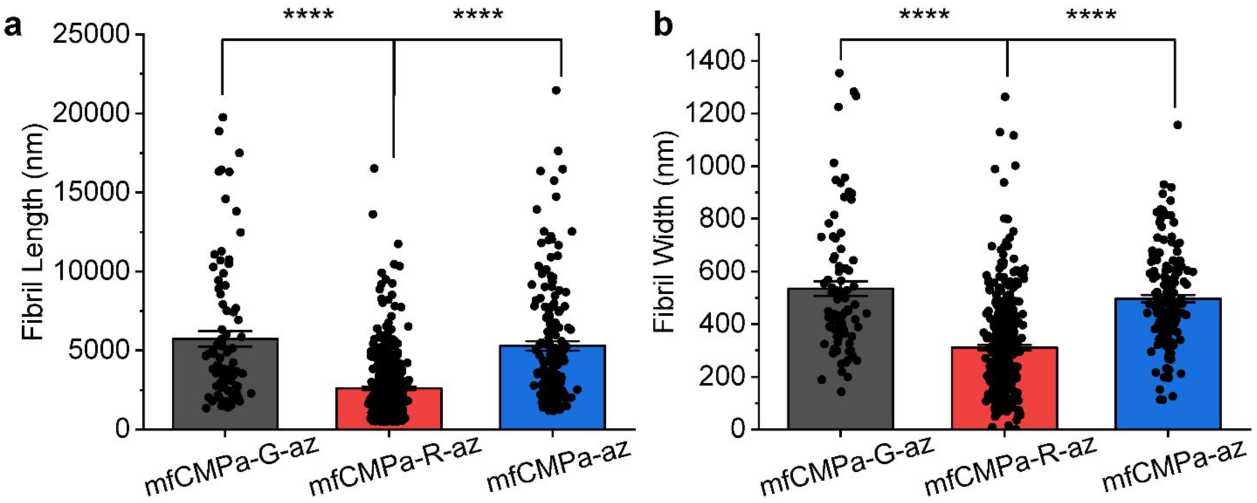

Figure S11. mfCMP fibril a) length and b) width analysis and comparison for mfCMPa-G-az, mfCMPa-R-az, and mfCMPa-az from STORM images (representative images shown in **Figure 3**), where statistical differences were observed for mfCMPa-R-az fibril lengths and widths relative to mfCMPa-G-az and mfCMPa-az (>70 fibrils measured for each mfCMP; ****$p < 0.0001$).

6. For improved clarity, please include a table of hydrogel formulations used in this study and a schematic of how the hydrogels were made. Please also specify why 5 mM concentration was used.

Response: We thank the reviewer for their suggestion and have updated the SI with hydrogel formulations in this study as new Tables S2-3. We have also added Figure S18, a schematic that explains the gel making process for cell encapsulation.

We chose a mfCMP concentration of 5 mM because previous studies that have used this concentration showed interesting cellular responses, with increased fibroblast speed and elongated morphology (Ford, et. al, J. Mat. Chem B., 2024). In this new contribution, we wished to focus on the effects of the designed materials on cell proliferation and growth, and accordingly, selected a breast cancer cell line known for spheroid formation with limited migration (luminal A, epithelial-like T47Ds). We hypothesized that the same concentration of mfCMP would affect cellular behavior with the breast cancer cells.

Excerpts from revised text:

Page 20-21:

Here, we chose to use T47D-GFP (green fluorescent protein), a luminal A breast cancer cell line transduced for constitutive expression of GFP to facilitate characterization of breast cancer cell

morphology and growth. In these studies, we wished to focus on the effects of the designed materials on cell proliferation and growth, and accordingly, selected a luminal A breast cancer cell line known for spheroid formation with limited migration,[67] where we previously have observed the importance of β1 integrin-binding by RGDS and GFOGER in cluster growth.[65] We hypothesized that the i) viscoelasticity that mfCMPs impart into the hydrogel-based synthetic matrix ii) in addition to integrin-binding sequence presentation from the mfCMPs would affect cellular processes like cluster formation, cell shape, and viability.

To form each hydrogel for these experiments, we took a dual-layer hydrogel approach (**Figure S18**) similar to previous studies,[65] now integrating the mfCMP (**Figure 5a**). Here, we chose a total mfCMP concentration of 5 mM based on our previous studies showing this concentration was effective for promoting increased fibroblast speed and elongation.[37]

SI:

Table S2. Concentrations of monomers used to form hydrogels for in situ gelation time sweeps, frequency sweeps, strain sweeps, and stress relaxation experiments.

	0 mM	5 mM	9 mM	13 mM	20 mM
PEG (mM thiol)	20	20	20	20	20
Linker Peptide (mM alloc)	18	13	9	5	0
mfCMPa-az (mM alloc)	0	5	9	13	20
Pendent RGD (mM alloc)	2	2	2	2	0

Table S3. Concentrations of monomers used to form hydrogels for stress relaxation experiments.

	E	VF	VBF
PEG (mM thiol)	20	20	20
Linker Peptide (mM alloc)	13	13	13
mfCMPa-az (mM alloc)	0	5	3
mfCMPa-G-az (mM alloc)	0	0	1
mfCMPa-R-az (mM alloc)	0	0	1
Pendent RGD (mM alloc)	1	1	0
Pendent GFOGER (mM alloc)	1	1	0

7. Page 17, line 48-49: Please specify what is meant by physical crosslinks.

Response: We appreciate the reviewer's comment and the opportunity to clarify. Physical crosslinks mean the electrostatic interactions between the mfCMP charged $(PKG)_n$ and $(DOG)_n$ blocks of adjacent triple helices and the hydrogen bonds formed between the proline and hydroxyproline residues of adjacent peptides.

Excerpts from revised text:

Page 16:

We hypothesized that hydrogels with mfCMPs would exhibit stress relaxation because of the collagen-like physical crosslinks between mfCMP peptides and fibrils (i.e., hydrogen bonding and sticky-end electrostatic interactions), allowing fibrils to slide past each other to dissipate stress induced by applied strain (**Figure 4a**). In the hydrogel, assembled mfCMPs are covalently crosslinked into the polymer network, and the mfCMPs then serve as physical crosslinks within the network owing to their physical interactions with each other via hydrogen bonding within triple helices and salt bridging within fibrils. The dynamics of these physical crosslinks have the potential to impart bioinspired, tunable viscoelasticity and provide a new complementary mechanism for modulating synthetic matrix properties.

8. Page 18, line 4: Please clarify how long the gels were stabilized before measurements were started (e.g., 105 s as mentioned in the manuscript?). Please also include these details in the methods section of the SI.

Response: Before stress relaxation experiments, a 2 second rise time was applied to the constant step strain of 15%. To clarify, this means that over that 2 second period, strain was ramped from 0 to 15%, after which measurements were immediately collected for the stress relaxation experiment that lasted for 10^5 additional seconds. The methods section has been updated with this clarification.

Excerpts from revised text:

Page 16-17:

Strain was first ramped from 0 to 15% over 2 seconds (2 second rise time to minimize noise at the start of the measurement[63]), then held constant at 15% for each sample for 10^5 seconds.

SI Page 9:

For stress relaxation measurements, 10^5 s of measurements followed a 15% strain (determined to be in the LVR by the strain sweeps). Before collecting stress relaxation measurements, the strain was first ramped from 0 to 15% over a period of 2 seconds (2 second rise time), immediately after which measurements were collected for up to 10^5 seconds.

9. Figure 4: Please enhance Figure 4a with a Chemdraw of crosslinking between the alkene groups on the peptide, cell-degradable linkers, and the PEG-SH.

Response: We thank the reviewer for their suggestion and have made the changes to Figure 4a.

Excerpts from revised text:

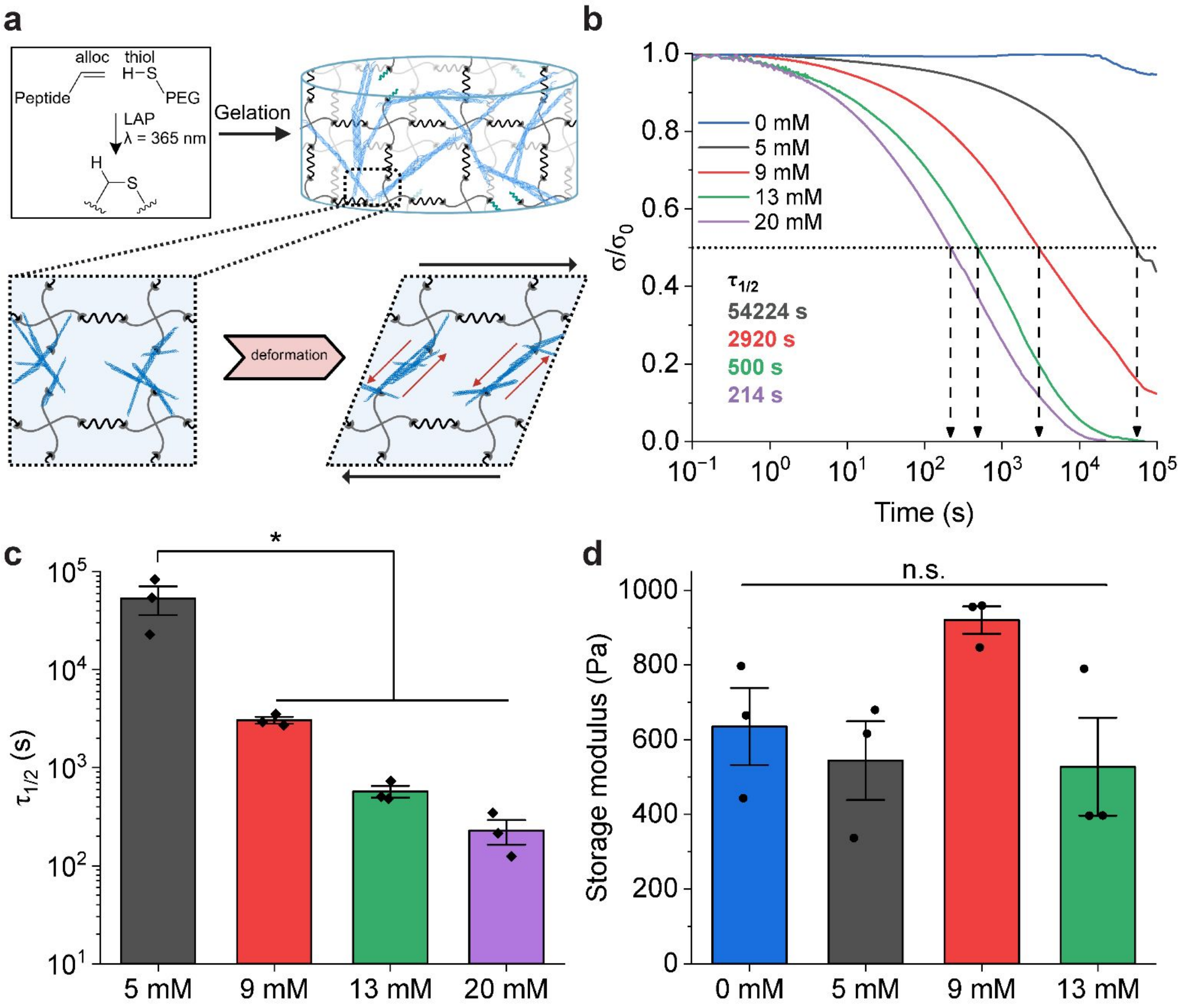

Figure 4. Viscoelasticity of mfCMP-PEG hydrogels. a) Hydrogel photocrosslinking schematic and proposed mechanism of stress relaxation in mfCMP-PEG hydrogels in response to a deformation. Physical crosslinks relax as the network is deformed and mfCMP fibrils slide past each other, dissipating mechanical energy. b) In situ stress relaxation profiles of representative hydrogels that contain increasing concentrations of mfCMPa-az. All replicates shown in **Figure**

S15. c) $\tau_{1/2}$ of hydrogels that contain increasing concentrations of mfCMPa-az. d) Equilibrium-swollen storage moduli of hydrogels containing 0 mM, 5 mM, 9 mM, and 13 mM of mfCMPa-az.

10. Figure 4 only contains measurements for the control mfCMP condition; however, Figure 5 uses mfCMPa-R-az and assumes viscoelastic behavior. It would be helpful and rigorous to support this assumption with rheological data.

Response: We have added the stress relaxation data for hydrogels containing only mfCMPa-R-az and mfCMPa-G-az as well as the formulations used in the cell experiments (E, VF, and VBF). These data are shown in new figures (Figure S19) and demonstrate all conditions with mfCMP show viscoelastic behavior.

Excerpts from revised text:

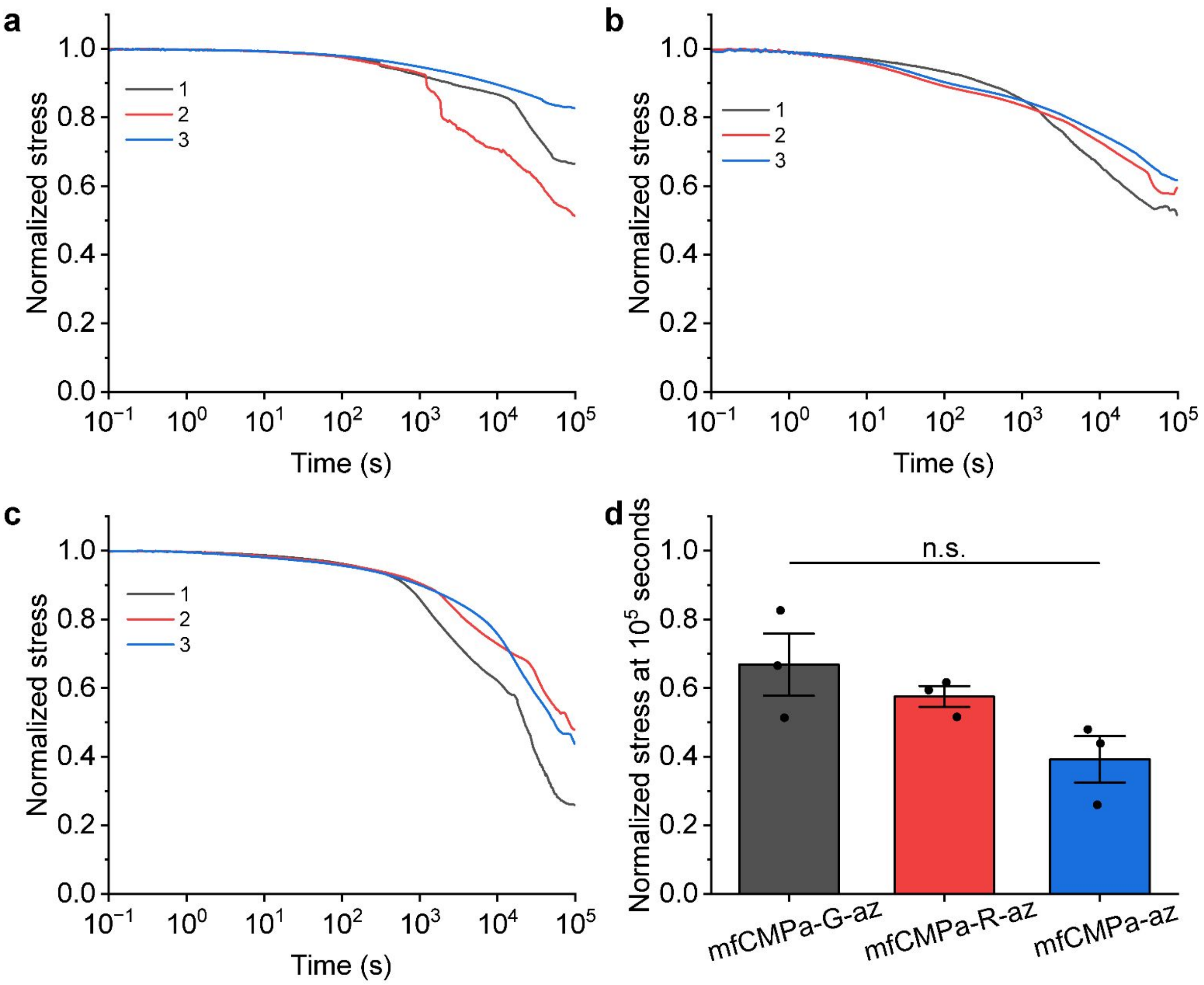

Figure S17. Stress relaxation behavior for hydrogels containing 5 mM a) mfCMPa-G-az, b) mfCMP-R-az, and c) mfCMPa-az. Stress is normalized to the maximum stress measured for each sample. d) Comparison of stress relaxation at 10^5 seconds for these compositions. Means ± standard error are shown for each condition for (n = 3) independent sample measurements. Statistical significance was determined by one-way ANOVA with Tukey's multiple comparisons test (n.s. = no statistical difference).

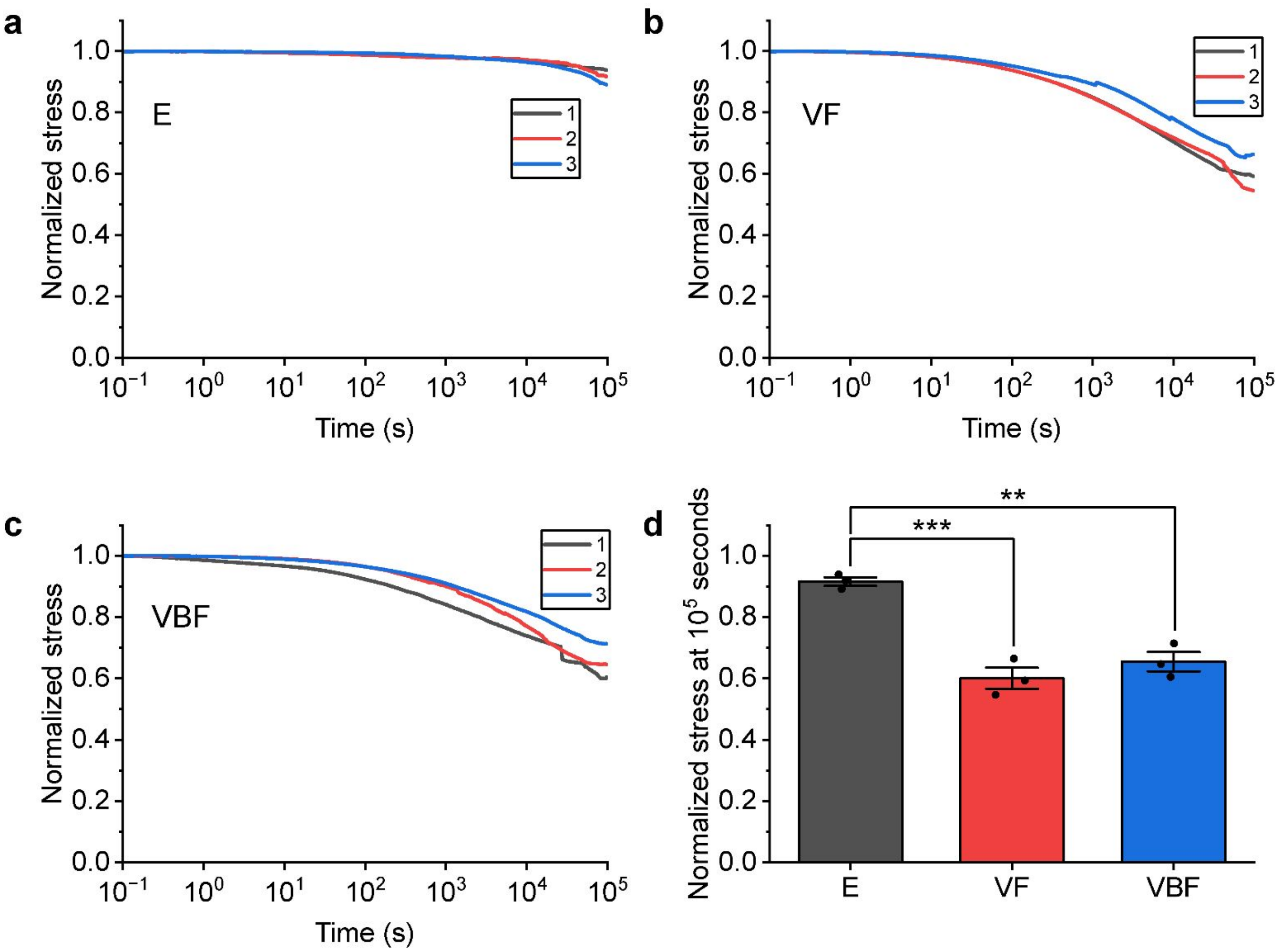

Figure S19. Stress relaxation behavior of hydrogel formulations used in cellular studies: a) E, b) VF, and c) VBF. Stress is normalized to the maximum stress measured for each sample. d) Comparison of average values for stress relaxation at 10^5 seconds for these compositions. Means ± standard error are shown for each condition for (n = 3) independent sample measurements. Statistical significance was determined by one-way ANOVA with Tukey's multiple comparisons test. Statistical significance is shown (*p < 0.05; **p < 0.01; ***p < 0.001; ****p < 0.0001).

Page 18:

Based on the viscoelastic behavior we observed in hydrogels with mfCMPa-az, we then tested the hypothesis that integrin-binding peptides could be presented from the mfCMP while still achieving similar stress relaxation profiles. Indeed, hydrogels containing 5 mM of mfCMPa-G-az and mfCMPa-R-az showed viscoelastic behavior similar to mfCMPa-az (**Figure S17**).

Page 22:

Further, each hydrogel composition (E, VF, VBF) was probed for viscoelastic behavior (**Figure S19**), where the VF and VBF formulations showed significantly greater stress relaxation than the E formulation over a period of 10^5 seconds.

11. Figure 4b: What was the fitting used for the half-lives in this figure?

Response: Relaxation half times were determined by the time at which 50% of the maximum stress was relaxed without any fitting. Based on the reviewer's feedback, we have done new analysis where we have fitted the existing stress relaxation data of hydrogels containing mfCMPa-az in Figure 4 to the generalized three-mode Maxwell model with fitted parameters (Figure S16, Table S1). There may be at least three modes of relaxation in our PEG-mfCMP hydrogels based on the results of this model fitting. We hypothesize that faster modes may be associated with the relaxation of the triple helix or fibril mfCMP assemblies in response to a deformation, and slower modes may be associated with poroelastic effect of the network, with opportunities to test this mechanistic hypothesis of the origin of the modes in future studies.

Excerpts from revised text:

Page 17-18:

These data (**Figure S15**) were then fitted (**Figure S16**) with a generalized Maxwell model with three modes (**Equation S2**), a viscoelastic model that describes three stress relaxation events in parallel.[64] The fitted parameters describing the stress, A_i, and relaxation time, τ_i, for each were determined (**Table S1**). This model fitting suggests that there are at least three modes of relaxation in our PEG-mfCMP hydrogels. We hypothesize that faster modes may be associated with the relaxation of the triple helix or fibril mfCMP assemblies in response to a deformation, and slower modes may be associated with poroelastic effect of the network,[9] with opportunities to test this mechanistic hypothesis of the origin of the modes in future studies.

SI Page 10:

Stress relaxation curves from Figures 4 and S15 were fitted to the generalized Maxwell model described by Equation 2, producing Figure S16 and Table S1:

$$\sigma(t) = \varepsilon_0 \sum_{i=1}^{n} G_i e^{-t/\tau_i} \quad (2)$$

where $\sigma(t)$ is the sheer stress as a function of t time (s) normalized to maximum stress, ε_0 is the applied step shear strain, G_i is the modulus of mode i normalized to maximum stress, and τ_i is the

relaxation time (s) of mode *i*. The following variables were defined: let A_i be equal to $\varepsilon_0 G_i$, the stress of mode *i* normalized to maximum stress, and let $i = 3$ modes. Fitting was done using the Microsoft Excel solver function. The objective was set to the sum of the squares of the measured normalized stresses for each timepoint, variables being changed were set to $A_{1\text{-}3}$ and $\tau_{1\text{-}3}$ with the constraints of positive values, and solving method was set to GRG Nonlinear. Initial guesses for $A_{1\text{-}3}$ and $\tau_{1\text{-}3}$ were set to 0.4, 0.35, 0.25, 200 s, 2000 s, and 20000 s, respectively.

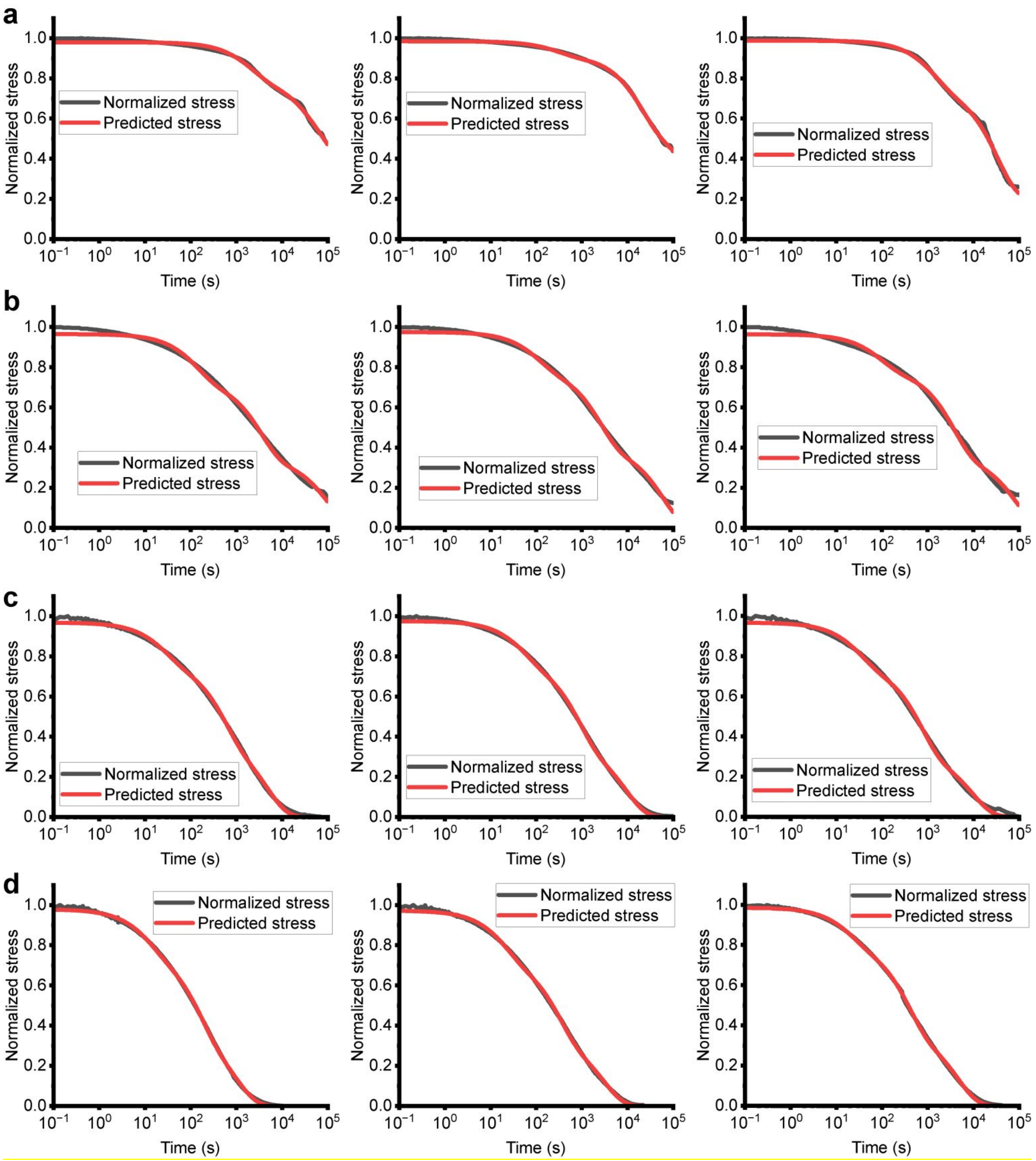

Figure S16. Stress relaxation curves containing mfCMPa-az at a) 5 mM, b) 9 mM, c) 13 mM, and d) 20 mM fitted to the generalized three mode Maxwell model.

Table S1. Fitted 3-mode Maxwell model parameters for hydrogels containing increasing concentrations of mfCMPa-az.

[mfCMPa-az] (mM)	A_1	A_2	A_3	τ_1	τ_2	τ_3
5	0.162 ± 0.048	0.390 ± 0.095	0.432 ± 0.12	1250 ± 520	31000 ± 5600	10300000 ± 980000
9	0.196 ± 0.018	0.406 ± 0.013	0.366 ± 0.017	111 ± 12	3140 ± 370	83100 ± 12000
13	0.200 ± 0.0093	0.417 ± 0.018	0.353 ± 0.023	36.8 ± 7.4	681 ± 88	6690 ± 1600
20	0.203 ± 0.017	0.421 ± 0.011	0.356 ± 0.010	17.7 ± 2.7	111 ± 64	2860 ± 1000

12. Typically, viscoelastic behavior is noted from the frequency response of the gels, and from analyzing the crossover frequency and slope of G' and G" in the frequency sweep. The frequency range used in this manuscript is quite small. Please perform the frequency sweep over an expanded range for formulations with different concentrations of peptide.

Response: We thank the reviewer for the suggestion and accordingly have collected additional data for frequency and strain sweeps at expanded ranges for hydrogels containing increasing concentrations of mfCMPa-az. These new data are shown in a revised Figure S14. While we do not observe a crossover frequency for any of the gel compositions tested, we do observe crossover points in the expanded strain sweeps.

Page 18:

For hydrogels containing mfCMPa-az, viscoelastic behavior was also seen as a crossover point between the storage and loss moduli in shear strain sweep tests, suggesting strain yielding. However, no crossover points were present in the frequency sweeps as is seen in some types of viscoelastic materials.[65]

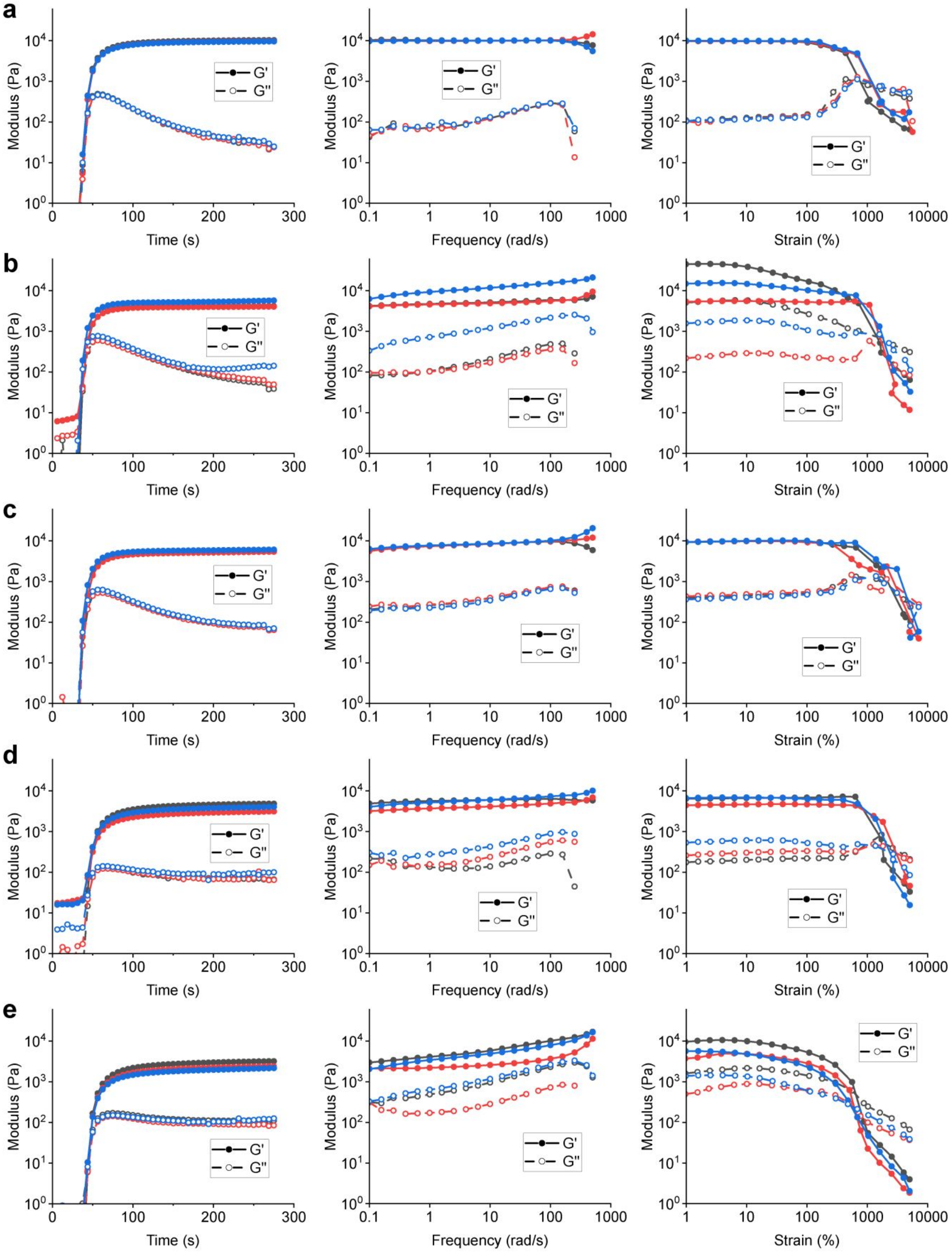

Figure S14. In situ rheology of mfCMP-PEG hydrogels with increasing concentrations of mfCMPa-az: a) 0 mM, b) 5 mM, c) 9 mM, d) 13 mM, and e) 20 mM. Gelation time sweeps, frequency sweeps, and strain sweeps were performed for (n = 3) hydrogels per condition.

Excerpts from revised text:

13. Please include STORM imaging for the 20 mM gels. Would you expect to see a much more interconnected network here due to its stress relaxation behavior?

Response: We appreciate the reviewer's question and agreed with the expected behavior. To probe this, we have performed new experiments and added new STORM images of hydrogels containing higher concentrations of mfCMPs (13 mM), demonstrating an increased density of fibrils and interconnected mfCMP network relative to a lower concentration of mfCMPs for which STORM images were shown in the original manuscript (5 mM, Figure 3). We were not able to image 20 mM mfCMP hydrogels owing to their loss of integrity with equilibrium swelling and sample processing in preparation for STORM imaging.

Excerpts from revised text:

Page 14:

Hydrogels with higher (13 mM) concentrations of mfCMP were also imaged using STORM (**Figure S13**), showing a higher density of fibrils for each mfCMP. Note, 20 mM mfCMP hydrogels could not be imaged using STORM owing to their loss of integrity with equilibrium swelling and sample processing in preparation for STORM imaging.

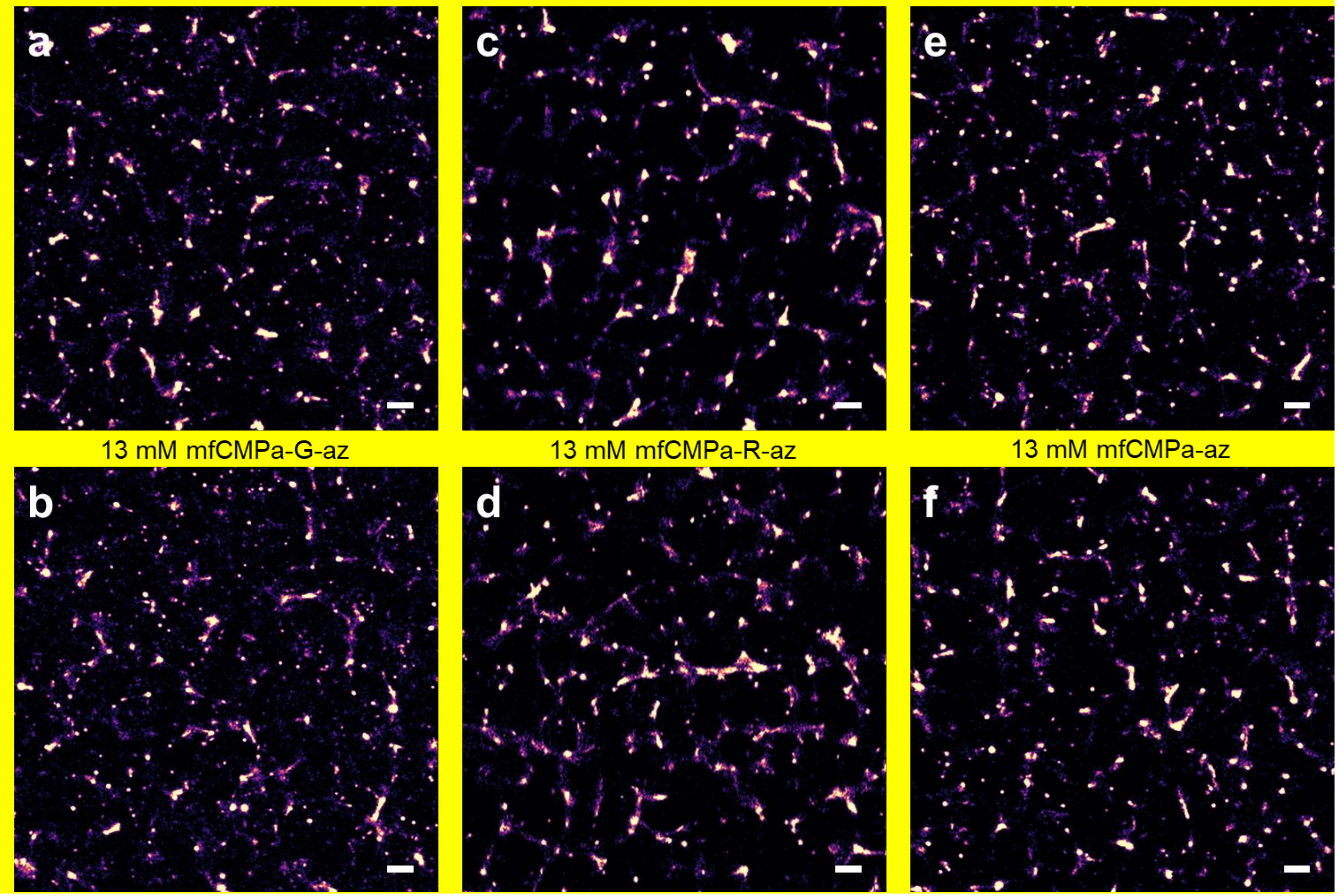

Figure S13. STORM imaging of PEG hydrogel containing mfCMPs at 13 mM. a-b) mfCMPa-G-az, c-d) mfCMPa-R-az, and e-f). mfCMPa-az. Scale bar = 500 nm.

14. Please include stress relaxation data for the viscoelastic, bioactive hydrogels shown in Figure 5. Please include a table of the gels formulated for this study for clarity.

Response: We appreciate this suggestion and have added stress relaxation data for the compositions used in Figure 5, as well as added tables describing their compositions for clarity in the SI (Tables S3).

Page 22:

Further, each hydrogel composition (E, VF, VBF) was probed for viscoelastic behavior (**Figure S19**), where the VF and VBF formulations showed significantly greater stress relaxation than the E formulation over a period of 10^5 seconds.

Table S3. Concentrations of monomers used to form hydrogels for stress relaxation experiments.

	E	VF	VBF
PEG (mM thiol)	20	20	20
Linker Peptide (mM alloc)	13	13	13
mfCMPa-az (mM alloc)	0	5	3
mfCMPa-G-az (mM alloc)	0	0	1
mfCMPa-R-az (mM alloc)	0	0	1
Pendent RGD (mM alloc)	1	1	0
Pendent GFOGER (mM alloc)	1	1	0

Excerpts from revised text:

Miscellaneous:

1. Purification of peptides: On page 8 line 27 of the main manuscript it says HPLC was used for purification, but in the SI figures it says UPLC. Which type of liquid chromatography was used? Please, keep it consistent.

Response: HPLC was used for purification, and UPLC-MS was used for characterization shown in Figures S1-4.

Excerpts from revised text:

Page 7:

These designs were rapidly synthesized by microwave-assisted solid phase peptide synthesis (SPPS), purified by heated reverse phase high performance liquid chromatography (HPLC) followed by dialysis, and the sequence identity verified by ultra-performance liquid chromatography-tandem mass spectrometry (UPLC-MS) (**Figures S1-2**).

SI Page 2:

Ultra-performance liquid chromatography-tandem mass spectrometry (UPLC-MS, Xevo G2-S QTof; Waters, Milford, MA) was used to confirm peptide identity.

2. Could you add references for the statement of page 10 line 36? "Wavelength scans show a characteristic polyproline type II peak at 225 nm associated with a collagen-like triple helix…"

Response: We thank the reviewer for their suggestion and have added references to support this statement.

Excerpts from revised text:

Page 7:

Wavelength scans show a characteristic polyproline type II peak at 225 nm associated with a collagen-like triple helix[43] for both mfCMPa-G-az and mfCMPa-R-az, as well as the mfCMPa-az control (**Figure 2a-c**).

3. Figure S7: Please show full NMR spectrum from 0 to 12 ppm. Please do not truncate relevant polymer peaks (i.e., please zoom out until the peaks are not cropped from the top). Please integrate and label all peaks of relevance.

Response: We thank the reviewer for their suggestion. We have made changes to the figure accordingly.

Excerpts from revised text:

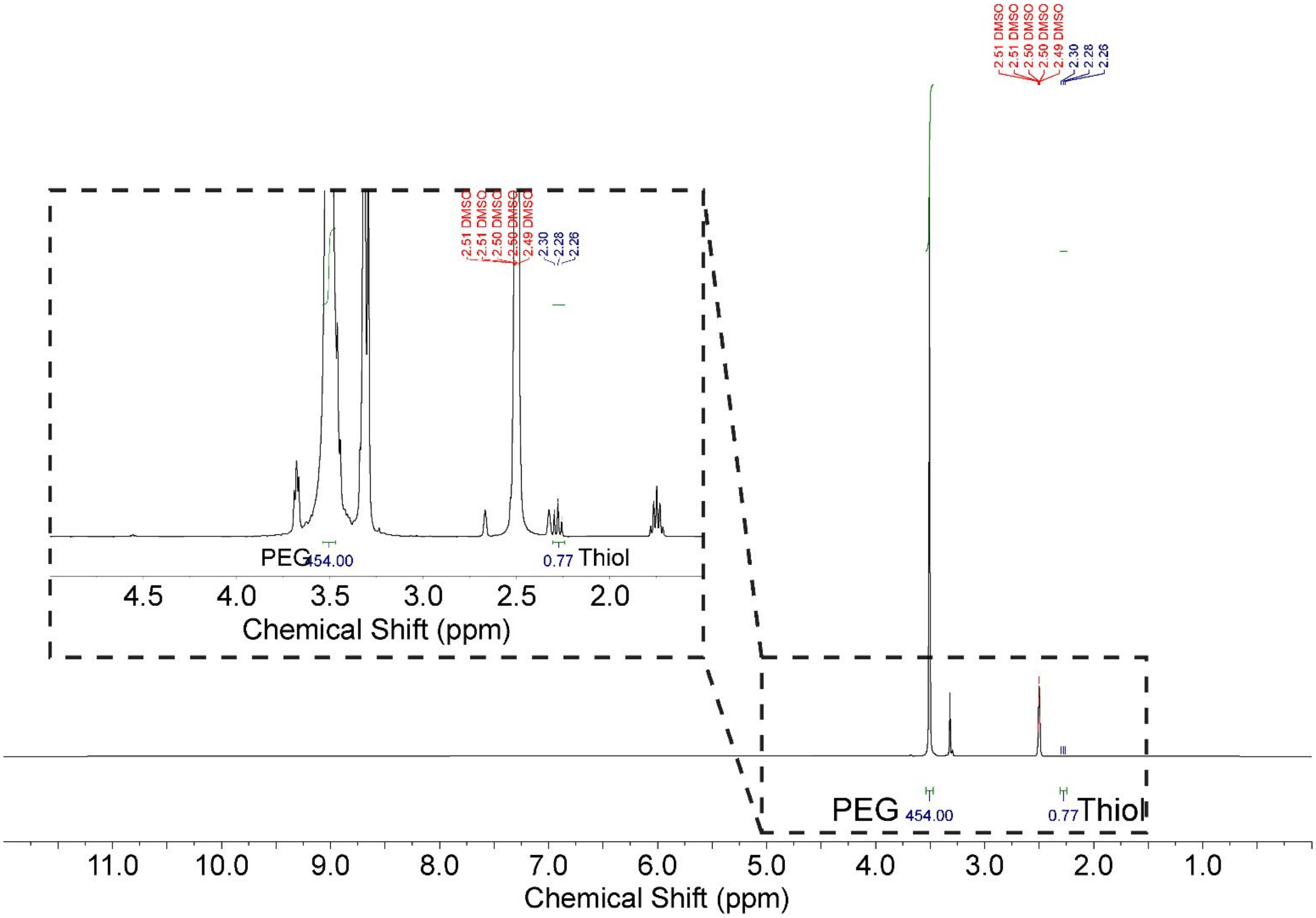

Figure S8. ^{1}H NMR spectrum for synthesized PEG-SH.

Reviewer: 2

Comments:
The authors have developed a fully synthetic materials system that captures much of the complexity of the native extracellular matrix: fibrillar architecture, viscoelastic mechanical properties, presentation of relevant bioactive signals (from the fibrillar structures), and proteolytic remodelability. The primary novelty derives from the use of the self-assembled fibrillar structures to (1) impart modes of viscous dissipation in the networks and (2) present relevant cell-adhesive motifs. These materials thus capture two key facets of natural ECM proteins like collagen that are not typically found in engineered ECM materials. The authors demonstrate using super resolution microscopy that the fibers maintain their structure after assembly into the composite hydrogels and exhibit diameters similar to native collagen. Finally, the authors demonstrate biological relevance for their engineered system using a cancer cell spheroid growth assay.

Response: We thank the reviewer for their thoughtful review and constructive feedback.

Major comments:

1. A key advantage of the system is the ability to decouple cell adhesive ligand presentation from microstructure and mechanics in the system. From the STORM imaging, are the networks of the three different self-assembling peptides statistically similar in terms of their structural properties (e.g., fiber density, orientation, etc.)?

Response: We thank the reviewer for their inquiry. Accordingly, we have quantitatively analyzed fibril length and width differences between the three different mfCMP sequences and added this analysis to a new Figure S11. We found that mfCMPa-R-az has significantly shorter lengths and widths than both mfCMPa-G-az and mfCMPa-az. Our analyses did not reveal any directional orientation, which is consistent with our expectations for the stochastically distributed fibrils. We hypothesize that this difference is due to the instabilities introduced by the integrin binding domain (RGDS**P**) within the mfCMPa-R-az sequence as described in the text.

Excerpts from revised text:

Page 13-14:
STORM images were used to quantify fibril lengths and widths (**Figure S11**). Notably, mfCMPa-R-az showed significantly reduced lengths and widths compared to the other mfCMPs. We hypothesize that this difference is due to the instability to its hierarchical structure introduced by the proline residue in the RGDSP domain disrupting the secondary and tertiary structures of the mfCMP-R-az as described above in the melting temperature analysis (**Figure 2**).

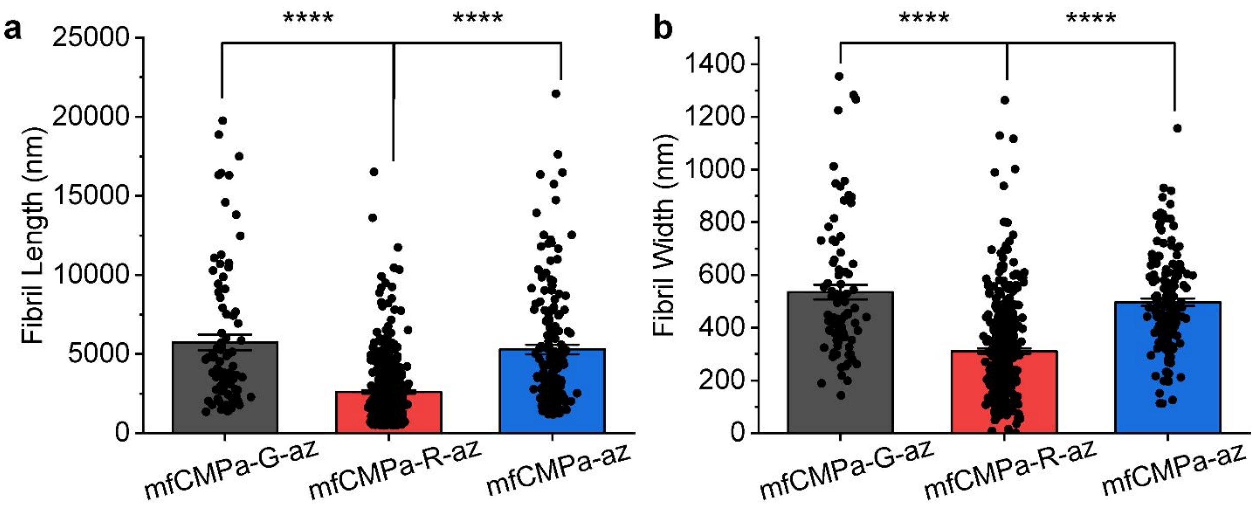

Figure S11. mfCMP fibril a) length and b) width analysis and comparison for mfCMPa-G-az, mfCMPa-R-az, and mfCMPa-az from STORM images (representative images shown in **Figure 3**), where statistical differences were observed for mfCMPa-R-az fibril lengths and widths relative to mfCMPa-G-az and mfCMPa-az (>70 fibrils measured for each mfCMP; ****$p < 0.0001$).

2. What is the cause of the larger spheroid volume in the bioactive CMP containing materials? Is it due to increased proliferation, or increased migration that allows the cells to cluster? Tracking the cells at day 1 vs. 4 vs. 7 using the existing data could provide insight. As the Ki67 levels at day 7 are not that different, if it is a proliferation effect, perhaps it is more pronounced at earlier time points. A third possibility is that the cells themselves take up more volume in materials that are more permissive (i.e., viscoelastic). Are the numbers of cells per field of view similar?

Response: We thank the reviewer for their insights and suggestions. After analyzing the cell counts per field from existing images, we have concluded that increased proliferation allows the cells to form growing clusters. VBF and VF conditions show statistically higher counts per field compared to E (Figure 5f), consistent with trends observed in Ki-67. We did not observe differences in cell volume within the clusters (Figure S25). Additionally, we have included videos of these compositions with encapsulated T47Ds from day 0, 8 hours after encapsulation, for qualitative observations of morphology and motility over 14 hours in the SI (Videos S1-9). These live videos demonstrate little migration for this epithelial-like luminal A breast cell type, which is expected for these spheroid-forming, weakly-metastatic cells.

Excerpts from revised text:

Page 23-24:

Cells in the viscoelastic hydrogel formulations trended toward a greater percentage of Ki-67 positive (%Ki-67^{+ve}) cells, although no statistical differences were observed. Importantly, consistent with this trend, we observed that the VF and VBF conditions both had significantly higher numbers of cells per field (**Figure 5f**), suggesting that cluster volume arises from increased proliferation in the viscoelastic hydrogel formulations with mfCMP. Further, we did not observe differences in cell volume (e.g., cells taking up more space owing to synthetic matrix viscoelasticity[5]) (**Figure S25**) or motility (e.g., cells migrating to form larger clusters[37, 73]) (**Videos S1-9**).

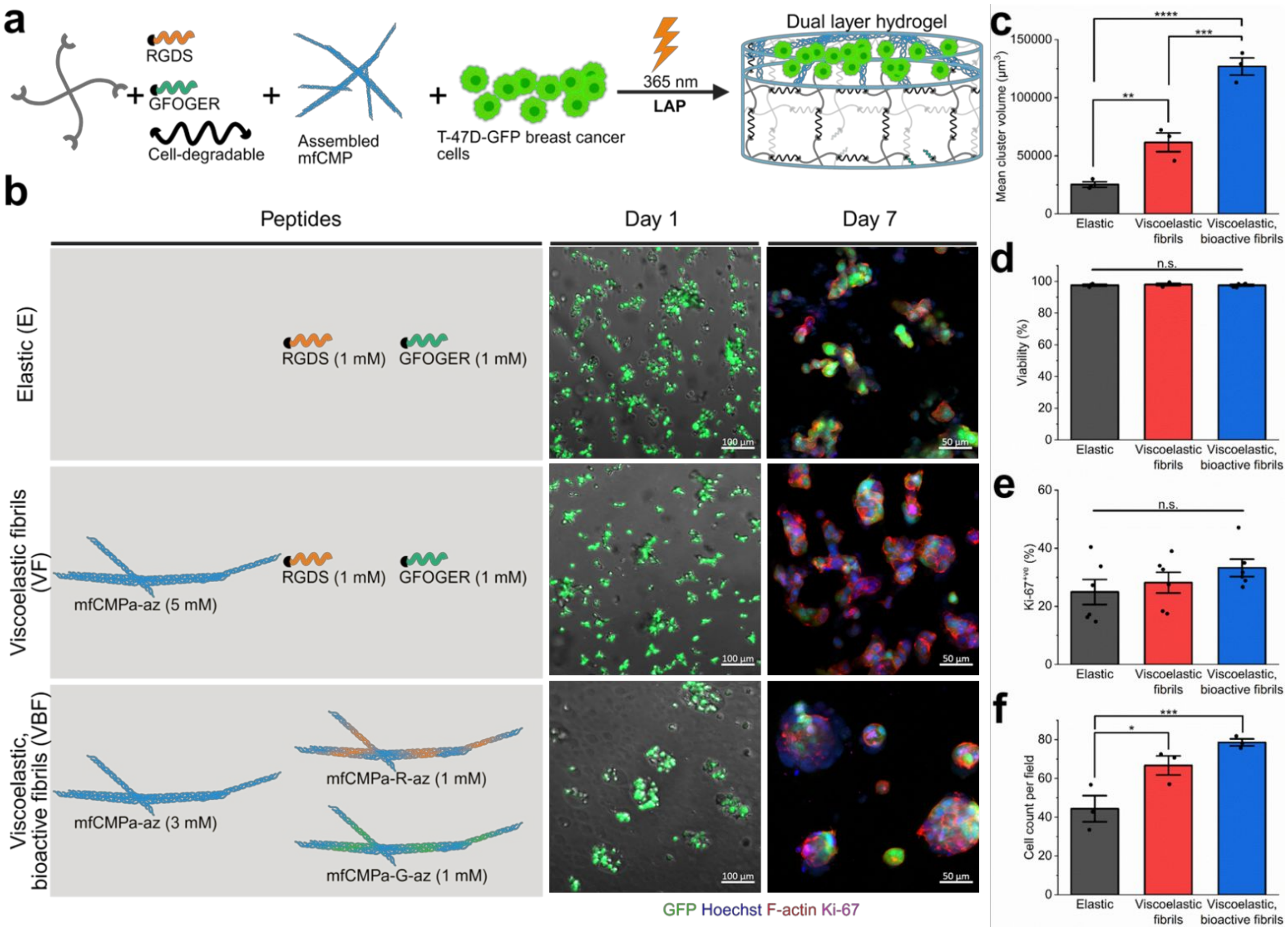

Figure 5. Cell encapsulation in mfCMP-PEG hydrogels. a) Schematic of cell encapsulation where PEG-SH macromer and basic components are photocrosslinked to form a larger bottom hydrogel layer. The same components plus mfCMP and cells are then photocrosslinked on top to form the cell-laden layer. b) Hydrogel compositions include i) an elastic formulation with pendent bioactive

peptides; ii) a viscoelastic composition with added mfCMP; and iii) viscoelastic, bioactive fibrillar composition where the pendent integrin binding peptides are replaced by bioactive mfCMPs (total mfCMP concentration 5 mM). Representative images are shown on days 1 (live imaging) and 7 (fixed immunostaining). c) Mean cluster volume of cell objects imaged in mfCMP-PEG hydrogels based on F-actin cytoskeleton staining on day 7. d) Viability of cells after 7 days based on Hoechst (nuclear) and ethidium homodimer (dead cells) staining. e) Percentage of Ki-67 positive cells based on staining on day 7. f) Number of cells per field of view based counting of Hoechst positive cells in imaged, fixed immunostained gels. Means ± standard error are shown for each condition for ($n = 3$) independent sample measurements. Statistical significance was determined by one-way ANOVA with Tukey's multiple comparisons test. Statistical significance is shown (*$p < 0.05$; **$p < 0.01$; ***$p < 0.001$; ****$p < 0.0001$).

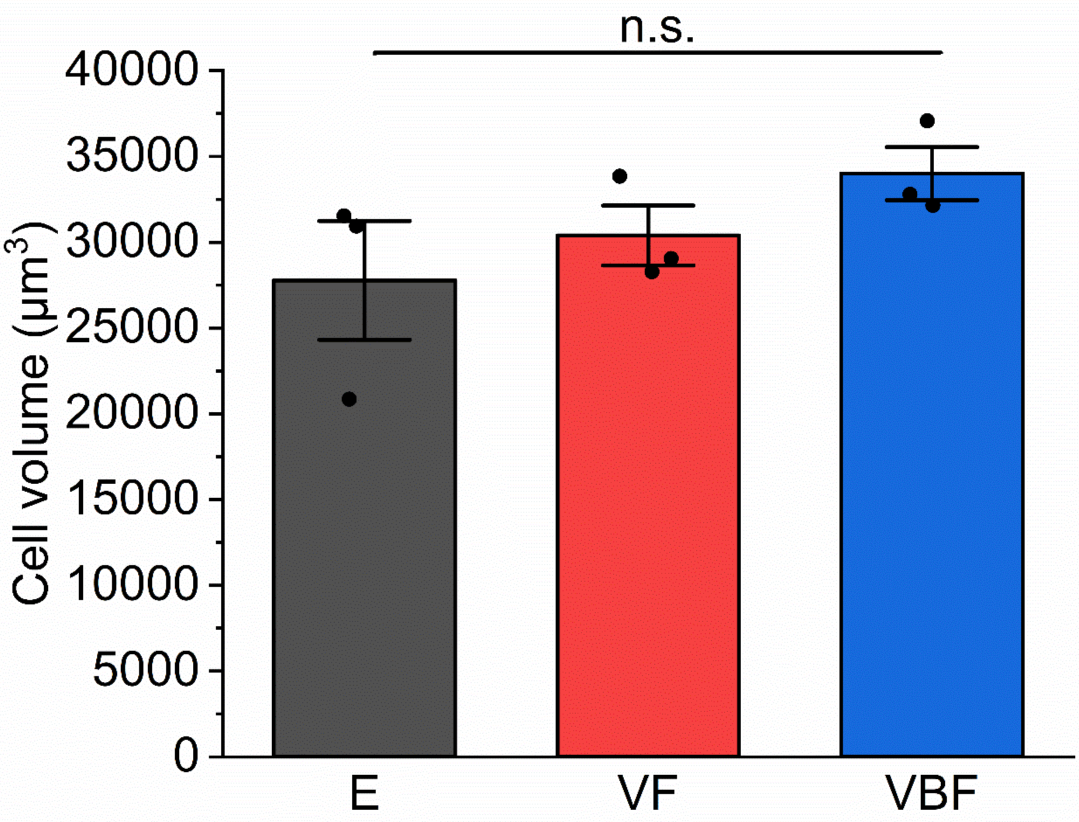

Figure S25. Comparison of mean cell volume for T47Ds in mfCMP-PEG hydrogels (conditions E, VF, VBF) on day 7 based on F-actin fluorescence. Means ± standard error are shown for each condition for (n = 3) independent averaged sample measurements. Statistical significance was determined by one-way ANOVA with Tukey's multiple comparisons test. Statistical significance is shown (*p < 0.05; **p < 0.01; ***p < 0.001; ****p < 0.0001).

3. Potentially related to comment 2, are the materials viscoplastic? That is, are the physical crosslinks from the fibrils in sufficiently high concentration to allow physical rearrangement in the networks without proteolysis? This could be one mechanism by which the cells make space to permit larger spheroid formation.

Response: We appreciate the reviewer's insights. We have collected additional data for frequency and strain sweeps at expanded ranges for hydrogels containing increasing concentrations of mfCMPa-az. These new data are shown in a revised Figure S14. In these new studies, we observe strain yielding behavior, suggesting that these mfCMP materials are

viscoplastic, which may contribute to large spheroid formation based on the literature (Chaudhuri, et. al., Nature, 2020).

Excerpts from revised text:

Page 18:

For hydrogels containing mfCMPa-az, viscoelastic behavior was also seen as a crossover point between the storage and loss moduli in shear strain sweep tests, suggesting strain yielding. However, no crossover points were present in the frequency sweeps as is seen in some types of viscoelastic materials.[65]

Page 24:

Note, we observed strain yielding behavior for mfCMP synthetic ECMs (**Figure S14**) suggesting viscoplasticity, which may contribute to large spheroid formation based on the literature.[9]

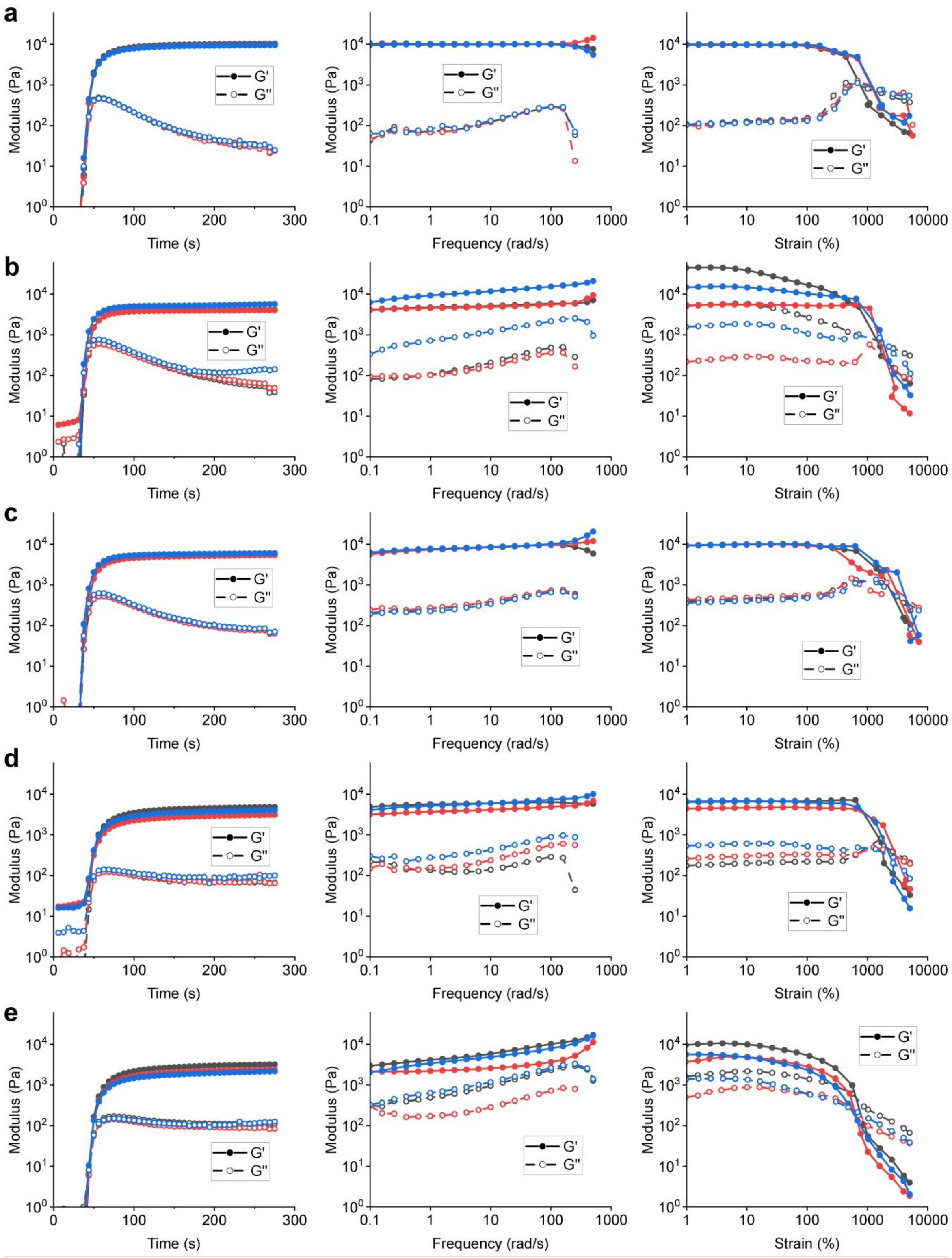

Figure S14. In situ rheology of mfCMP-PEG hydrogels with increasing concentrations of mfCMPa-az: a) 0 mM, b) 5 mM, c) 9 mM, d) 13 mM, and e) 20 mM. Gelation time sweeps, frequency sweeps, and strain sweeps were performed for (n = 3) hydrogels per condition.

4. Do the authors have a hypothesis as to why the gels presenting the adhesive sequences from the fibers, as opposed to pendant from the PEG network, yield the largest spheroids? This result highlights a key feature of their system, which is that adhesive sequences can be presented in more native-like conformations from the fibrils.

Response: We thank the reviewer for their insights and suggestion. We hypothesize that the adhesive sequences being presented in more native-like conformation from the fibrils, rather than as a pendant group attached to amorphous PEG, leads to difference in β1-integrin engagement. Based on the reviewers' comments, we have performed new immunostaining studies and do observe differences in β1 integrin organization (Figure S23). In the VBF condition, we observed small puncta-like fluorescent regions suggesting integrin clustering, which is reported more broadly as a cellular response to collagen I and supporting the relevance of the integrin-binding mfCMPs (Mana, et. al., Life Sci. All., 2022.; Ostrowska-Podhorodecka. et. al., J. of Cell Sci., 2021).

Excerpts from revised text:

Page 23:

Integrin β1 was also stained in all conditions (**Figure S23**). While β1 integrin is observed in all conditions, small puncta-like fluorescent regions were observed the in VBF condition, suggesting integrin clustering[71] which has been reported more broadly as a cellular response to collagen I.[72] β1-integrin clustering also has been reported to promote fibronectin deposition, suggesting a potential mechanism by which increased fibronectin expression was observed in VBF. Overall, these observations support the relevance of the integrin-binding mfCMPs to capture aspects of the functions of collagen I with a more native-like conformation for presentation of the integrin-binding site from the fibrils, rather than as a pendant group from amorphous PEG, leading to difference in β1-integrin engagement and cellular responses.

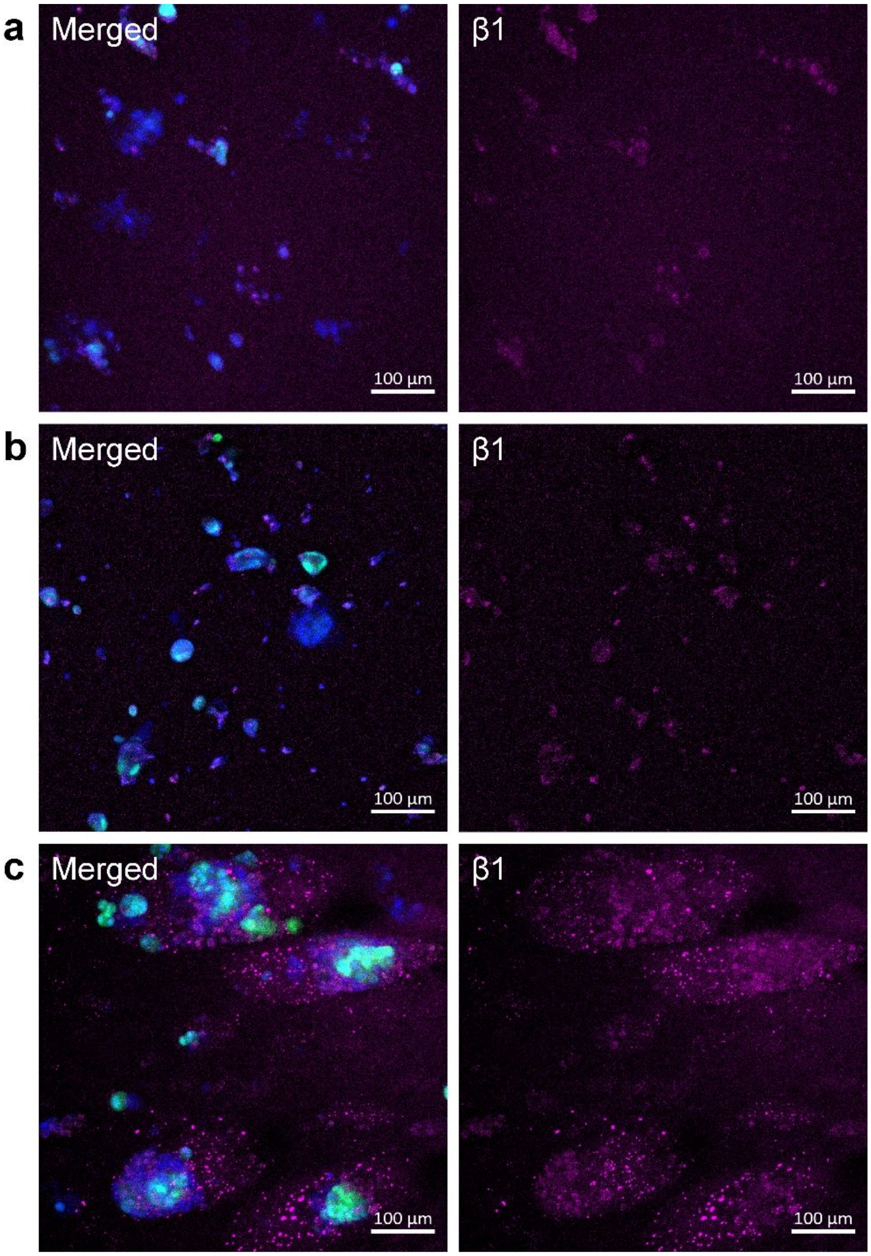

Figure S23. β1 integrin staining of fixed hydrogels for conditions a) E, b) VF, and c) VBF. Fixed samples and stained (nuclei, blue; GFP, green; β1 integrin, magenta; scale bars = 100 µm). We hypothesize that the fluorescent puncta observed for β1 integrin in VBF condition are clustered integrins in response to the fibrillar, bioactive mfCMPs. Note, α2 integrin, which is associated with α2β1 binding to collagen I, is not internalized intracellularly when integrin turnover occurs,[9] which we hypothesize leads to some retention of integrins surrounding some cell clusters.

Minor comments:

1. On page 18, the mean t1/2 for the 20 mM mfCMP gels is listed as 125 s, but this does not seem to agree with the graphs in figure 4b,c.

Response: We thank the reviewer for their feedback and have corrected this error in this figure.

Excerpts from revised text:

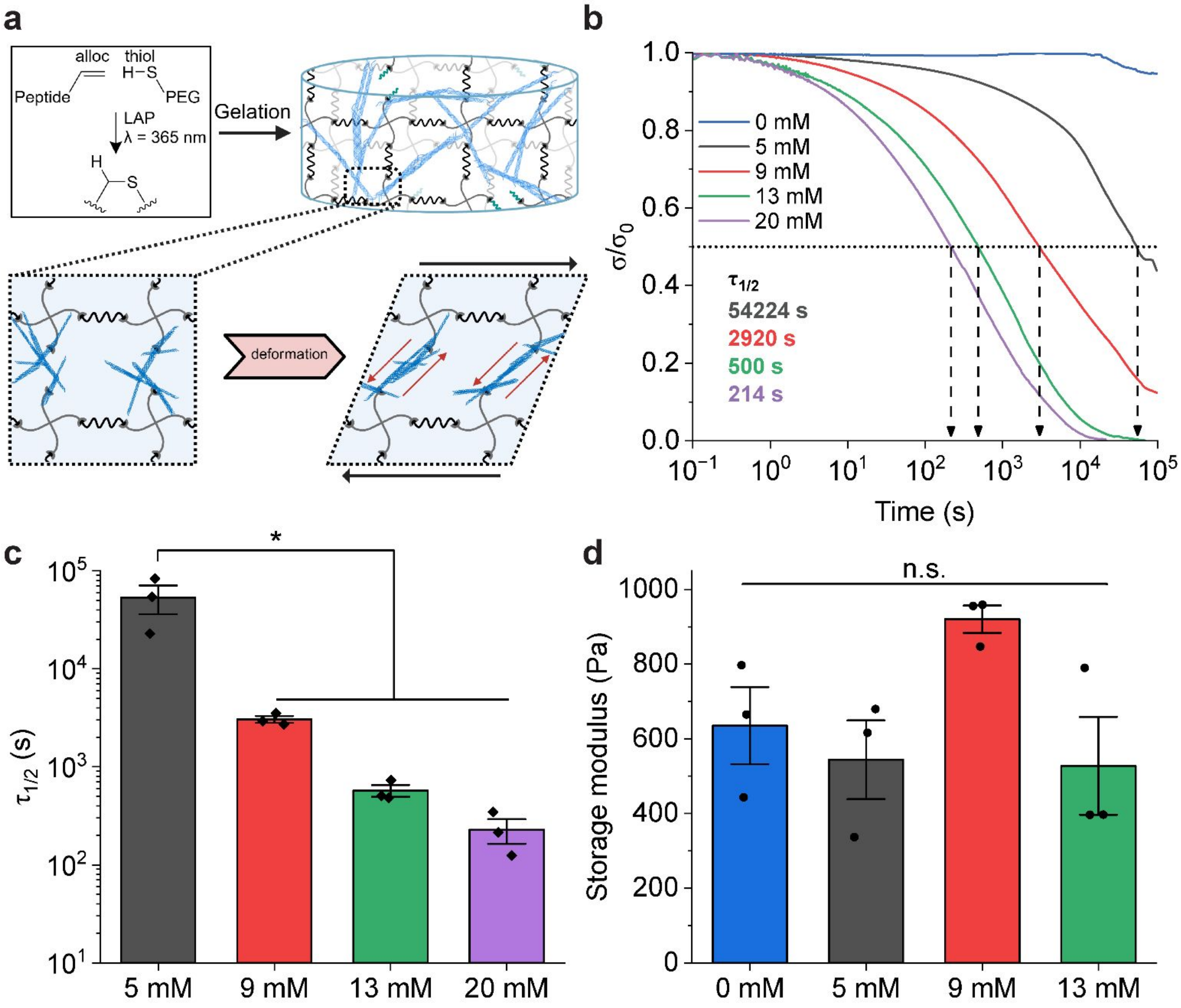

Figure 4. Viscoelasticity of mfCMP-PEG hydrogels. a) Hydrogel photocrosslinking schematic and proposed mechanism of stress relaxation in mfCMP-PEG hydrogels in response to a deformation:

physical crosslinks relax as the network is deformed and mfCMP fibrils slide past each other, dissipating mechanical energy. b) In situ stress relaxation profiles of representative hydrogels that contain increasing concentrations of mfCMPa-az. All replicates shown in **Figure S15**. c) $\tau_{1/2}$ of hydrogels that contain increasing concentrations of mfCMPa-az. d) Equilibrium-swollen storage moduli of hydrogels containing 0 mM, 5 mM, 9 mM, and 13 mM of mfCMPa-az. Means ± standard error for each condition are shown for (n = 3) independent sample measurements. Statistical significance was determined by one-way ANOVA with Tukey's multiple comparisons test. Statistical significance is shown (*$p < 0.05$; n.s. $p > 0.05$).

2. The storage modulus for the 20 mM mfCMP networks is not included in figure 4d, despite the respective stress relaxation data being reported.

Response: While were able to measure stress relaxation for in situ formed 20 mM mfCMP hydrogels, the swollen storage modulus of this formulation could not be measured owing to loss of hydrogel integrity with equilibrium swelling and sample processing. We have clarified this within the revised manuscript.

Excerpts from revised text:

Page 18:
Note that the equilibrium storage moduli of hydrogels containing 20 mM mfCMPa-az could not be measured owing to their loss of integrity with equilibrium swelling and sample processing.

3. In figure S14, there appears to be staining for the mfCMP in the purely elastic sample, though I believe from the text that this sample should not contain any mfCMP.

Response: In this figure, there is background shown from the AF647-alkyne fluorophore used to stain the mfCMPs in all conditions, including the purely elastic sample. This has been clarified within the caption for the figure.

Excerpts from revised text:

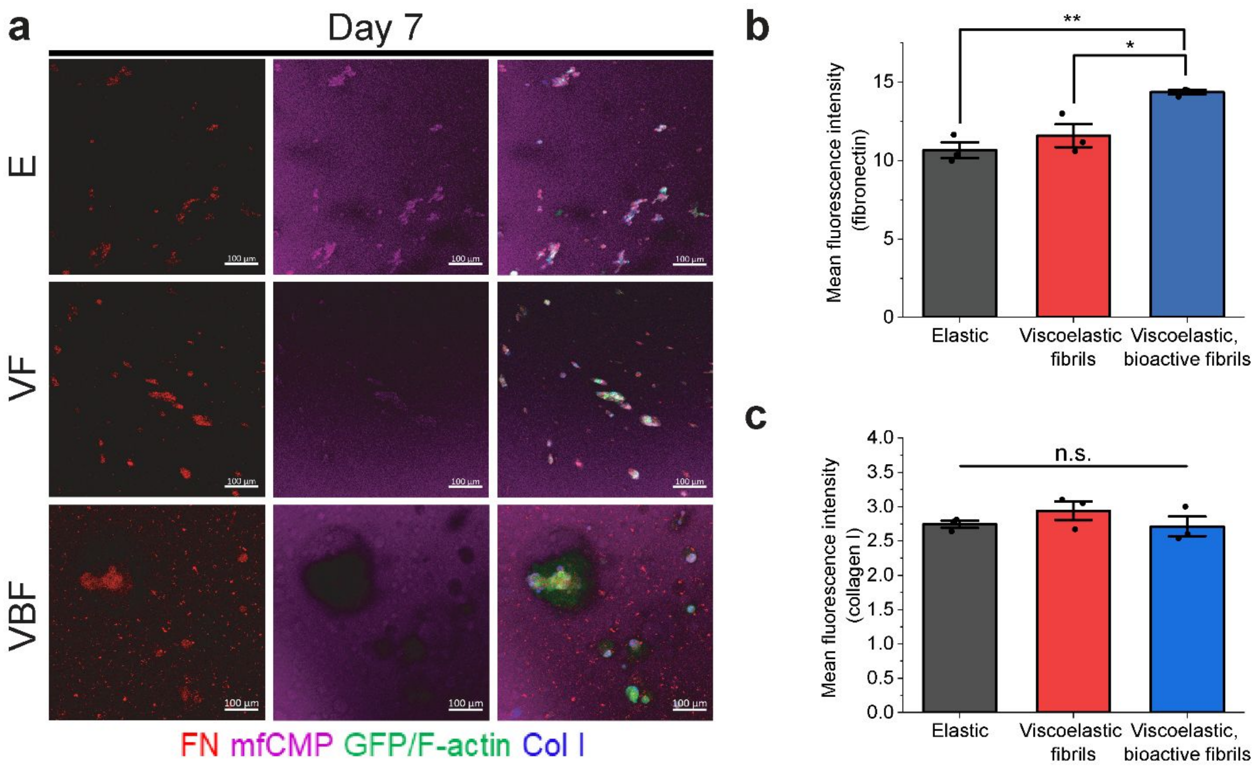

Figure S22. Secreted protein staining. a) Hydrogels with encapsulated T47D cells were stained for fibronectin, F-actin, collagen I, and mfCMP. Mean fluorescence intensity of immunostained b) fibronectin and c) collagen I in E, VF, and VBF hydrogels 7 days after encapsulation of T47Ds. Note that in all conditions, there remains background fluorescence from the alkyne-AlexaFluor 647 used to label mfCMP. Means ± standard error are shown for each condition for (n = 3) independent sample measurements. Statistical significance was determined by one-way ANOVA with Tukey's multiple comparisons test. Statistical significance is shown (*p < 0.05; **p < 0.01).

Reviewer: 3

Comments:
In their manuscript, Kloxin and coworkers describe the use of multifunctional collagen-mimetic peptide (mfCMP)-containing hydrogels to create synthetic collagen network mimics with tunable viscoelasticity. The authors extend their previous system of mfCMP-containing hydrogels to include mfCMPs embedded with integrin-binding sequences, thereby enabling the controlled presentation of distinct cell-binding sites for modeling both intact and denatured collagen I. The authors describe the design of their mfCMPs to include functional groups for (1) end-to-end fibril elongation via electrostatic interactions (using (PKG) and (DOG), (2) covalent cross-linking via photoinitiated thiol-ene click chemistry previously used by the group (using alloc-functionalized lysine), and (3) reactive handles for in situ hydrogel labeling (using azide-functionalized lysine). The authors describe the self-assembly of their mfCMPs into triple helices, which hierarchically assemble into fibrillar structures, as visualized by transmission electron microscopy. The authors confirmed the incorporation of fibrillar, fluorophore-labeled mfCMPs into their PEG-SH hydrogel system using a super-resolution imaging technique (stochastic optical reconstruction (STORM) imaging) that allows for the localization of single fluorophores. Furthermore, the authors demonstrated that incorporating mfCMPs into hydrogels altered the material's shear-stress relaxation properties, with hydrogels containing higher concentrations of mfCMPs exhibiting greater viscoelasticity and shorter stress-relaxation half-times. The authors attributed this shift in viscoelasticity to a change in the crosslinking network, from irreversible covalent linkages between polymers to reversible physical linkages between the mfCMPs, thereby enabling tunable viscoelasticity in their system. Finally, the authors performed cell culture experiments using T47D-GFP breast cancer cells in dual-layer hydrogels of varying compositions to evaluate changes in cellular responses. The authors assessed three different hydrogel formulations: (1) a hydrogel with pendant integrin binding peptides added but no mfCMPs (referred to as elastic (E) formulation), (2) a hydrogel with pendant integrin binding peptides added and an mfCMP that formed fibrillar structures but did not include an integrin binding sequence (referred to as viscoelastic fibrillar (VF) formulation), and (3) a hydrogel with no pendant integrin binding peptides added and with fibrillar mfCMPs incorporating both RGD and FOGER integrin binding sequences (referred to as viscoelastic, bioactive fibrillar (VBF) formulation). The authors showed that cells grown on their VF and VBF hydrogels produced larger cell clusters, with the VBF group forming the largest clusters and exhibiting the highest fibronectin production. However, all three groups showed similar cell viability and proliferation, as measured by Ki-67 nuclear marker expression. The authors concluded that their synthetic, self-assembling mfCMP-hydrogel system enables tunable viscoelasticity and bioactivity and represents a step toward better modeling of diverse biological collagen environments.

Response: We thank the reviewer for their thorough and thoughtful summary and constructive feedback.

Comments:

1. In Figure 2, could the authors comment on why the melt curve for the mfCMPa-G-az FOGER-containing peptide has a different shape than the mfCMP-R-az and mfCMP-a-az ones?

<u>Response</u>: We hypothesize that the melting curve from mfCMPa-G-az has a different shape when compared to the others because of this peptide's increased triple helical stability. Rather than a different shape, we believe that the curve is the same shape, shifted to the right. Since mfCMPa-G-az has glycine residues in the integrin binding domain that align with the X-Y-G polyproline helical structure, their triple helical assemble is more stable compared to mfCMPa-R-az that has a proline in the G of the X-Y-G structure. Additionally, mfCMPa-G-az has a more stable triple helical conformation compared to mfCMPa-az, owing to it having 6 additional $(POG)_n$ repeats. Further, to better characterize the differences and similarities between the melting of the different mfCMP designs in response to specific reviewer comments, we have performed additional quantitative analyses and revised Figure 2 and Figure S6.

<u>Excerpts from revised text:</u>

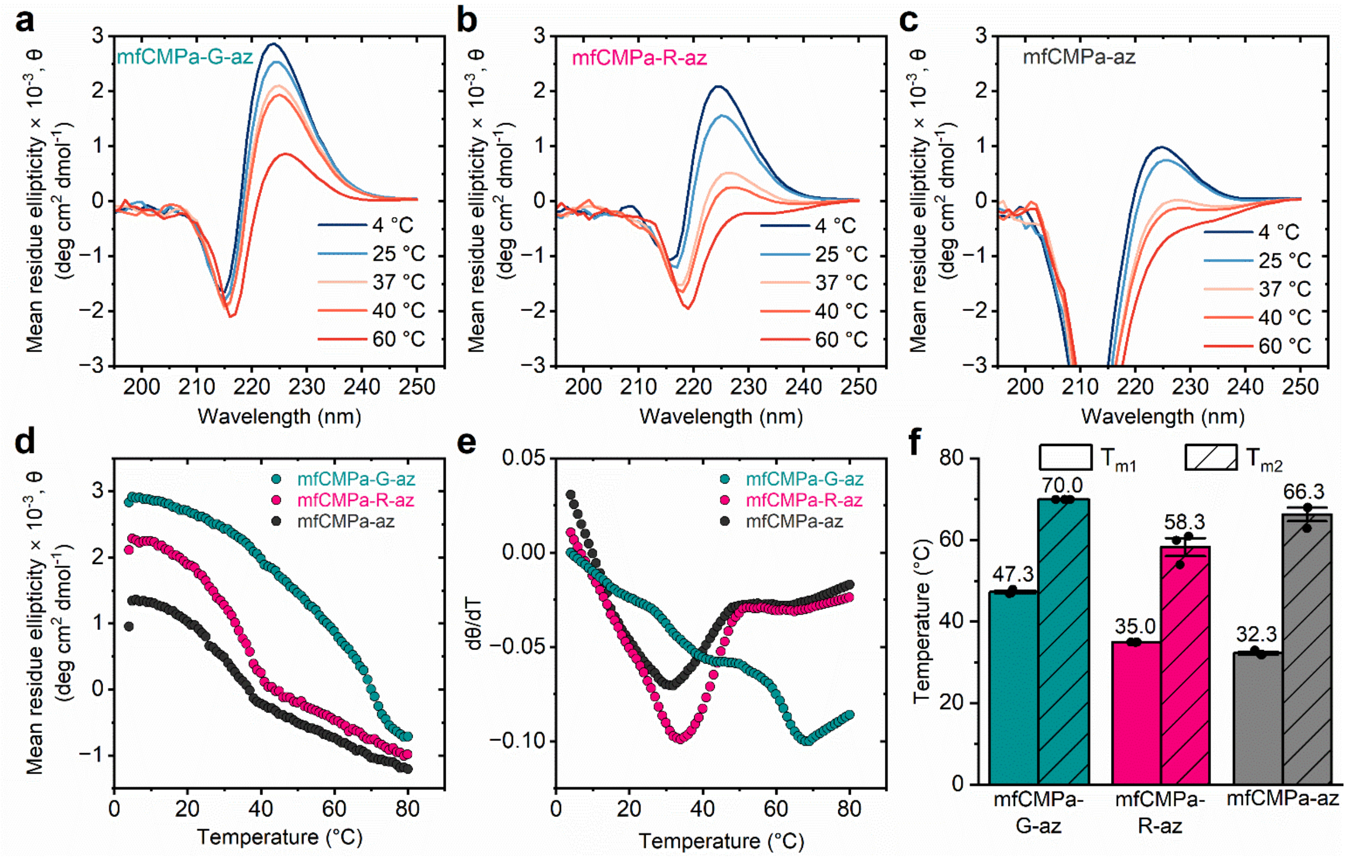

Figure 2. Triple helix characterization of mfCMPs in Dulbecco's phosphate-buffered saline by CD spectroscopy. Wavelength scans of a) mfCMPa-G-az, b) mfCMPa-R-az, and c) mfCMP-az as temperature is ramped from 4 ˚C to 80 ˚C show the characteristic polyproline type II peak (225 nm). d) Temperature scans of mfCMPs at 225 nm show nonlinear "melting" as temperature increases, indicating that triple-helical conformations are present. e) First-order derivative curves of d) show minima that describe melting events for mfCMP triple helices. f) Temperatures of melting events of mfCMPs. The two largest melting events are reported for each sequence as determined by the inflection points of (d), identified by analysis of second-order derivatives of the data in (d) (**Figure S6**). Results shown are from a representative sample of multiple trials ($n = 3$). All replicates are shown in **Figures S5-6**.

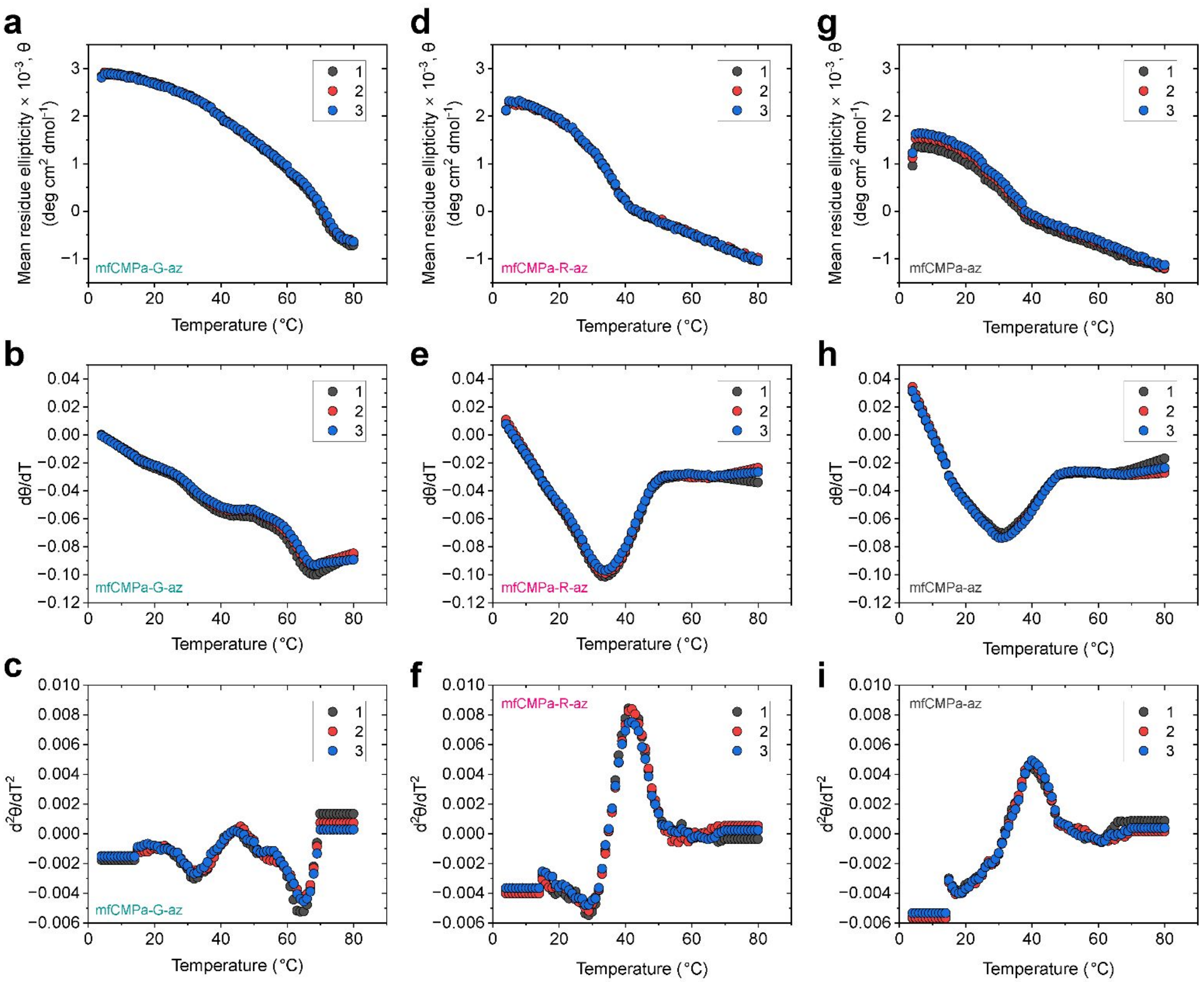

Figure S6. Temperature scans of mfCMPs at 225 nm with their first- and second-order derivatives. a-c) mfCMPa-G-az, d-f) mfCMPa-R-az, and g-i) mfCMPa-az in DPBS measured at 0.3 mM after assembly. For each, (*n* = 3) independent samples were measured. The two points where the second order derivative plot crosses zero with the highest changes in magnitude were identified and reported as the prominent melting temperatures (**Figure 2)**.

2. In the caption of Figure 4, the descriptions for panels a) and b) are swapped.

Response: We thank the reviewer for catching this inadvertent typographical error and have changed Figure 4 accordingly.

Excerpts from revised text:

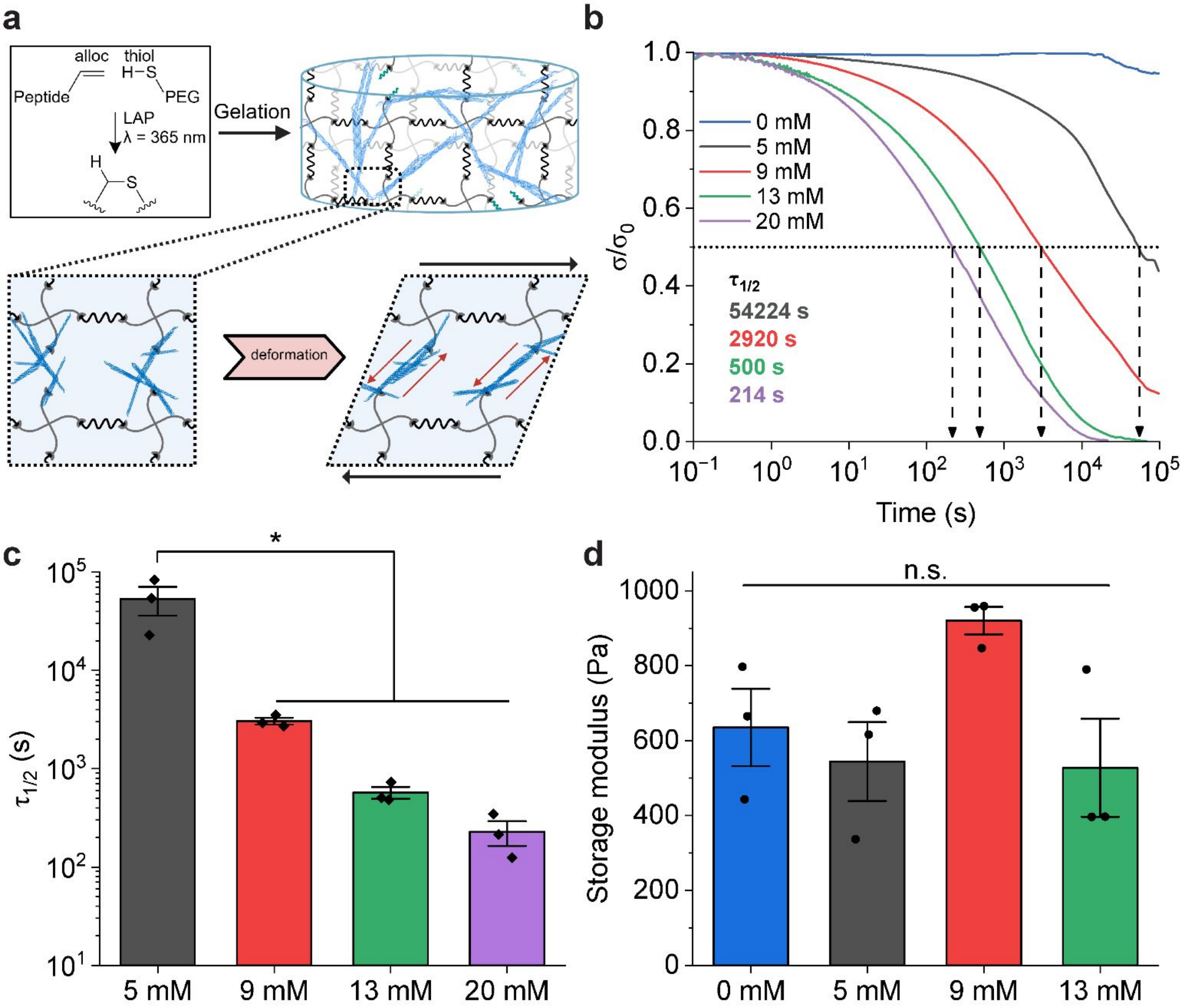

Figure 4. Viscoelasticity of mfCMP-PEG hydrogels. a) Hydrogel photocrosslinking schematic and proposed mechanism of stress relaxation in mfCMP-PEG hydrogels in response to a deformation: physical crosslinks relax as the network is deformed and mfCMP fibrils slide past each other, dissipating mechanical energy. b) In situ stress relaxation profiles of representative hydrogels that contain increasing concentrations of mfCMPa-az. All replicates shown in **Figure S15**. c) $\tau_{1/2}$ of hydrogels that contain increasing concentrations of mfCMPa-az. d) Equilibrium-swollen storage

moduli of hydrogels containing 0 mM, 5 mM, 9 mM, and 13 mM of mfCMPa-az. Means ± standard error for each condition are shown for (n = 3) independent sample measurements. Statistical significance was determined by one-way ANOVA with Tukey's multiple comparisons test. Statistical significance is shown (*$p < 0.05$; n.s. $p > 0.05$).

3. Could the authors include a comment on how the stress relaxation properties of their mf-CMP-containing hydrogels might be different while still preserving a similar equilibrium-swollen storage modulus?

Response: The storage modulus of these hydrogels as defined by rubber elasticity theory (e.g., G' ~ $\rho_x Q^{-\frac{1}{3}}$) is dominated by weight percent of PEG and its crosslinking, where a balance of non-assembling peptide linker and assembled mfCMP linker are used. After swelling, a similar increase in volume is seen in all conditions, decreasing the moduli to similar levels. The stress relaxation behavior is then dependent on the mfCMP concentration and how many physical crosslinks there are relative to covalent crosslinks, where hypothesize that the physical crosslinks can dissipate stress. We have clarified these points in the main text. Additionally, we have clarified the hydrogel formulations with the addition of Tables S2 and S3.

Excerpts from revised text:

Page 16:

In the hydrogel, assembled mfCMPs are covalently crosslinked into the polymer network, and the mfCMPs then serve as physical crosslinks within the network owing to their physical interactions with each other via hydrogen bonding within triple helices and salt bridging within fibrils.

Page 18:

After swelling, a similar increase in volume is seen in all conditions, decreasing the moduli to similar levels, where the equilibrium-swollen storage moduli of the hydrogels containing 0, 5, 9, or 13 mM mfCMPa-az were statistically similar and remained in the range of 500-1000 Pa (**Figure 4d**).

Table S2. Concentrations of monomers used to form hydrogels for in situ gelation time sweeps, frequency sweeps, strain sweeps, and stress relaxation experiments.

	0 mM	5 mM	9 mM	13 mM	20 mM
PEG (mM thiol)	20	20	20	20	20
Linker Peptide (mM alloc)	18	13	9	5	0
mfCMPa-az (mM alloc)	0	5	9	13	20
Pendent RGD (mM alloc)	2	2	2	2	0

Table S3. Concentrations of monomers used to form hydrogels for stress relaxation experiments.

	E	VF	VBF
PEG (mM thiol)	20	20	20
Linker Peptide (mM alloc)	13	13	13
mfCMPa-az (mM alloc)	0	5	3
mfCMPa-G-az (mM alloc)	0	0	1
mfCMPa-R-az (mM alloc)	0	0	1
Pendent RGD (mM alloc)	1	1	0
Pendent GFOGER (mM alloc)	1	1	0

4. Could the authors comment on why they chose to perform the stress-relaxation experiments exclusively with their mfCMP-a-az peptide, which lacks the FOGER and RGD integrin-binding motifs? Additionally, could the authors comment on how they expected the mechanical properties of their system to differ compared to their previously studied system (Kloxin et al., Biomater. Sci. 2020), which used a similar mfCMP lacking an integrin binding sequence and that seems to differ only in the addition of the N-terminal azide-functionalized lysine and glycine residues? Was the purpose of this experiment to evaluate the properties of their existing system further?

Response: We chose to perform stress relaxation experiments first on a mfCMP design (mfCMPa-az) and hydrogel formulation that had been shown previously to influence cellular responses (mesenchymal cell morphology and motility) to understand its stress relaxation profile and ask the question if stress relaxation was imparted by the inclusion of mfCMP, which we had not previously examined. Based on those observations, we then tested the hypothesis that integrin-binding peptides could be presented from the mfCMP while still achieving similar stress relaxation profiles. Together, these formulations then allowed us to examine the effects of elasticity (E), viscoelasticity (VF), or viscoelasticity and more native-like integrin binding (VBF) on cellular responses specifically focusing on spheroid formation and growth. We appreciate from this reviewer's and other reviewers' comments that these points were not fully clear in the original manuscript and have edited the text to clarify. Additionally, we have performed new experiments to support the properties of these new formulations (Figures S19-20).

Excerpts from revised text:

Page 17:

We chose to perform stress relaxation experiments first on a mfCMP design (mfCMPa-az) and hydrogel formulation that had been shown previously to influence cellular responses (mesenchymal cell morphology and motility[37]) to understand its stress relaxation profile and probe if stress relaxation was imparted by the inclusion of mfCMP, which we had not previously examined.

Page 22:

Further, each hydrogel composition (E, VF, VBF) was probed for viscoelastic behavior (**Figure S19**), where the VF and VBF formulations showed significantly greater stress relaxation than the E formulation over a period of 10^5 seconds. Additionally, these hydrogel compositions showed statistically similar swollen equilibrium storage moduli (**Figure S20**).

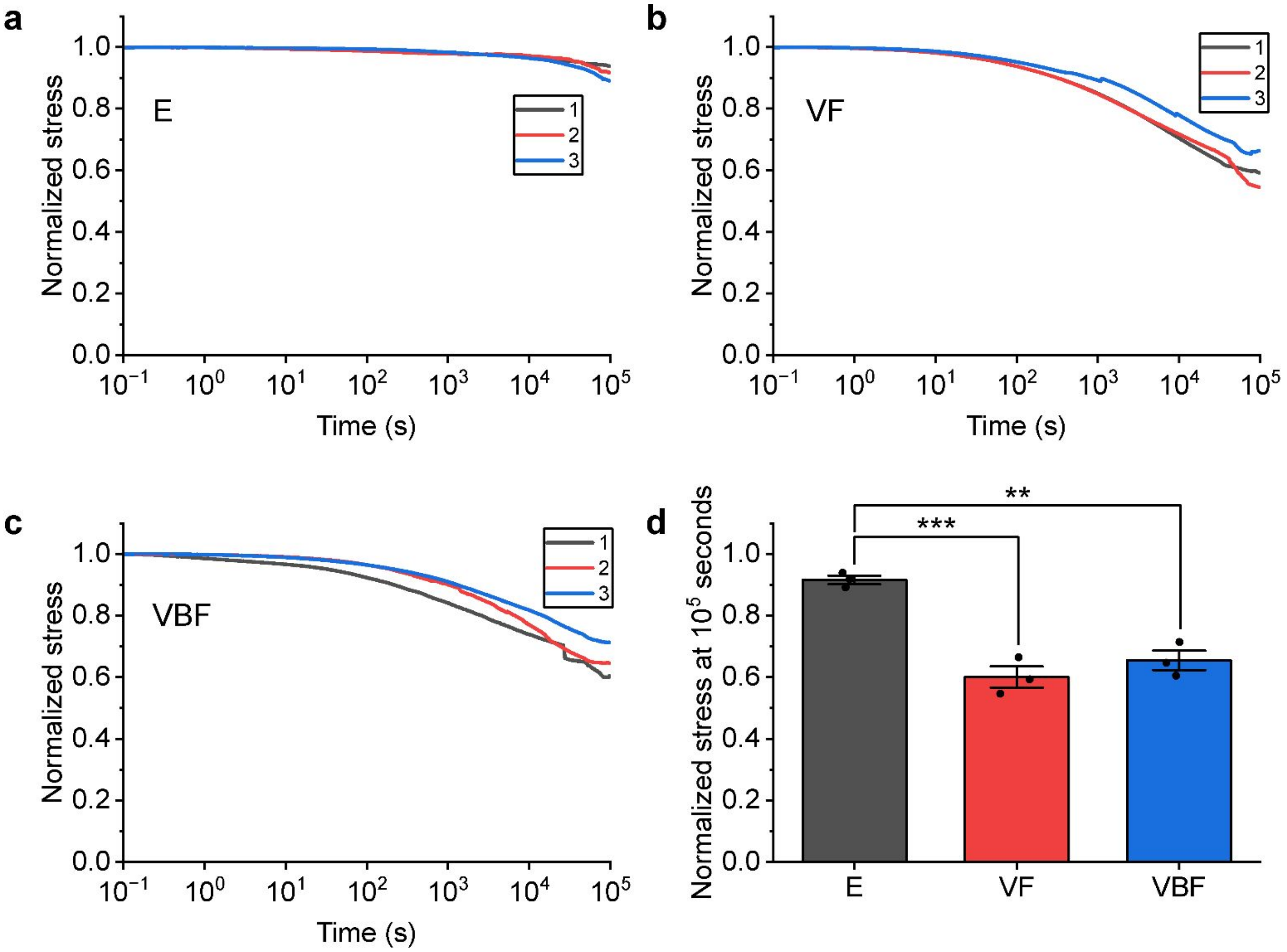

Figure S19. Stress relaxation behavior of hydrogel formulations used in cellular studies: a) E, b) VF, and c) VBF. Stress is normalized to the maximum stress measured for each sample. d) Comparison of average values for stress relaxation at 10^5 seconds for these compositions. Means ± standard error are shown for each condition for (n = 3) independent sample measurements. Statistical significance was determined by one-way ANOVA with Tukey's multiple comparisons test. Statistical significance is shown (*p < 0.05; **p < 0.01; ***p < 0.001; ****p < 0.0001).

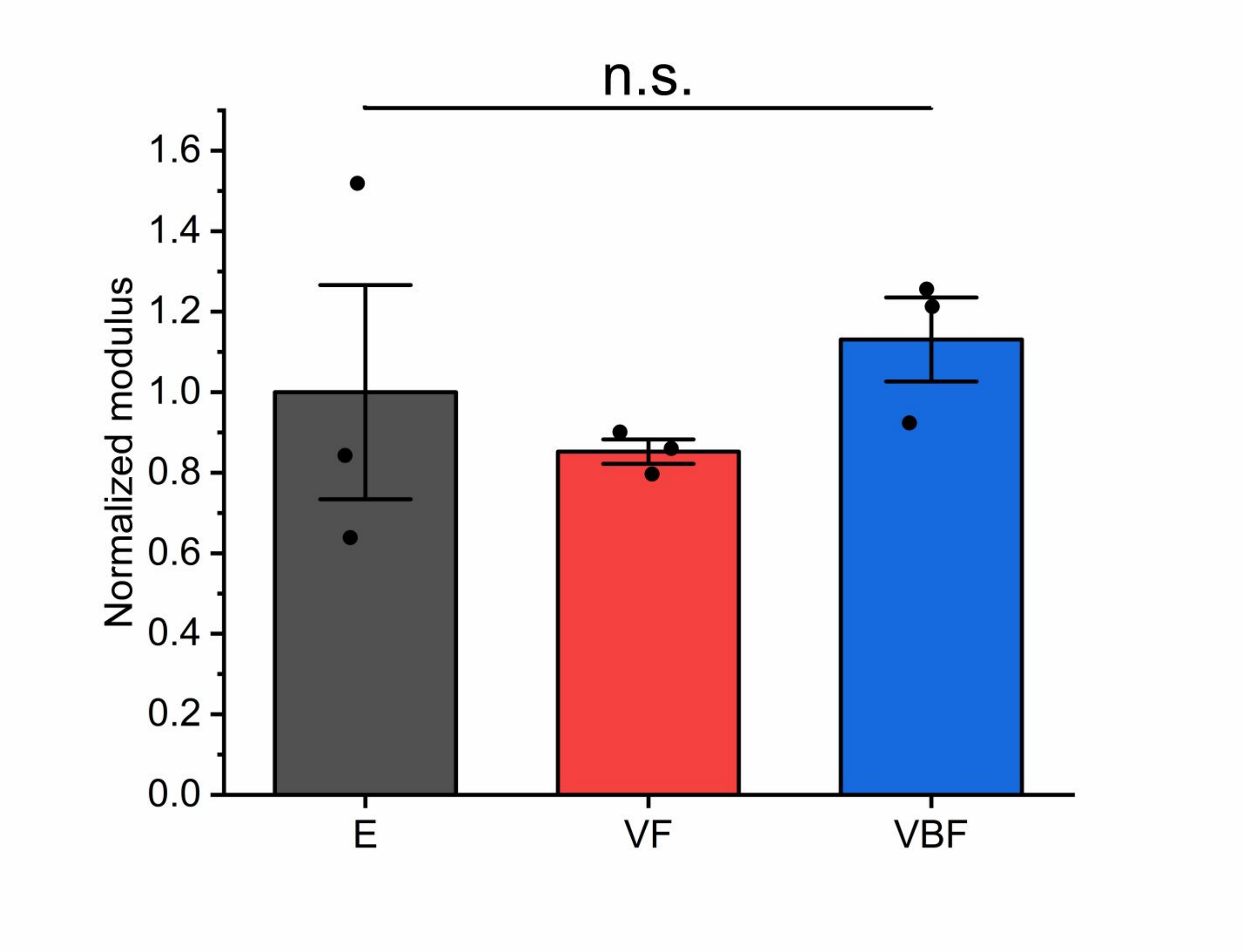

Figure S20. Equilibrium-swollen storage moduli of E, VF, and VBF hydrogel compositions, normalized to the E condition. Means ± standard error are shown for each condition for (n = 3) independent sample measurements. Statistical significance was determined by one-way ANOVA with Tukey's multiple comparisons test (n.s. = no statistical significance).

5. Could the authors include a comment reconciling their previous statement that "Inclusion of the alloc reactive handle was allowing the mfCMPs to serve as crosslinks within

the photopolymerized network, enabling the formation of hydrogels with integrated assembled mfCMP nanostructures while maintaining control over mechanical properties." (Kloxin et al., Biomater. Sci. 2020) with the physical, reversible mfCMP linkages imparting different stress relaxation properties described in this work? Could the authors clarify whether they are referring to covalent crosslinks between the mfCMPs and the hydrogel polymer network in this previous work, but attributing the change in stress relaxation properties in this work to physical linkages between the mfCMPs?

Response: We appreciate the reviewer's comment and the opportunity to clarify. The statement in that previous work is describing the covalent linkages between the alloc of the mfCMP and the thiol of the PEG that allows for the mfCMPs to be incorporated into the hydrogel polymer network, as the reviewer nicely described in their last sentence. The mfCMPs then serve as physical crosslinks because they are physically interacting with each other via hydrogen bonding within triple helices and salt bridging within fibrils. The quoted, published statement is the same as what is being described in this work in terms of the reversible physical crosslinks, where in this new contribution we are testing the hypothesis that these physical crosslinks impart stress relaxation to the hydrogel.

Excerpts from revised text:

Page 16:

In the hydrogel, assembled mfCMPs are covalently crosslinked into the polymer network, and the mfCMPs then serve as physical crosslinks within the network owing to their physical interactions with each other via hydrogen bonding within triple helices and salt bridging within fibrils.

6. For their cellular response experiments, could the authors clarify the purpose and configuration of the pendant FOGER integrin-binding peptides in the E and VF conditions? Did the authors verify that this peptide was in triple-helical conformation? Given that this sequence must be in a triple-helical conformation to be active, as the authors note in the Introduction section, I am concerned that it is not an appropriate control for comparison with the fibrillar mfCMPs if its conformation was inactive for cell recognition.

Response: We thank the reviewer for their inquiry and feedback. The purpose of the GFOGER peptides in the E and VF was for display of the integrin-binding sequence from the hydrogel polymer network for comparison to cell response with its display from the fibrils in VBF. We agree with the reviewer that the GFOGER sequence in E and VF needs to be in the triple helical confirmation to be active. Accordingly, we have performed new CD experiments probing the melting behavior of the pendent GFOGER peptide used in the E and VF conditions. These

results confirm that this GFOGER pendent peptide does form a triple helix with a melting temperature of 49.4 ± 0.4 ˚C (Figure S7).

Excerpts from revised text:

Page 22:

Note that the pendent GFOGER peptide used also forms a stable triple helix characterized by CD with a T_m of 49.4 ± 0.4 ˚C (**Figure S7**).

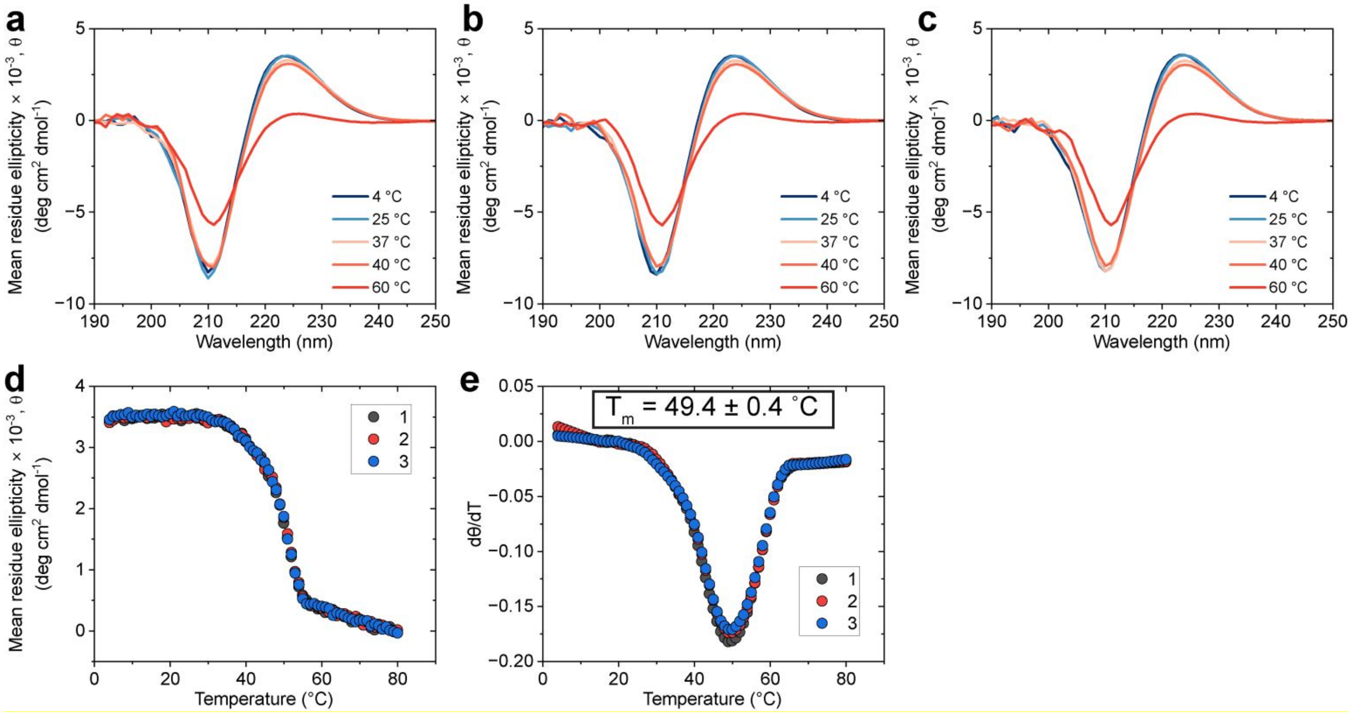

Figure S7. CD measurements for pendent alloc-functionalized GFOGER peptide in DPBS measured at 0.3 mM after assembly. a-c) Wavelength scans, d) temperature scans, and e) first-order derivatives of the temperature scan curves. For each type of scan, (n = 3) independent samples were measured.

7. In Figure 5, could the authors elaborate on the rationale for testing the cell response of their system exclusively with the T47D cell line? Given that a novel aspect of the mfCMPs presented in their manuscript seems to be the inclusion of the integrin-binding motif, why did the authors choose a cell line that forms clusters and is less able to show interaction between the cell and the mfCMP–hydrogel system? Especially given that previous work by the group has used hMSC cells to show differences in cell morphology and interaction with mfCMP-containing

hydrogels? Could the authors either show both cell types or provide a compelling rationale for testing only the T47D cells?

Response: We appreciate the reviewer's perspective and feedback. As mentioned by the reviewer, in our previous study (Ford, et. al, J. Mat. Chem B., 2024) we have shown how human lung fibroblasts show an elongated, activated morphology with increased cell speed and cluster size after being encapsulated in hydrogels with mfCMP. In the studies presented here in this new contribution, we wished to focus on the effects of the designed materials on cell proliferation and growth, and accordingly, selected a breast cancer cell line known for spheroid formation with limited migration (luminal A, epithelial-like T47Ds). We had shown in prior work the importance of β1-integrin binding in spheroid growth with these cells in a hydrogel without mfCMPs (Sawicki, et al., APL Bioengineering, 2019). Additionally, others in the literature have shown differences in spheroid growth based on viscoelasticity (Sievers, et. al., Adv. Hea. Mat., 2023). We hypothesized that mfCMPs in the same concentration of used our previous study (5 mM mfCMPa-az in Ford, J Mat Chem B, 2024) would affect cellular behavior with the breast cancer cell line with potential differential responses based on viscoelasticity (E vs. VF), integrin-binding (VF vs. VBF), or both (E vs. VBF).

Excerpts from revised text:

Page 20:

In these studies, we wished to focus on the effects of the designed materials on cell proliferation and growth, and accordingly, selected luminal A breast cancer cells known for spheroid formation and limited migration,[69] where we have previously observed the importance of β1 integrin-binding by RGDS and GFOGER in T47D cluster growth within elastic PEG-peptide hydrogels.[67]

Page 21:

Here, we chose a total mfCMP concentration of 5 mM based on our previous studies showing this concentration was effective for promoting increased fibroblast speed and elongation.[37]

8. In Figure 5/S14, could the authors comment on why cells might be producing more fibronectin in their VBF hydrogel system? And, why is this increased fibronectin production and cell cluster volume evidence that their VBF system is a better model of the collagen-rich microenvironments of tumors?

Response: We thank the reviewer for their inquiry. β1-integrin engagement and clustering been reported to promote cell proliferation and fibronectin deposition, suggesting a potential mechanism by which increased fibronectin expression was observed in VBF (Mana, et. al., Life

Sci. All., 2022). Based on the reviewers' comments, we have performed new β1 integrin immunostaining studies toward suggesting a potential mechanism for the differences in cluster growth and fibronectin production in the VBF condition (Figure S23). While β1 integrin is observed in all conditions, in the VBF condition, we observed small puncta-like fluorescent regions suggesting integrin clustering, which is reported more broadly as a cellular response to collagen I and supporting the relevance of the integrin-binding mfCMPs (Ostrowska-Podhorodecka. et. al., J. of Cell Sci., 2021).

While the VBF condition captures some of these tumor microenvironment complexities, our aim with this work was to decouple viscoelasticity from integrin binding presentation and probe how those effects change cellular response, inspired by aspects of collagen-rich microenvironments in which cancer cells grow but not specifically to create a better model of the tumor microenvironment.

Excerpts from revised text:

Page 23:

Integrin β1 was also stained in all conditions (**Figure S23**). While β1 integrin is observed in all conditions, small puncta-like fluorescent regions were observed the in VBF condition, suggesting integrin clustering[71] which has been reported more broadly as a cellular response to collagen I.[72] β1-integrin clustering also has been reported to promote fibronectin deposition, suggesting a potential mechanism by which increased fibronectin expression was observed in VBF. Overall, these observations support the relevance of the integrin-binding mfCMPs to capture aspects of the functions of collagen I with a more native-like conformation for presentation of the integrin-binding site from the fibrils, rather than as a pendant group from amorphous PEG, leading to difference in β1-integrin engagement and cellular responses.

Page 24:

While the VBF condition captures some of aspects of the tumor microenvironment ECM, our aim with this work was to decouple viscoelasticity from integrin binding presentation and probe how those effects change cellular response, inspired broadly by aspects of the collagen-rich microenvironments in which cancer cells grow with future opportunities for integrating complexity for representing specific tissue microenvironments.

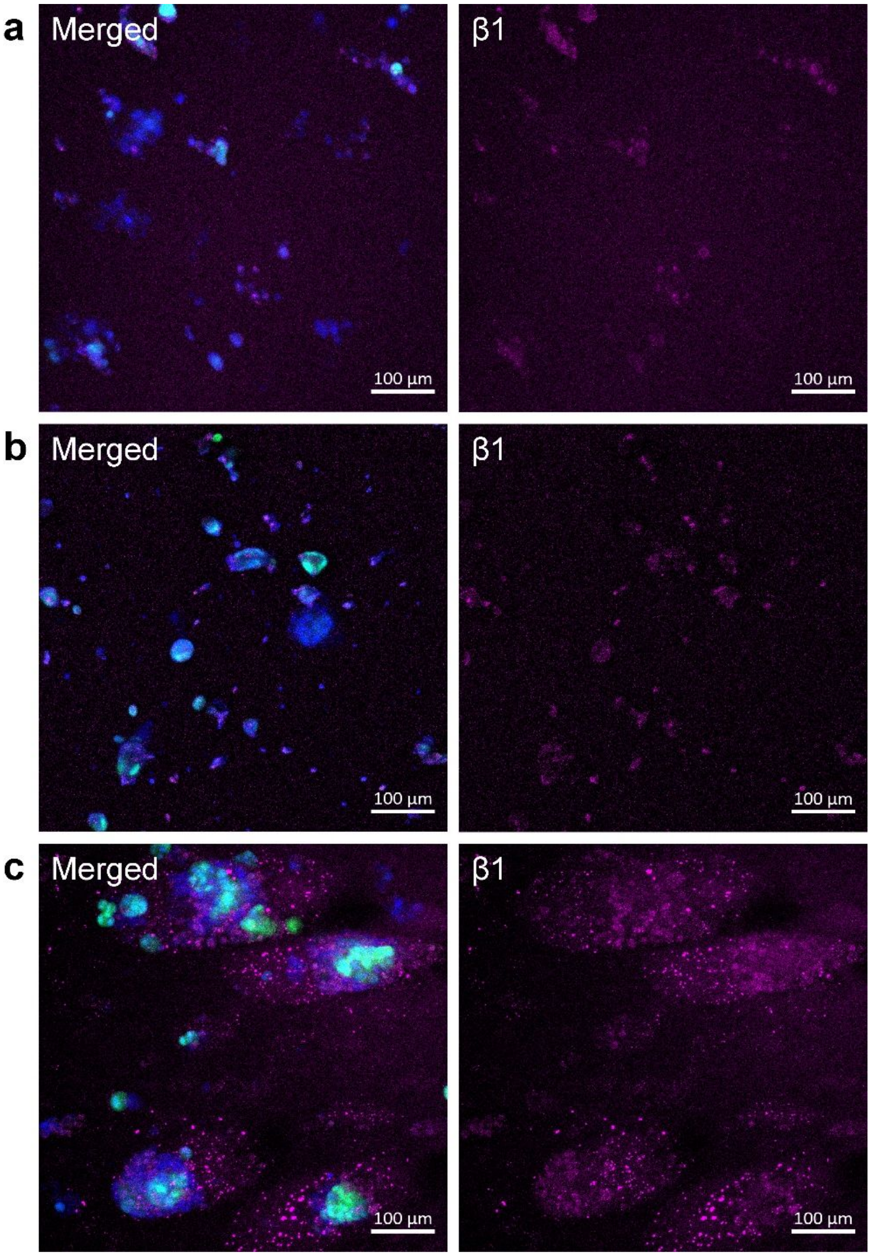

Figure S23. β1 integrin staining of fixed hydrogels for conditions a) E, b) VF, and c) VBF. Fixed samples and stained (nuclei, blue; GFP, green; β1 integrin, magenta; scale bars = 100 μm). We hypothesize that the fluorescent puncta observed for β1 integrin in VBF condition are clustered integrins in response to the fibrillar, bioactive mfCMPs. Note, α2 integrin, which is associated with α2β1 binding to collagen I, is not internalized intracellularly when integrin turnover occurs,[9] which we hypothesize leads to some retention of integrins surrounding some cell clusters.

9. In Figure S14, the authors report using an anti-collagen I antibody. Could the authors comment on differences in type I collagen production between cells cultured on their E, VF, and VBF formulations? Or, comment on why they chose not to quantify type I collagen production between the conditions and only quantify fibronectin expression?

Response: We thank the reviewer for their feedback. Accordingly, we have quantitatively analyzed collagen I deposition via fluorescence and found no differences amongst the E, VF, and VBF conditions (Figure S22). While quantitative differences in collagen I expression were not observed, localization of secreted collagen I within the pericellular matrix within these synthetic ECM is consistent with prior observations with other cell types (Locke. et. al, Tis. Eng. Part C, 2020).

Excerpts from revised text:

Page 23:

Interestingly, while collagen I and fibronectin deposition were observed in all conditions, increased fibronectin was observed in the VBF condition (**Figure S22**). While quantitative differences in collagen I expression were not observed, localization of secreted collagen I within the pericellular matrix within these synthetic ECM is consistent with prior observations with other cell types.[70]

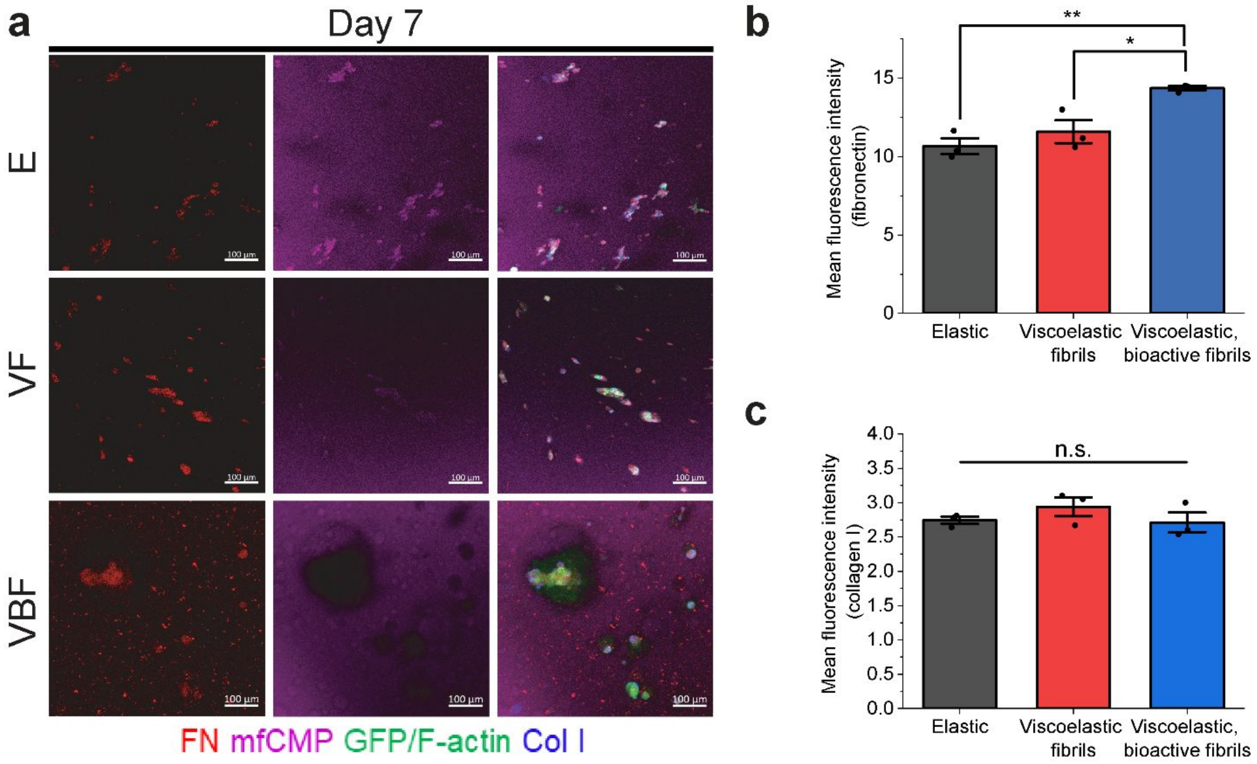

Figure S22. Secreted protein staining. a) Hydrogels with encapsulated T47D cells were stained for fibronectin, F-actin, collagen I, and mfCMP. Mean fluorescence intensity of immunostained b) fibronectin and c) collagen I in E, VF, and VBF hydrogels 7 days after encapsulation of T47Ds. Note that in all conditions, there remains background fluorescence from the alkyne-AlexaFluor 647 used to label mfCMP. Means ± standard error are shown for each condition for (n = 3) independent sample measurements. Statistical significance was determined by one-way ANOVA with Tukey's multiple comparisons test. Statistical significance is shown (*$p < 0.05$; **$p < 0.01$).

Reviewer: 4

Comments:
With pleasure, I have read the article “Synthetic Surrogates of Collagen-Rich Microenvironments: Integrating Modular Bioactive Fibrillar Structure and Tunable Viscoelasticity via Multifunctional Assembling Peptides” by Castro and coworkers. The paper is relevant timely, and well executed. There are a few places where the article could be strengthened in presentation and analysis.

Response: We thank the reviewer for their thoughtful and constructive feedback.

Major:

1. The claim is that these materials integrate adhesion motifs non-stochastically within a hydrogel; however, the hydrogel formation and functionalization results in a fairly stochastic nature. Can this claim be sharpened? I understand the fact that the adhesion sites are positioned in the self-assembled structures, yet these are still stochastically incorporated in the microstructure of the hydrogel. Sharpening this claim will help the understanding/acceptance.

Response: We thank the reviewer for their inquiry. We would like to clarify that the claim in the manuscript is that the mfCMPs present the integrin binding motifs from a fibrillar conformation, compared throughout the bulk through pendent peptide group presentation. While the fibrils are randomly distributed throughout the hydrogel, we hypothesize that there is local organization of integrin binding sequences within the fibril and triple helical assembles of the mfCMPs. Further, we hypothesized that this local organization of binding sites provided by the mfCMPs would affect cell interactions with the hydrogel via integrin clustering. To probe this hypothesis, we have performed new experiments to stain β1-integrin. In the VBF condition, we observed small puncta-like fluorescent regions suggesting integrin clustering, which is reported more broadly as a cellular response to collagen I and supporting the relevance of the integrin-binding mfCMPs (Mana, et. al., Life Sci. All., 2022.; Ostrowska-Podhorodecka. et. al., J. of Cell Sci., 2021).

Excerpts from revised text:

Page 23:

Integrin β1 was also stained in all conditions (**Figure S23**). While β1 integrin is observed in all conditions, small puncta-like fluorescent regions were observed the in VBF condition, suggesting integrin clustering[71] which has been reported more broadly as a cellular response to collagen I.[72] β1-integrin clustering also has been reported to promote fibronectin deposition, suggesting a potential mechanism by which increased fibronectin expression was observed in VBF. Overall, these observations support the relevance of the integrin-binding mfCMPs to

capture aspects of the functions of collagen I with a more native-like conformation for presentation of the integrin-binding site from the fibrils, rather than as a pendant group from amorphous PEG, leading to difference in β1-integrin engagement and cellular responses.

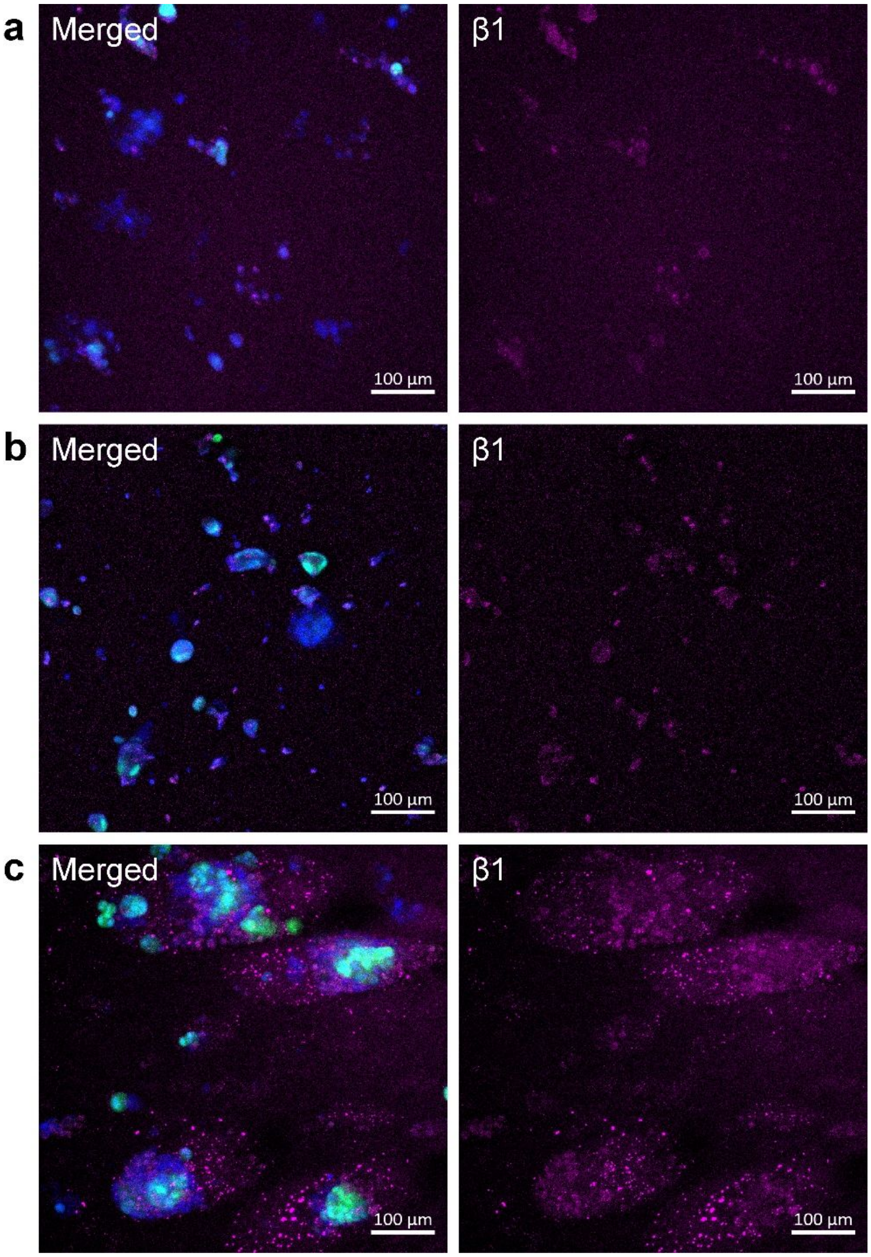

Figure S23. β1 integrin staining of fixed hydrogels for conditions a) E, b) VF, and c) VBF. Fixed samples and stained (nuclei, blue; GFP, green; β1 integrin, magenta; scale bars = 100 µm). We hypothesize that the fluorescent puncta observed for β1 integrin in VBF condition are clustered integrins in response to the fibrillar, bioactive mfCMPs. Note, α2 integrin, which is associated

with α2β1 binding to collagen I, is not internalized intracellularly when integrin turnover occurs,[9] which we hypothesize leads to some retention of integrins surrounding some cell clusters.

2. Can the rheology be modelled via a maxwell or similar function? I think that you do not have just changes in the t ½, but also in the onset of the relaxation and the balance between slow and fast events. In a perfectly ideal network, this can be modeled a bit, and I wonder how far off these networks are. Can this give some insight into the mechanism as well? Katashima has a nice review a few years back in Polymer Journal, though I know some good work has been done since then.

Response: We thank the reviewer for their suggestion of modeling the stress relaxation behavior of these hydrogels. We have done new analysis where we have fitted the existing stress relaxation data of hydrogels containing mfCMPa-az in Figure 4 to the generalized three-mode Maxwell model with fitted parameters (Figure S16, Table S1). There may be at least three modes of relaxation in our PEG-mfCMP hydrogels based on the results of this model fitting. We hypothesize that faster modes may be associated with the relaxation of the triple helix or fibril mfCMP assemblies in response to a deformation, and slower modes may be associated with poroelastic effect of the network, with opportunities to test this mechanistic hypothesis of the origin of the modes in future studies.

Excerpts from revised text:

Page 17-18:

These data (**Figure S15**) were then fitted (**Figure S16**) with a generalized Maxwell model with three modes (**Equation S2**), a viscoelastic model that describes three stress relaxation events in parallel.[64] The fitted parameters describing the stress, A_i, and relaxation time, τ_i, for each were determined (**Table S1**). This model fitting suggests that there are at least three modes of relaxation in our PEG-mfCMP hydrogels. We hypothesize that faster modes may be associated with the relaxation of the triple helix or fibril mfCMP assemblies in response to a deformation, and slower modes may be associated with poroelastic effect of the network,[9] with opportunities to test this mechanistic hypothesis of the origin of the modes in future studies.

SI Page 10:

Fitting Stress Relaxation Data to the Generalized Maxwell Model: Stress relaxation curves from Figures 4 and S15 were fitted to the generalized Maxwell model described by Equation 2, producing Figure S16 and Table S1:

$$\sigma(t) = \varepsilon_0 \sum_{i=1}^{n} G_i e^{-t/\tau_i} \quad (2)$$

where $\sigma(t)$ is the sheer stress as a function of t time (s) normalized to maximum stress, ε_0 is the applied step shear strain, G_i is the modulus of mode i normalized to maximum stress, and τ_i is the relaxation time (s) of mode i. The following variables were defined: let A_i be equal to $\varepsilon_0 G_i$, the stress of mode i normalized to maximum stress, and let i = 3 modes. Fitting was done using the Microsoft Excel solver function. The objective was set to the sum of the squares of the measured normalized stresses for each timepoint, variables being changed were set to A_{1-3} and τ_{1-3} with the constraints of positive values, and solving method was set to GRG Nonlinear. Initial guesses for A_{1-3} and τ_{1-3} were set to 0.4, 0.35, 0.25, 200 s, 2000 s, and 20000 s, respectively.

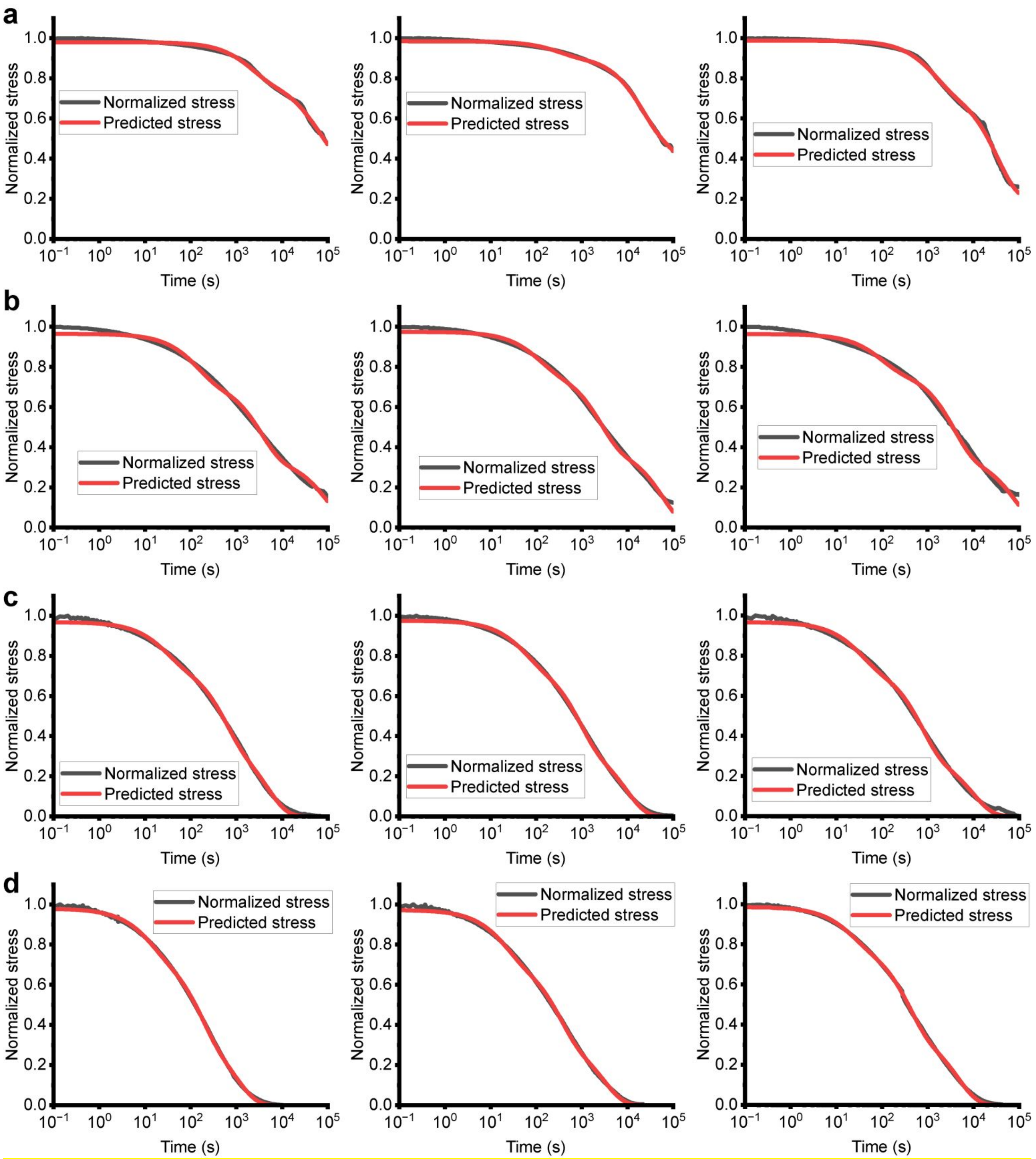

Figure S16. Stress relaxation curves containing mfCMPa-az at a) 5 mM, b) 9 mM, c) 13 mM, and d) 20 mM fitted to the generalized three mode Maxwell model.

Table S1. Fitted 3-mode Maxwell model parameters for hydrogels containing increasing concentrations of mfCMPa-az.

[mfCMPa-az] (mM)	A_1	A_2	A_3	τ_1	τ_2	τ_3
5	0.162 ± 0.048	0.390 ± 0.095	0.432 ± 0.12	1250 ± 520	31000 ± 5600	10300000 ± 980000
9	0.196 ± 0.018	0.406 ± 0.013	0.366 ± 0.017	111 ± 12	3140 ± 370	83100 ± 12000
13	0.200 ± 0.0093	0.417 ± 0.018	0.353 ± 0.023	36.8 ± 7.4	681 ± 88	6690 ± 1600
20	0.203 ± 0.017	0.421 ± 0.011	0.356 ± 0.010	17.7 ± 2.7	111 ± 64	2860 ± 1000

3. The results shown in Figure 5 are impressive and nicely show differences, but it looks to me like these are yet another formulation. Here the MMP degradable linkers remain constat, while Figure 4 shows that replacing MMP linkers with CMPs leads to the stress relaxation. In Figure 5 the number of covalent crosslinks is not changed, and more dynamic crosslinks are added, which also likely increases the moduli of the hydrogel. If I am missing something critical, then please try to make it more clear to the reader what the alignment of the experiments is.

Response: We thank the reviewer for their inquiry. The reviewer is correct in their assessment that in Figure 4, MMP degradable crosslinks are being replaced with physical mfCMP crosslinks, and that in Figure 5, covalent crosslinks are not changed. We made this choice of constant degradable linker for the cell experiments to ensure that the swollen storage moduli and MMP-degradability of these hydrogels were constant between conditions. We have performed new stress relaxation experiments in Figure S19 for the cell experiment gel formulations, showing that the mfCMP-containing formulations exhibit significantly more stress relaxation compared to the elastic formulation. Here, we show that even when the number of covalent crosslinks is held constant, adding physical mfCMP crosslinks imparts stress relaxation behavior to the hydrogel. Additionally, we have provided Tables S2-3 clarifying the different formulations used in this work.

Excerpts from revised text:

Page 22:

Further, each hydrogel composition (E, VF, VBF) was probed for viscoelastic behavior (**Figure S19**), where the VF and VBF formulations showed significantly greater stress relaxation than the E formulation over a period of 10^5 seconds. Additionally, these hydrogel compositions showed statistically similar swollen equilibrium storage moduli (**Figure S20**). Together these rheometric

measurements confirm that adding physical mfCMP crosslinks imparts stress relaxation behavior while modulus and crosslink density are held constant.

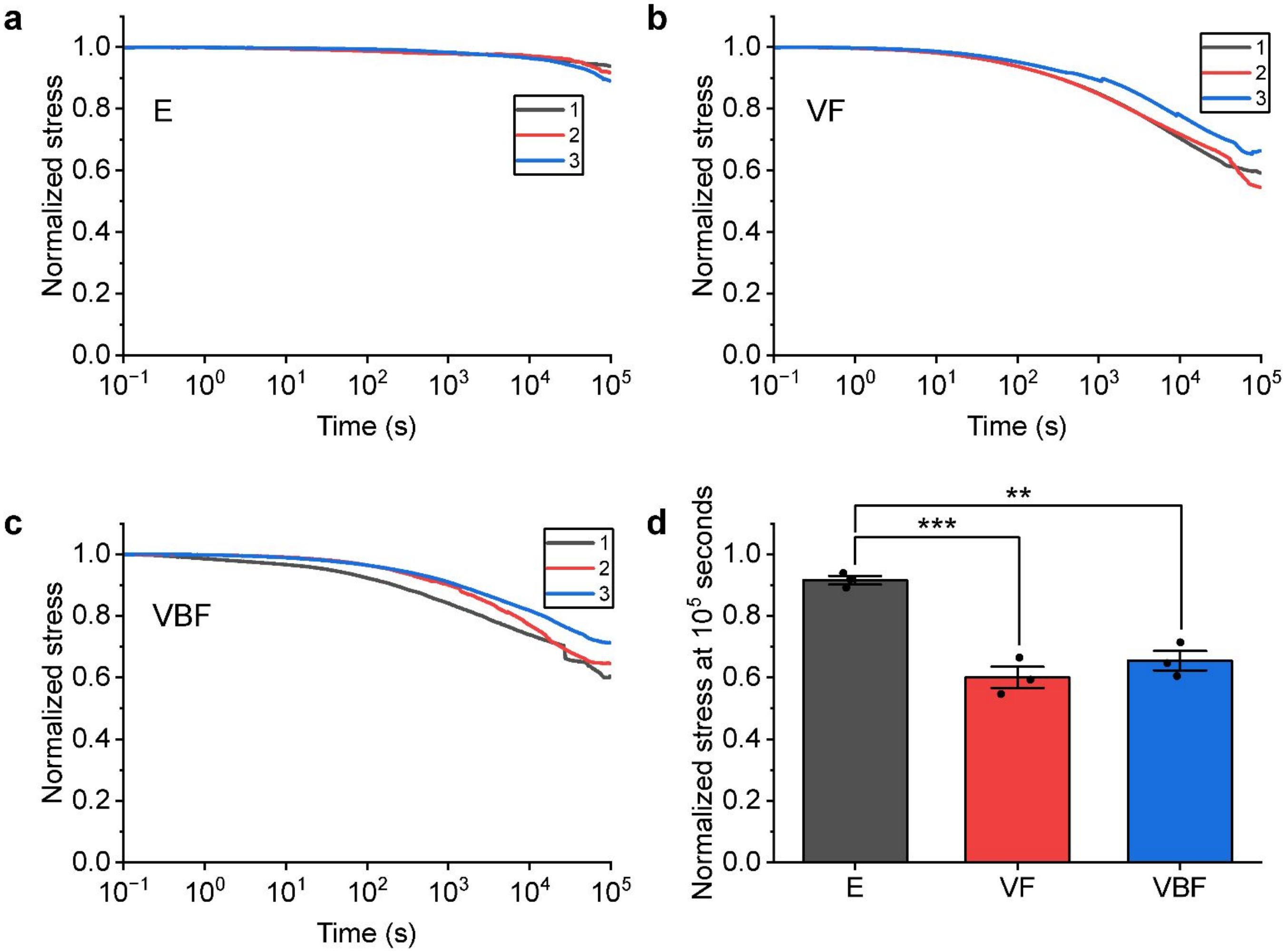

Figure S19. Stress relaxation behavior of hydrogel formulations used in cellular studies: a) E, b) VF, and c) VBF. Stress is normalized to the maximum stress measured for each sample. d) Comparison of average values for stress relaxation at 10^5 seconds for these compositions. Means ± standard error are shown for each condition for (n = 3) independent sample measurements. Statistical significance was determined by one-way ANOVA with Tukey's multiple comparisons test. Statistical significance is shown (*p < 0.05; **p < 0.01; ***p < 0.001; ****p < 0.0001).

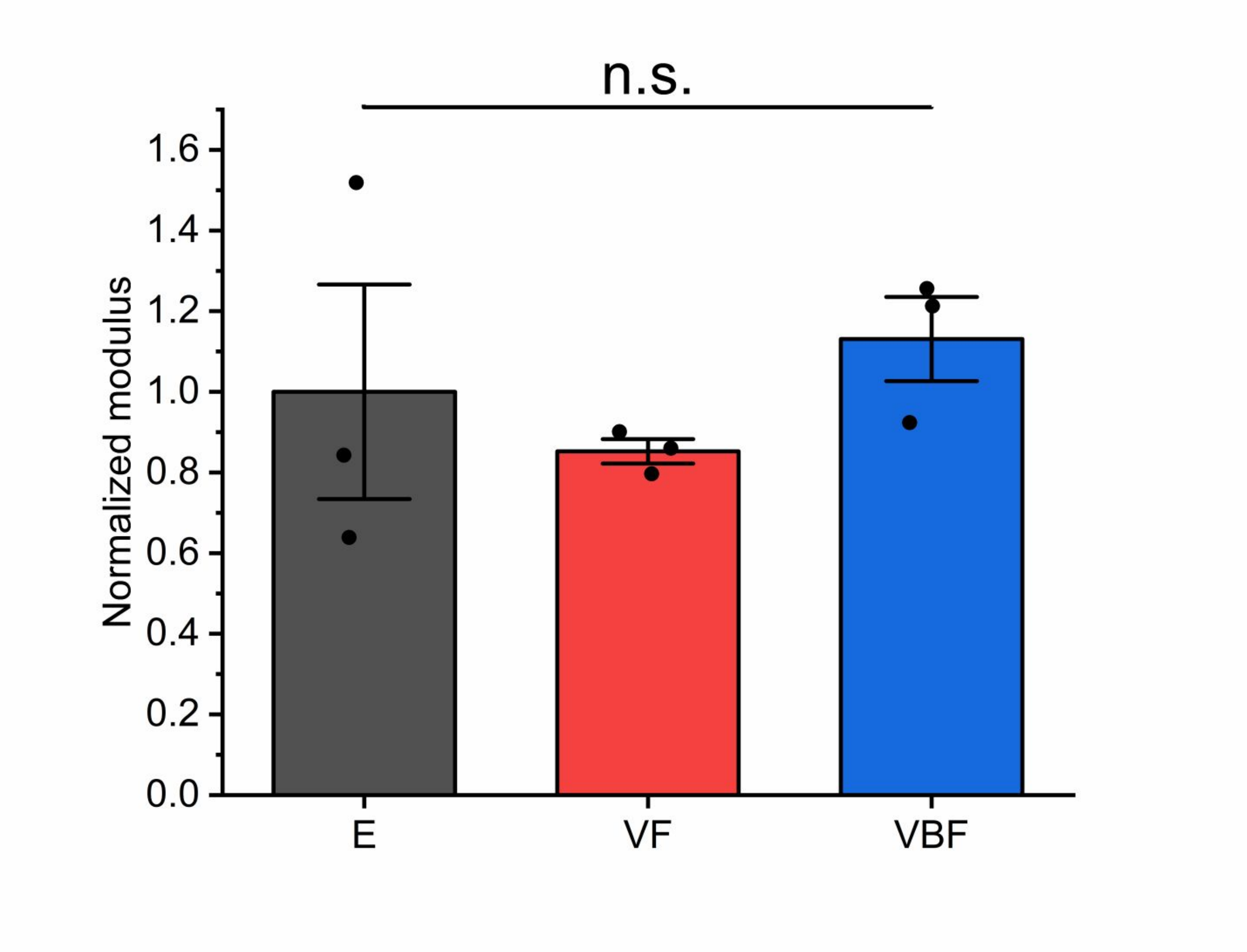

Figure S20. Equilibrium-swollen storage moduli of E, VF, and VBF hydrogel compositions, normalized to the E condition. Means ± standard error are shown for each condition for (n = 3) independent sample measurements. Statistical significance was determined by one-way ANOVA with Tukey's multiple comparisons test (n.s. = no statistical significance).

Table S2. Concentrations of monomers used to form hydrogels for in situ gelation time sweeps, frequency sweeps, strain sweeps, and stress relaxation experiments.

	0 mM	5 mM	9 mM	13 mM	20 mM
PEG (mM thiol)	20	20	20	20	20
Linker Peptide (mM alloc)	18	13	9	5	0
mfCMPa-az (mM alloc)	0	5	9	13	20
Pendent RGD (mM alloc)	2	2	2	2	0

Table S3. Concentrations of monomers used to form hydrogels for stress relaxation experiments.

	E	VF	VBF
PEG (mM thiol)	20	20	20
Linker Peptide (mM alloc)	13	13	13
mfCMPa-az (mM alloc)	0	5	3
mfCMPa-G-az (mM alloc)	0	0	1
mfCMPa-R-az (mM alloc)	0	0	1
Pendent RGD (mM alloc)	1	1	0
Pendent GFOGER (mM alloc)	1	1	0

4. There are some claims in the paper that are not supported by the data.

a. Page 11 line 28, "All mfCMPs showed a first melting event (Tm1) near 37 ˚C, indicating that around 50% of the triple helices remained intact at this temperature, like natural collagen I" Figure 2d does not support this claim based on the CD intensities and changes. If there is more data to support this claim, please make sure to point it out.

Response: We thank the reviewer for bringing this inadvertent oversight to our attention. After further quantitative analysis, we have determined that the percentage of assembled triple helices at 37 ˚C for mfCMPa-G-az, mfCMPa-R-az, and mfCMPa-az are as follows: 79.6 ± 0.211%, 47.5 ± 0.213%, and 46.2 ± 0.172%, respectively. These values were calculated by dividing the mean residue ellipticity at 37 ˚C by the difference between the maximum and minimum mean residue ellipticity for each melting plot. We also have performed further quantitative analyses of the melting temperatures for clarity as described our response to the next point (Figure 2, Figure S6).

Excerpts from revised text:

Page 10:

The percentage of assembled triple helices at 37 ˚C for mfCMPa-G-az, mfCMPa-R-az, and mfCMPa-az were estimated to be 79.6 ± 0.211%, 47.5 ± 0.213%, and 46.2 ± 0.172%, respectively. These values were calculated by dividing the mean residue ellipticity at 37 ˚C by the difference between the maximum and minimum mean residue ellipticities for each melting curve.

Minor:

1. There is a decent difference in Figure 2 on the melting behavior of the RGD vs COGEFR derivative. Any clue why? Or what this difference is?

Response: We thank the reviewer for their discussion and curiosity. We hypothesize that these differences are due to the inherent differences in triple helix stability between the two sequences. Specifically, since the mfCMPa-R-az has a P in the G position of the X-Y-G triplet polyproline-

like repeat peptide structure (GRGDS**P**), it is less stable compared to mfCMPa-G-az, where the analogous position has the G (FOGER**G**).

To better identify the melting events and describe the approach for reproducibility, we now have quantitatively identified melting events by analyzing the second order derivatives for the melting curves for all mfCMPs. The two points where the second order derivative plot crosses zero with the highest changes in magnitude are reported. Note, minor differences in the observed melting temperatures were observed relative to what was reported in the original manuscript. The text has been updated to reflect this quantitative analysis; Figure S6 has been updated to include these plots; and Figure 2 has been updated to reflect the melting temperatures identified with this analysis.

Excerpts from revised text:

Page 10:

The first- and second-order derivatives of these curves were then taken to identify inflection points as melting events (**Figure 2e-f**).

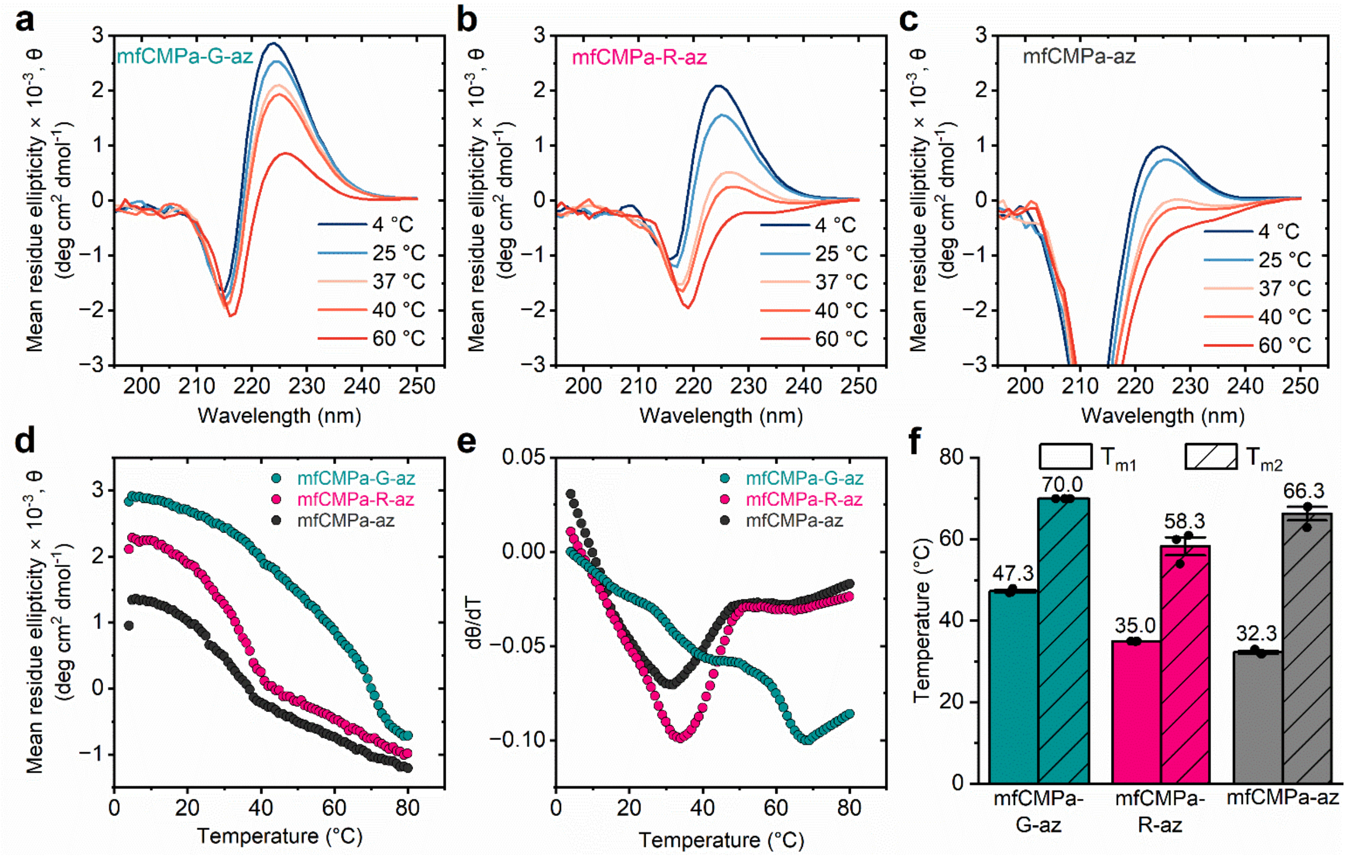

Figure 2. Triple helix characterization of mfCMPs in Dulbecco's phosphate-buffered saline by CD spectroscopy. Wavelength scans of a) mfCMPa-G-az, b) mfCMPa-R-az, and c) mfCMP-az as temperature is ramped from 4 ˚C to 80 ˚C show the characteristic polyproline type II peak (225 nm). d) Temperature scans of mfCMPs at 225 nm show nonlinear "melting" as temperature increases, indicating that triple-helical conformations are present. e) First-order derivative curves of d) show minima that describe melting events for mfCMP triple helices. f) Temperatures of melting events of mfCMPs. The two largest melting events are reported for each sequence as determined by the inflection points of (d), identified by analysis of second-order derivatives of the data in (d) (**Figure S6**). Results shown are from a representative sample of multiple trials ($n = 3$). All replicates are shown in **Figures S5-6**.

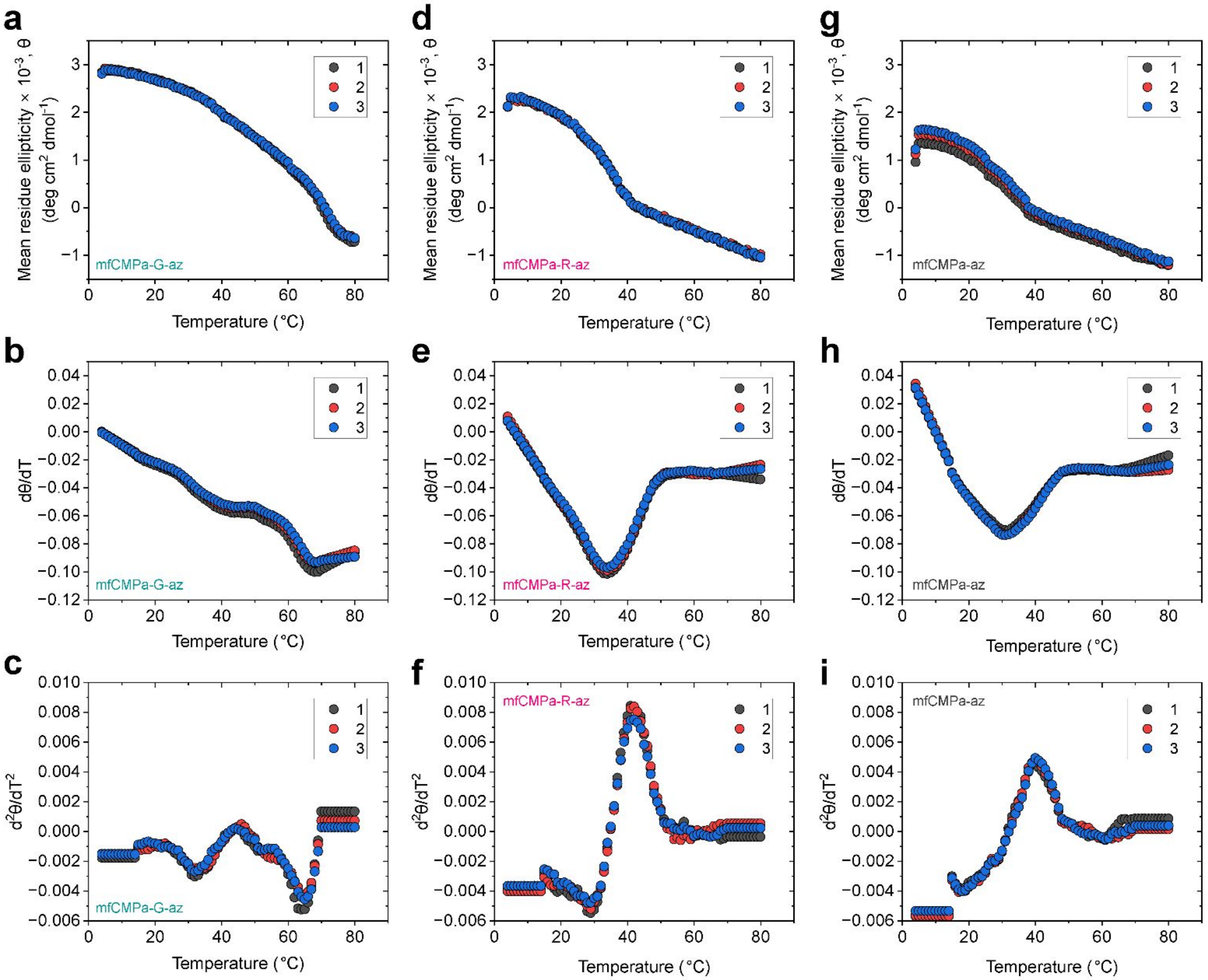

Figure S6. Temperature scans of mfCMPs at 225 nm with their first- and second-order derivatives. a-c) mfCMPa-G-az, d-f) mfCMPa-R-az, and g-i) mfCMPa-az in DPBS measured at 0.3 mM after assembly. For each, (n = 3) independent samples were measured. The two points where the second order derivative plot crosses zero with the highest changes in magnitude were identified and reported as the prominent melting temperatures (**Figure 2)**.

2. I am not sure I fully understand the hydrogel formulations. It seems that the formulation for the functionalized CMPs also contains K(alloc)GWGRGDS? So two different binding sites in the hydrogel (one in the helix, one pendant)?

Response: We thank the reviewer for their inquiry. The hydrogel formulations for the rheological characterization were made with the pendent RGD peptide, analogous to previous

studies where pendent peptides were included (Ford, et. al, J. Mat. Chem B., 2024). We hypothesize that the integrin binding peptide should not affect stress relaxation behavior as this type of viscoelasticity is dependent on mfCMP concentration, as described in our response to point 3. We have also included tables further clarifying all gel formulations as seen in our response to point 3 (Tables S2-3).

3. I do not see the supplier or structure of the alkyne fluorophores provided in the manuscript.

Response: We thank the reviewer for bringing this to our attention. We have added the supplier for the alkyne fluorophores in the manuscript. The structures of the fluorophores are not publicly available from the supplier.

Excerpts from revised text:

SI Page 7:
For imaging by confocal microscopy, AlexaFluor 488 (20 mM in water, ThermoFisher, Waltham, MA, Cat. No. A10267) was used. For super-resolution STORM imaging, AlexaFluor 647 (0.2 mM in water, ThermoFisher, Waltham, MA, Cat. No. A10278) was used for its high number of cycles and photon output per cycle and its high resistance to photobleaching.

4. The mechanism of CMP fiber slipping as a mechanism for stress relaxation is a good hypothesis, but I would argue that it is hard to prove. The CMP's could simply be disassociating and reassociating via sticky ends, no? As in the melting curves.

Response: We agree with the reviewer that the mechanism is hard to test. In the schematic, we hypothesize that the fibril slipping mechanism is the disassociating and reassociating via sticky ends. However, the melting curves are not a direct measurement of the sticky end formation, they are rather more directly measuring how ordered the polyproline type II helix is due to the triple helical conformation. We have clarified these points in the revised manuscript.

Excerpts from revised text:

Page 10:
For these assembling peptide sequences, these melting curves are measuring how ordered the polyproline type II helix is due to the triple helical conformation and are performed at lower concentrations (0.3 mM) than used in hydrogel formulations to avoid the formation of higher ordered structures that would scatter light and impede CD measurements (e.g., fibrils formed by sticky end interactions).

Page 16:

We hypothesized that hydrogels with mfCMPs would exhibit stress relaxation because of the collagen-like physical crosslinks between mfCMP peptides and fibrils (i.e., hydrogen bonding and sticky-end electrostatic interactions), allowing fibrils to slide past each other to dissipate stress induced by applied strain (**Figure 4a**). In the hydrogel, assembled mfCMPs are covalently crosslinked into the polymer network, and the mfCMPs then serve as physical crosslinks within the network owing to their physical interactions with each other via hydrogen bonding within triple helices and salt bridging within fibrils.

5. I wonder what the driving force for the cells to infiltrate into the upper hydrogel (the one experimentally being tested) are. How is it possible to have the cells only infiltrate upwards and the analysis be done only in the upper hydrogel. I could understand if the bottom was not degradable; however, in this setup it is. Both a scientific and a technical question. It is a neat setup, but curious how it works and how data analysis is done here.

Response: We would like to clarify that the cell studies in this work were encapsulated within the synthetic matrix during hydrogel formation: the cells were homogenously mixed with the hydrogel precursor solution, then crosslinked on top of a previously crosslinked cell-free hydrogel, resulting in 3D hydrogel-cell construct with a top cell-laden layer and a cell-free bottom layer. This layered geometry has been previously used to allow observations of homogenous responses of metabolically active cancer cells in the 3D culture while imparting easy handling (Sawicki, et. al., APL Bioeng., 2019; Pradhan, et. al., Sci. Adv., 2023). We have added a schematic of this gel formation process as Figure S18 to clarify. Infiltration from the top cell-laden layer into the bottom cell-free layer was not seen in any of the hydrogel replicates throughout all conditions. We generally do not expect this epithelial-like cell type to migrate or infiltrate significantly. We have also included videos in the SI showing minimal migration from 8-22 hours after the encapsulation.

Excerpts from revised text:

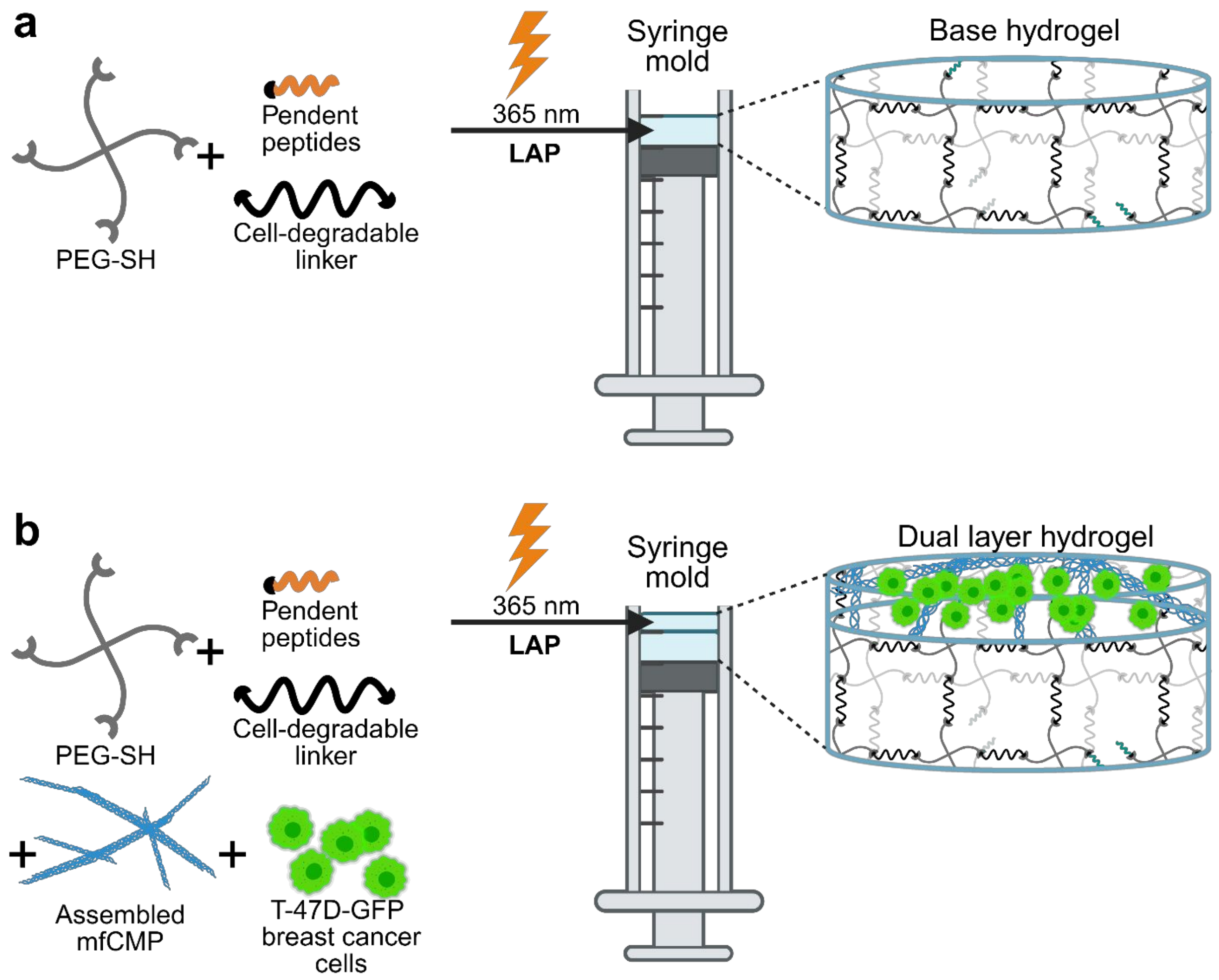

Figure S18. Schematic showing how dual-layer hydrogels were made for cell encapsulation experiments. a) First, PEG-SH, any pendent peptides, cell degradable linker peptide, and LAP are mixed and added to a syringe mold, then photocrosslinked. b) Next, the same monomer components are mixed and added to lyophilized mfCMP, then added to a cell pellet. After mixing again, this precursor solution is added to the same syringe mold containing the base layer, and photocrosslinked.

6. Were any difference other than cluster size noticed in the cells?

Response: We thank the reviewer for their inquiry. After analyzing the cell counts per field from existing images, we have concluded that increased proliferation allows the cells to form growing clusters. VBF and VF conditions show statistically higher counts per field compared to E (Figure 5f), consistent with trends observed in Ki-67. We did not observe differences in cell volume within the clusters between conditions (Figure S25). We also have performed new β1 integrin staining showing differences in the VBF condition as described in our response to question 1.

Excerpts from revised text:

Page 23:

Cells in the viscoelastic hydrogel formulations trended toward a greater percentage of Ki-67 positive (%Ki-67^{+ve}) cells, although no statistical differences were observed. Importantly, consistent with this trend, we observed that the VF and VBF conditions both had significantly higher numbers of cells per field (**Figure 5f**), suggesting that cluster volume arises from increased proliferation in the viscoelastic hydrogel formulations with mfCMP. Further, we did not observe differences in cell volume (e.g., cells taking up more space owing to synthetic matrix viscoelasticity[5]) (**Figure S25**) or motility (e.g., cells migrating to form larger clusters[37, 73]) (**Videos S1-9**).

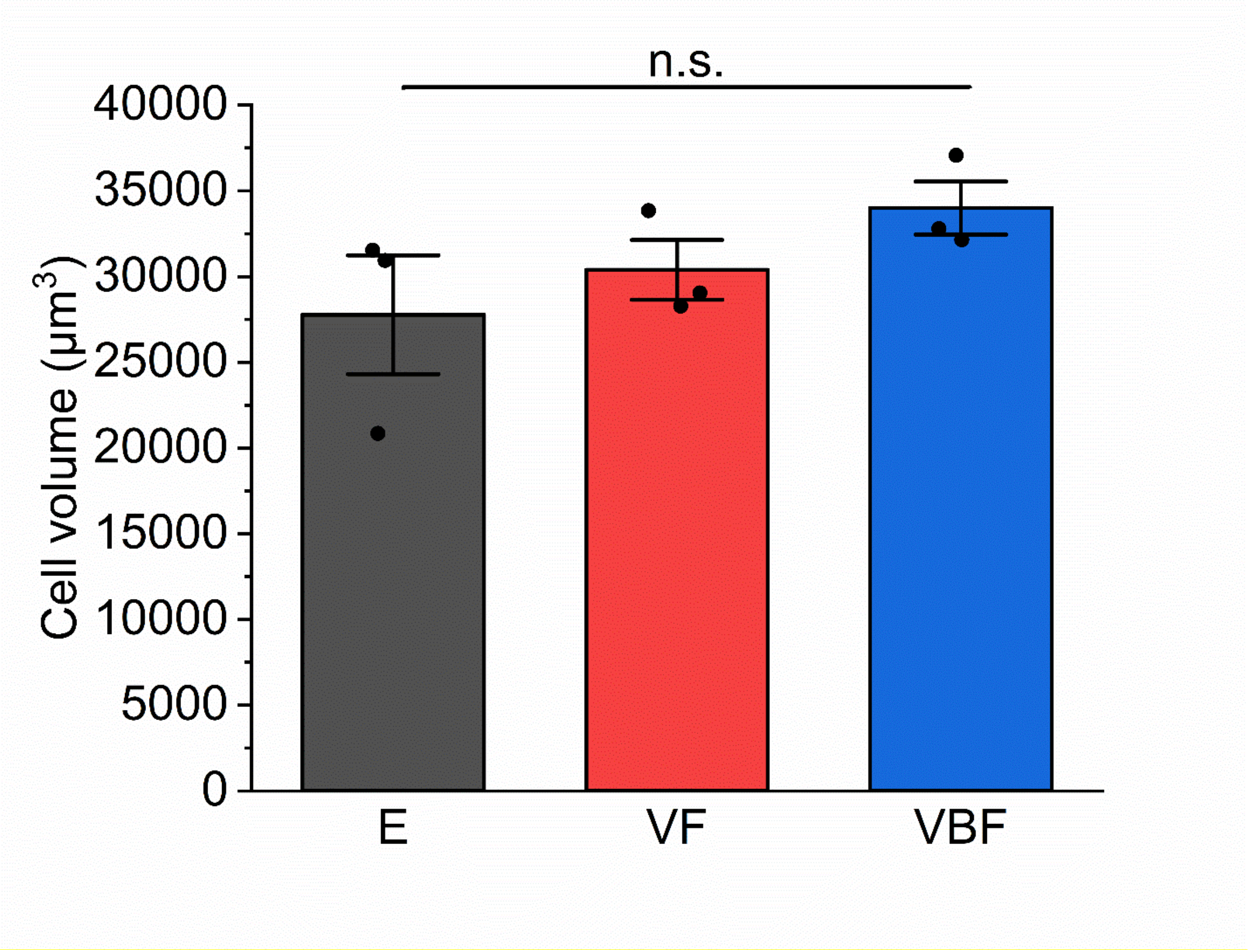

Figure S25. Comparison of mean cell volume for T47Ds in mfCMP-PEG hydrogels (conditions E, VF, VBF) on day 7 based on F-actin fluorescence. Means ± standard error are shown for each condition for (n = 3) independent averaged sample measurements. Statistical significance was

determined by one-way ANOVA with Tukey's multiple comparisons test. Statistical significance is shown (*$p < 0.05$; **$p < 0.01$; ***$p < 0.001$; ****$p < 0.0001$).

7. The cell cluster size from day 1 to day 7 seems to follow the same trend. Is there a chance that the seeding methods or viscosity of the gel precursors themselves led to this difference as well? Meaning an indirect effect?

Response: We thank the reviewer for their suggestions. The encapsulation methods for all conditions were the same where the gel precursor solution was pipette-mixed with the cell pellet, so we do not anticipate that there were differences during this process. To further answer the reviewer's question, we have included additional videos as described in our response to question 5, visualizing the encapsulated cells over-time during hours 8-22 following encapsulation. Here, we do not observe qualitative differences in cluster shape or size between conditions. However, we hypothesize that the differences in cluster growth, as described in the manuscript, are due to higher proliferation in response to the viscoelasticity and integrin presentation imparted by the mfCMPs.

Excerpts from revised text:

Page 22:
From the real-time videos following the encapsulation, we observe little migration for this epithelial-like luminal A breast cell type, which is expected for these spheroid-forming, weakly-metastatic cells.

oc-2025-021753.R2

Name: Peer Review Information for "Synthetic Surrogates of Collagen-Rich Microenvironments: Integrating Modular Bioactive Fibrillar Structure and Tunable Viscoelasticity via Multifunctional Assembling Peptides"

Second Round of Reviewer Comments

Reviewer: 4

Comments to the Author

Thanks to the authors for a very clear an thorough consideration of the reviewer's comments. Also very well crafted response with clear changes highlighted and figures presented.

Reviewer: 1

Comments to the Author

The authors have done a nice job addressing my comments and revising the manuscript.

Reviewer: 2

Comments to the Author

The authors have addressed the reviewers' concerns.

Reviewer: 3

Comments to the Author

I have reviewed the revised manuscript and the authors' point-by-point response. Overall, the revisions satisfactorily address my prior comments, notably, validation that the pendant GFOGER control forms a stable triple helix by CD, addition of stress-relaxation data for the specific E/VF/VBF cellular formulations, and clarification of covalent versus physical (reversible) mfCMP interactions.

I recommend acceptance of the revised manuscript.

Author's Response to Peer Review Comments:

RESUBMISSION RESPONSE

Editor:

Comments:
Thank you for your recent submission to ACS Central Science. We have now received the reviews for your manuscript and I am pleased to inform you they were quite positive.

However, there remain important points that require attention and must be addressed before a final decision can be made. Please address these comments before submitting a second revised version.

One of the key issues raised by the reviewers concerns the existence of multiple peaks in the HPLC traces shown in the SI which imply that the peptides that are the focus of the study are not pure. Although this issue is addressed in the revision, in my view the additional statements added are confusing and do not do much to address the points raised by the reviewers. For example, in Figures S1 and S3, multiple peaks are clearly present in the HPLC traces. In the response, these features are attributed to aggregation or to peptides with missing residues; however, this interpretation would benefit from further clarification and supporting evidence. The issue is not well addressed in the revised paper, or in the captions to the figures. The lead author needs to engage in resolving this problem, so that it is transparently addressed in the 2nd revised version.

Response: We thank the editor for his valuable feedback and consideration of our revised manuscript. As the Editor noted, the existence of multiple peaks in Figures S1 and S3 implies that the peptides are not completely pure, and we agree that further explanation and discussion are needed.

While not 100% pure, the peptides are predominantly the desired sequences with reproducible workflows and consistent and well characterized properties for rigor and reproducibility. We appreciate the opportunity to clarify this as described below and in the revised main text and SI.

Additionally, from the feedback of the reviewers and editor, we appreciate the confusion created by the way the data were originally presented, with each SI Figure presenting a type of characterization for specific classes of peptides (mfCMPs, non-mfCMP). We accordingly have revised these figures to present all of the data for an individual peptide in an individual SI Figure. Figures S1-S4 are now reorganized and revised into new Figures S1-S6.

For further clarification regarding the mfCMPs in original Figure S1, multiple peaks were present in the UPLC traces in Figure S1 mainly due to amino acid residue deletions as

shown in the associated MS in Figure S2. Specifically, one common side product is the PO deletion, where one group of adjacent P and O residues are not coupled during synthesis of these long sequences with multiple (POG) repeats as previously reported for mfCMP designs (e.g., Ford et al., *ACS Biomaterials Science & Engineering*, 2021). Other minor impurities also are observed that are not easily identifiable, which we speculate are due to side chains of amino acid residues that react during the lengthy synthesis and purification process. Similarly, non-mfCMP peptides had minor amino acid deletions in original Figures S3-4, where their identities have been clarified in revised SI figures.

To attempt to separate the desired peptide from these impurities, we used a slow gradient during HPLC purification (~0.7 % ACN/min) along with a column heater set to 65 ˚C. As noted in response to Reviewer 1's comment of in the first resubmission, two slower HPLC gradients were tried to separate out these impurities and quantify peptide purity. However, we found that these slower gradients did not lead to further separation, and the total elution time of the peptide remained constant between different gradients (roughly 2 minutes). These data are now in the SI as Figure S7. Based on these observations, we speculated that the challenges in purification were due to assembly / aggregation of the peptides during the purification.

Based on the Editor's feedback, we have performed new DLS experiments on the mfCMPs used in this work, as shown in new Figure S8. Here, we see multiple broad peaks across many orders of magnitude of size, showing different size scales of aggregates in the solvent used for HPLC purification at both 25 ˚C and 65 ˚C. We hypothesize that peaks below 10 nm in size correspond to the triple helix, while peaks at larger sizes correspond to larger assembled structures / aggregates.

Despite the challenges that this aggregation presents during purification, we observe that 1) the majority product is the desired mfCMP molecular weight via UPLC-MS and that 2) these mfCMPs reproducibly assemble into triple helices and fibrils, tune the physical properties of hydrogels built with them, and control cellular behavior as described in the manuscript.

Broadly, to clarify the workflows and purities of the peptides, we have remade Figures S1-4 (mfCMPs and non-mfCMPs) into separate figures for each peptide in the SI. Here we aimed to isolate the m/z values in each separate 'peak' (e.g., shoulders) observed within the UPLC trace as well as show the total combined m/z values for the UPLC trace. In these updated figures, we also included HPLC traces from the purification workflow to show which individual fraction was used for the characterization and experiments in this work.

Excerpts from revised text:

SI Figures:

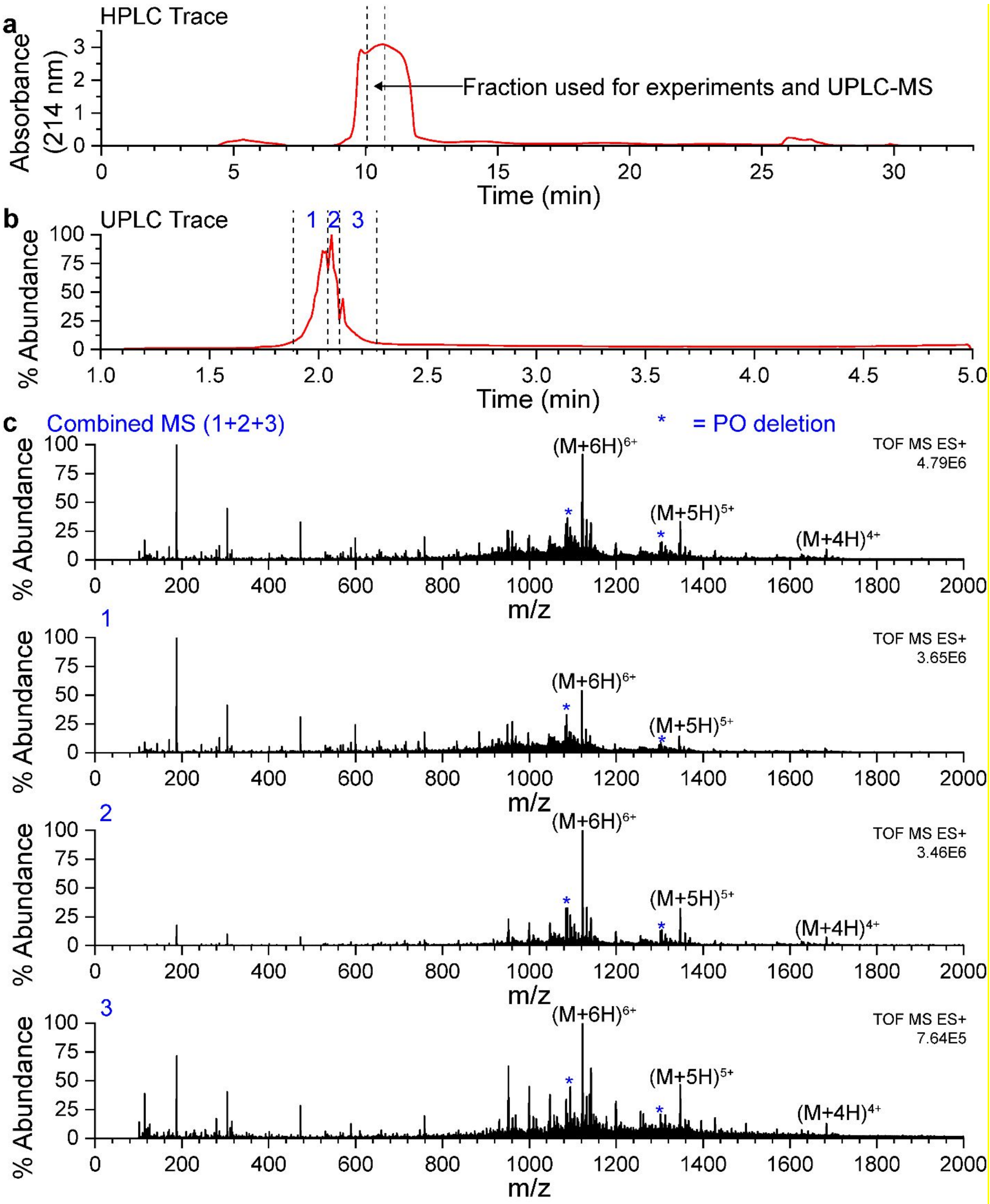

Figure S1. Purification and characterization of mfCMPa-G-az. a) Absorbance channel (214 nm) during HPLC purification showing collection time (10.0 to 10.8 min) of the fraction used for all experiments in this work. The peptide eluted from 23.9 to 24.9% acetonitrile (9.5 to 12.0 min) over a gradient of 0.7% acetonitrile per minute (18% to 30%). b) UPLC-MS chromatograms for purified peptide showing c) MS integration for the UPLC trace followed by separate MS integration for peaks within UPLC trace. Note, in addition to the prominent product peaks, some lower molecular weight species were observed that were unable to be separated by HPLC purification; these largely are minor residue deletions that are not observed to affect downstream properties batch-to-batch.

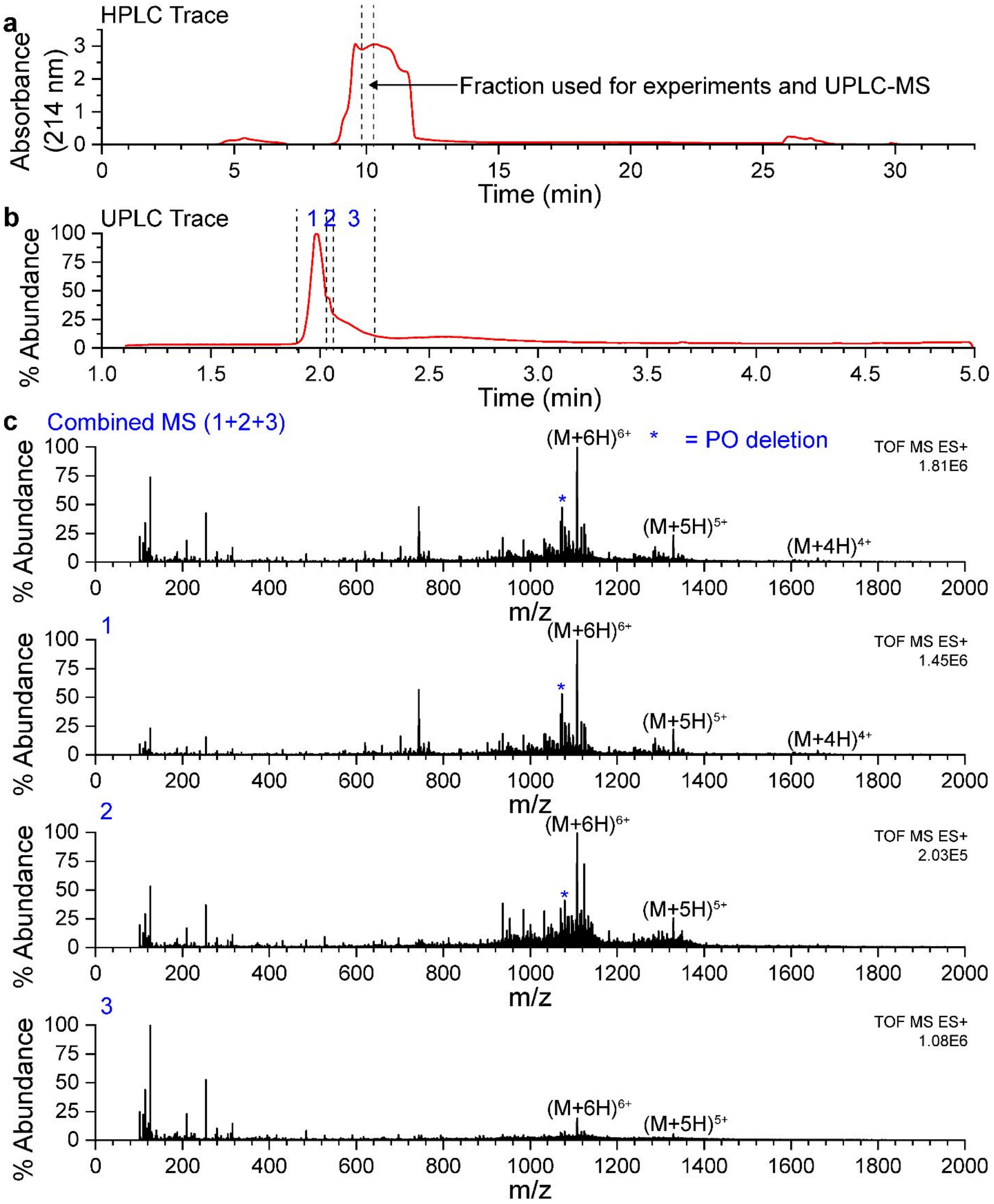

Figure S2. Purification and characterization of mfCMPa-R-az. a) Absorbance channel (214 nm) during HPLC purification showing collection time (9.9 to 10.2 min) of the fraction used for all experiments in this work. The peptide eluted from 23.5 to 24.9% acetonitrile (9.0 to 12 min) over

a gradient of 0.7% acetonitrile per minute (18% to 30%). b) UPLC-MS chromatograms for purified peptide showing c) MS integration for the UPLC trace followed by separate MS integration for peaks within UPLC trace. Note, in addition to the prominent product peaks, some lower molecular weight species were observed that were unable to be separated by HPLC purification; these largely are minor residue deletions that are not observed to affect downstream properties batch-to-batch.

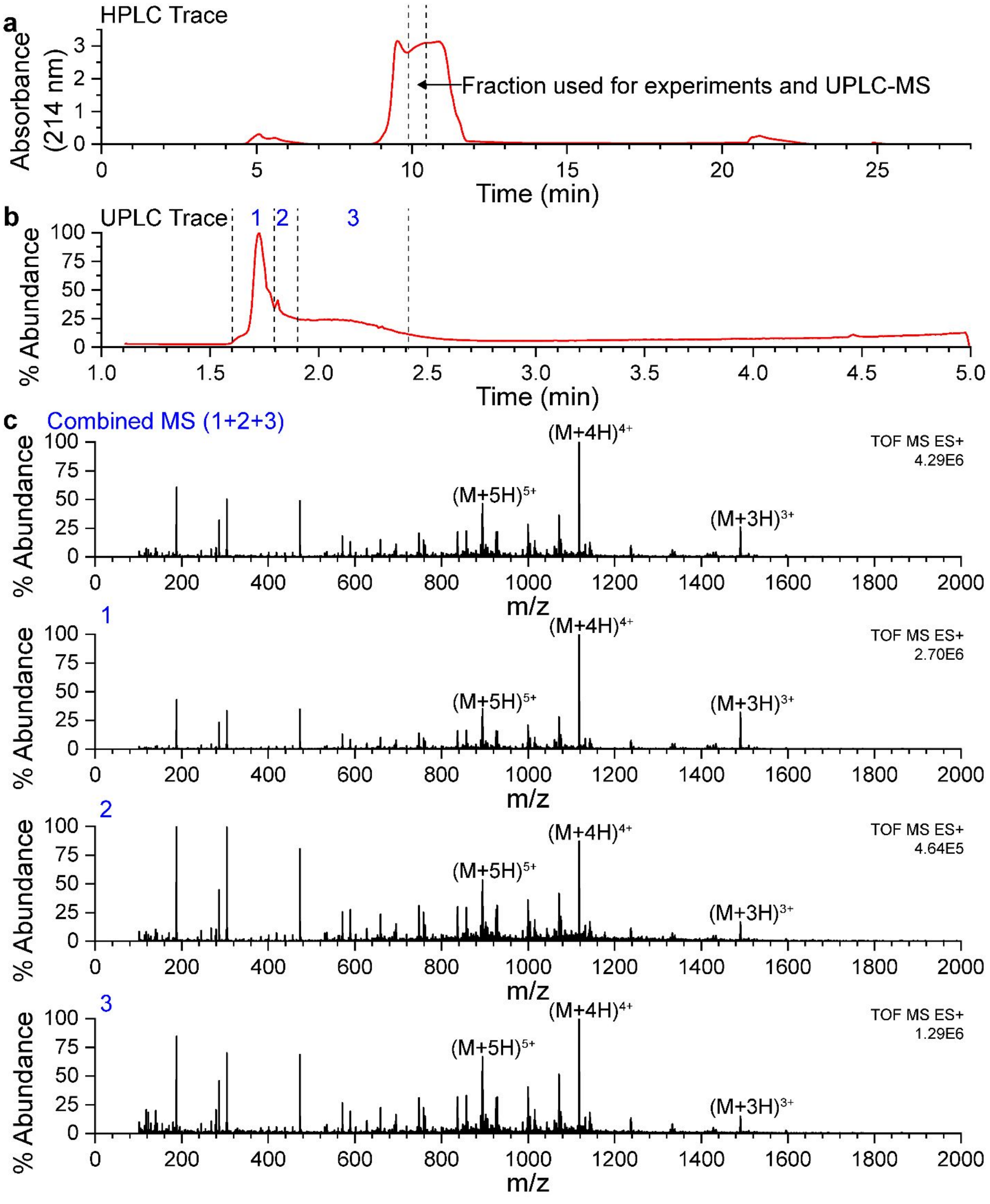

Figure S3. Purification and characterization of mfCMPa-az. a) Absorbance channel (214 nm) during HPLC purification showing collection time (9.9 to 10.3 min) of the fraction used for all experiments in this work. The peptide eluted from 24.2 to 26.4% acetonitrile (9.0 to 11.7 min) over

a gradient of 0.83% acetonitrile per minute (20% to 30%). b) UPLC-MS chromatograms for purified peptide showing c) MS integration for the UPLC trace followed by separate MS integration for peaks within UPLC trace. Note, in addition to the prominent product peaks, some lower molecular weight species were observed that were unable to be separated by HPLC purification; these largely are minor residue deletions that are not observed to affect downstream properties batch-to-batch.

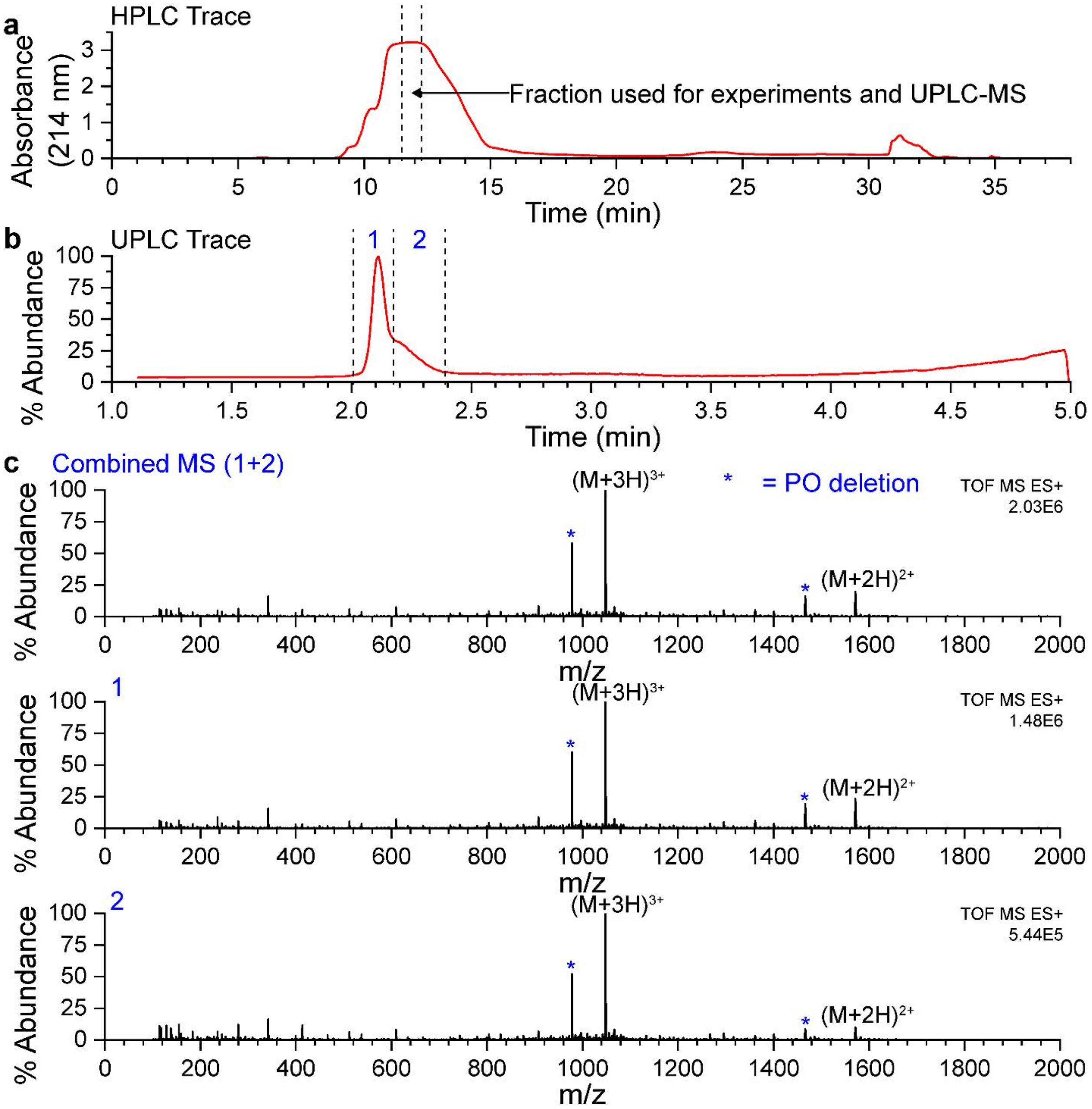

Figure S4. Purification and characterization of pendent GFOGER peptide. a) Absorbance channel (214 nm) during HPLC purification showing collection time (11.3 to 12.2 min) of the fraction used for all experiments in this work. The peptide eluted from 20.4 to 23.3% acetonitrile (10-14 min) over a gradient of 0.73% acetonitrile per minute (16% to 32%). b) UPLC-MS chromatograms for purified peptide showing c) MS integration for the UPLC trace followed by separate MS integration for peaks within UPLC trace. Note, in addition to the prominent product peaks, some lower molecular weight species were observed that were unable to be separated by HPLC purification; these largely are minor residue deletions that are not observed to affect downstream properties batch-to-batch.

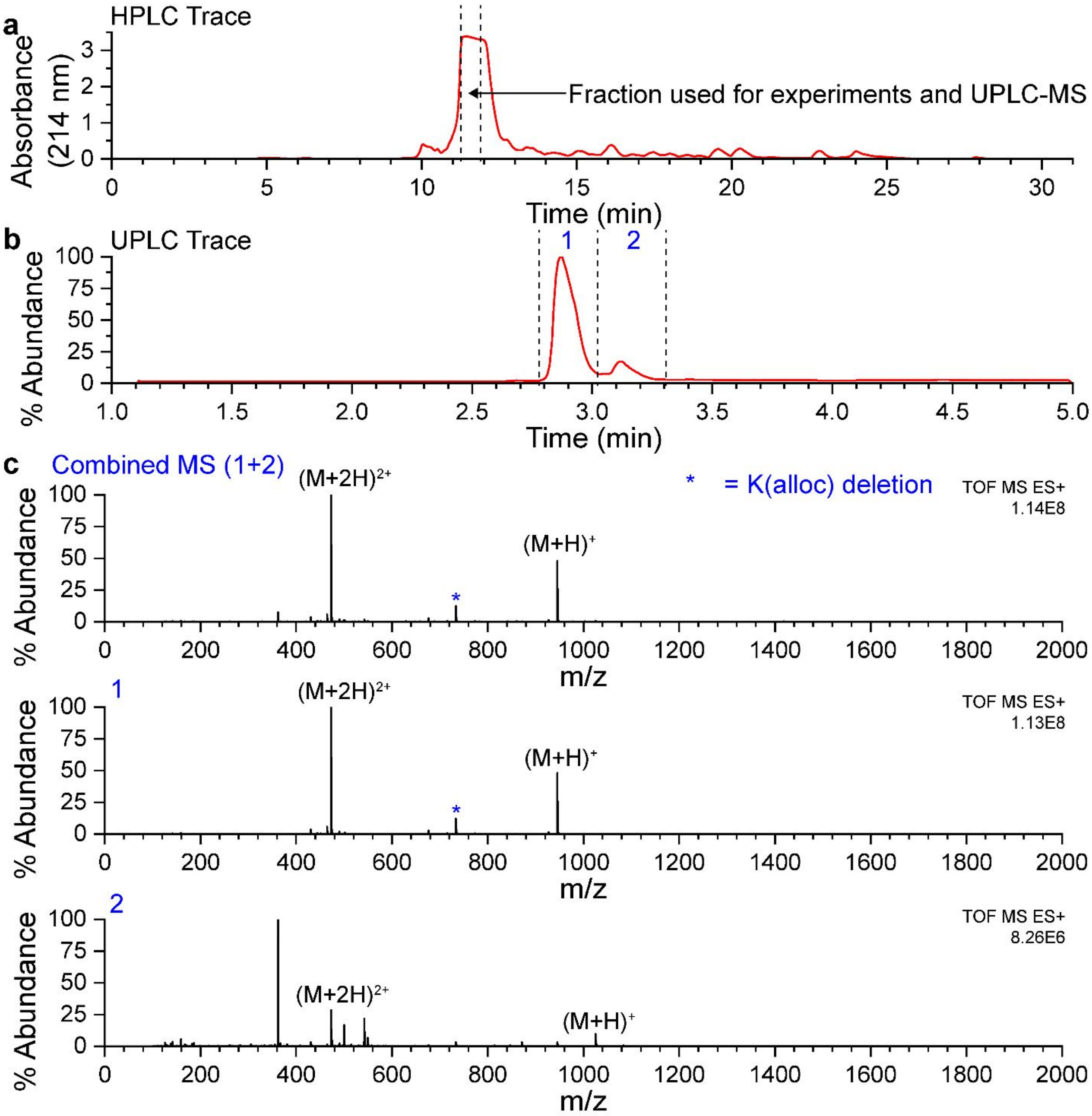

Figure S5. Purification and characterization of pendent RGD peptide. a) Absorbance channel (214 nm) during HPLC purification showing collection time (11.2 to 11.8 min) of the fraction used for all experiments in this work. The peptide eluted from 26.5 to 28.5% acetonitrile (10.5 to 12.5 min) over a gradient of 1% acetonitrile per minute (20%-35). b) UPLC-MS chromatograms for purified peptide showing c) MS integration for the UPLC trace followed by separate MS integration for peaks within UPLC trace. Note, in addition to the prominent product peaks, some lower molecular

weight species were observed; these largely are minor residue deletions that are not observed to affect downstream properties batch-to-batch.

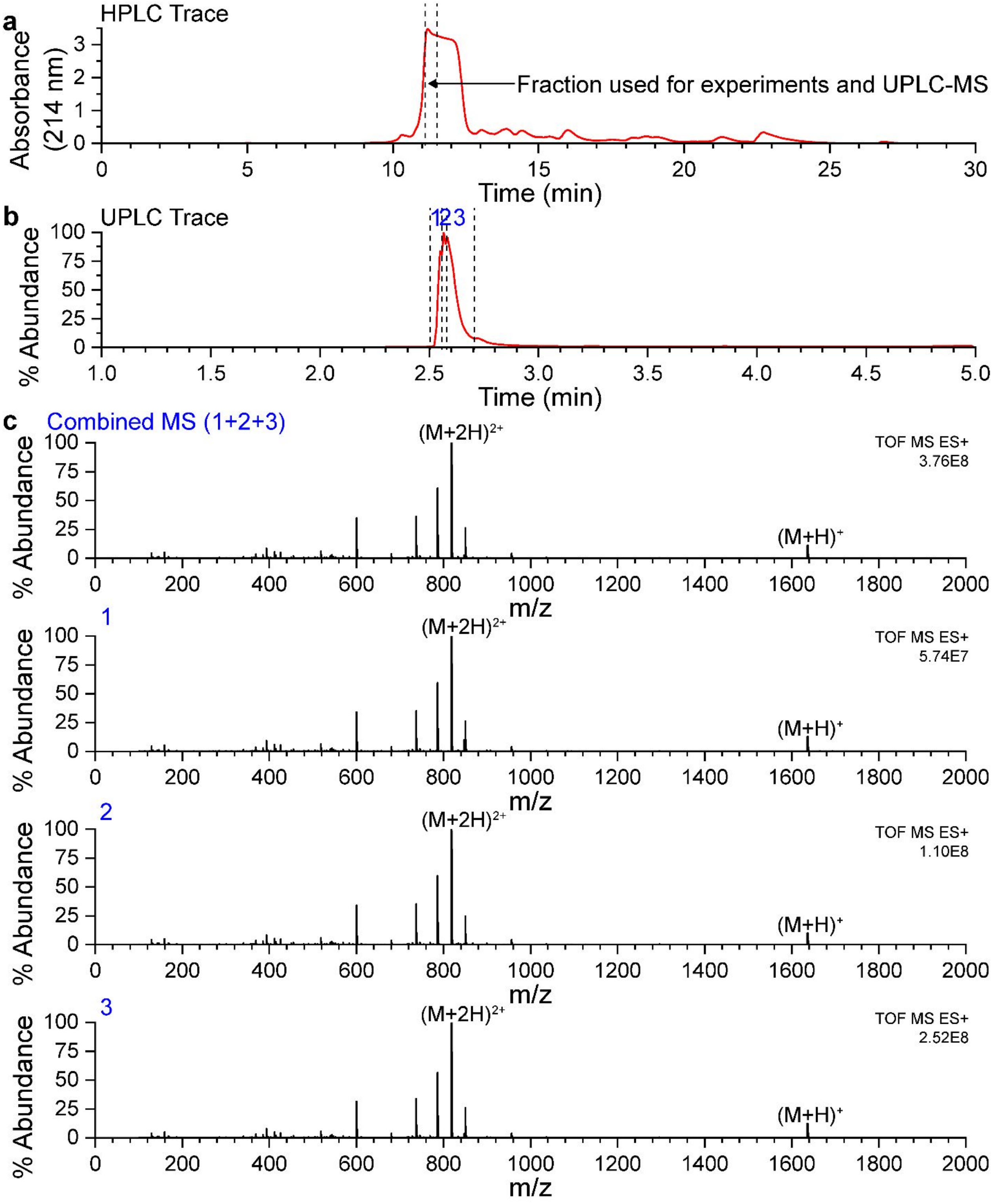

Figure S6. Purification and characterization of degradable linker peptide. a) Absorbance channel (214 nm) during HPLC purification showing collection time (11.1 to 11.5 min) of the fraction used for all experiments in this work. The peptide eluted from 29.0 to 30.1% acetonitrile (10.8 to 12.5 min) over a gradient of 0.6% acetonitrile per minute (25% to 33%). b) UPLC-MS chromatograms for purified peptide showing c) MS integration for the UPLC trace followed by separate MS integration for peaks within UPLC trace. Note, in addition to the prominent product peaks, some lower molecular weight species were observed; these largely are minor residue deletions that are not observed to affect downstream properties batch-to-batch.

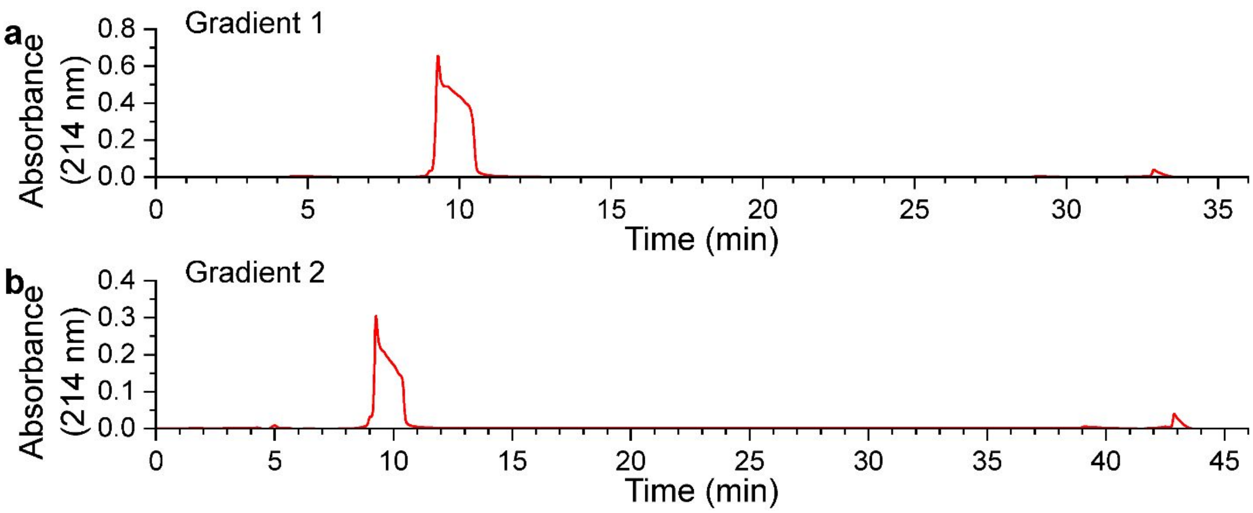

Figure S7. HPLC traces of mfCMPa-R-az at slower gradients. a) Gradient 1: 22 to 23.7% acetonitrile over 20 minutes (4 to 24 min; 0.085% acetonitrile per min). b) Gradient 2: 23.4 to 23.7% acetonitrile over 30 minutes (4 to 34 min; 0.01% acetonitrile per min). Using slower gradients did not change peptide elution time (9 min to 11 min) or separation.

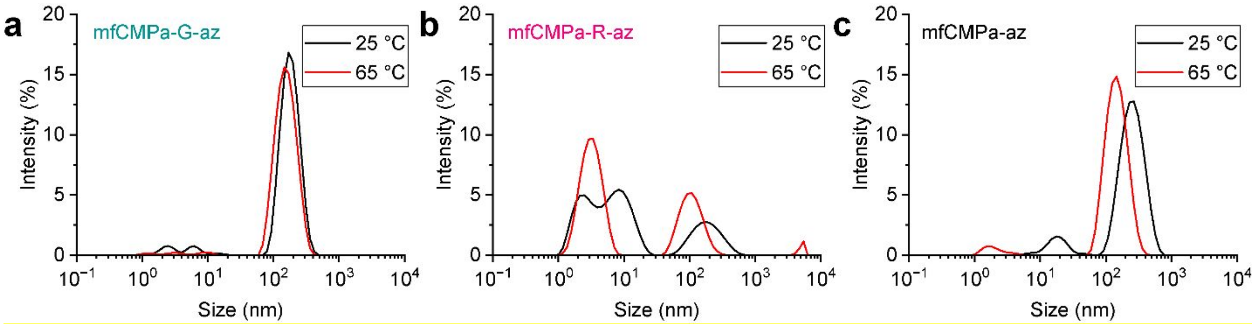

Figure S8. DLS measurements of mfCMPs in 95/5% water/acetonitrile at 25 ˚C and 65 ˚C. a) mfCMPa-G-az b) mfCMPa-R-az, and c) mfCMPa-az. All mfCMPs show multiple broad peaks

across many orders of magnitude of size, demonstrating aggregation in the solvent used during HPLC purification despite heating of the column. We hypothesize that peaks below 10 nm in size correspond to the triple helix, while peaks at larger sizes correspond to larger assembled structures or aggregates. For each peptide, independent samples ($n = 3$) were measured and averaged.

Comments:

1) In SI figure captions and text, it is repeatedly stated: "Note, in addition to the prominent product peaks, some _lower molecular species_ were observed…"
This should be changed everywhere this statement is made to read "lower molecular weight species" if this is the intended meaning.

Response: We thank the editor for the feedback in identifying this inadvertent typographical error in the SI figure captions. The statement is meant to say "lower molecular weight species" as the editor noted. All figure captions and SI text have been changed accordingly.

Excerpts from revised text:

SI page 3:

Ultra-performance liquid chromatography-tandem mass spectrometry (UPLC-MS, Xevo G2-S QTof; Waters, Milford, MA) was used to confirm peptide identity. Note, in addition to the prominent product peaks, some lower molecular weight species were observed that were unable to be separated by HPLC purification; these largely are minor residue deletions that are not observed to affect downstream properties batch-to-batch.

Captions, SI Figures S1-6:

Note, in addition to the prominent product peaks, some lower molecular **weight** species were observed that were unable to be separated by HPLC purification; these largely are minor residue deletions that are not observed to affect downstream properties batch-to-batch.

2) The captions to Figures S1 and S3 refer to chromatograms at specific times or solvent compositions, yet these times do not at all coincide with the x-axis in the figures (which uniformly show times from 1 – 5 minutes). I assume the listed retention times correspond to the previous HPLC separation, but the way it is presented in the UPLC captions is confusing. It would be better if the impurity/multiple peak issue is addressed in the main text where the synthesis and characterization is provided (e.g. p. 8 of the .pdf).

Response: We thank the editor for his feedback and agree that the description of HPLC elution times and solvent compositions in the captions of UPLC figures was confusing. As described above, we have remade these SI figures to include the HPLC traces from the purification workflow in addition to the UPLC-MS data for individual purified peptides that were collected. We have also updated the main text with discussion of the impurity and multiple peak issue.

Excerpts from revised text:

Page 7:

Note, while we observe consistent properties batch to batch as detailed below, multiple peaks were observed in UPLC traces of these peptides because of minor impurities due to amino acid residue deletions. Specifically, one common side product is the PO deletion, where one group of adjacent P and O residues are not coupled during synthesis of these long sequences with multiple (POG) repeats as previously reported for mfCMP designs.[41] Other minor impurities also are observed that are not easily identifiable, which we speculate are due to side chains of amino acid residues that react during the lengthy synthesis and purification process. To attempt to separate the desired mfCMP peptides from these impurities, we used a slow gradient during HPLC purification (~0.7 % acetonitrile per min) along with a column heater set to 65 ˚C. While slower HPLC gradients were tried after this, further separation was not achieved (**Figure S7**), and we speculated that the challenges in purification were due to assembly or aggregation of the peptides. To probe this, dynamic light scattering (DLS) measurements were performed on mfCMPs dissolved in HPLC solvent (**Figure S8**), and different size scales of aggregates were observed at both 25 ˚C and 65 ˚C. Despite the challenges that this aggregation presents during purification, we observe that the majority product is the desired mfCMP molecular weight via UPLC-MS, and these mfCMPs reproducibly assemble into triple helices and fibrils and tune the properties of hydrogels built with them, as described below.

Page 13:

Linker and pendent peptides were purified by HPLC and characterized by UPLC-MS (**Figures S4-6**). Note these non-mfCMP peptides show some minor impurities as described above for the mfCMPs.
